# A Screen Identifies the Oncogenic Micro-RNA miR-378a-5p as a Negative Regulator of Oncogene-Induced Senescence

**DOI:** 10.1371/journal.pone.0091034

**Published:** 2014-03-20

**Authors:** Susanne Marije Kooistra, Lise Christine Rudkjær Nørgaard, Michael James Lees, Cornelia Steinhauer, Jens Vilstrup Johansen, Kristian Helin

**Affiliations:** 1 Biotech Research and Innovation Centre, University of Copenhagen, Copenhagen, Denmark; 2 Centre for Epigenetics, University of Copenhagen, Copenhagen, Denmark; 3 The Bioinformatics Centre, Department of Biology, University of Copenhagen, Copenhagen, Denmark; 4 The Danish Stem Cell Center (DanStem), University of Copenhagen, Copenhagen, Denmark; IRCCS-Policlinico San Donato, Italy

## Abstract

Oncogene-induced senescence (OIS) can occur in response to hyperactive oncogenic signals and is believed to be a fail-safe mechanism protecting against tumorigenesis. To identify new factors involved in OIS, we performed a screen for microRNAs that can overcome or inhibit OIS in human diploid fibroblasts. This screen led to the identification of miR-378a-5p and in addition several other miRNAs that have previously been shown to play a role in senescence. We show that ectopic expression of miR-378a-5p reduces the expression of several senescence markers, including p16^INK4A^ and senescence-associated β-galactosidase. Moreover, cells with ectopic expression of miR-378a-5p retain proliferative capacity even in the presence of an activated Braf oncogene. Finally, we identified several miR-378a-5p targets in diploid fibroblasts that might explain the mechanism by which the microRNA can delay OIS. We speculate that miR-378a-5p might positively influence tumor formation by delaying OIS, which is consistent with a known pro-oncogenic function of this microRNA.

## Introduction

Senescence is a permanent exit from the cell cycle that can be driven by different cellular and environmental signals. Critical shortening of telomeres, oxidative stress, DNA damage and aberrant activation of certain oncogenes can all lead to cellular senescence [1], [2]. The latter case is termed oncogene induced senescence (OIS) and is thought to function as a mechanism to prevent tumorigenesis [3], [4]. The presence of oncogenic BRAF^V600E^, for example, results in senescence *in vivo*, which is evident from senescent melanocytes that can be found in benign human naevi [5].

Although the precise molecular pathway leading from the activated oncogenes to the final growth arrest is not known, it involves activation of the RAF-MEK-ERK kinase pathway that results in transcriptional activation of the *INK4A-ARF-INK4B* locus. This important tumor suppressor locus codes for 3 proteins (p16^INK4A^, p15^INK4B^ and p14^ARF^) that all are activated by cellular stress and regulate cellular proliferation by feeding into the p53 and pRB tumor suppressor pathways [6]. Mouse cells depend on both p19^ARF^-p53 and p16^INK4A^-pRB pathways during OIS. However, in human cells it appears that p16^INK4A^ plays a more prominent role, as certain cell types only require p16^INK4A^ expression to induce senescence [7], [8]. The importance of this locus for senescence is also evident from the fact that among the factors that have been identified to regulate OIS are several proteins that regulate expression of the *INK4A-ARF-INK4B* locus, including the Polycomb group protein BMI1 [9], [10] and the histone demethylase JMJD3 [11], [12].

Recent results have shown the importance of senescence as a tumor barrier *in vivo*. For instance, failure to clear senescent cells after introduction of oncogenic *Nras* in the liver leads to increased incidence of hepatocellular carcinoma [13]. The protective effect involves immune surveillance and extensive communication of the pre-malignant senescent cells with their environment, probably through the acquisition of the senescence-associated secretory phenotype (SASP) [13], [14].

Several microRNAs (miRNAs) have also been shown to be involved in senescence. miRNAs are transcribed, processed into hairpin intermediates called pre-microRNAs (pre-miRs), and cleaved to give mature 21–23 nucleotide long miRNAs that often function by targeting the 3′ untranslated region (UTR) of mRNA transcripts, thereby downregulating the expression of their targets [15]. miRNAs that can either induce or help cells to evade senescence have been identified and they include miRNAs that target the p53 pathway, the p16^INK4A^-pRB pathway and the SASP [16], [17].

To identify new factors involved in OIS, we performed a screen for miRNAs that can overcome or inhibit OIS in human diploid fibroblasts, using p16^INK4A^ expression as a readout. We expressed a library of 471 human pre-miRs in human fibroblasts, which were subsequently induced to senesce by activation of the Braf oncogene [18]. Our screen identified several miRNAs that can regulate senescence, among which miR-378a-5p (previously miR-378 and miR-378*), a miRNA that is expressed in several types of cancer and has oncogenic properties [19]–[23]. The introduction of miR-378a-5p oligonucleotides resulted in reduced activation of p16^INK4A^ upon activation of Braf and allowed cells to retain proliferative capacity even in the presence of the activated oncogene. We furthermore identified putative miR-378a-5p target mRNAs in human fibroblasts by high throughput RNA sequencing. Taken together our results suggest that miR-378a-5p can have a positive effect on tumor formation by preventing full activation of the senescence program. These results are in agreement with the oncogenic features of miR-378a-5p

## Results

### Identification of miRNAs regulating OIS

**Figure 1:**
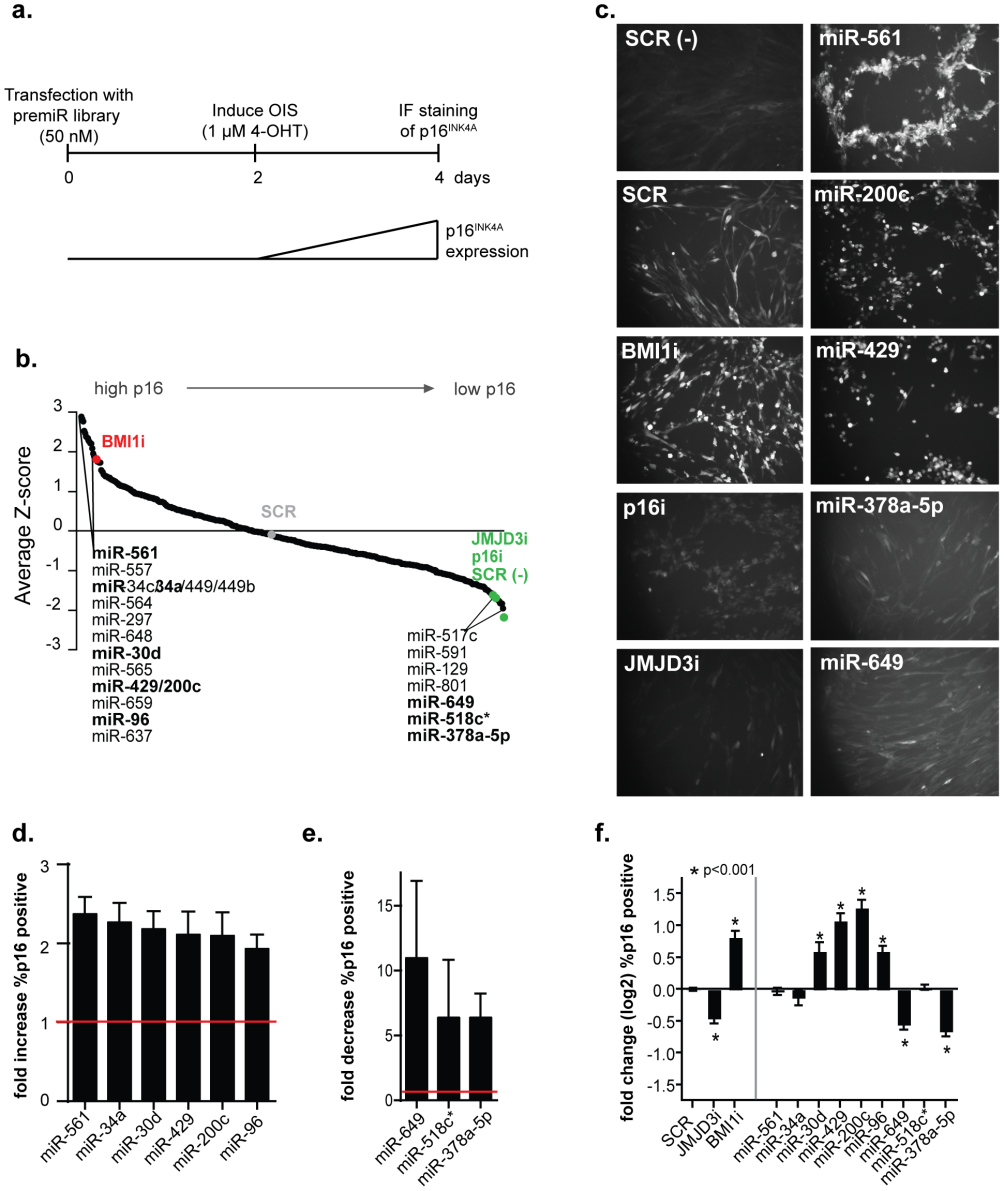
Identification of miRNAs that affect p16^INK4A^ expression during oncogene-induced senescence. (a) Graphical representation of the workflow. The screen was performed in 384 well-format in TIG3 ΔBraf:ER cells with a library containing 471 human pre-miRs. 48 hours after transfection the cells were treated with 4-hydroxy-tamoxifen (4-OHT) for 48 hours and p16^INK4A^ levels were determined by immunofluorescense (IF) and analyzed automatically. (b) Screen results. The percentage of p16^INK4A^ positive cells was determined for each well using the Hoechst signal to determine the total cell number. Following automated image analysis, the Z-score was calculated for each miRNA based on the average percentage of p16^INK4A^ positive cells per plate. All listed miRNAs are considered potential hits; the ones indicated in bold were chosen for further validation. BMI1i, p16i and JMJD3i represent controls in which BMI1, p16^INK4A^ or JMJD3 were downregulated by siRNAs. SCR: scrambled control. (-) indicates scrambled control cells in which senescence was not induced. (c) Immunofluorescense images taken as part of the screening process. Controls and selected hits are shown. (d) Fold change relative to the plate average of all hits increasing the percentage of p16^INK4A^ positive cells in the screen (red line indicates the overall average). (e) Fold change relative to the plate average of all hits decreasing the percentage of p16^INK4A^ positive cells in the screen (red line indicates the overall average). (f) The average of p16^INK4A^ induction in 5 independent experiments. IF for p16^INK4A^ followed by automated image analysis was used as a readout and scrambled (SCR) control samples were used for normalization. Averages are shown with SEM and t-tests were used to evaluate differences. Asterisks indicate a significant difference (p<0.001) in p16^INK4A^ induction.

To identify miRNAs with a role in OIS, we used the human diploid fibroblast line TIG3, which was immortalized with telomerase and expressed a conditional form of the mouse Braf oncogene (ΔBraf:ER), that allows senescence to be induced by treatment with 4-hydroxytamoxifen (4-OHT) [11], [18]. Immunofluorescent staining of p16^INK4A^ was used as a measure for senescence and the screen was essentially performed as follows: cells were transfected on day 0, treated with 1 µM 4-OHT on day 2 and fixed for analysis on day 4 (Figure 1a). A library containing 471 human pre-miRs (miRNA mimics) was reverse transfected into TIG3-hTERT-ΔBraf:ER cells and their effect on senescence determined (Figure 1b). In addition to a scrambled control (SCR), we used siRNAs against *BMI1*, *INK4A* and *JMJD3* (BMI1i, p16i, JMJD3i) as controls in every plate in the screen. Cells transfected with the SCR control were treated with ethanol as a technical control for the staining and image analysis procedure (SCR (-) Figure 1). The behavior of the controls in each plate was determined (Figure S1a). The control siRNAs included in the screen (i.e. knockdown of BMI1 or JMJD3) have been shown to play a biological role in senescence [9]–[12] and therefore all miRNAs that performed better than the control siRNAs were considered potential hits (listed miRNAs in Figure 1b). In the screen we identified 16 miRNAs that had a positive effect on p16 expression and 7 miRNAs whose expression resulted in reduced p16 levels. Images of controls and selected miRNAs are shown in Figure 1c, pictures for all miRNAs considered hits are depicted in Figures S2, S3). The fold change in percentage p16 positive cells relative to each plate average was approximately 2.25 for hits increasing (Figure 1d) and 5–10 fold for hits decreasing (Figure 1e) the percentage of p16^INK4A^ positive cells.

To validate the results from the screen, we designed miRNA oligonucleotides that were used in all subsequent experiments. To ensure incorporation of the desired strand into RISC, we designed the miRNA oligonucleotides with a wobble at the 5′ end of the mature strand [24]. miRNAs indicated in bold font (Figure 1b) were selected for validation. They were transfected in 96-well plate format and analyzed by automated image analysis (Figure 1f). Out of the selected potential hits miRNAs-30d, 429, 200c, 96, 649 and 378a-5p were successfully validated, and showed a significant effect on p16^INK4A^ levels after senescence induction compared to the scrambled control (Figure 1f, asterisks). The miRNAs increasing p16^INK4A^ expression include 2 members of the miR-200/429 family, of which miR-200c has been shown to be a transcriptional target of p53 and to be involved in senescence induction [25], [26]. Interestingly, the miR-200/429 family can also regulate several members of the polycomb family [27], [28].

2 miRNAs miR-649 and miR-378a-5p that seemed to help cells overcome senescence were identified. In this paper we have focused on miR-378a-5p, because it was shown to be over expressed in several cancer types and implicated in the development and maintenance of cancer cells *in vitro* [19]–[23].

### Increased expression of miR-378a-5p can partly overcome OIS

**Figure 2:**
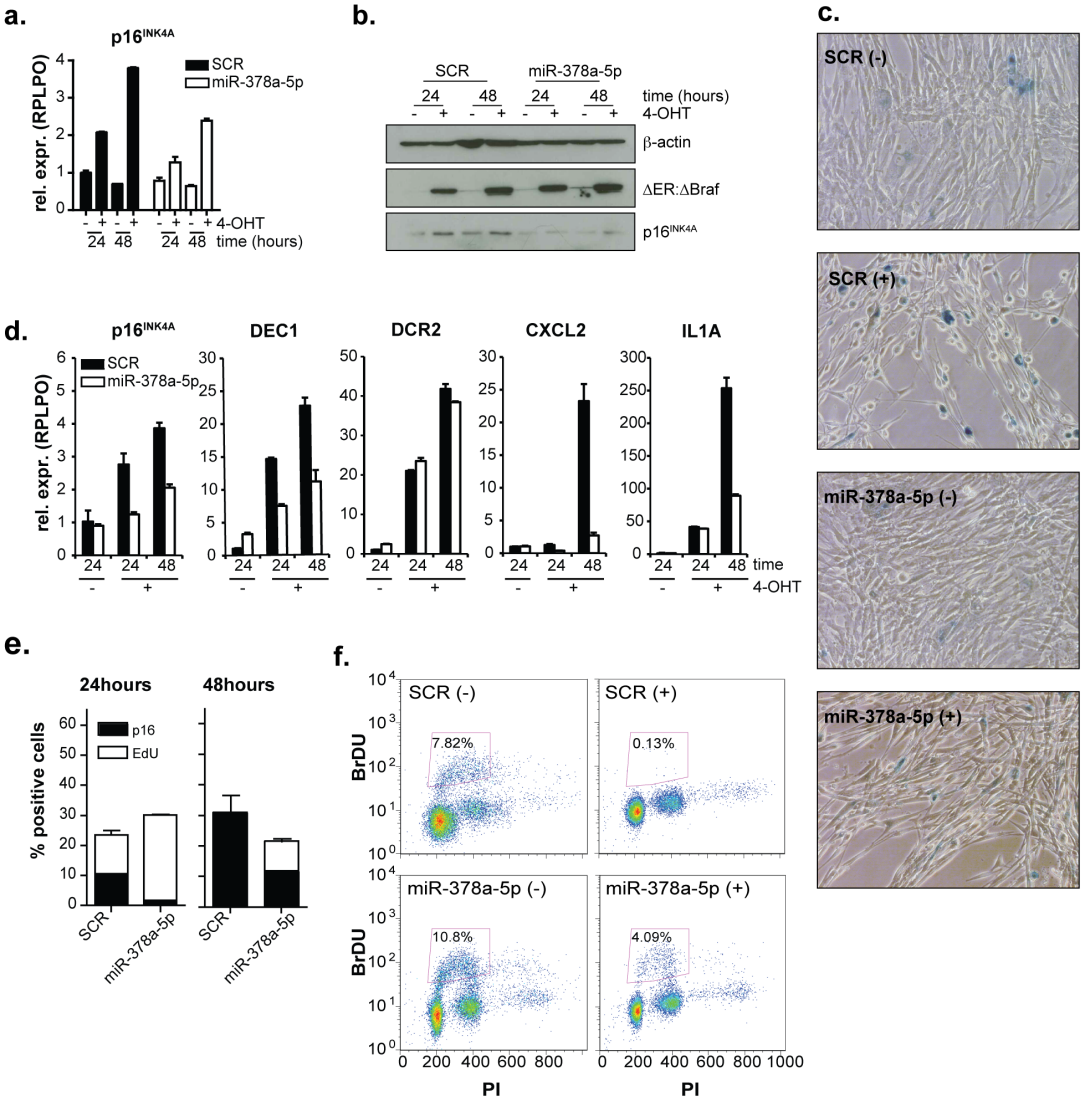
miR-378a-5p overexpression impairs oncogene-induced senescence. TIG3 ΔBraf:ER cells were transfected with scrambled control (SCR) or miR-378a-5p or miR378a-3p and after 48 hours treated with 4-OHT (+) or ethanol (−). (a) RT-qPCR using the housekeeping gene *RPLPO* as a reference and (b) western blot analysis of p16^INK4A^ expression after 24 and 48 hours of senescence induction (c) Senescence-associated β-gal staining after 48 hours of ethanol or 4-OHT treatment. (d) RT-qPCR analysis of the indicated senescence markers after 24 and 48 hours. Expression of *RPLPO* was determined and used for normalization. (e) Quantification of EdU and p16^INK4A^ staining of cells treated with ethanol or 4-OHT for 24 and 48 hours. Averages of 3 replicates are shown with the standard deviation. (f) Flow cytometric analysis of BrdU and propidium iodide staining of cells treated with ethanol or 4-OHT for 48 hours.

Our immunofluorescence staining demonstrates that miR-378a-5p is capable of reducing p16^INK4A^ protein levels. Moreover, as shown in Figures 2a,b, mir-378a-5b can also repress p16^INK4A^ mRNA and protein levels. Importantly, miR-378a-5p expression does not alter the levels of Braf:ER. To determine whether cells are still undergoing senescence in the presence of ectopically expressed miR-378a-5p, we determined the levels of senescence associated β-galactosidase (SA- βGAL; Figure 2c) and of several other markers of senescence [7] (Figure 2d). Morphologically, cells transfected with miR-378a-5p oligonucleotides were more similar to the untreated (- 4-OHT) control cells than the spindle-shaped morphology normally observed in fibroblast cells expressing oncogenic Braf (Figure 2c and [5]). Moreover, the number of SA- βGAL positive cells was reduced in cells transfected with miR-378a-5p oligonucleotides as compared to control (Figure 2c). Consistent with miR-378a-5p having a role in regulating senescence, the induction of the senescence markers DEC1, CXCL2 and IL1A was impaired in cells overexpressing miR-378a-5p (Figure 2d). Though another tested marker, DCR2, was not altered, these data show that the expression of miR-378a-5p is sufficient to prevent cells from fully entering the senescence program.

Since the definition of senescence is an irreversible arrest of proliferation, we also determined the proliferation rate by BrdU and EdU incorporation in these cells. We found that miR-378a-5p transfected cells showed increased EdU incorporation, while at the same time p16^INK4A^ levels were reduced upon Braf activation (Figure 2e) after 24 and 48 hours. This was confirmed by BrdU incorporation assay after 48 hours, where only 0.13% of the SCR control cells had incorporated BrdU vs 4.09% of the miR-378a-5p expressing cells (Figure 2f). Taken together these results show that miR-378a-5p expression can lead to a delay in execution of oncogene-induced senescence.

### Identification of potential miR-378-5p target mRNAs

**Figure 3:**
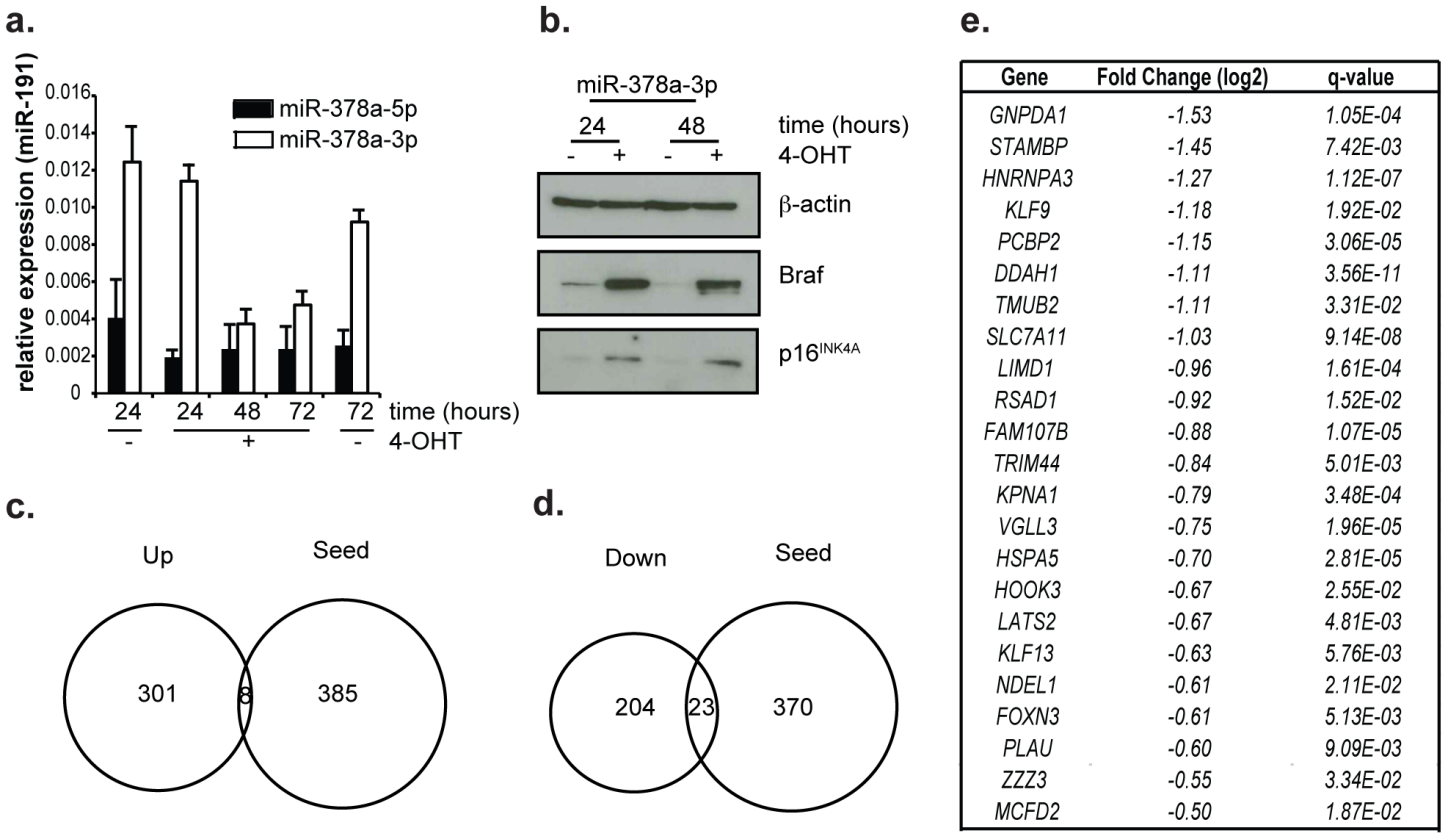
Identification of miR-378a-5p targets in TIG3 ΔBraf:ER cells. (a) Relative expression of miR-378a-5p and miR-378a-3p in TIG3 ΔBraf:ER cells during induction of senescence. miR-191 expression was used as a reference. (b) p16^INK4A^ protein expression of TIG3 ΔBraf:ER cells transfected with miR-378a-3p after 48 hours treatment with 4-OHT (+) or ethanol (−). (c) Venn diagram of genes significantly up regulated following over expression of miR-378a-5p and all genes predicted to have the seed sequence in their 5′UTR. (d) Venn diagram of genes significantly down regulated following over expression of miR-378a-5p and all genes predicted to have the seed sequence in their 5′UTR. (e) List of 19 genes significantly down regulated and containing the miR-378a-5p seed sequence. Their fold-change and false discovery rate (q-value) are indicated. RNA sequencing was used to measure gene expression.

miRNA genes are transcribed and in the process of generating the mature miRNA, two distinct mature miRNAs can be formed and become active. We measured the expression of both mature miRNAs transcribed from the *miR-378a* gene, miR-378a-5p and miR-378a-3p, by RT-qPCR analysis and found that both are expressed at low levels in TIG3 cells (Figure 3a). We expected to find miR-378a-5p at low levels, as it counteracts senescence, which these cells can normally undergo upon receiving the appropriate signals. In order to determine whether the effect we observe for miR-378a-5p is specific to this miRNA, we expressed miR-378a-3p oligonucleotides in TIG3-hTERT-ΔBraf:ER cells, which did not result in decreased p16^INK4A^ protein levels upon senescence induction (Figure 3b) as compared to SCR control transfected cells (Figure 2b).

Two targets of miR-378-5p as predicted by Targetscan 5.2 [29], SP1 and SUFU, previously have been implicated in regulation of p16^INK4A^ [30], [31]. Therefore, we tested whether their expression was affected by miR-378a-5p in TIG3-hTERT-ΔBraf:ER cells, and if their knockdown was sufficient to copy the phenotype observed upon miR-378a-5p over expression. However, we did not find the two genes differentially expressed and knockdown of SUFU or SP1 alone using 2 different siRNAs per gene or in combination did not result in altered expression of p16^INK4A^ in response to Braf expression in TIG3 cells (Figure S4a, b). Therefore, the two genes did not explain the effect of miR-378a-5p, and we used RNA sequencing to identify genes whose expression was altered after expressing miR-378a-5p.

Cells were transfected with miR-378a-5p oligonucleotides or controls, and mRNA was extracted 48 hours after transfection. mRNA of biological replicate samples were sequenced and analyzed using TopHat and Cuffdiff [32]. We identified 309 genes that were upregulated (Figure 3c, table S1) and 227 genes that were downregulated in response to miR-378a-5p expression (Figure 3d, table S1). These differences in expression comprise both primary and secondary effects. As miRNAs target their mRNAs using a 6–8 nucleotide long seed sequence present in their 5 prime end, we overlapped the list of differentially expressed genes with the predicted targets for miR-378a-5p (Figure 3e). Downregulated genes containing a match for the seed-sequence of miR-378a-5p with their relative fold change and false discovery rates (q-values) are indicated in Figure 3e. We then validated the expression changes in 3 independent biological replicates (Figure S5a) and could confirm the downregulation by miR-378a-5p of 20 genes (*GNPDA1*, *STAMBP*, *HNRNPA3*, *KLF9*, *PCBP2*, *TMUB2*, *SLC7A11*, *LIMD1*, *RSAD1*, *TRIM44*, *KPNA1*, *VGLL3*, *HOOK3*, *LATS2*, *KLF13*, *NDEL1*, *FOXN3*, *PLAU*, *ZZZ3* and *MCFD2*). Though several of these have been described as potential tumor suppressor genes, or to be downregulated in different tumor types [33]–[42], the role of these proteins in senescence induction has not been determined.

In addition to the targets that we identified by RNA sequencing, miR-378a-5p has been shown to be involved in tumorigenesis and tumor maintenance by regulating SUFU, TUSC2, TOB2, GABPA and ESRRg [19]–[23]. In our RNA sequencing experiment, the expression of these genes was not significantly altered and we therefore analyzed their expression by RT-qPCR (Figure S5b). Here again we found that they were not differentially expressed upon miR-378a-5p expression, and it is therefore unlikely that these genes contribute to regulating senescence in TIG3-hTERT-ΔBraf:ER.

## Discussion

The microRNA miR-378a-5p previously has been identified as an oncogene, where it has been shown to function in different pathways depending on the cell type studied. miR-378a-5p enhances cell survival, proliferation rate and angiogenesis in glioblastoma cells, through targeting of SUFU and TUSC2 [19]. In mammary cells, *miR-378a* is a transcriptional target of Myc, and it regulates oncogenic transformation through the regulation of TOB2 expression [20]. In addition, miR-378a-5p has been shown to be involved in increasing cell proliferation of breast cancer cells by mediating a metabolic shift. Through downregulation of GABPA and ERRγ, two PGC-1β partners, miR–378a–5p helps to orchestrate the Warburg effect in breast cancer cells [21].

Here, we have shown that over expression of miR-378a-5p allows human fibroblasts to escape the full senescence program upon induction of oncogenic Braf. As the expression of none of the previously published targets of miR-378a-5p is affected in TIG3 cells, they are most likely not involved here. Therefore we performed experiments to identify 20 potential mRNA targets of miR-378a-5p in human fibroblasts. Even though further experiments will be required to determine the contribution of the 20 identified target genes in oncogene-induced senescence, we hypothesize that one, or perhaps more likely, more than one of these 20 putative miR-378a-5p target genes contribute to the observed phenotype. In summary, we have shown that the oncogenic microRNA miR-378a-5p can contribute to overriding oncogene-induced senescence *in vitro*. Since senescence forms a barrier against tumor formation *in vivo*, we speculate.that the observed effect on senescence induction could provide miR-378a-5p with an additional mechanism for how it is involved in tumorigenesis.

## Materials and Methods

### Cell culture and siRNA transfections

The human diploid cell line TIG3 (from the Japanese Cancer Research Resources Bank, Tokyo, Japan) was immortalized with telomerase (hTERT) and transduced with a retrovirus generated from pMSCV_blast_-ΔBraf:ER in order to be able to induce senescence [11], [18]. Cells were maintained in DMEM (Gibco) supplemented with 10% FBS (Hyclone) and penicillin/streptomycin (Gibco). Senescence was induced by treatment with 1 µM 4-hydroxytamoxifen (4-OHT, Sigma) from a 1 mM stock in 96% ethanol. As a control, cells were treated with ethanol alone.

siRNA and miRNA oligonucleotides were introduced into TIG3 cells at a final concentration of 50 nM by reverse transfection using Lipofectamine 2000 (Invitrogen) according to the manufacturer's instructions. The siRNAs and miRNA oligonucleotides that were used are listed in table S2.

### siRNA screen and p16^INK4A^ immunofluoresence

The screen was performed using a human-pre-miR-library containing 471 human pre-miRs (Ambion). TIG3-hTERT-ΔBraf:ER cells were reverse transfected in quadruplicates in 384-well plates and after 48 hours treated with 1 µM of 4-OHT for an additional 48 hours. Each plate contained cells transfected with control siRNAs (scrambled, JMJD3i, BMI1i). Cells were fixed by adding an equal volume of 4% formaldehyde to the wells, after which they were permeabilized with Triton-x-100 and stained with p16^INK4A^ antibody (sc-56330, Santa Cruz) and Hoechst 33258 (Invitrogen). Automated image analysis was performed on the INCell Analyzer 1000 (GE Healthcare) using a 20× objective and 6 images per well (containing approximately 100 cells per image) and the percentage of p16^INK4A^ positive cells per image determined using the INCell Analyzer Workstation 3.6 software (GE Healthcare). The Hoechst signal was used to determine the total cell number in each image and the p16^INK4A^ values of the 6 images were averaged for further analysis. For hit determination, Z-scores were calculated for each well using the respective plate averages (Figure S1b).

### Antibodies for Western blotting

The following antibodies were used in Western blot analysis: p16^INK4A^ (DCS50), ΔBraf:ER (sc-166 Santa Cruz) and β-actin (Ab6276 Abcam)

### Senescence associated β-galactosidase staining

Senescence associated β-galactosidase staining was performed as previously described [43].

### BrdU and EdU incorporation assays

Cells were pulsed with 33 µM BrdU or 8 µM EdU for 3 hours prior to fixation. BrdU treated cells were fixed and stained with an antibody against BrdU (Beckson & Dickinson) and propidium iodide (Sigma), after which they were analyzed by flow cytometry using a FACSCalibur (BD biosciences). EdU treated cells, which were grown in 96-well plates, were fixed and stained for p16^INK4A^ as described above. EdU was detected using Click-IT EdU chemistry (Invitrogen) according to manufacturer's protocol and automated image analysis as described above.

### RNA extraction and RT-qPCR

RNA for mRNA expression analysis was extracted using the RNeasy Plus kit (Qiagen) and reverse transcribed using Taqman reverse transcription reagents (Applied Biosystems). Quantitative PCR was done on a LightCycler480 (Roche), using LightCycler 480 SYBR Green I Master mix (Roche). Primer sequences are listed in table S3. RNA for miRNA expression analysis was extracted with the miRNeasy kit (Qiagen), reverse transcribed with Taqman MicroRNA reverse transcription kit (Applied biosystems) and quantified using Taqman MicroRNA assays for miR-191 (assay ID 002299), miR-378-3p (assay ID 002243) and miR-378-5p (assay ID 000567). Differences in expression were determined using the 2−ΔΔCt method [44], using the housekeeping gene *RPLP0* and *miR-191* for normalization.

### RNA sequencing

TIG3-hTERT-ΔBraf:ER cells were transfected with scrambled control or miR-378-5p oligonucleotides. 48 hours after transfection, RNA was extracted from 3 biological replicates, it's quality monitored on the 2100 expert Bioanalyzer (Agilent), and prepared for sequencing using the NEBNext mRNA sample Prep Master Mix Set 1(New England Biolabs). The amplified cDNA was analyzed by Solexa/Illumina high-throughput sequencing. The tags were mapped to the human genome (assembly hg19) with TopHat [45] and differential expression was determined with Cufflinks [32] at an FDR cut-off value <0.5.

## Supporting Information

Figure S1 **Identification of miRNAs that affect p16^INK4A^ expression during oncogene-induced senescence.**
(TIF)

Figure S2 **p16^INK4A^ induction during the screen.**
(TIF)

Figure S3 **p16^INK4A^ induction during the screen.**
(TIF)

Figure S4 **SUFU and SP1 are not involved in regulation of senescence by miR-378a-5p.**
(TIF)

Figure S5 **Validation of RNA sequencing.**
(TIF)

Table S1 **RNA sequencing data.**
(XLS)

Table S2 **Sequences of siRNA and miRNA oligonucleotides.**
(DOCX)

Table S3 **Primers used for RT-qPCR analysis.**
(DOCX)
