# Correction: A Screen Identifies the Oncogenic Micro-RNA miR-378a-5p as a Negative Regulator of Oncogene-Induced Senescence

**DOI:** 10.1371/journal.pone.0272206

**Published:** 2022-07-21

**Authors:** Susanne Marije Kooistra, Lise Christine Rudkjær Nørgaard, Michael James Lees, Cornelia Steinhauer, Jens Vilstrup Johansen, Kristian Helin

The S2 Fig miR-34c p16^INK4A^ panel of [1] was inadvertently duplicated and incorrectly used to represent the S2 Fig miR-449b p16^INK4A^ results. The corrected S2 Fig below has been updated so that the correct miR-449b p16^INK4A^ is presented, and the individual-level data underlying S2 Fig, are provided in S1 File below.

In addition, in S2 Fig and S3 Fig, the images were mislabeled, stating that a DAPI-signal is presented. As stated in the Materials and Methods section, the cells were stained with Hoechst; the updated figures, with corrected labelling, are provided with this notice below.

The first author explained that for each condition in the screen, 12 images (6 Hoechst and 6 P16^INK4A^) were collected. They clarified that, with the exception of the representative images presented in S2 Fig, the original image data used for quantification of the results presented in Fig 1 are no longer available; similarly, the 12 collected images per well underlying S1, S2, and S3, S4 Figs panel B, are no longer available. Additionally, the sequencing data were not deposited in an online repository, and therefore, the raw sequencing data used to generate Fig 3 panels C, D, and E, are no longer available.

This article [1] was submitted under the previous PLOS Data Availability policy [2] and thus did not have a Data Availability statement at the time. The updated Data Availability Statement for this article is: The individual-level data used for the preparation of the Fig 1 panels D, E, and F, are provided in S2 and S3 Files below. The quantified individual-level data used for the analysis and other data that were used to generate the figures are available upon request from the corresponding author.

The authors apologize for the error with S2 Fig.

## Supporting information

S2 Fig p16^INK4A^ induction during the screen. (TIF)

S3 Fig p16^INK4A^ induction during the screen. (TIF)

S1 File Underlying data used to generate S2 Fig. (ZIP)

S2 File Individual-level data used to generate Figs 1D and 1E. (XLSX)

S3 File Individual-level data used to generate Fig 1F. The validations of the miRNA’s affecting OIS were done in several experiments; the data is provided as Excel files for the individual experiments and as a combined file that was used to generate the graphs (validations combined_Fig_1f.xlsx).
(ZIP)
