# Supplementary figures and images for: A Screen Identifies the Oncogenic Micro-RNA miR-378a-5p as a Negative Regulator of Oncogene-Induced Senescence
Source: PLoS One. 2014 Mar 20;9(3):e91034. doi: 10.1371/journal.pone.0091034 (PMC3961217; doi:10.1371/journal.pone.0091034)

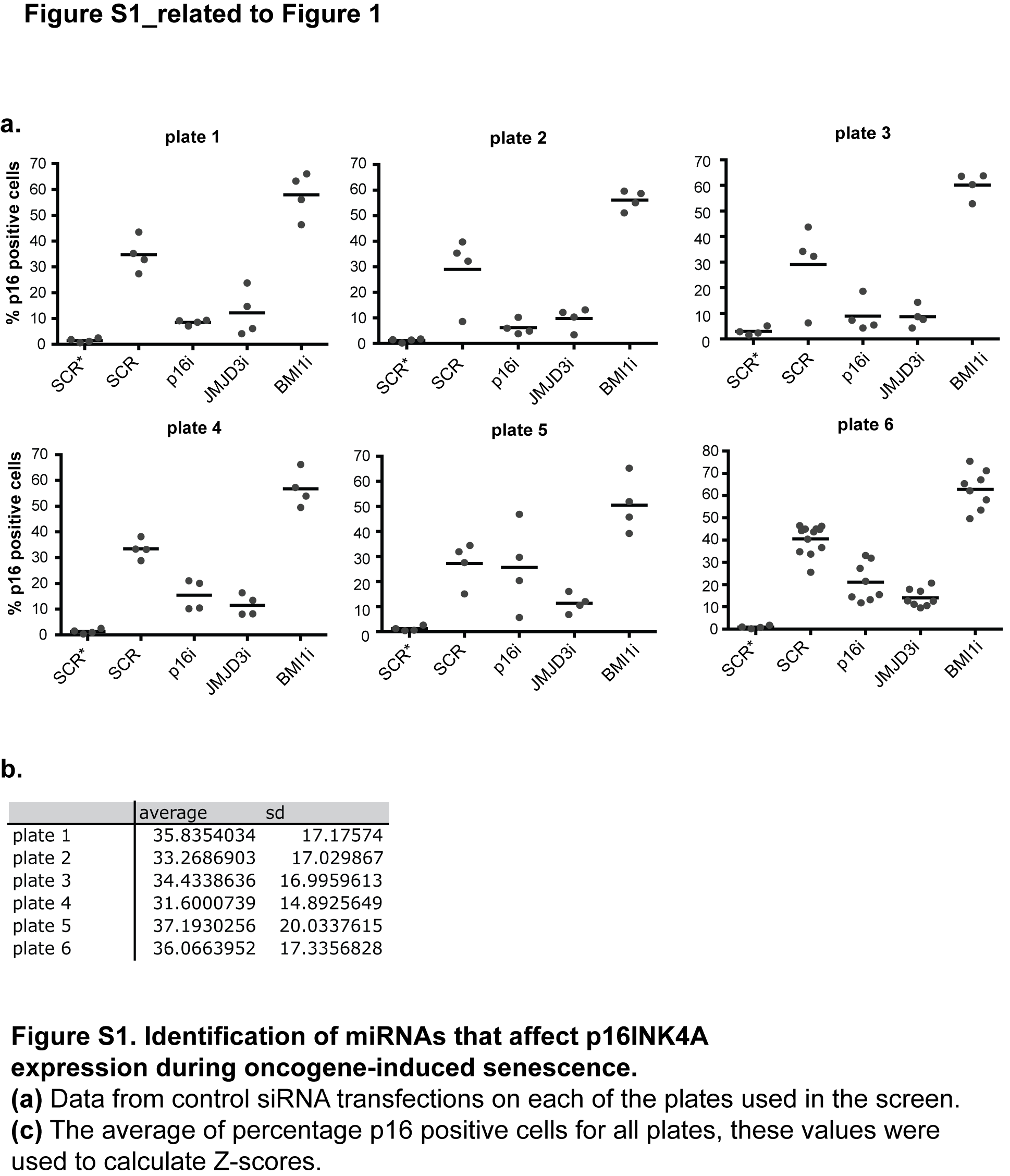

Supplement: Figure S1 — Identification of miRNAs that affect p16INK4A expression during oncogene-induced senescence. (TIF) [file pone.0091034.s001.tif]

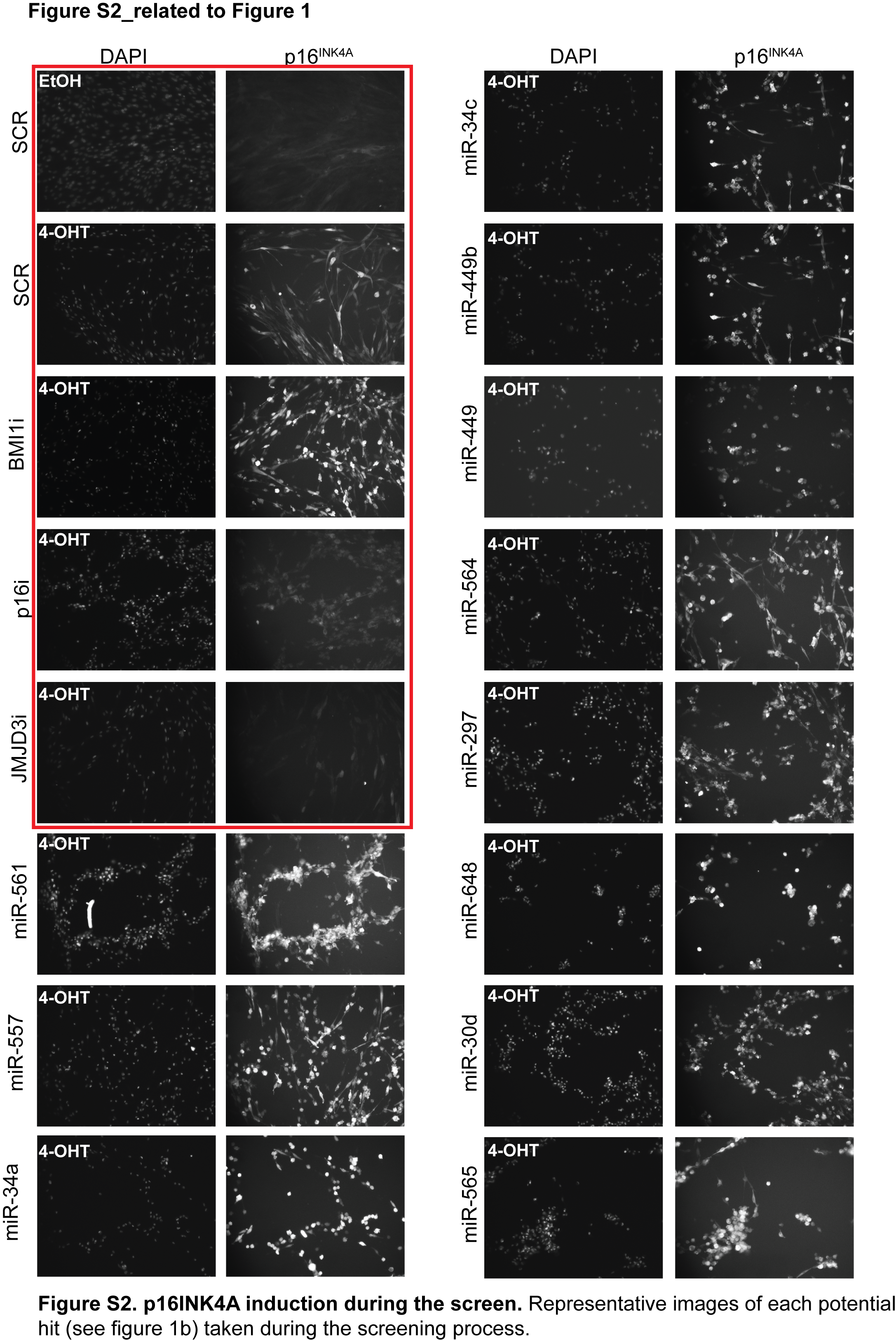

Supplement: Figure S2 — p16INK4A induction during the screen. (TIF) [file pone.0091034.s002.tif]

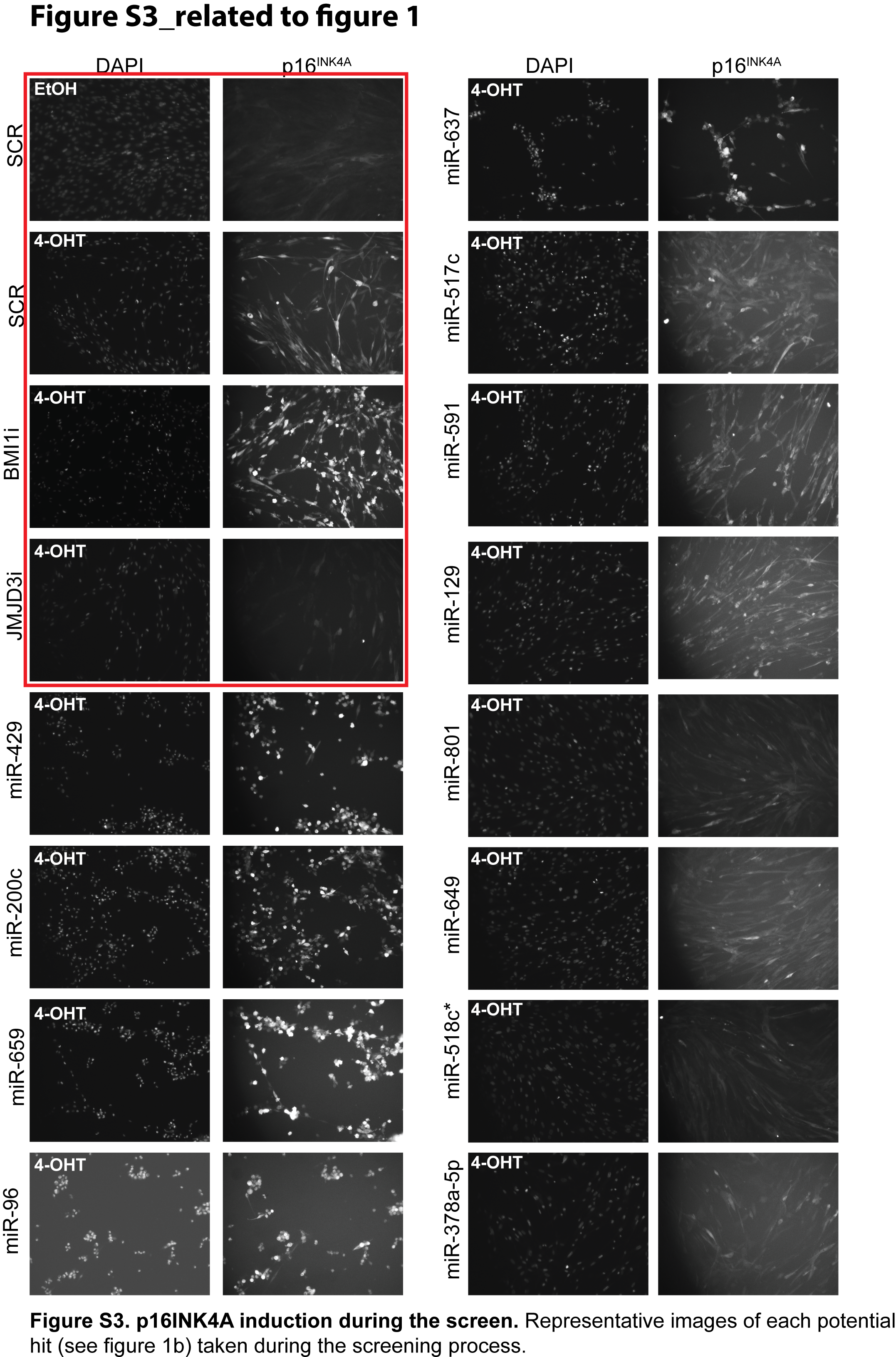

Supplement: Figure S3 — p16INK4A induction during the screen. (TIF) [file pone.0091034.s003.tif]

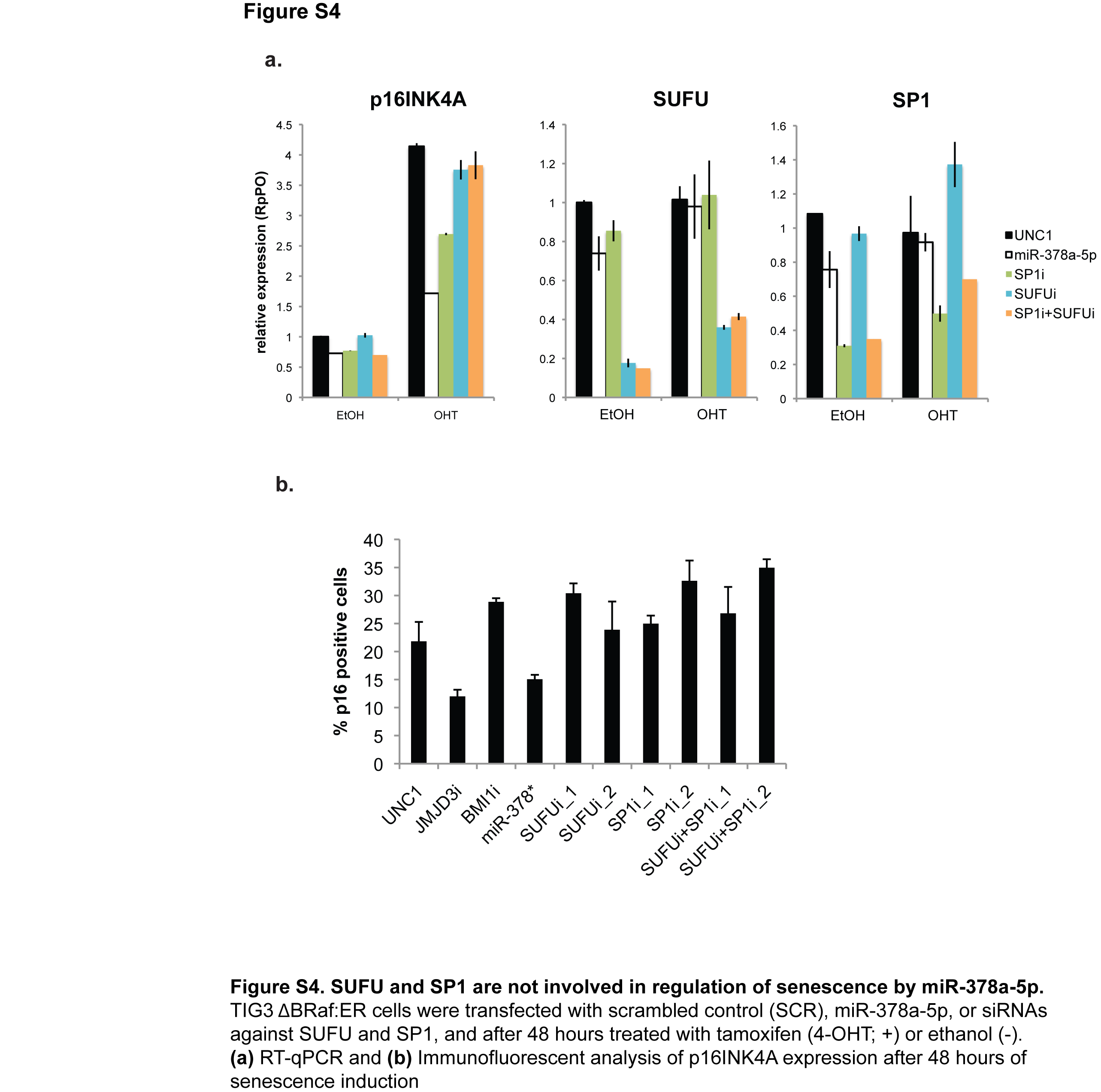

Supplement: Figure S4 — SUFU and SP1 are not involved in regulation of senescence by miR-378a-5p. (TIF) [file pone.0091034.s004.tif]

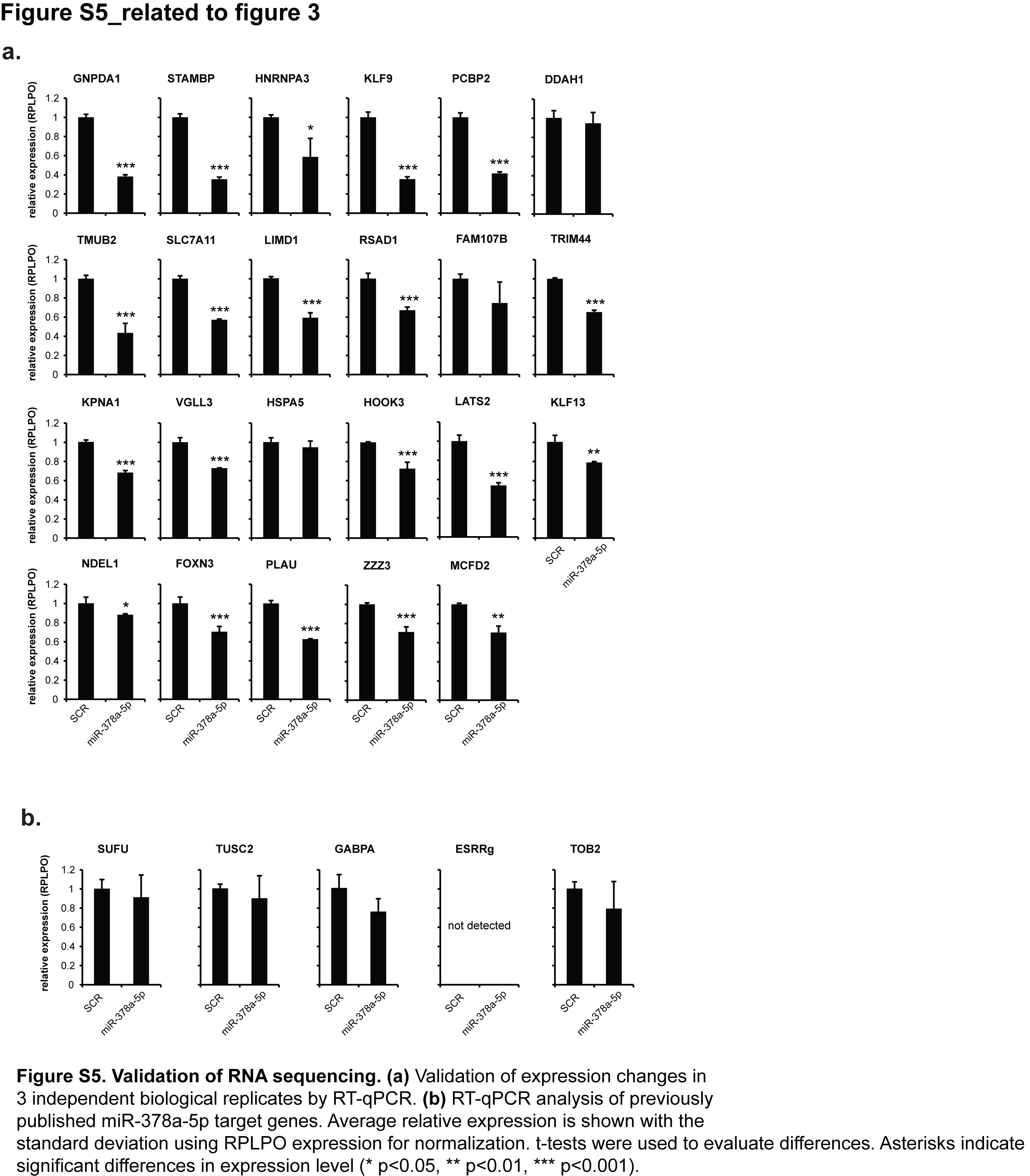

Supplement: Figure S5 — Validation of RNA sequencing. (TIF) [file pone.0091034.s005.tif]
