# Supplementary material for: A Screen Identifies the Oncogenic Micro-RNA miR-378a-5p as a Negative Regulator of Oncogene-Induced Senescence
Source: PLoS One. 2014 Mar 20;9(3):e91034. doi: 10.1371/journal.pone.0091034 (PMC3961217; doi:10.1371/journal.pone.0091034)
Supplement: Table S2 — Sequences of siRNA and miRNA oligonucleotides. (DOCX) [file pone.0091034.s007.docx]

**Table S2**. Sequences of siRNA and miRNA oligonucleotides

| **Name** | **Sense (sequence 5’-3’)** | **Antisense (sequence 5’-3’)** |
| --- | --- | --- |
| scrambled control  p16^INK4A^  Jmjd3  BMI1  SP1_1  SP1_2  SUFU_1  SUFU_2 | Universal Negative Control 1 (Sigma)  CUGCCCAACGCACCGAAUA[dT][dT]  GGCGACAGAAGGAGCAUCA[dT][dT]  GCGGUAACCACCAAUCUUC[dT][dT]  CAUACCAGGUGCAAACCAA[dT][dT]  CUACUACUACCACCAGCAA[dT][dT]  GACCAUAUUUGAGAUCGAU[dT][dT]  GACUUCCAGUGUAACAGUU[dT][dT] | -  UAUUCGGUGCGUUGGGCAG[dT][dT]  UGAUGCUCCUUCUGUCGCC[dT][dT]  GAAGAUUGGUGGUUACCGC[dT][dG]  UUGGUUUGCACCUGGUAUG[dT][dT]  UUGCUGGUGGUAGUAGUAG[dT][dT]  AUCGAUCUCAAAUAUGGUC[dT][dT]  AACUGUUACACUGGAAGUC[dT][dT] |
|  | | |
| **Name** | **Mature strand  (sequence 5’-3’)** | **Complementary strand (sequence 5’-3’)** |
| miR-378-5p  miR-378-3p  miR-561  miR-34a  miR-30d  miR-429  miR-200c  miR-96  miR-649  miR-518c* | CUCCUGACUCCAGGUCCUGUGU[dA][dA]  acuggacuuggagucagaagg[dA][dA]  CAAAGUUUAAGAUCCUUGAAGU[dA][dA]  UGGCAGUGUCUUAGCUGGUUGUU[dA][dA]  UGUAAACAUCCCCGACUGGAAG[dA][dA]  UAAUACUGUCUGGUAAAACCGU[dA][dA]  UAAUACUGCCGGGUAAUGAUGG[dA][dA]  UUUGGCACUAGCACAUUUUUGC[dA][dA]  AAACCUGUGUUGUUCAAGAGUC[dA][dA]  ucucuggagggaagcacuuucug[dA][dA] | ACACAGGACCUGGAGUCAGGAA[dA][dA]  CCUUCUGACUCCAAGUCCAGG[dA][dA]  ACUUCAAGGAUCUUAAACUUUA[dA][dA]  AACAACCAGCUAAGACACUGCCC[dA][dA]  CUUCCAGUCGGGGAUGUUUACC[dA][dA]  ACGGUUUUACCAGACAGUAUUC[dA][dA]  CCAUCAUUACCCGGCAGUAUUC[dA][dA]  GCAAAAAUGUGCUAGUGCCAAC[dA][dA]  GACUCUUGAACAACACAGGUUG[dA][dA]  CAGAAAGUGCUUCCCUCCAGAGC[dA][dA] |
