# Supplementary material for: A Screen Identifies the Oncogenic Micro-RNA miR-378a-5p as a Negative Regulator of Oncogene-Induced Senescence
Source: PLoS One. 2014 Mar 20;9(3):e91034. doi: 10.1371/journal.pone.0091034 (PMC3961217; doi:10.1371/journal.pone.0091034)
Supplement: Table S3 — Primers used for RT-qPCR analysis. (DOCX) [file pone.0091034.s008.docx]

**Table S3.** Primers used for RT-qPCR analysis

| **Name** | **forward primer (5’-3’)** | **reverse primer (5’-3’)** |
| --- | --- | --- |
| RPLP0  p16^INK4A^  DEC1  DCR2  CXCL2  IL1A  GABPA  SUFU  TUSC2  ESRRg  TOB2  GNPDA1  STAMBP  HNRNPA3  KLF9  PCBP2  DDAH1  TMUB2  SLC7A11  LIMD1  RSAD1  FAM107B  TRIM44  KPNA1  VGLL3  HSPA5  HOOK3  LATS2  KLF13  NDEL1  FOXN3  PLAU  ZZZ3  MCFD2 | TTCATTGTGGGAGCAGAC  GAAGGTCCCTCAGACATCCCC  AAAATCATTGCCCTGCAGAGT  TTGCTTCCAACAATTTGCCTT  GGGCAGAAAGCTTGTCTCAACCCC  ACCTCACGGCTGCTGCATTACA  ggacgggtctaggtgagaca  AGCATGTACAGGAATGTGGGGA  ggagacaatcgtcaccaagaa  CCTACGCTAACACTGTCGCA  caccctggagggagaagc  acccagggccagagaagta  atcagcagggcctcatca  gtggtggcagcagaggtagt  GGCTGTGGGAAAGTCTATGGAA  agatctgcgtggtcatgttg  cttccggactgcgtcttc  gcggctcaaattcctcaat  ccatgaacggtggtgtgtt  gttgaaattagaagccctcacc  gctccagactggcagagttc  GCAGTGGTGTTTCACGCTTC  ggactgaaggccgctatg  agcttgggccatcacaaa  tcccagtatctgcccaacc  agctgtagcgtatggtgctg  AACGAGACCGACTGTTCCAC  agcaagaaatggccaaagc  AGCACAAGTGCCACTACGC  ccattaaggagcagttgcataag  gccagtgccatgatgctt  ttgctcaccacaacgacatt  ctggcagaagatagcagatgaa  tggcaataatttgcttgatgg | CAGCAGTTTCTCCAGAGC  CCCTGTAGGACCTTCGGTGAC  GCACATGTCTGGAAACCTGAG  CACTGACACACGGTGTCTCTGG  GCGCAATCCAGGTGGCCTCT  TCCTTCAGCAGCACTGGTTGGT  tggctggagtatttcaaaggat  CTCATGGACTCTGTTGTCACCA  gggtgatccagcttcacg  GCTGGAAGGTTCCGTCTTGA  ggcagagaatcagcacagg  gcttcttgtagcagccaagtg  gagagaaacgcggtctgtg  caggaccaccgccatagtt  GCGGGAGAACTTTTTAAGGCAG  tgctgtacctgtcctgacca  tgcttctttcatcatgtcaacc  cttgtccagggaagtatttgct  gaccctctcgagacgcaac  cctttgctgcatttcacaca  cggagctcagtgtcatctagg  TCCAGCTCGAGGCATTTAGG  tcttcatttggtcccgagtt  cggcttgatacaacccagtt  tgctgaataccgctaacttcttc  aaggggacatacatcaagcagt  GCTGCCTTTTTATGCAGGGT  ggtagaggatcttccgcatct  GCGCGAACTTCTTGTTGCAG  gcctttgttcaaagtcttcca  gcgctccattttggatca  ggcaggcagatggtctgtat  tctgtactcggctggcaac  tcactcattagtggtgcctgtt |
