# Supplementary figures and images for: Correction: A Screen Identifies the Oncogenic Micro-RNA miR-378a-5p as a Negative Regulator of Oncogene-Induced Senescence (part 1 of 2)
Source: PLoS One. 2022 Jul 21;17(7):e0272206. doi: 10.1371/journal.pone.0272206 (PMC9302827; doi:10.1371/journal.pone.0272206)

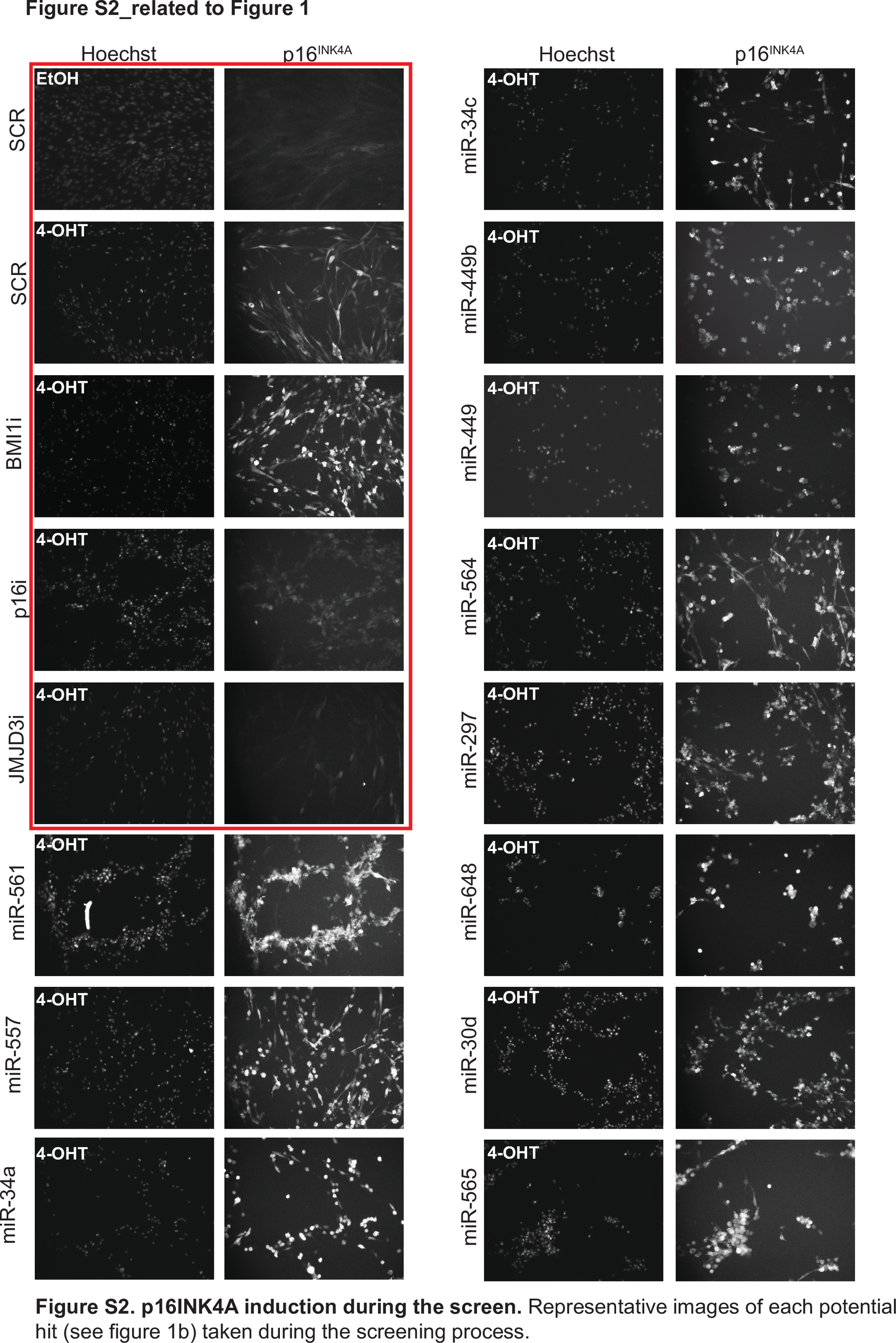

Supplement: S2 Fig — (TIF) [file pone.0272206.s001.tif]

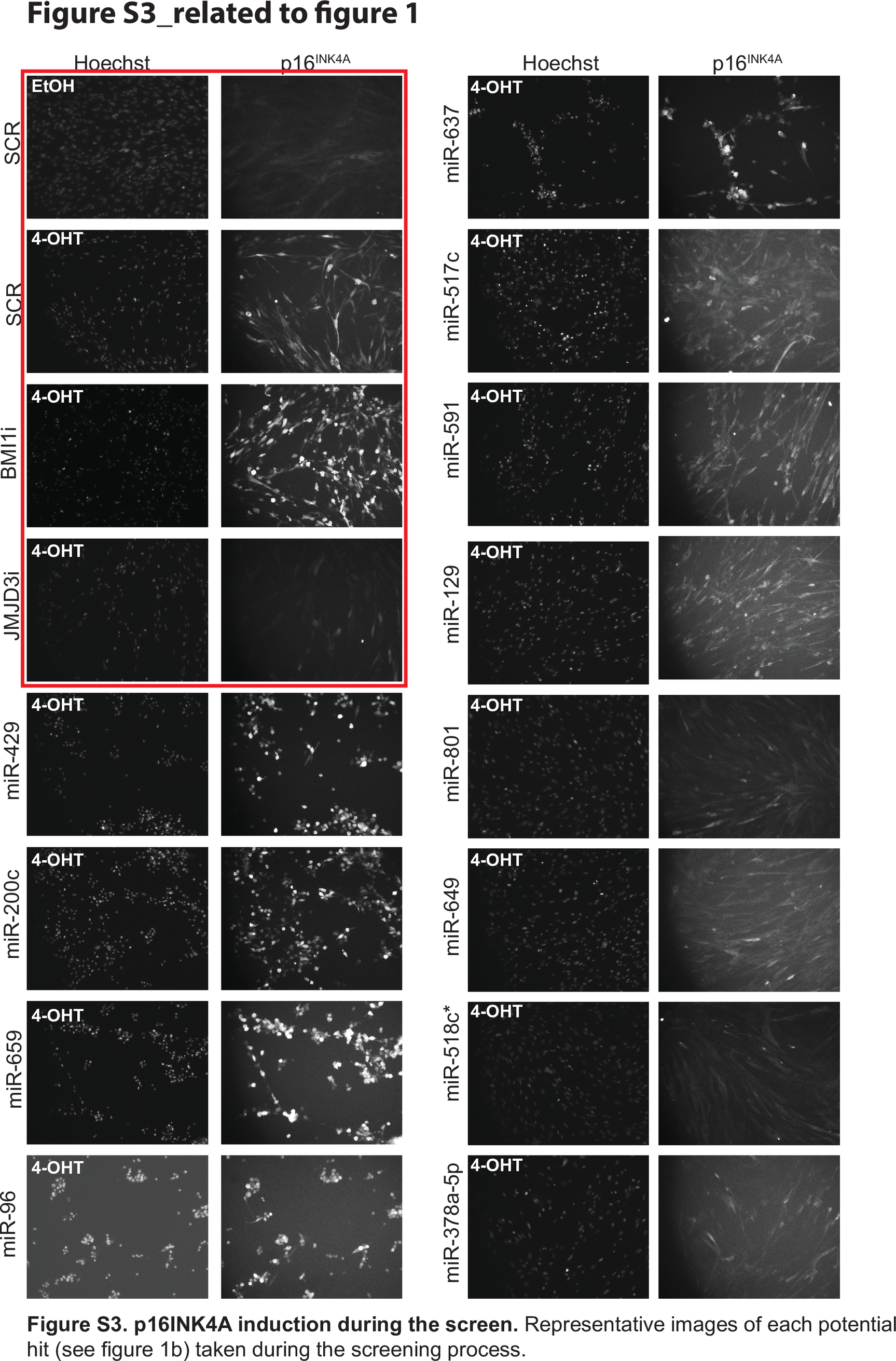

Supplement: S3 Fig — (TIF) [file pone.0272206.s002.tif]

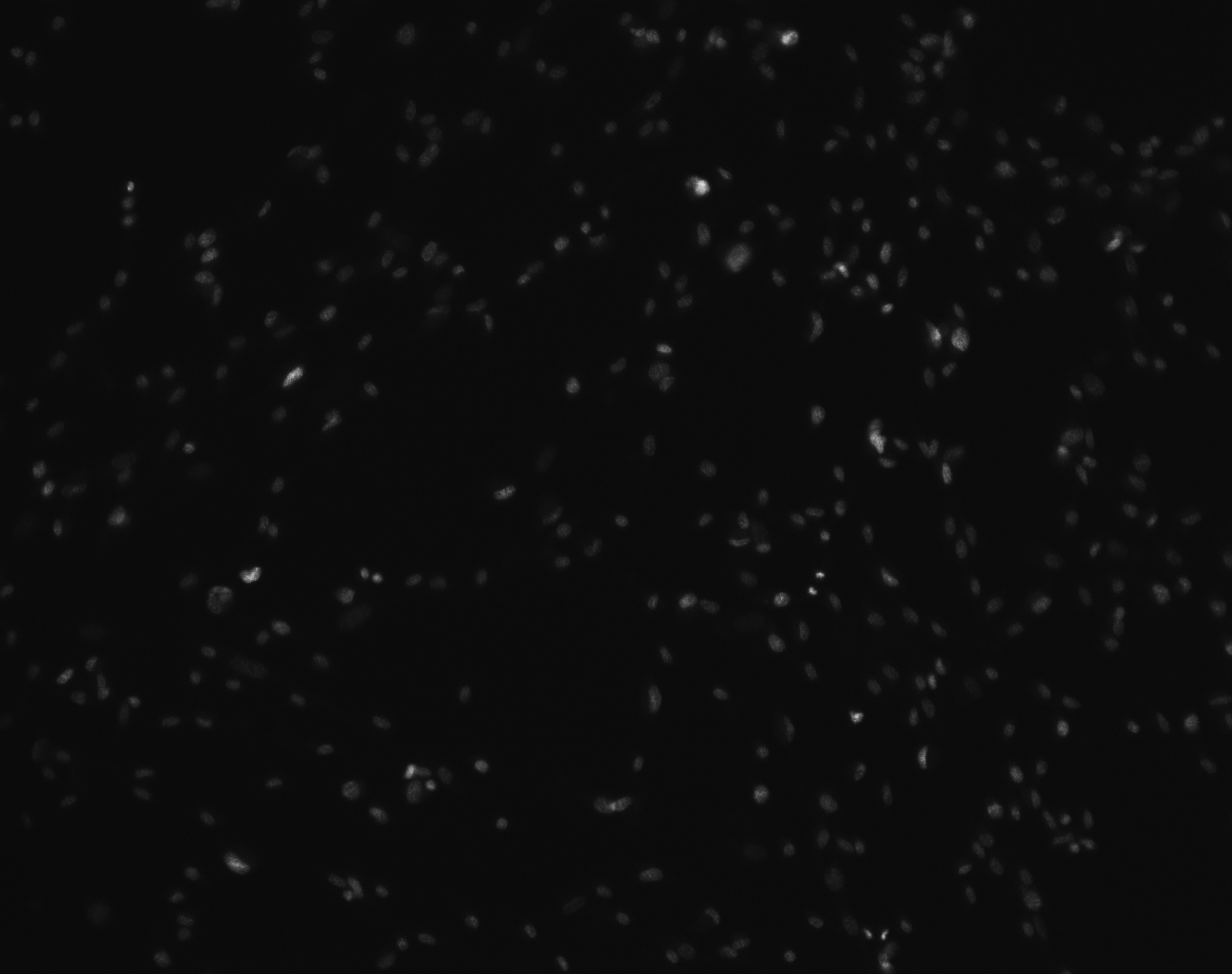

Supplement: S1 File — (ZIP) [file pone.0272206.s003.zip › new/BMI1/dapi.tif]

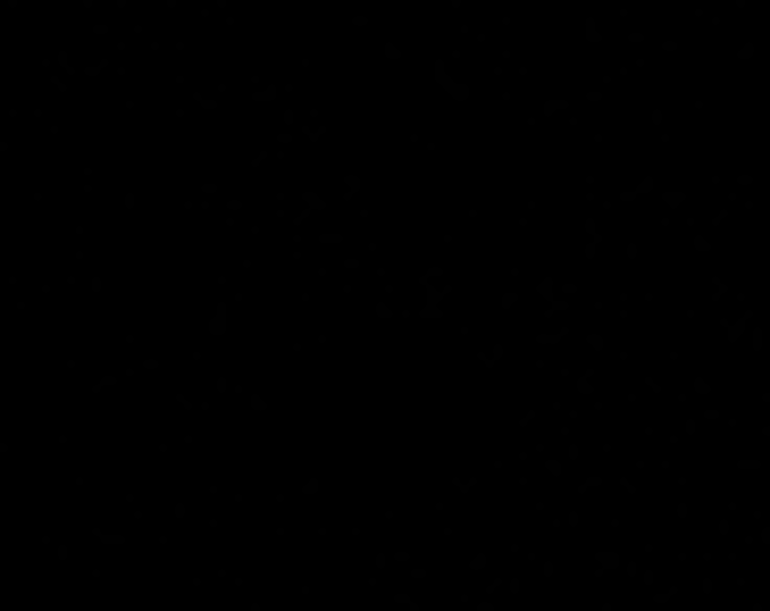

Supplement: S1 File — (ZIP) [file pone.0272206.s003.zip › new/BMI1/J - 11(fld 1 wv D360_40x - HQ460_40m)_thumb.tif]

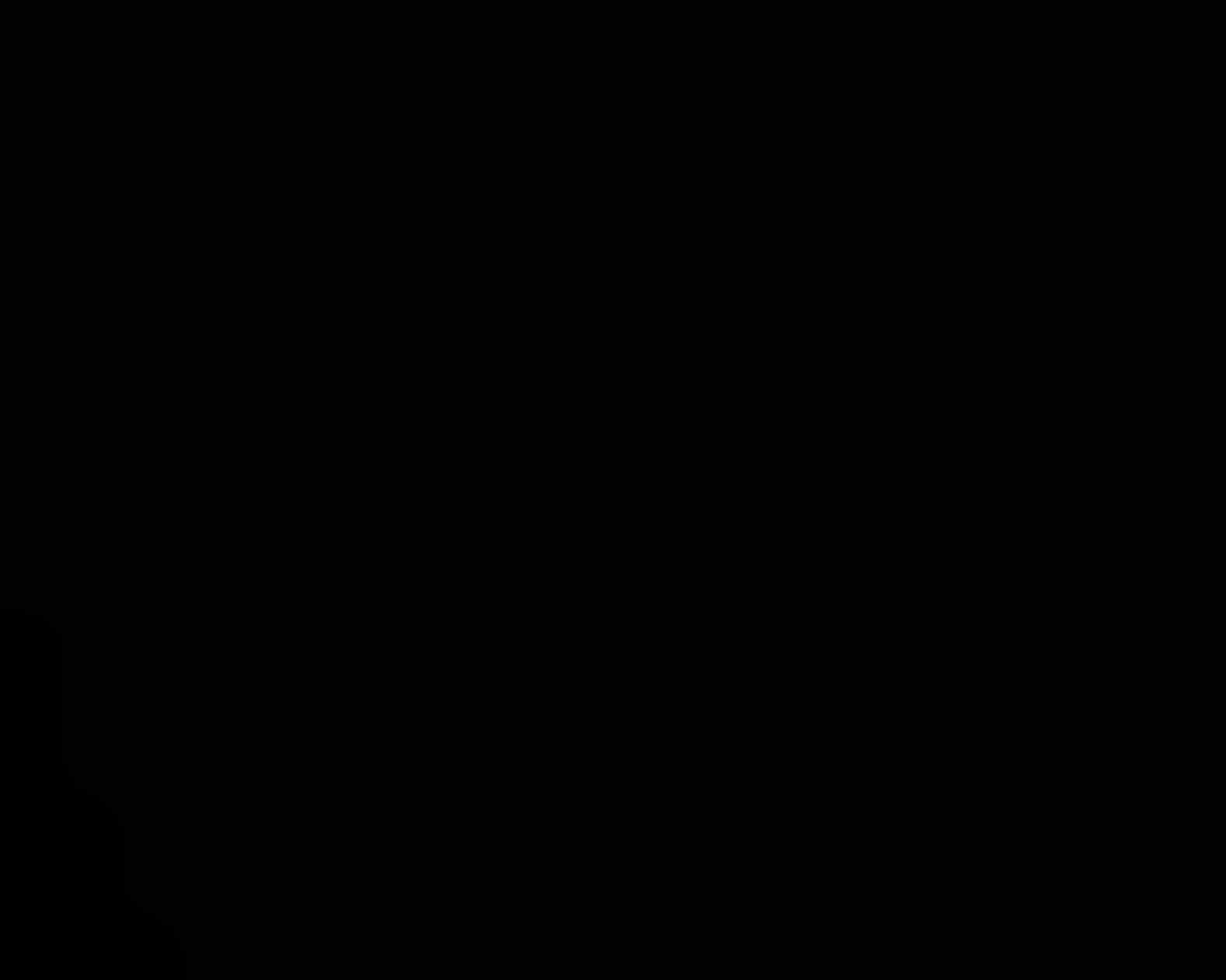

Supplement: S1 File — (ZIP) [file pone.0272206.s003.zip › new/BMI1/J - 11(fld 1 wv S475_20x - HQ535_50m).tif]

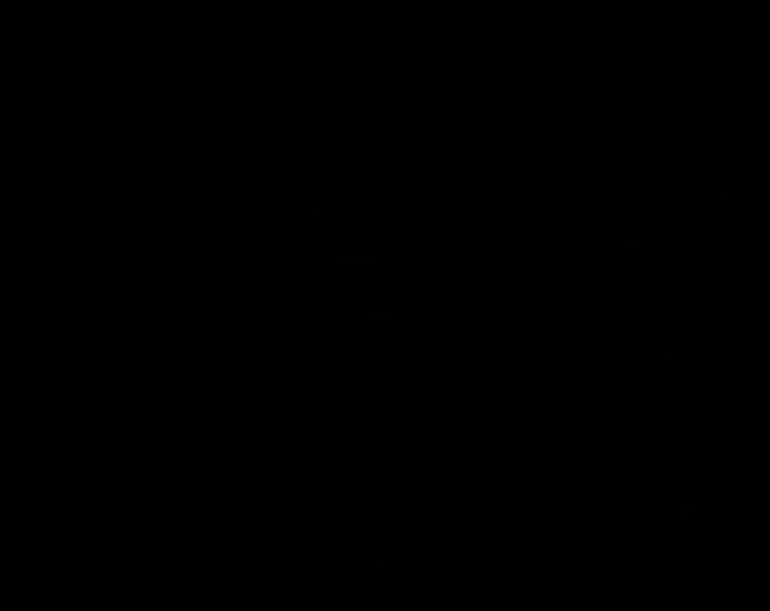

Supplement: S1 File — (ZIP) [file pone.0272206.s003.zip › new/BMI1/J - 11(fld 1 wv S475_20x - HQ535_50m)_thumb.tif]

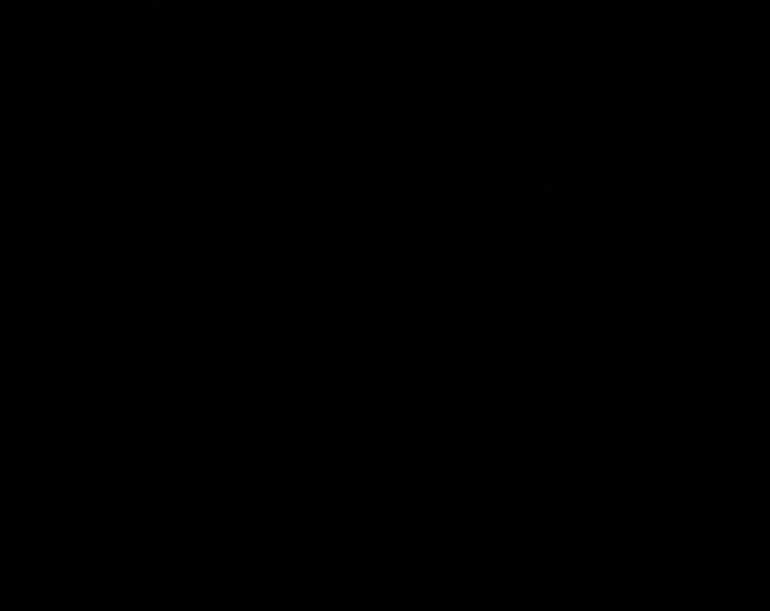

Supplement: S1 File — (ZIP) [file pone.0272206.s003.zip › new/BMI1/J - 11(fld 2 wv D360_40x - HQ460_40m)_thumb.tif]

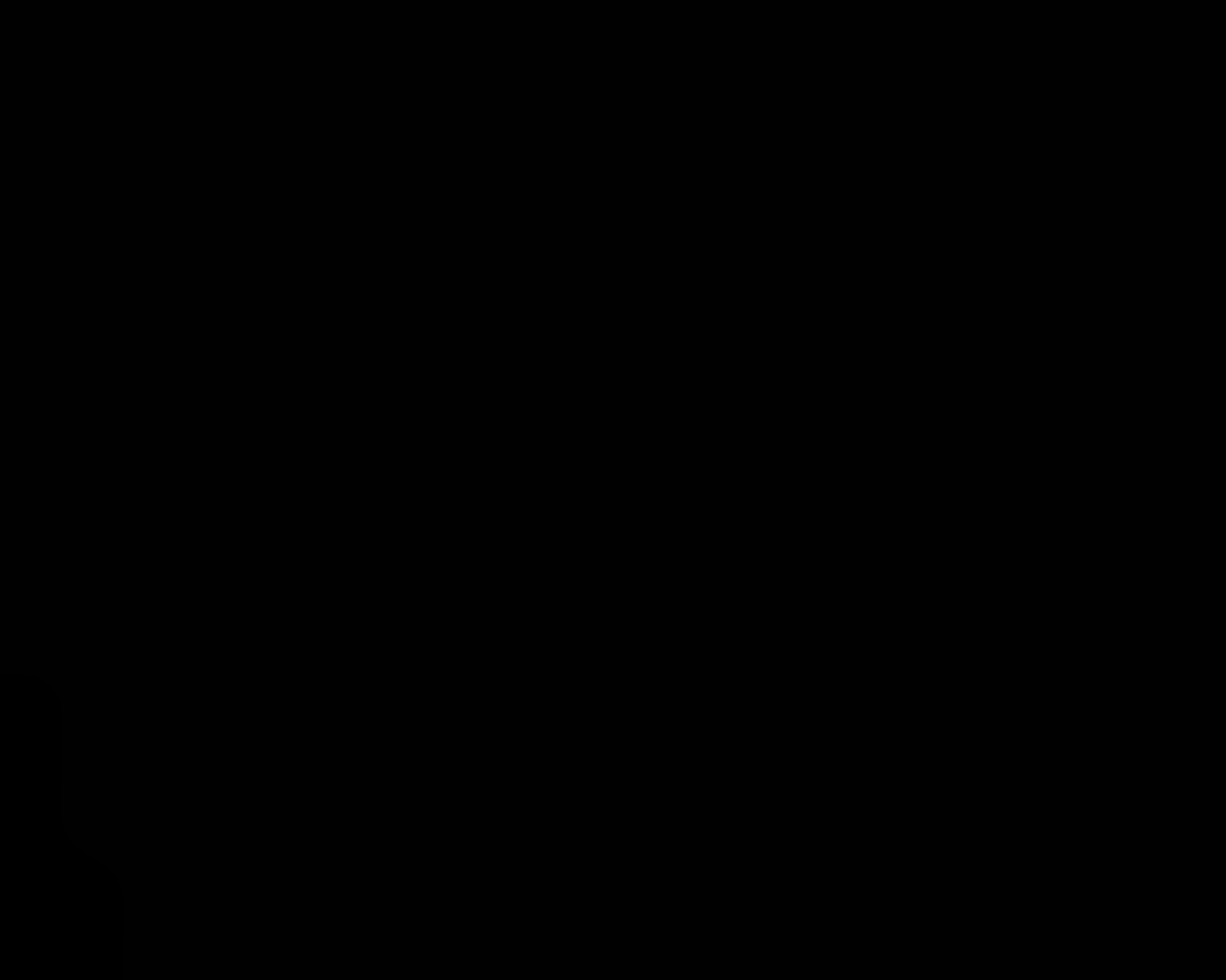

Supplement: S1 File — (ZIP) [file pone.0272206.s003.zip › new/BMI1/J - 11(fld 2 wv S475_20x - HQ535_50m).tif]

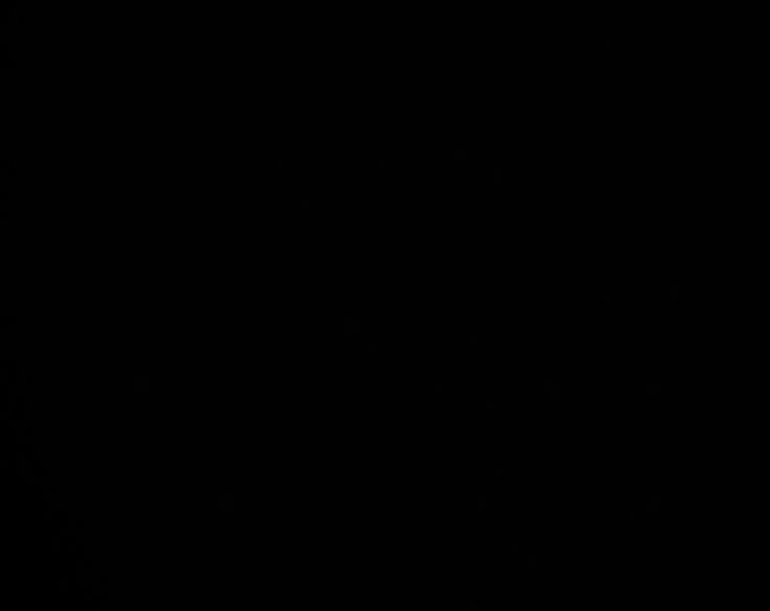

Supplement: S1 File — (ZIP) [file pone.0272206.s003.zip › new/BMI1/J - 11(fld 2 wv S475_20x - HQ535_50m)_thumb.tif]

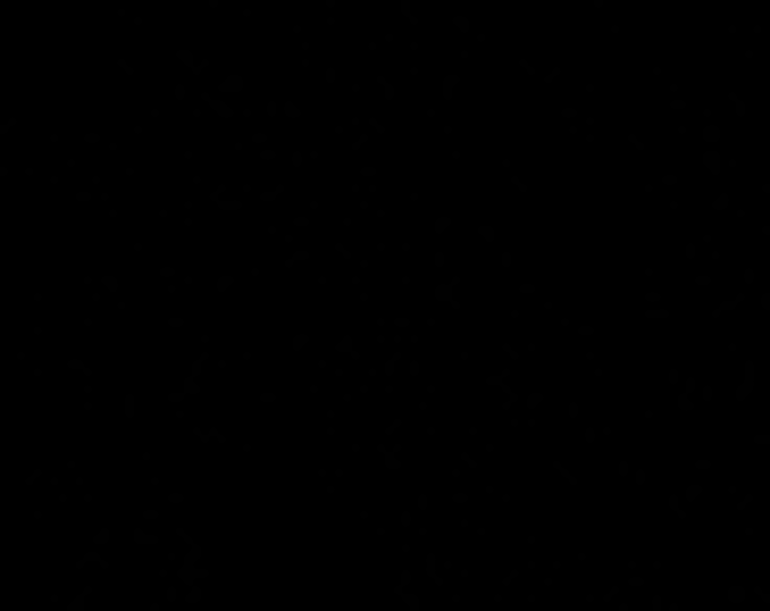

Supplement: S1 File — (ZIP) [file pone.0272206.s003.zip › new/BMI1/J - 11(fld 3 wv D360_40x - HQ460_40m)_thumb.tif]

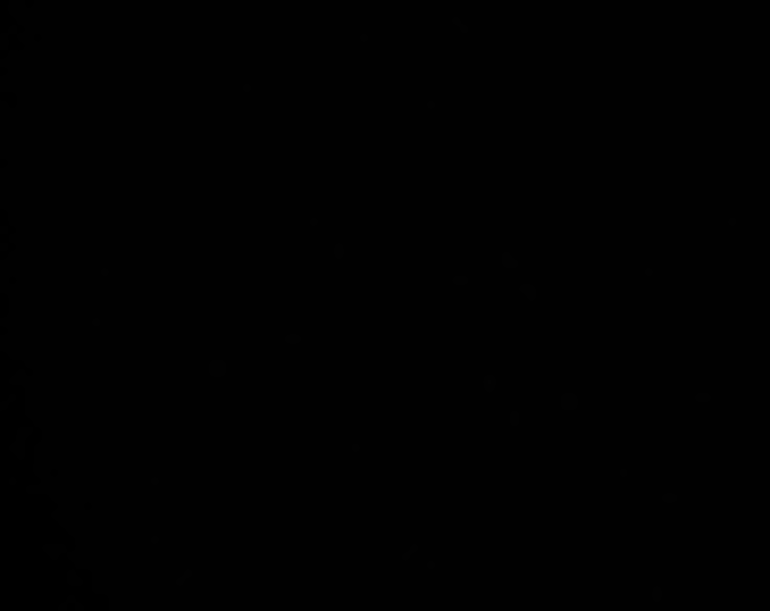

Supplement: S1 File — (ZIP) [file pone.0272206.s003.zip › new/BMI1/J - 11(fld 3 wv S475_20x - HQ535_50m)_thumb.tif]

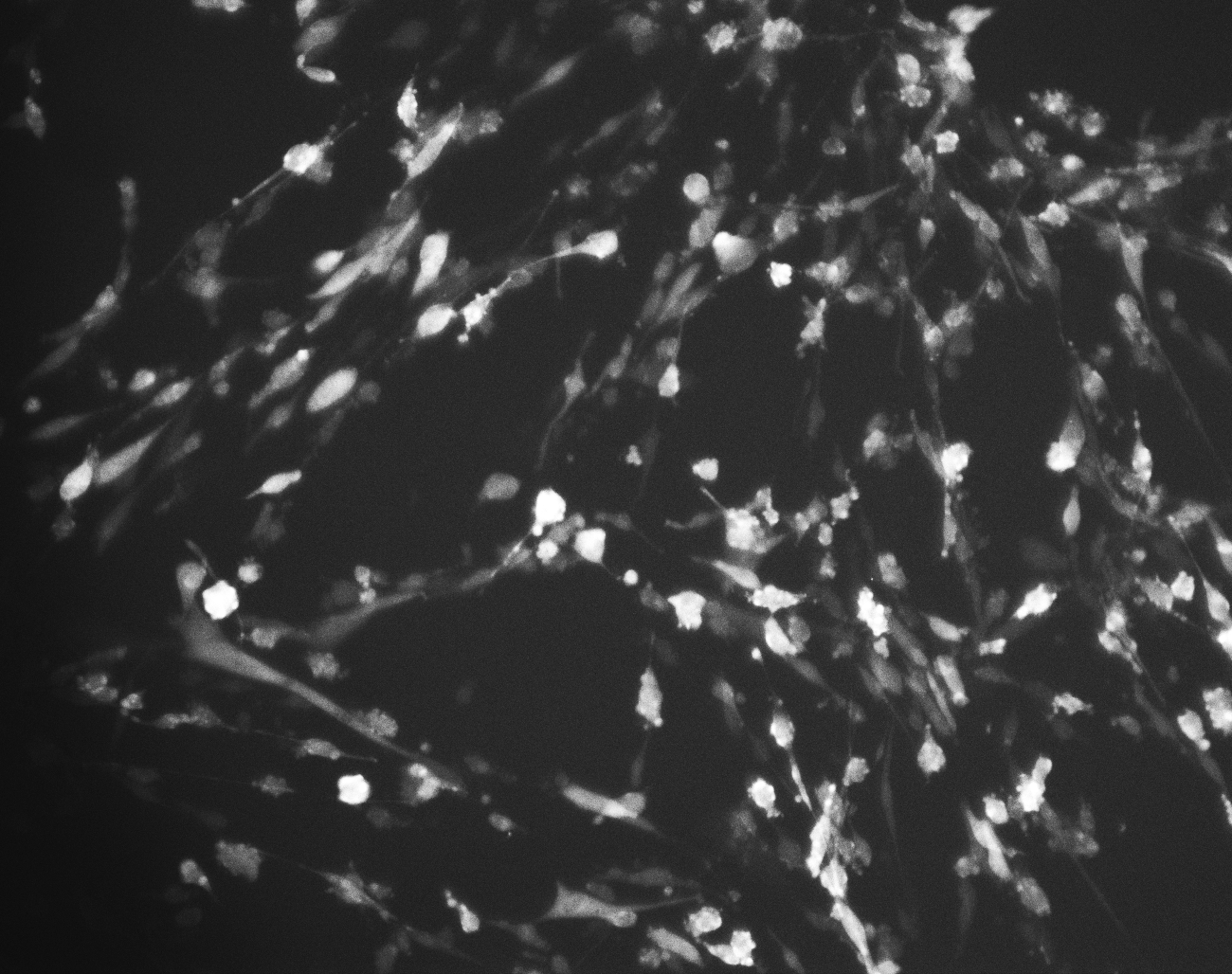

Supplement: S1 File — (ZIP) [file pone.0272206.s003.zip › new/BMI1/p16.tif]

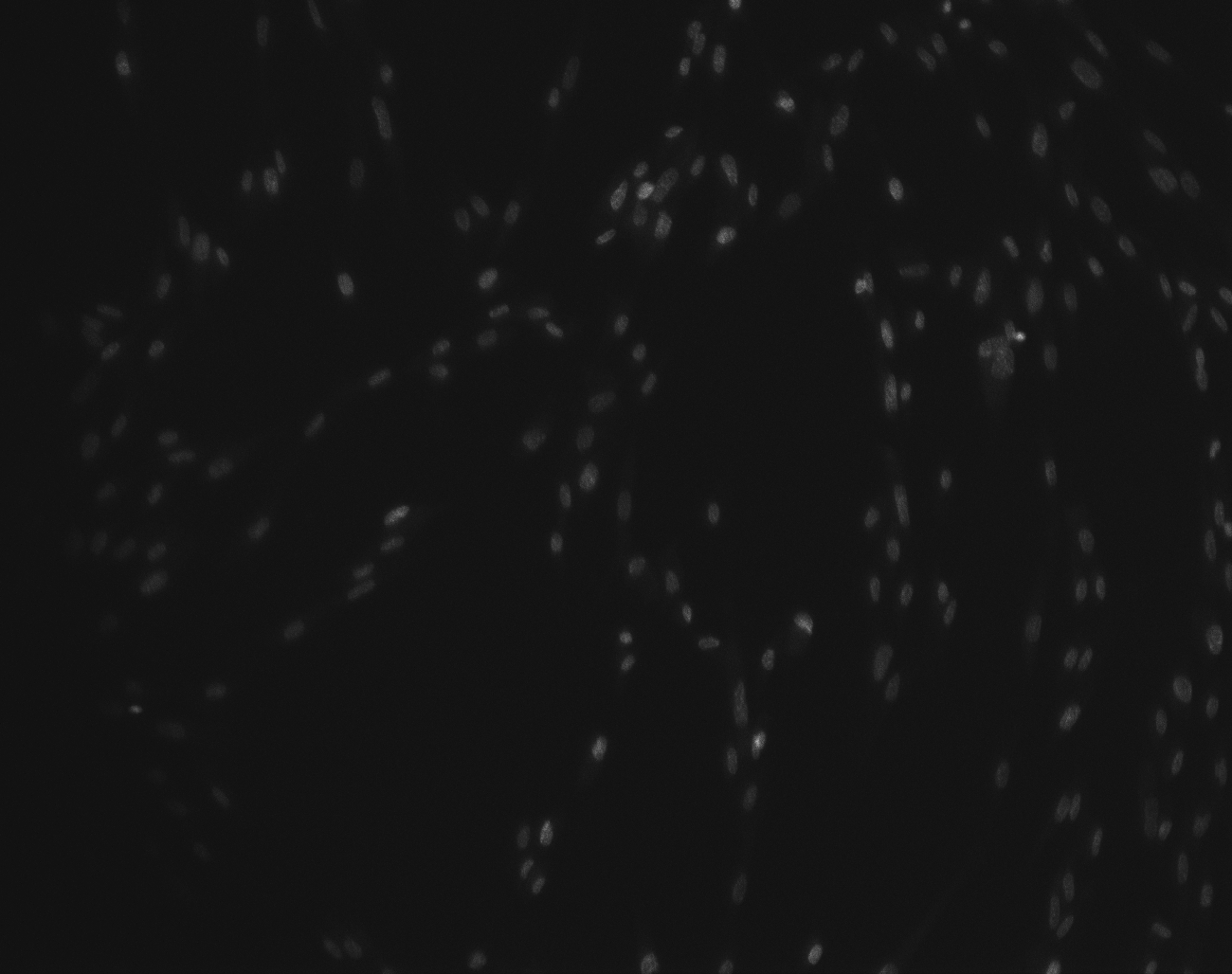

Supplement: S1 File — (ZIP) [file pone.0272206.s003.zip › new/Jmjd3/dapi.tif]

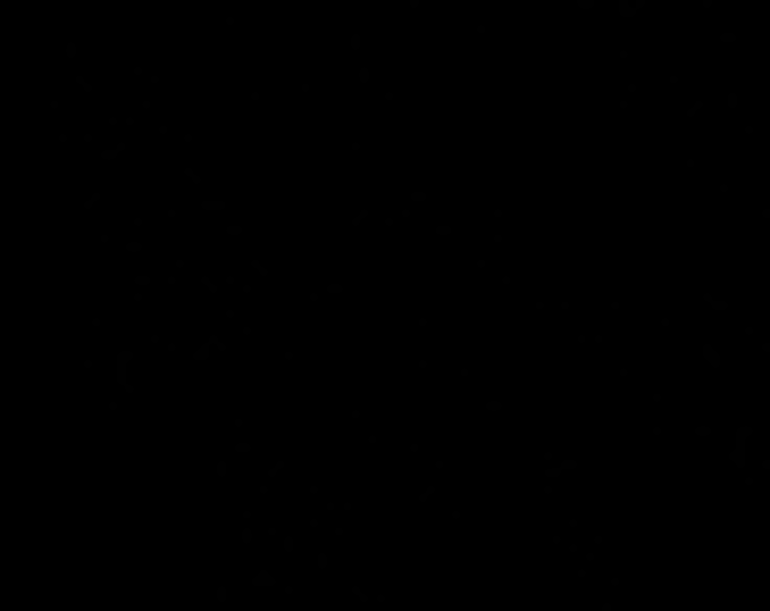

Supplement: S1 File — (ZIP) [file pone.0272206.s003.zip › new/Jmjd3/G - 23(fld 1 wv D360_40x - HQ460_40m)_thumb.tif]

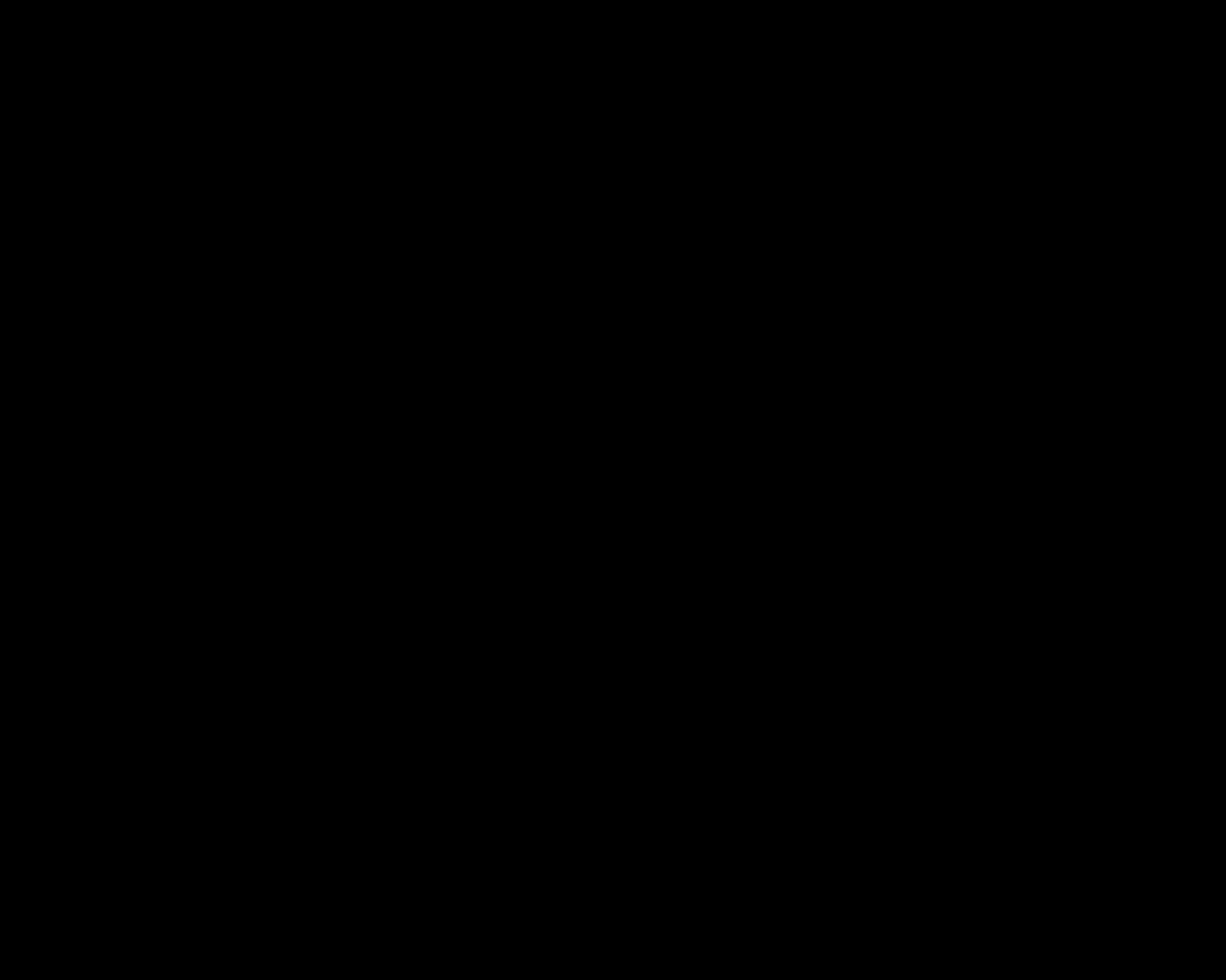

Supplement: S1 File — (ZIP) [file pone.0272206.s003.zip › new/Jmjd3/G - 23(fld 1 wv S475_20x - HQ535_50m).tif]

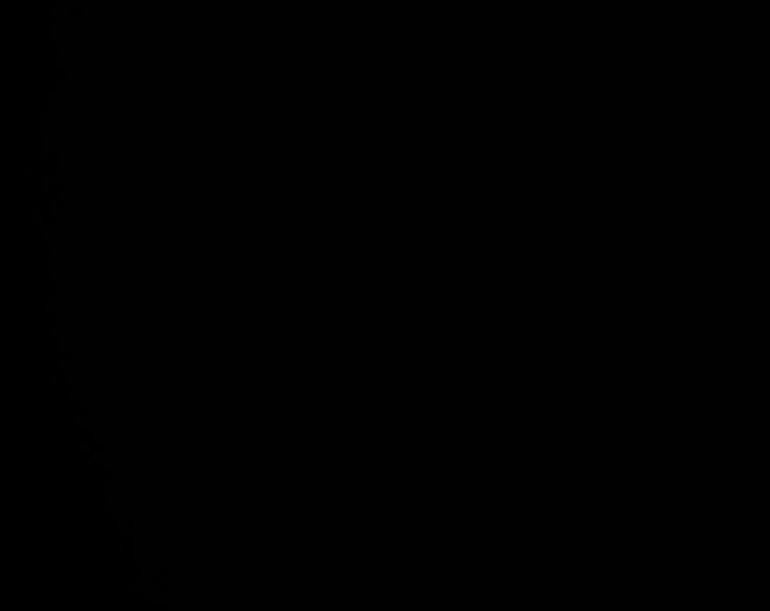

Supplement: S1 File — (ZIP) [file pone.0272206.s003.zip › new/Jmjd3/G - 23(fld 1 wv S475_20x - HQ535_50m)_thumb.tif]

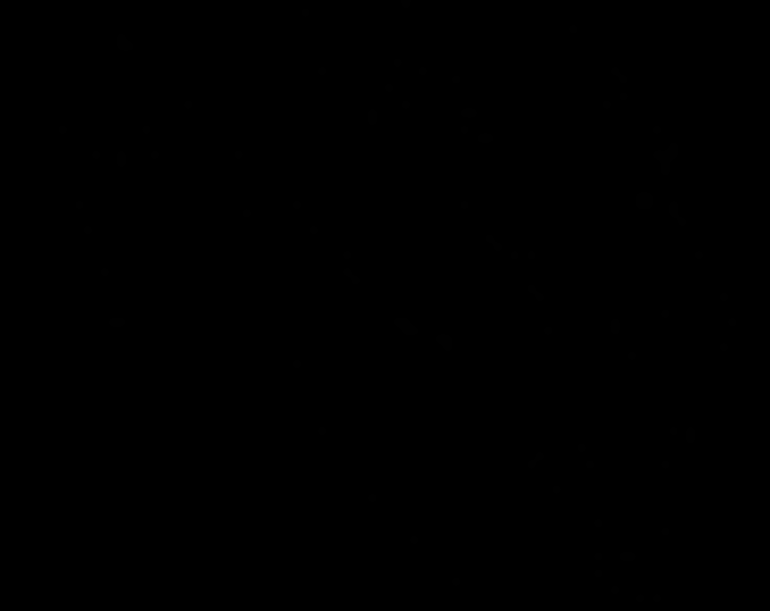

Supplement: S1 File — (ZIP) [file pone.0272206.s003.zip › new/Jmjd3/G - 23(fld 2 wv D360_40x - HQ460_40m)_thumb.tif]

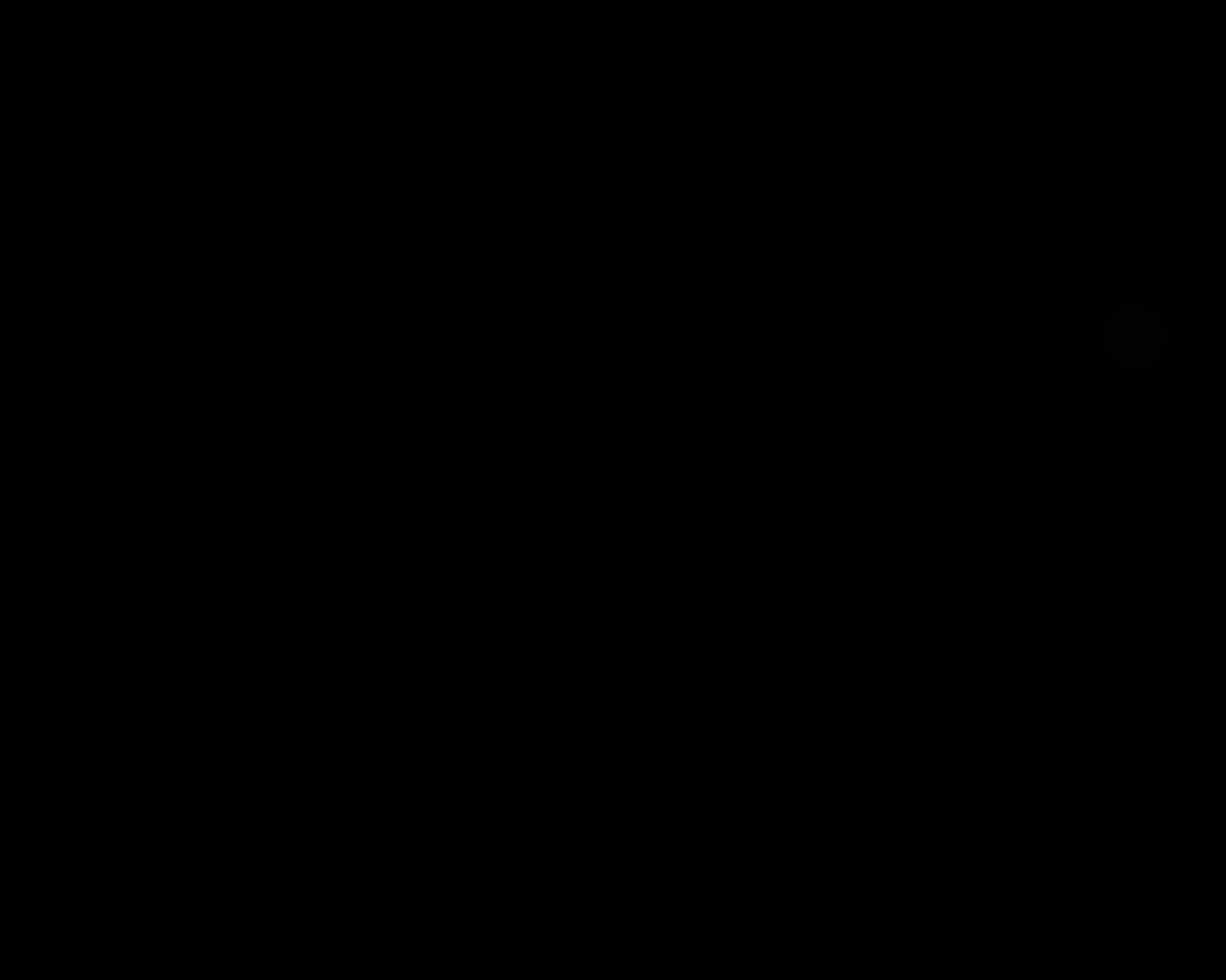

Supplement: S1 File — (ZIP) [file pone.0272206.s003.zip › new/Jmjd3/G - 23(fld 2 wv S475_20x - HQ535_50m).tif]

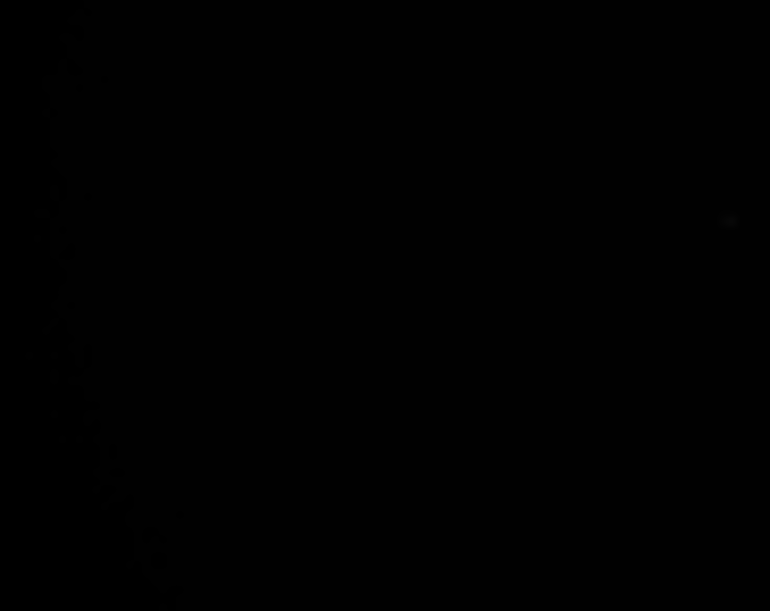

Supplement: S1 File — (ZIP) [file pone.0272206.s003.zip › new/Jmjd3/G - 23(fld 2 wv S475_20x - HQ535_50m)_thumb.tif]

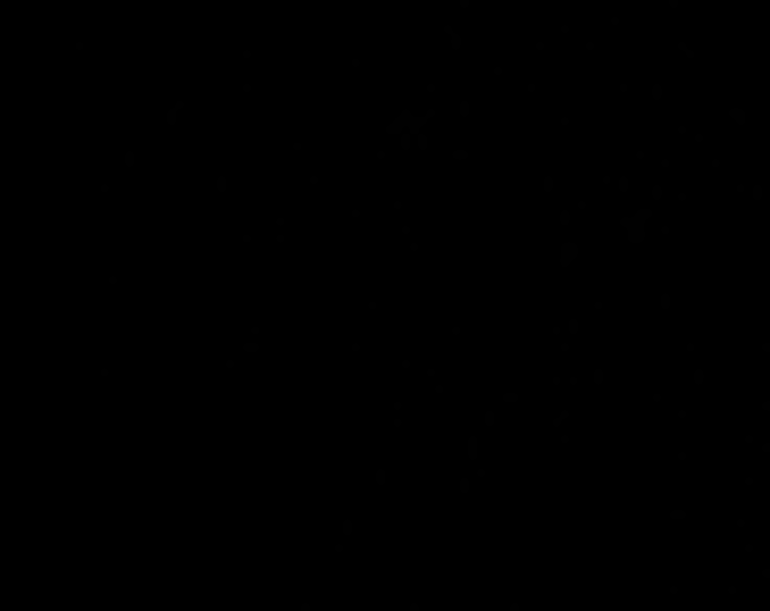

Supplement: S1 File — (ZIP) [file pone.0272206.s003.zip › new/Jmjd3/G - 23(fld 3 wv D360_40x - HQ460_40m)_thumb.tif]

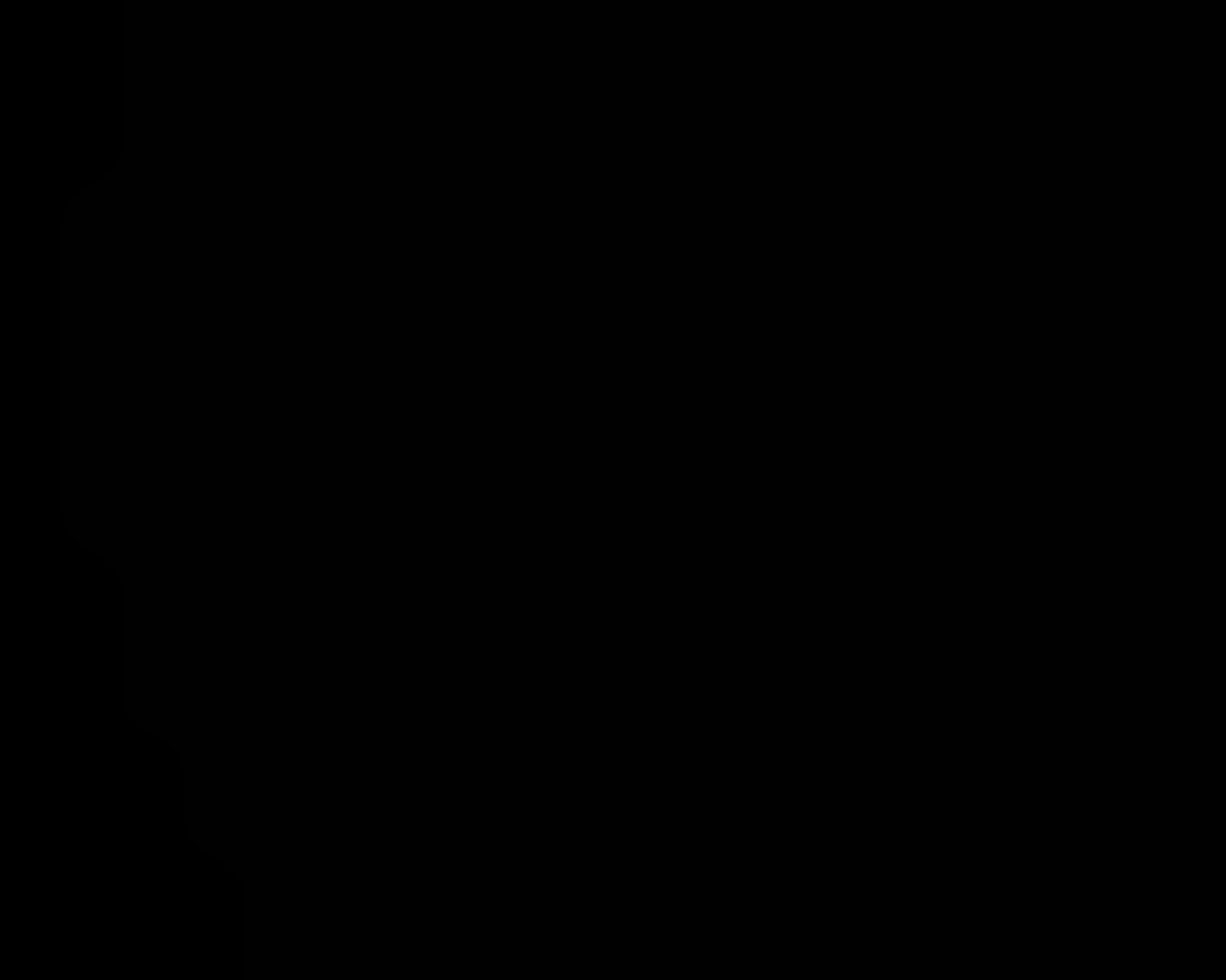

Supplement: S1 File — (ZIP) [file pone.0272206.s003.zip › new/Jmjd3/G - 23(fld 3 wv S475_20x - HQ535_50m).tif]

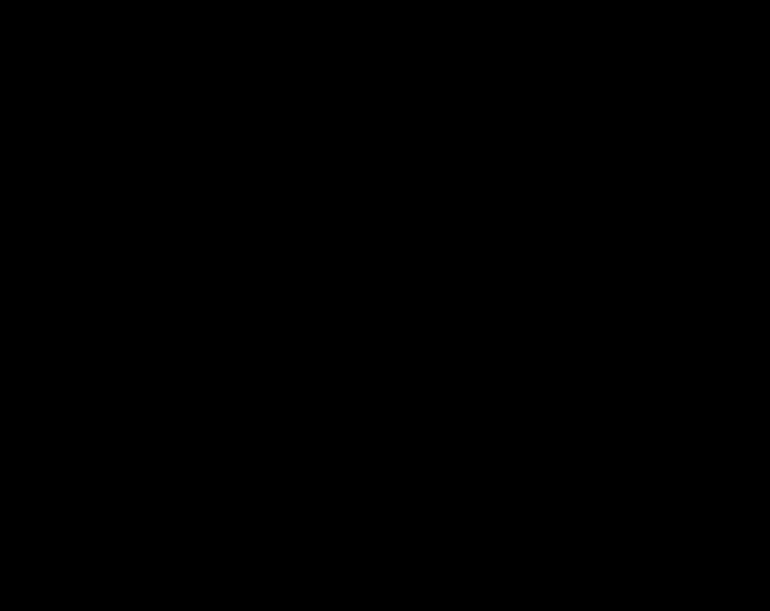

Supplement: S1 File — (ZIP) [file pone.0272206.s003.zip › new/Jmjd3/G - 23(fld 3 wv S475_20x - HQ535_50m)_thumb.tif]

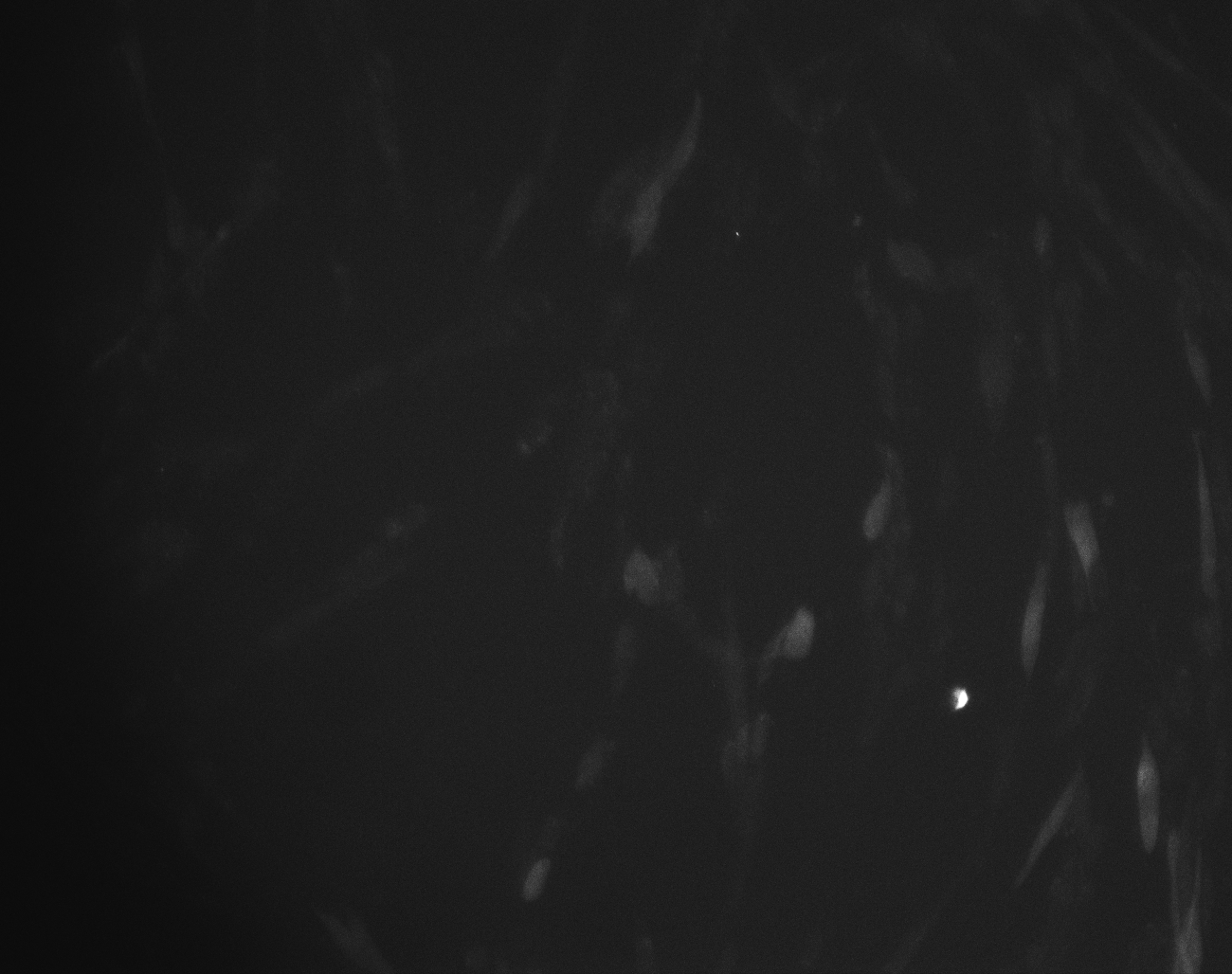

Supplement: S1 File — (ZIP) [file pone.0272206.s003.zip › new/Jmjd3/p16.tif]

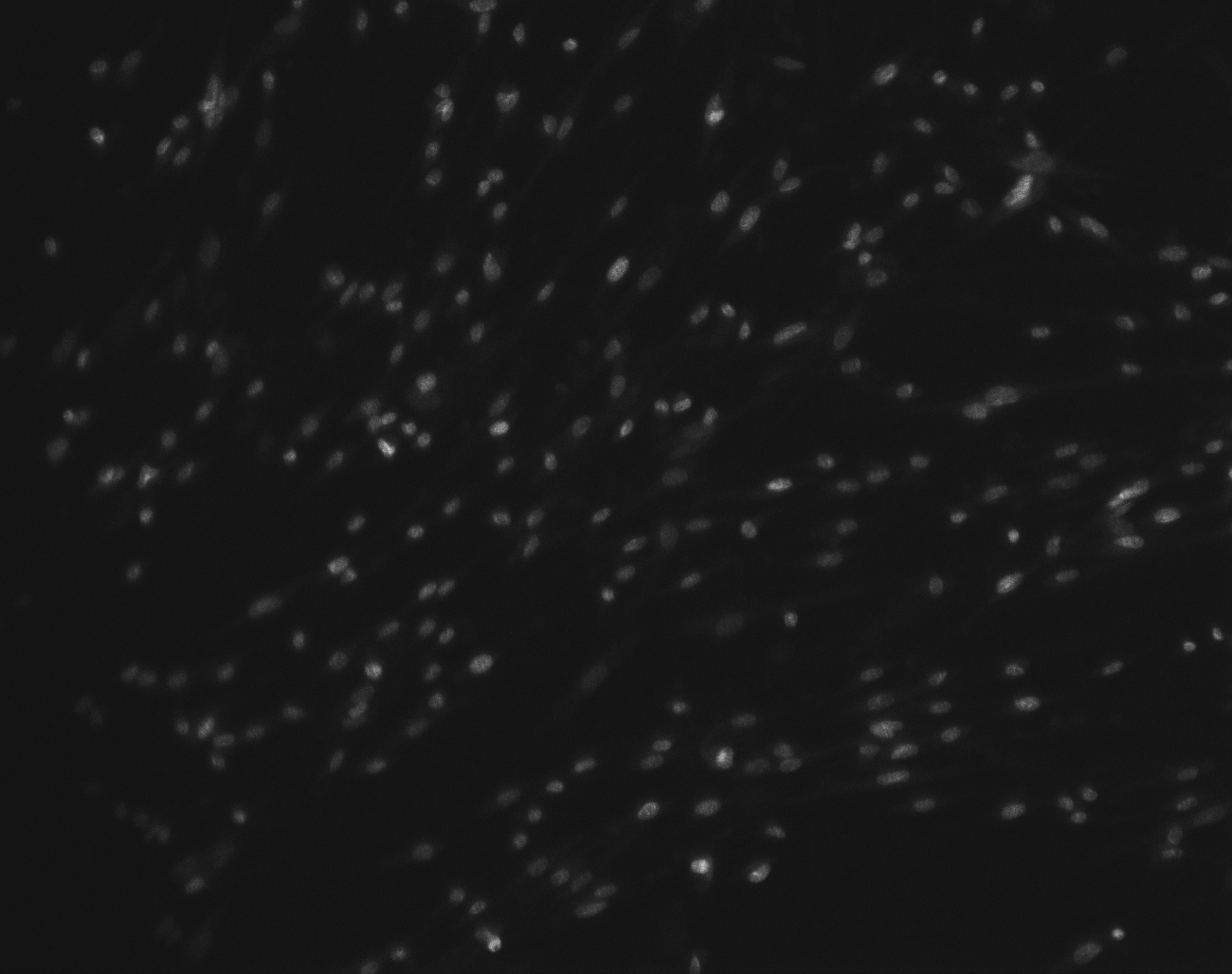

Supplement: S1 File — (ZIP) [file pone.0272206.s003.zip › new/miR129/dapi.tif]

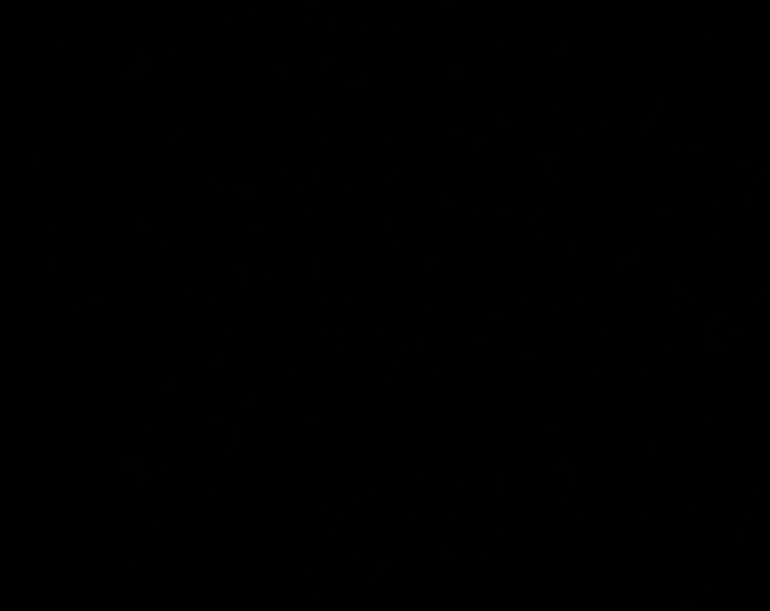

Supplement: S1 File — (ZIP) [file pone.0272206.s003.zip › new/miR129/E - 12(fld 1 wv D360_40x - HQ460_40m)_thumb.tif]

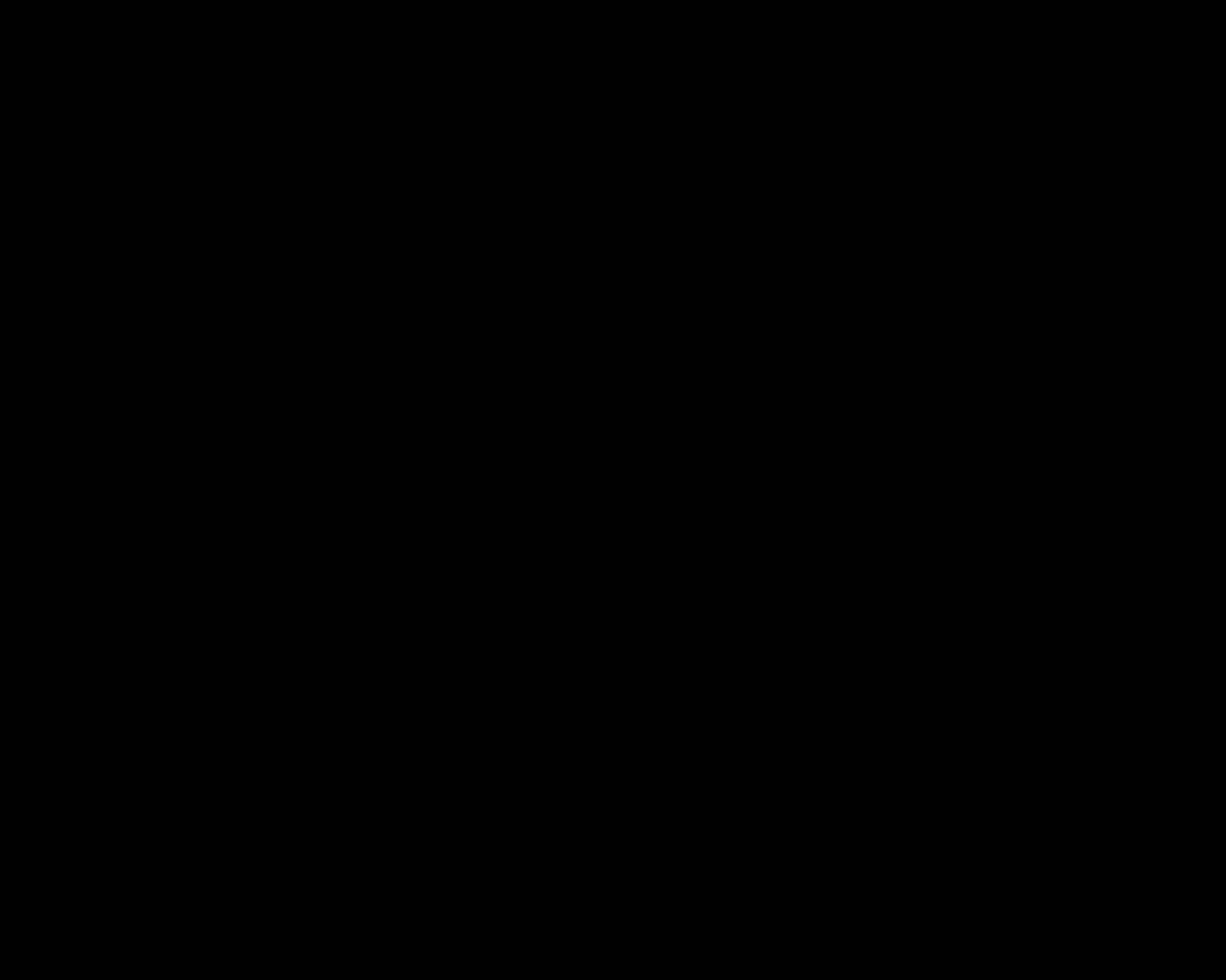

Supplement: S1 File — (ZIP) [file pone.0272206.s003.zip › new/miR129/E - 12(fld 1 wv S475_20x - HQ535_50m).tif]

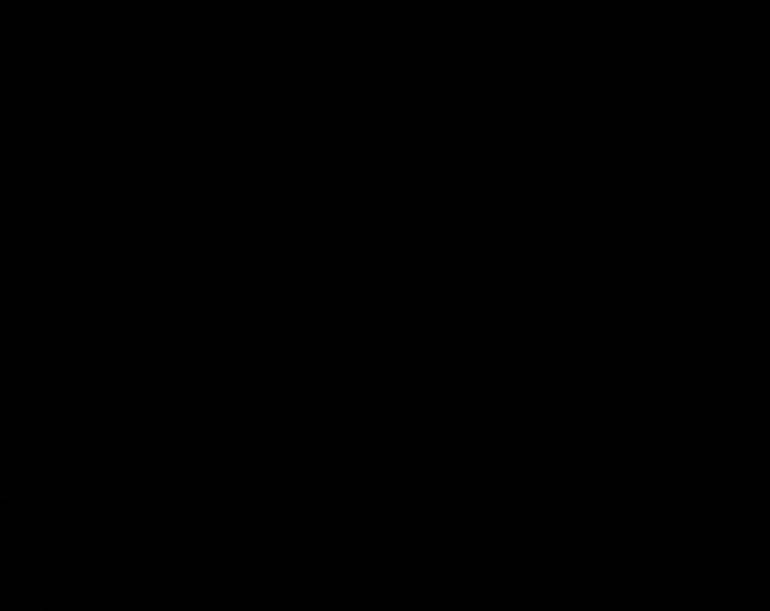

Supplement: S1 File — (ZIP) [file pone.0272206.s003.zip › new/miR129/E - 12(fld 1 wv S475_20x - HQ535_50m)_thumb.tif]

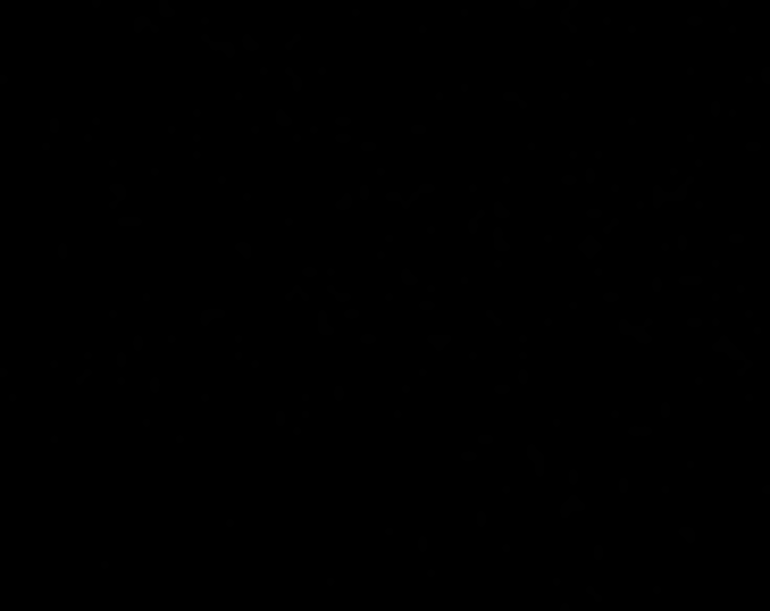

Supplement: S1 File — (ZIP) [file pone.0272206.s003.zip › new/miR129/E - 12(fld 2 wv D360_40x - HQ460_40m)_thumb.tif]

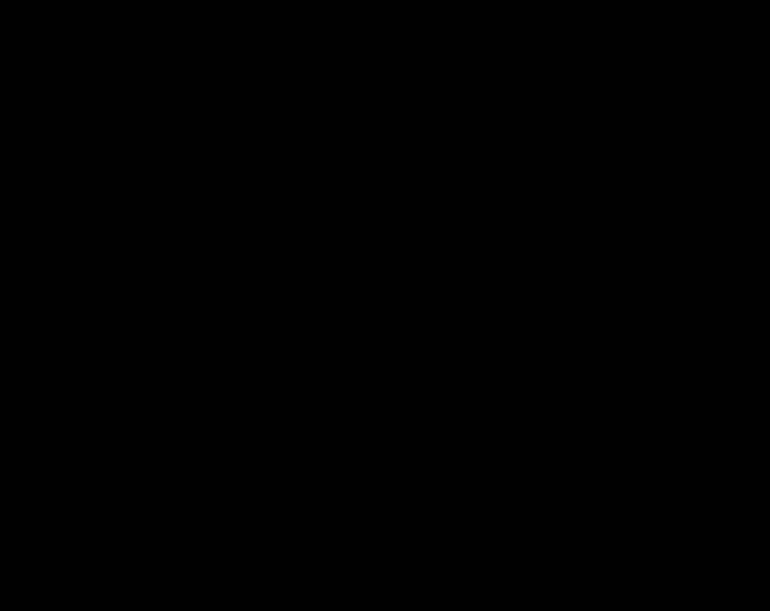

Supplement: S1 File — (ZIP) [file pone.0272206.s003.zip › new/miR129/E - 12(fld 2 wv S475_20x - HQ535_50m)_thumb.tif]

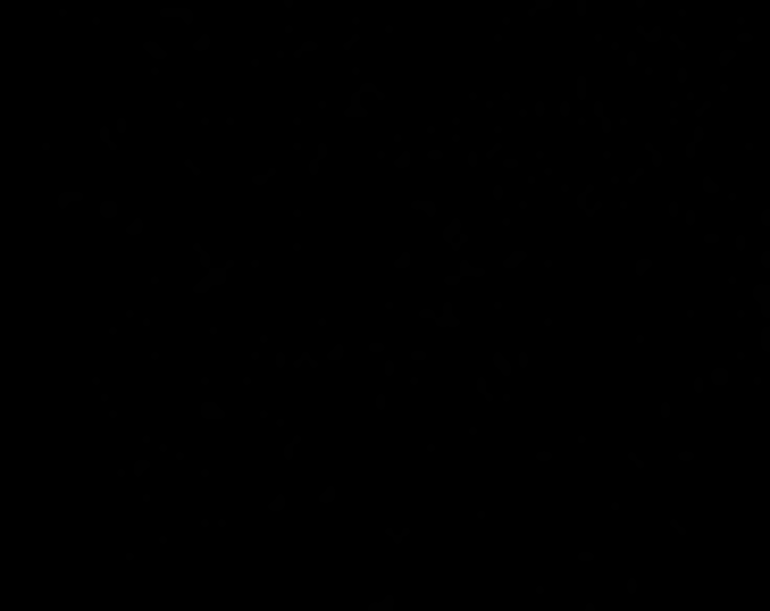

Supplement: S1 File — (ZIP) [file pone.0272206.s003.zip › new/miR129/E - 12(fld 3 wv D360_40x - HQ460_40m)_thumb.tif]

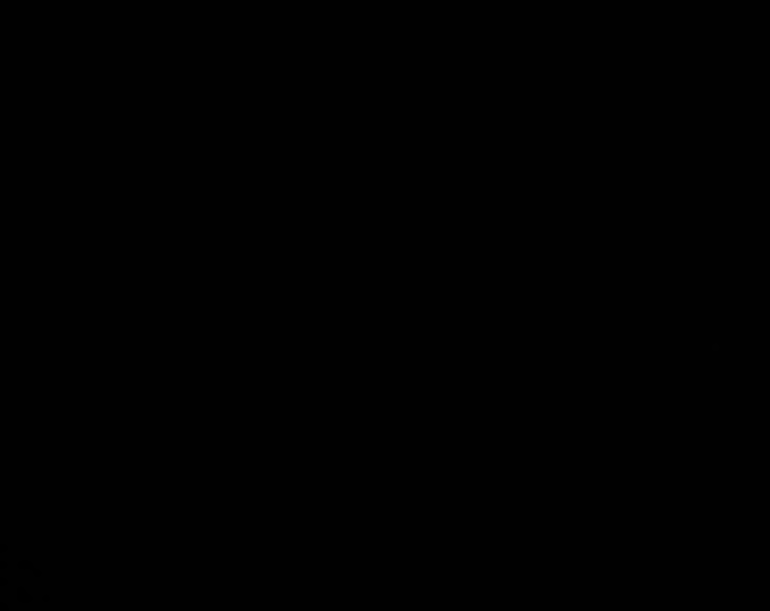

Supplement: S1 File — (ZIP) [file pone.0272206.s003.zip › new/miR129/E - 12(fld 3 wv S475_20x - HQ535_50m)_thumb.tif]

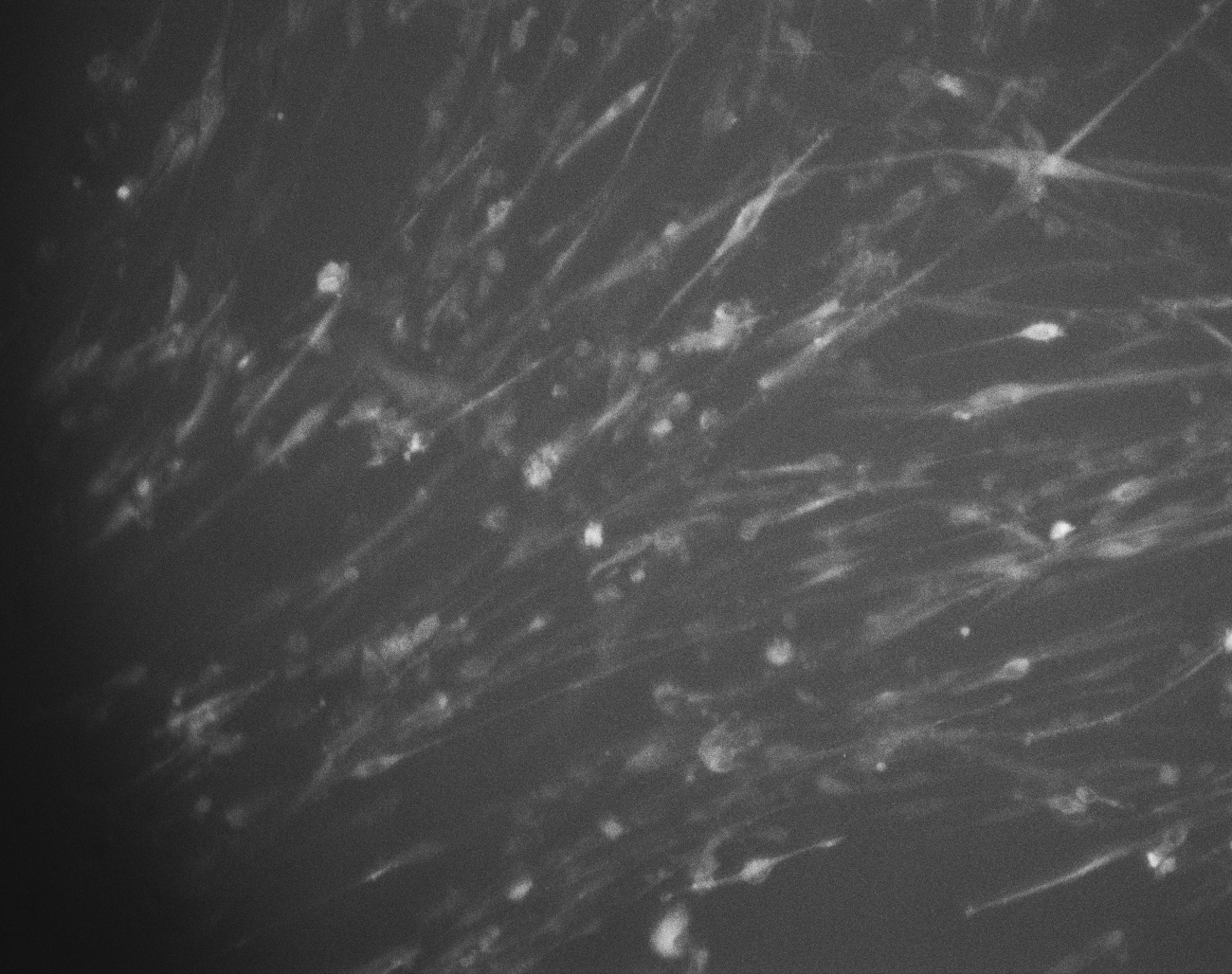

Supplement: S1 File — (ZIP) [file pone.0272206.s003.zip › new/miR129/p16.tif]

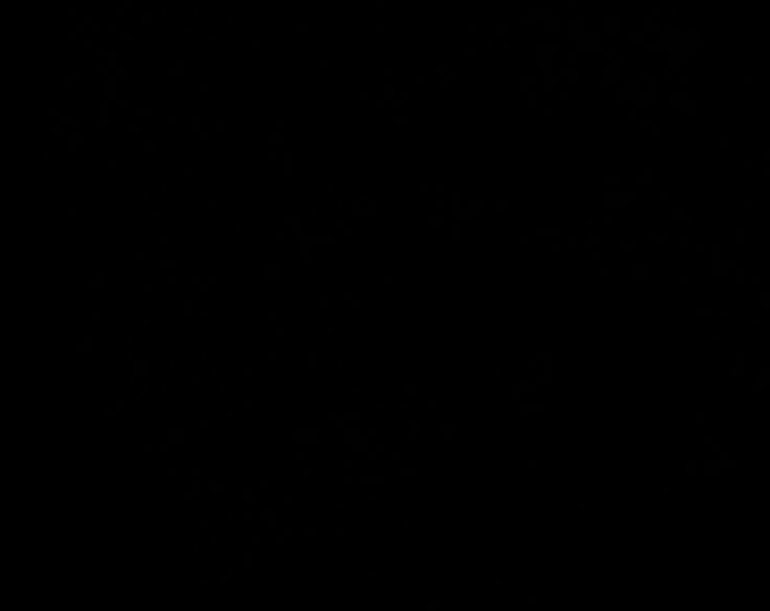

Supplement: S1 File — (ZIP) [file pone.0272206.s003.zip › new/mir200c/D - 7(fld 1 wv D360_40x - HQ460_40m)_thumb.tif]

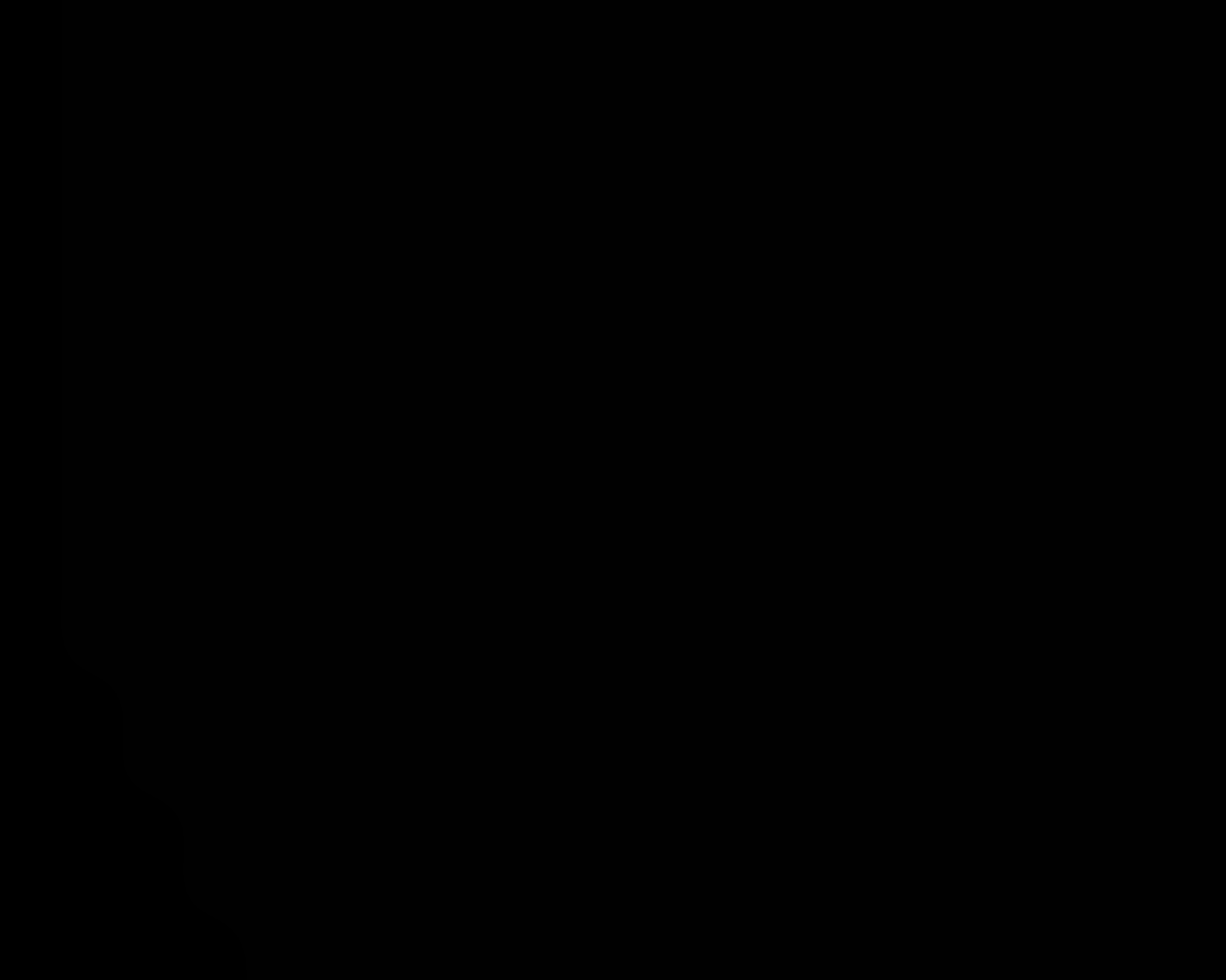

Supplement: S1 File — (ZIP) [file pone.0272206.s003.zip › new/mir200c/D - 7(fld 1 wv S475_20x - HQ535_50m).tif]

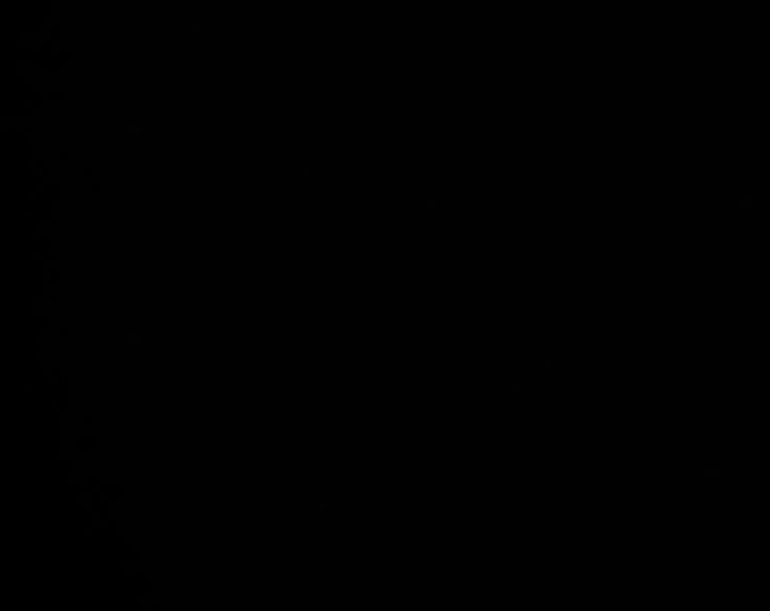

Supplement: S1 File — (ZIP) [file pone.0272206.s003.zip › new/mir200c/D - 7(fld 1 wv S475_20x - HQ535_50m)_thumb.tif]

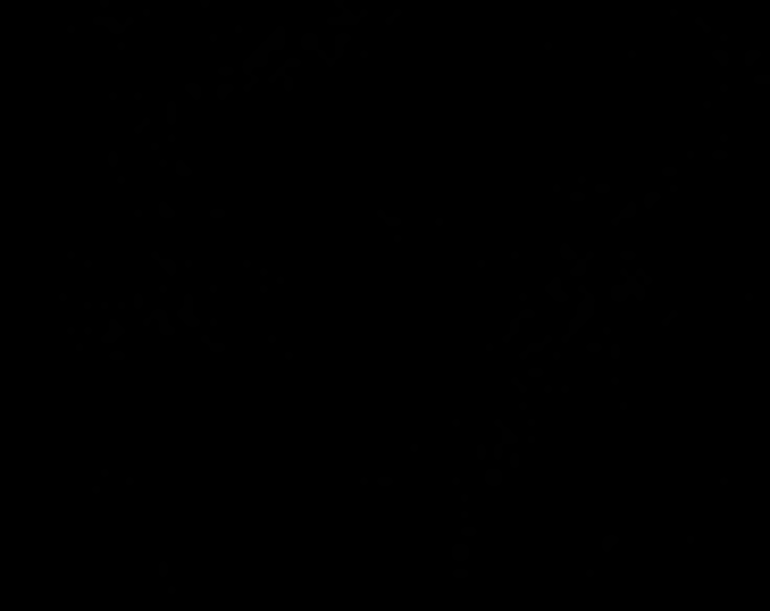

Supplement: S1 File — (ZIP) [file pone.0272206.s003.zip › new/mir200c/D - 7(fld 2 wv D360_40x - HQ460_40m)_thumb.tif]

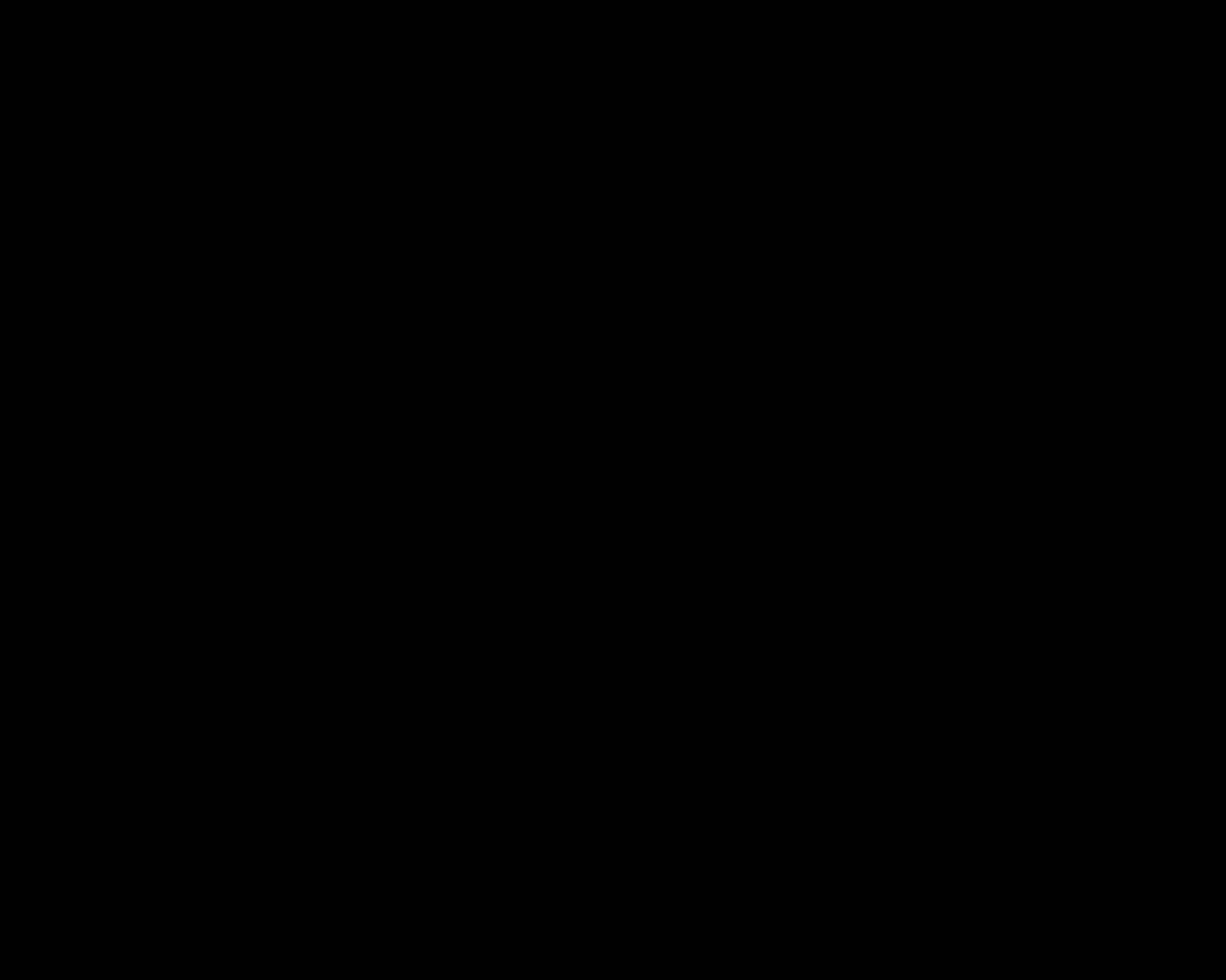

Supplement: S1 File — (ZIP) [file pone.0272206.s003.zip › new/mir200c/D - 7(fld 2 wv S475_20x - HQ535_50m).tif]

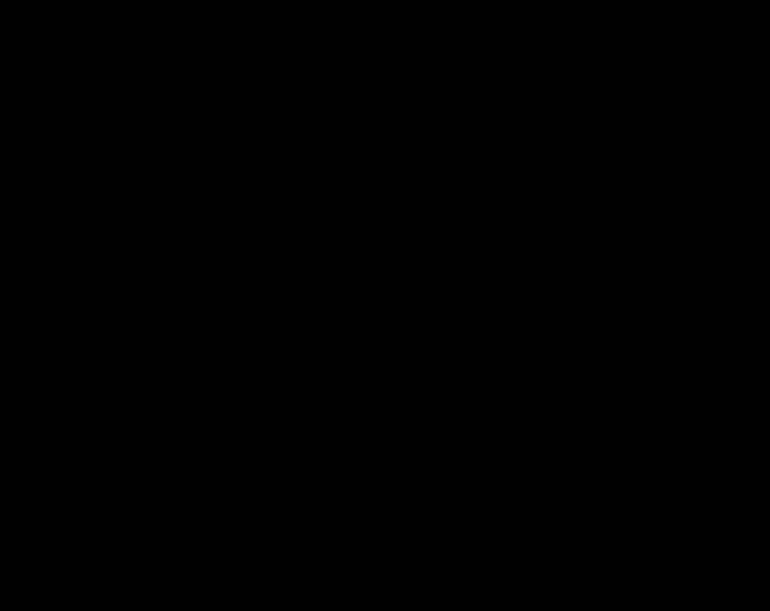

Supplement: S1 File — (ZIP) [file pone.0272206.s003.zip › new/mir200c/D - 7(fld 2 wv S475_20x - HQ535_50m)_thumb.tif]

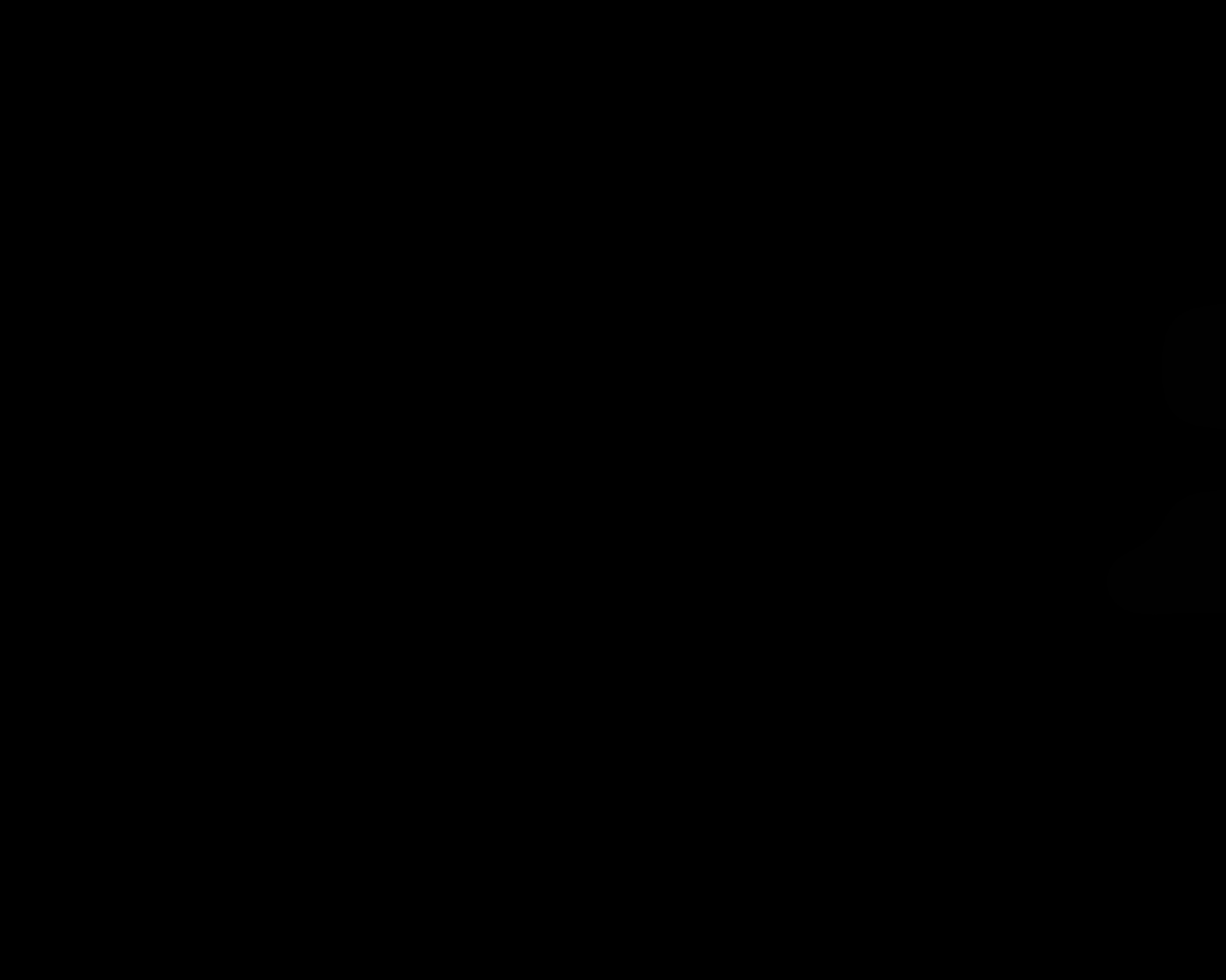

Supplement: S1 File — (ZIP) [file pone.0272206.s003.zip › new/mir200c/D - 7(fld 3 wv D360_40x - HQ460_40m).tif]

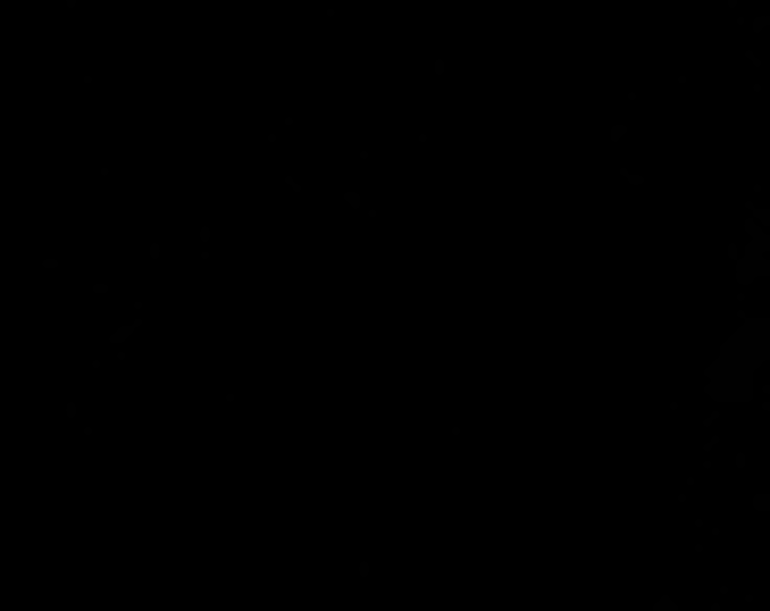

Supplement: S1 File — (ZIP) [file pone.0272206.s003.zip › new/mir200c/D - 7(fld 3 wv D360_40x - HQ460_40m)_thumb.tif]

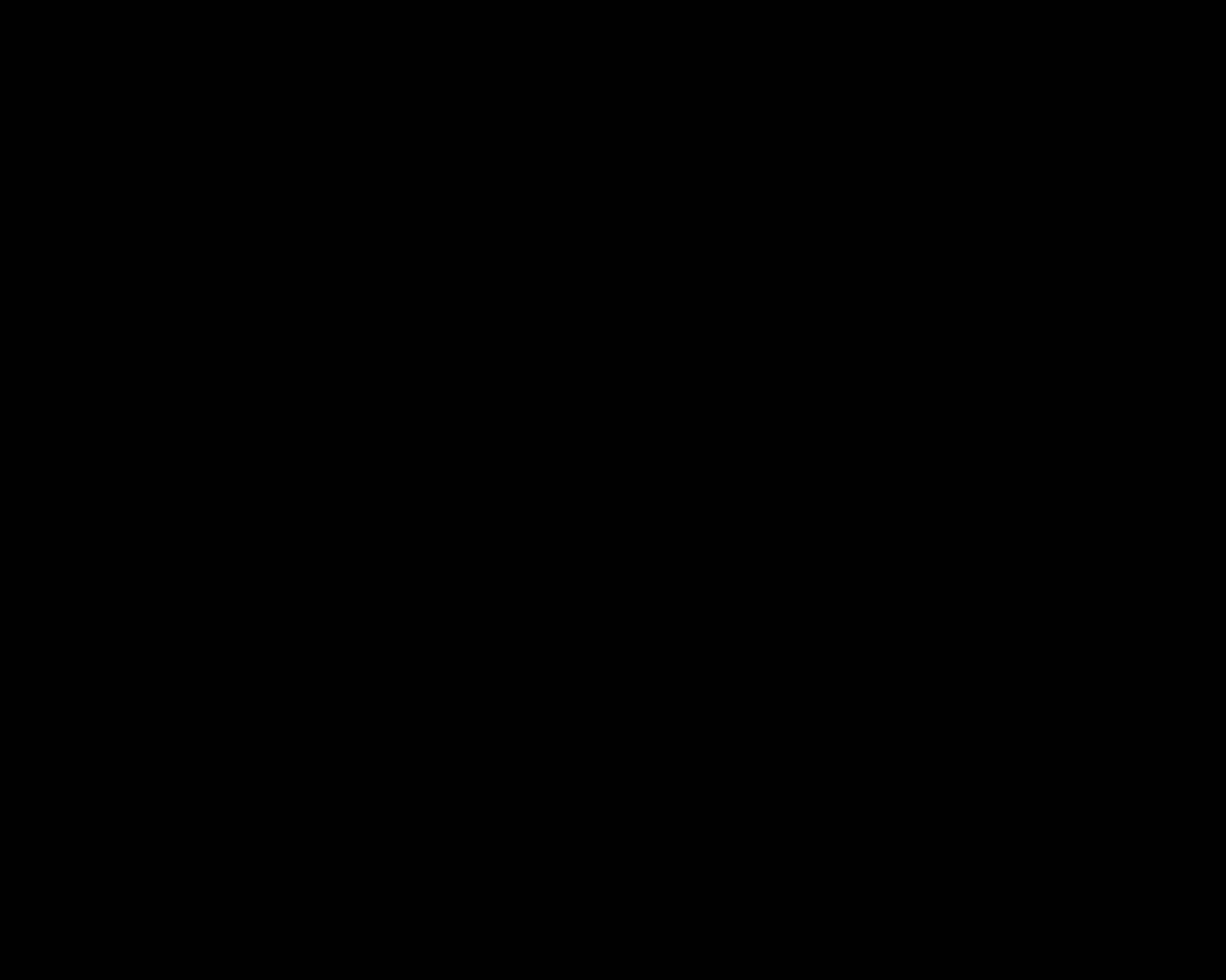

Supplement: S1 File — (ZIP) [file pone.0272206.s003.zip › new/mir200c/D - 7(fld 3 wv S475_20x - HQ535_50m).tif]

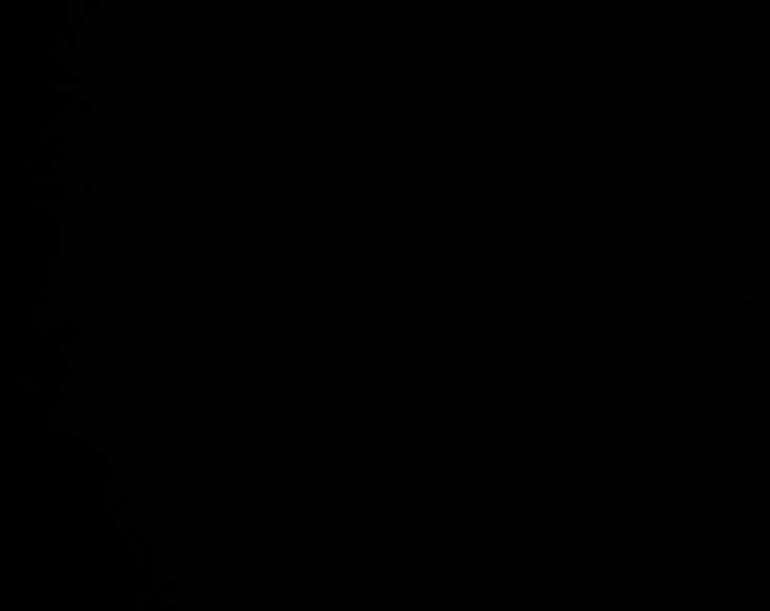

Supplement: S1 File — (ZIP) [file pone.0272206.s003.zip › new/mir200c/D - 7(fld 3 wv S475_20x - HQ535_50m)_thumb.tif]

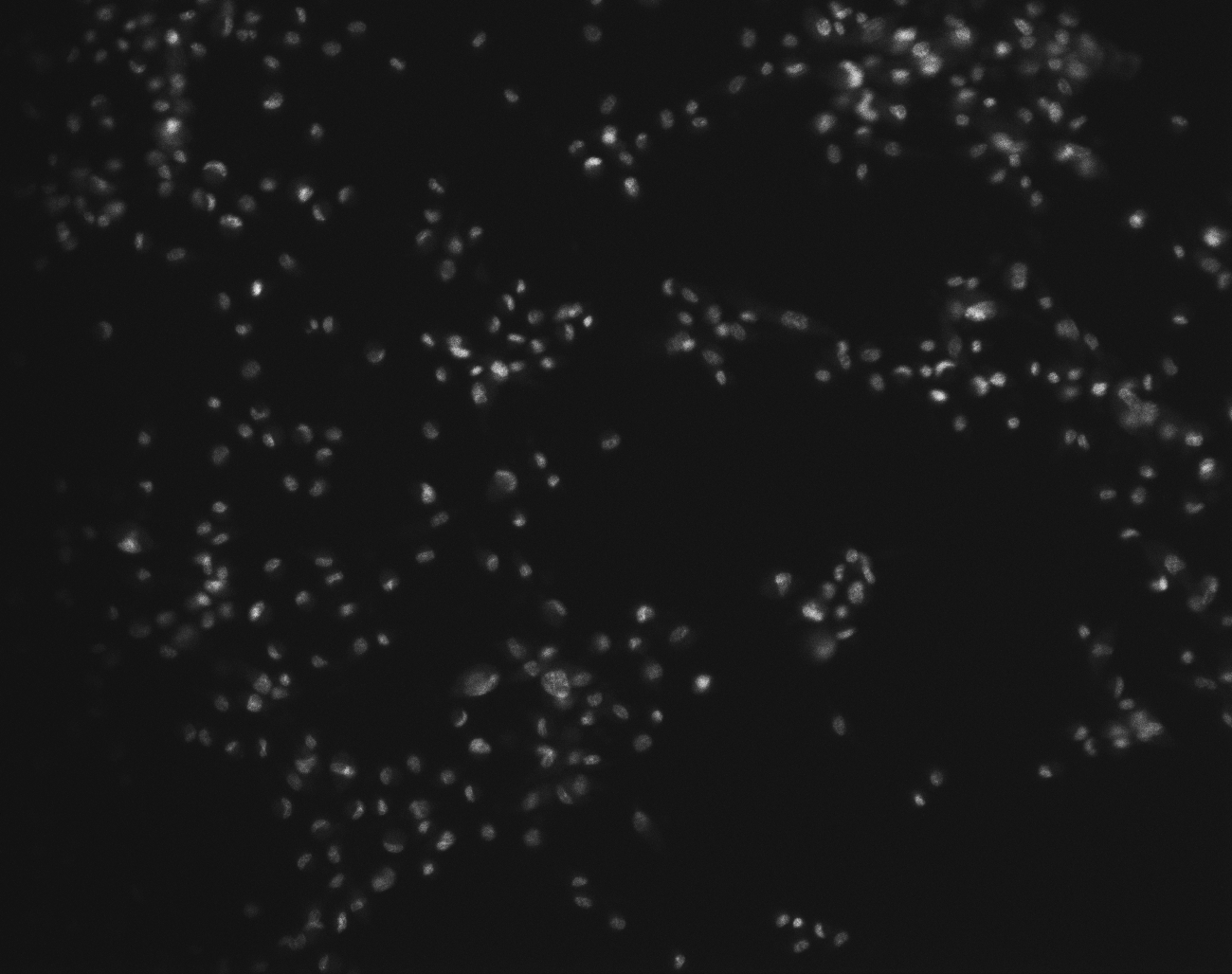

Supplement: S1 File — (ZIP) [file pone.0272206.s003.zip › new/mir200c/dapi.tif]

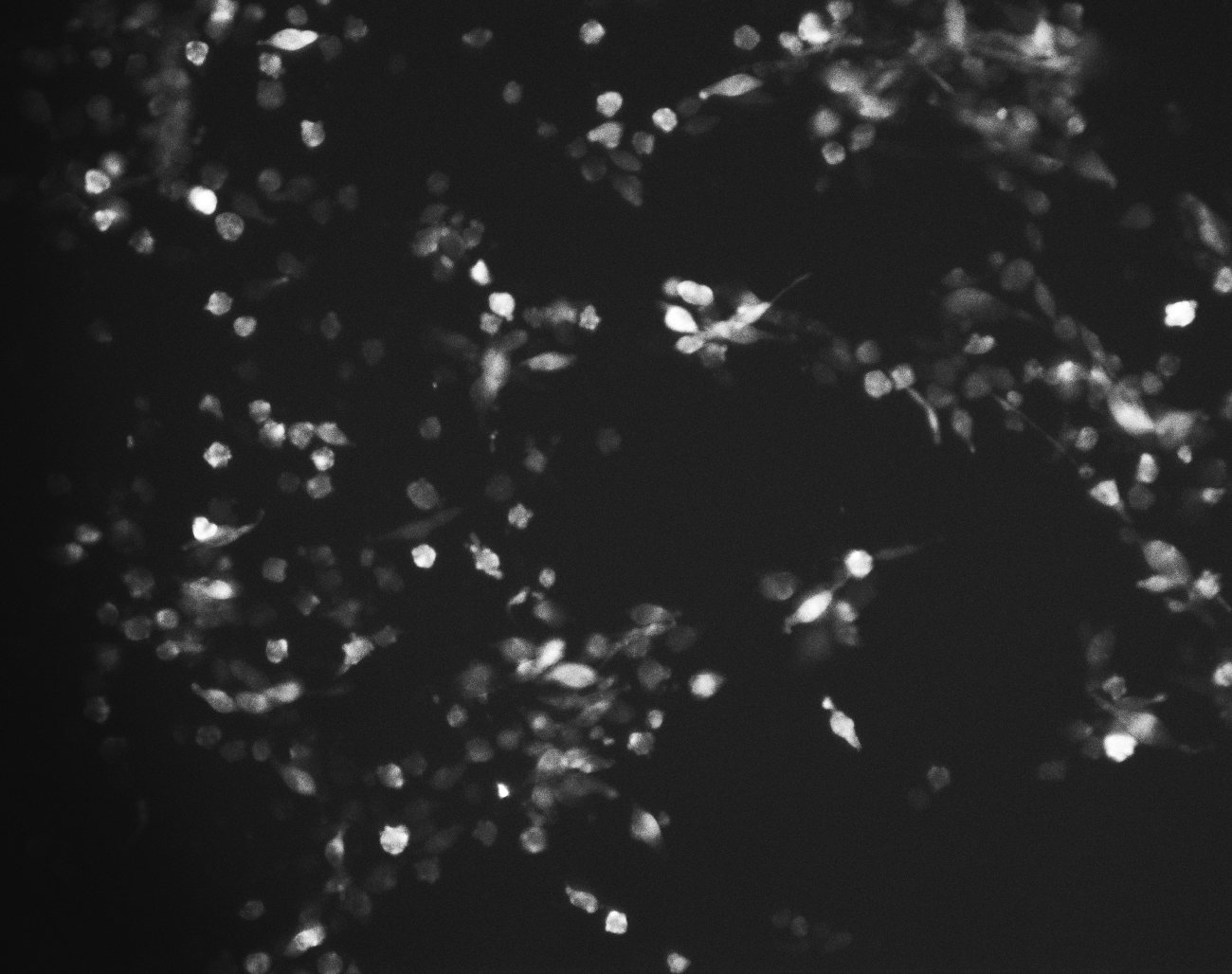

Supplement: S1 File — (ZIP) [file pone.0272206.s003.zip › new/mir200c/p16.tif]

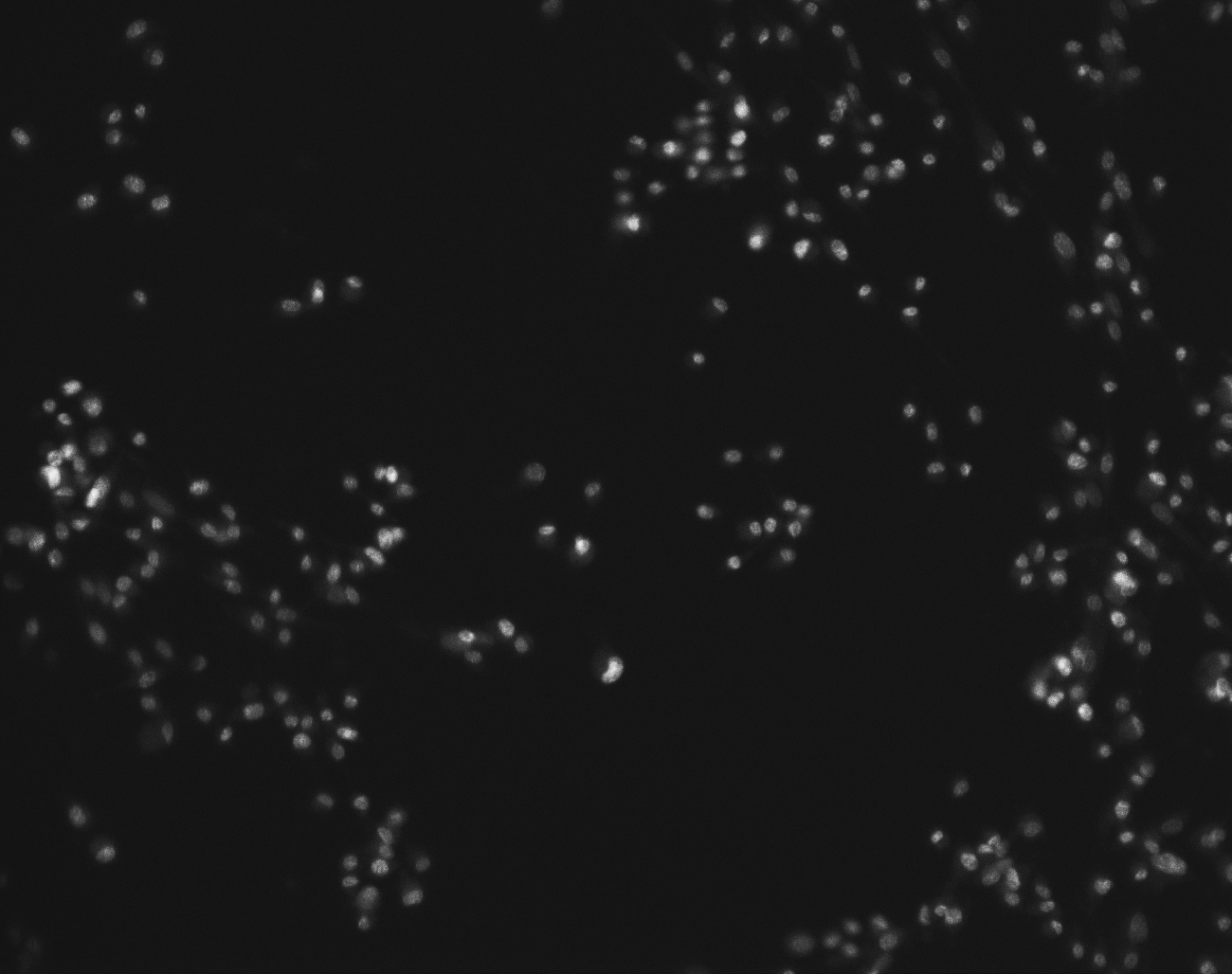

Supplement: S1 File — (ZIP) [file pone.0272206.s003.zip › new/miR297/dapi.tif]

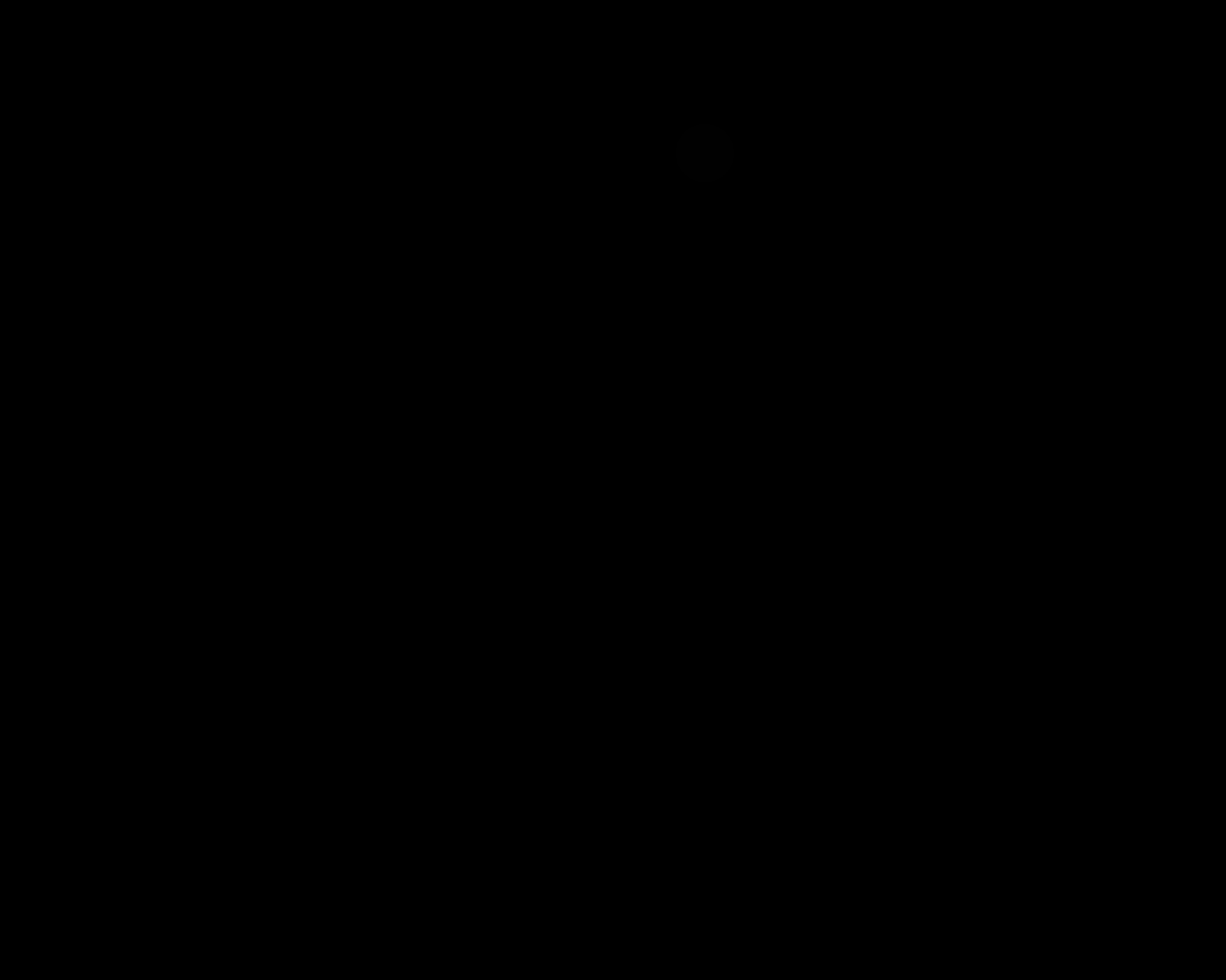

Supplement: S1 File — (ZIP) [file pone.0272206.s003.zip › new/miR297/E - 17(fld 1 wv D360_40x - HQ460_40m).tif]

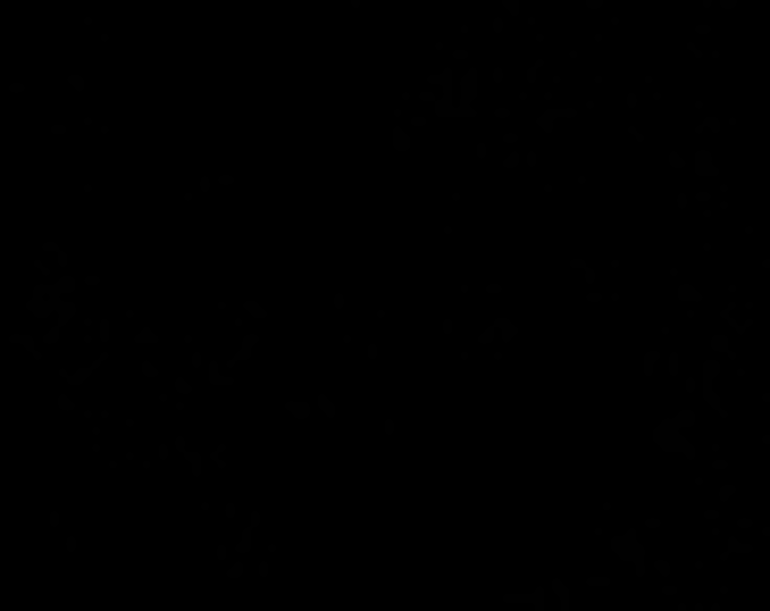

Supplement: S1 File — (ZIP) [file pone.0272206.s003.zip › new/miR297/E - 17(fld 1 wv D360_40x - HQ460_40m)_thumb.tif]

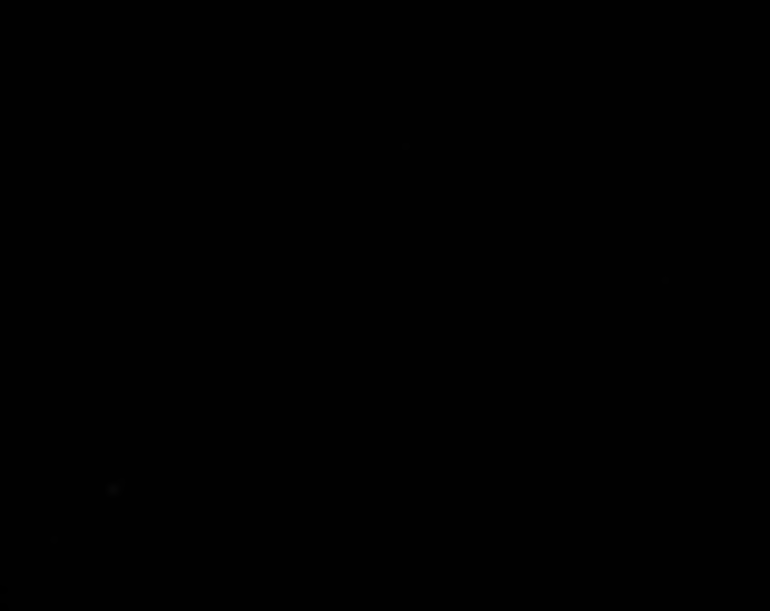

Supplement: S1 File — (ZIP) [file pone.0272206.s003.zip › new/miR297/E - 17(fld 1 wv S475_20x - HQ535_50m)_thumb.tif]

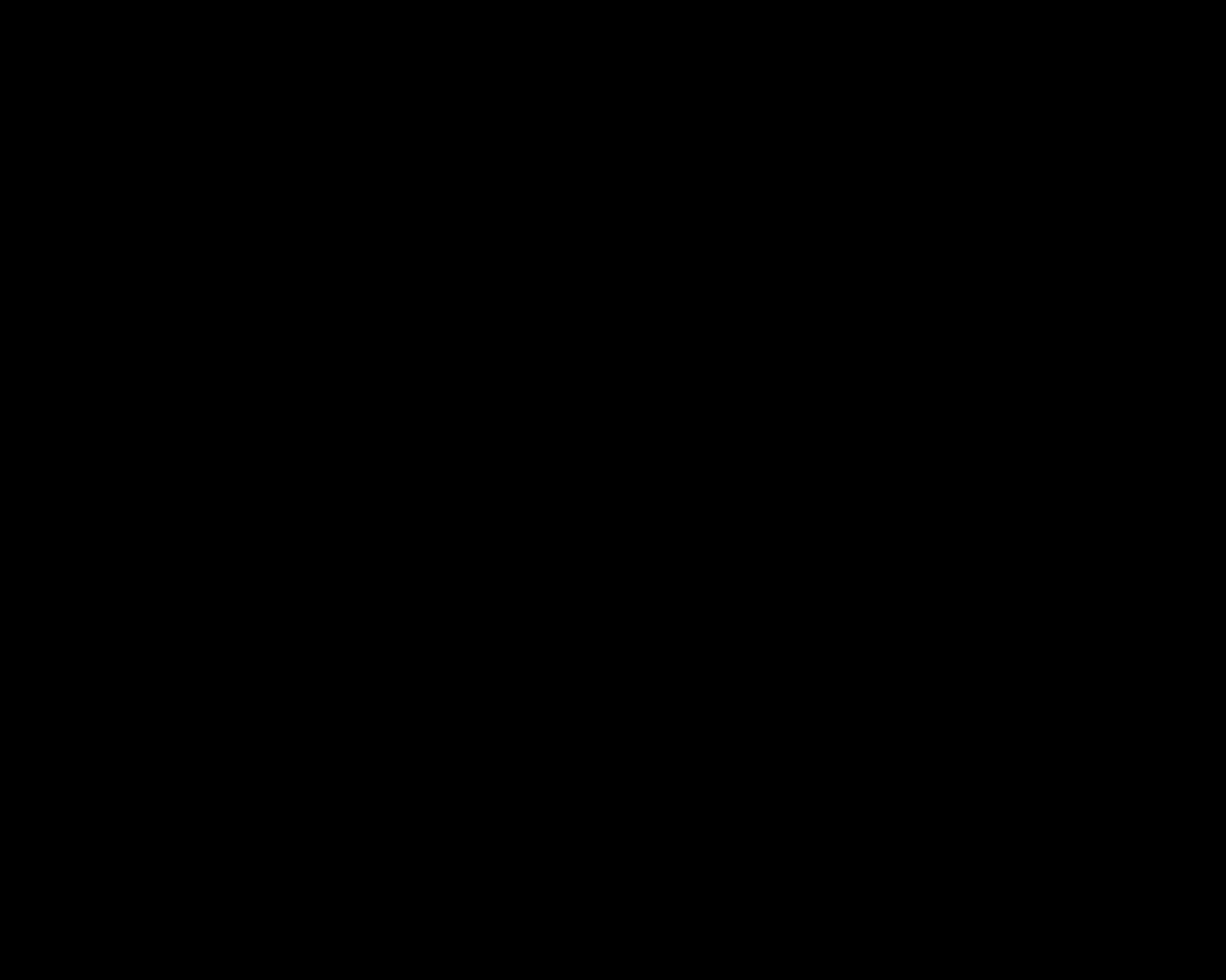

Supplement: S1 File — (ZIP) [file pone.0272206.s003.zip › new/miR297/E - 17(fld 2 wv D360_40x - HQ460_40m).tif]

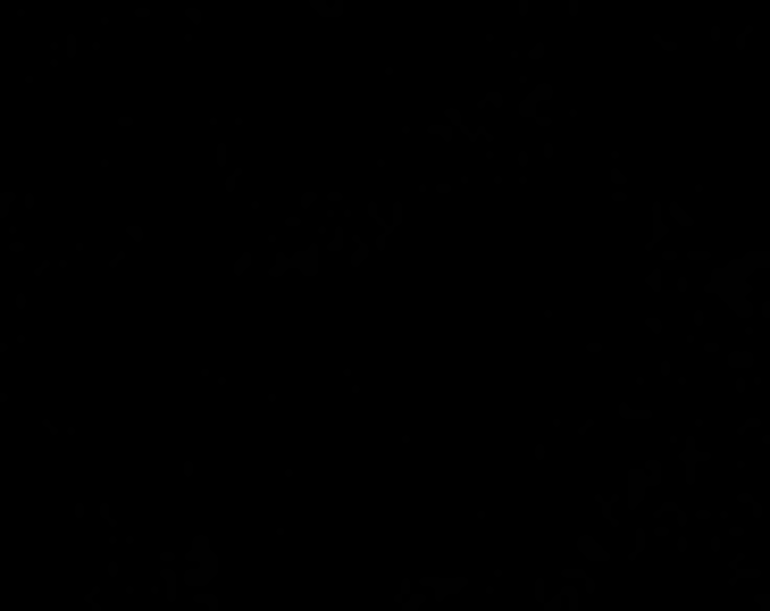

Supplement: S1 File — (ZIP) [file pone.0272206.s003.zip › new/miR297/E - 17(fld 2 wv D360_40x - HQ460_40m)_thumb.tif]

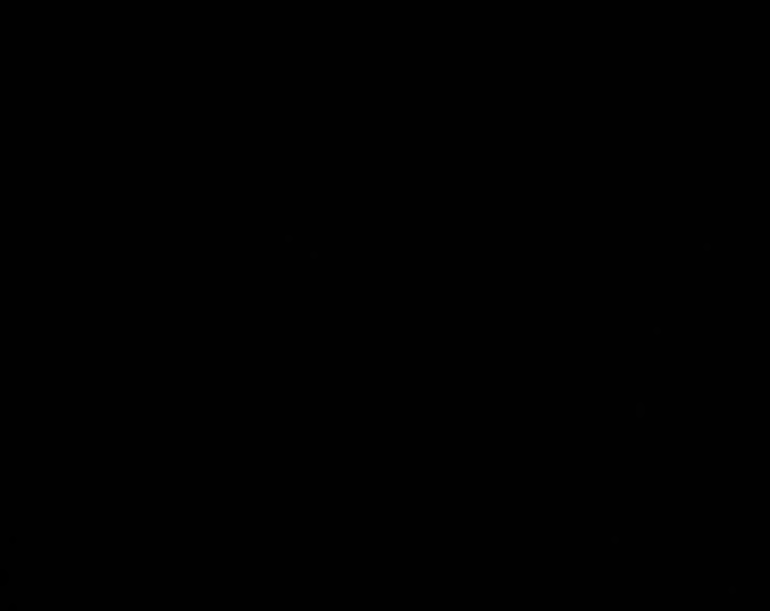

Supplement: S1 File — (ZIP) [file pone.0272206.s003.zip › new/miR297/E - 17(fld 2 wv S475_20x - HQ535_50m)_thumb.tif]

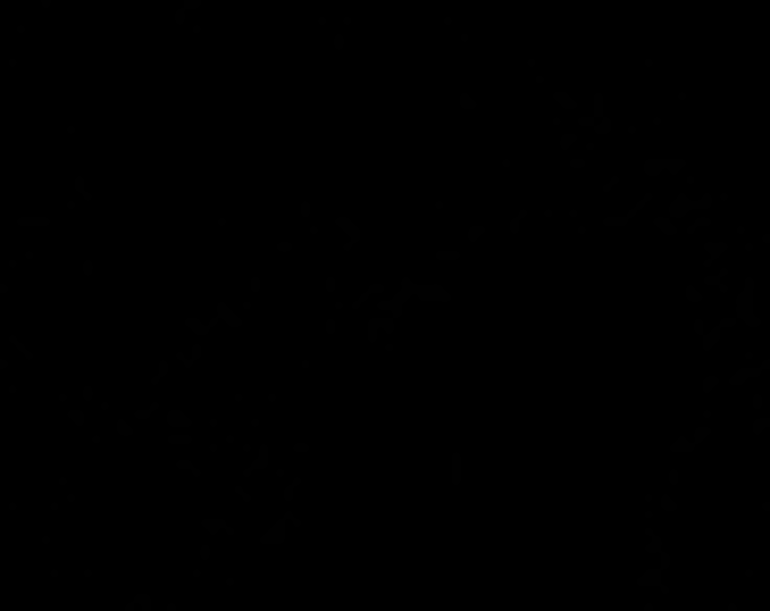

Supplement: S1 File — (ZIP) [file pone.0272206.s003.zip › new/miR297/E - 17(fld 3 wv D360_40x - HQ460_40m)_thumb.tif]

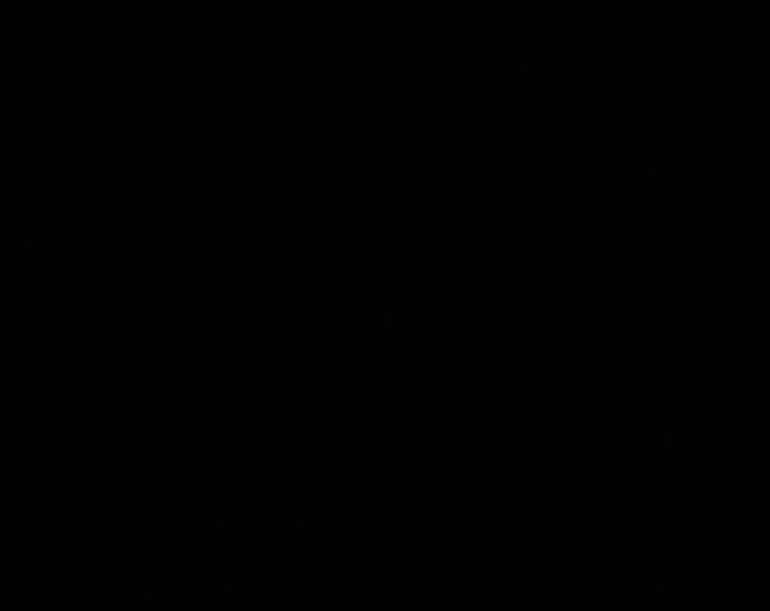

Supplement: S1 File — (ZIP) [file pone.0272206.s003.zip › new/miR297/E - 17(fld 3 wv S475_20x - HQ535_50m)_thumb.tif]

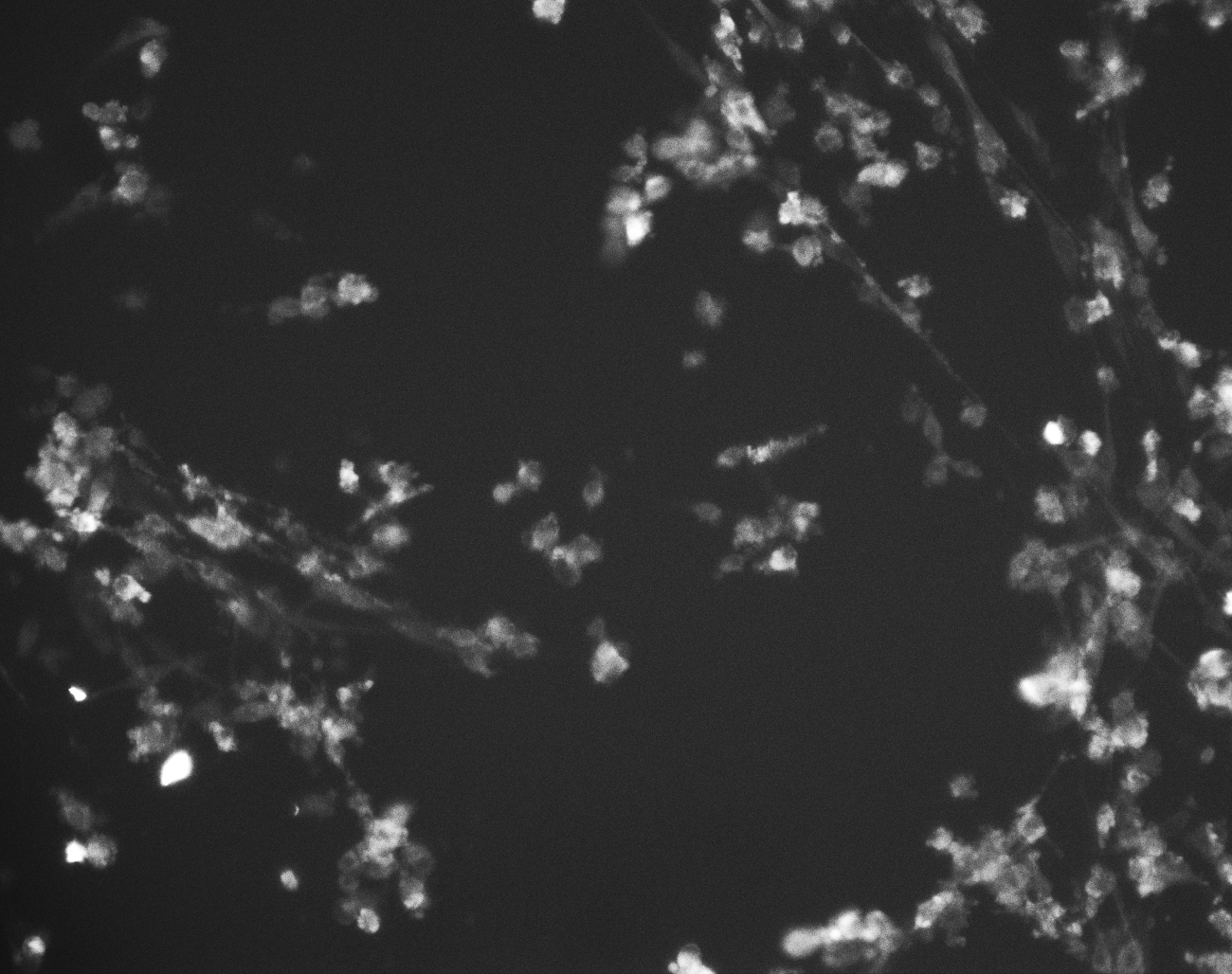

Supplement: S1 File — (ZIP) [file pone.0272206.s003.zip › new/miR297/p16.tif]

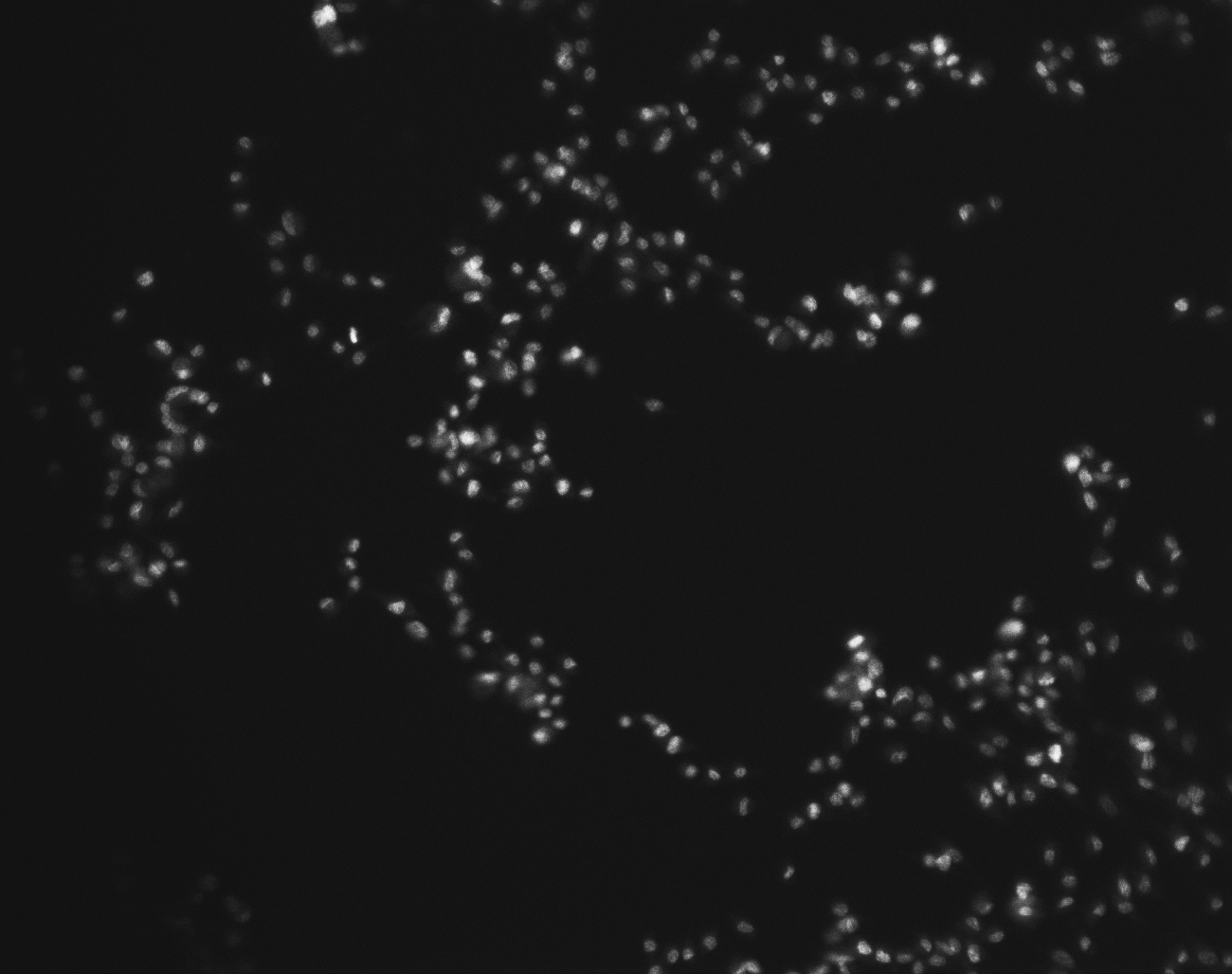

Supplement: S1 File — (ZIP) [file pone.0272206.s003.zip › new/mir30d/dapi.tif]

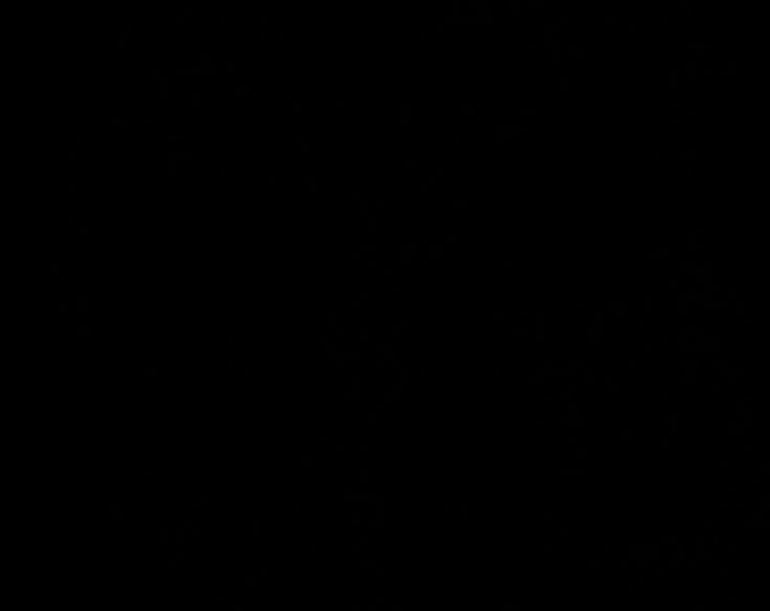

Supplement: S1 File — (ZIP) [file pone.0272206.s003.zip › new/mir30d/M - 4(fld 1 wv D360_40x - HQ460_40m)_thumb.tif]

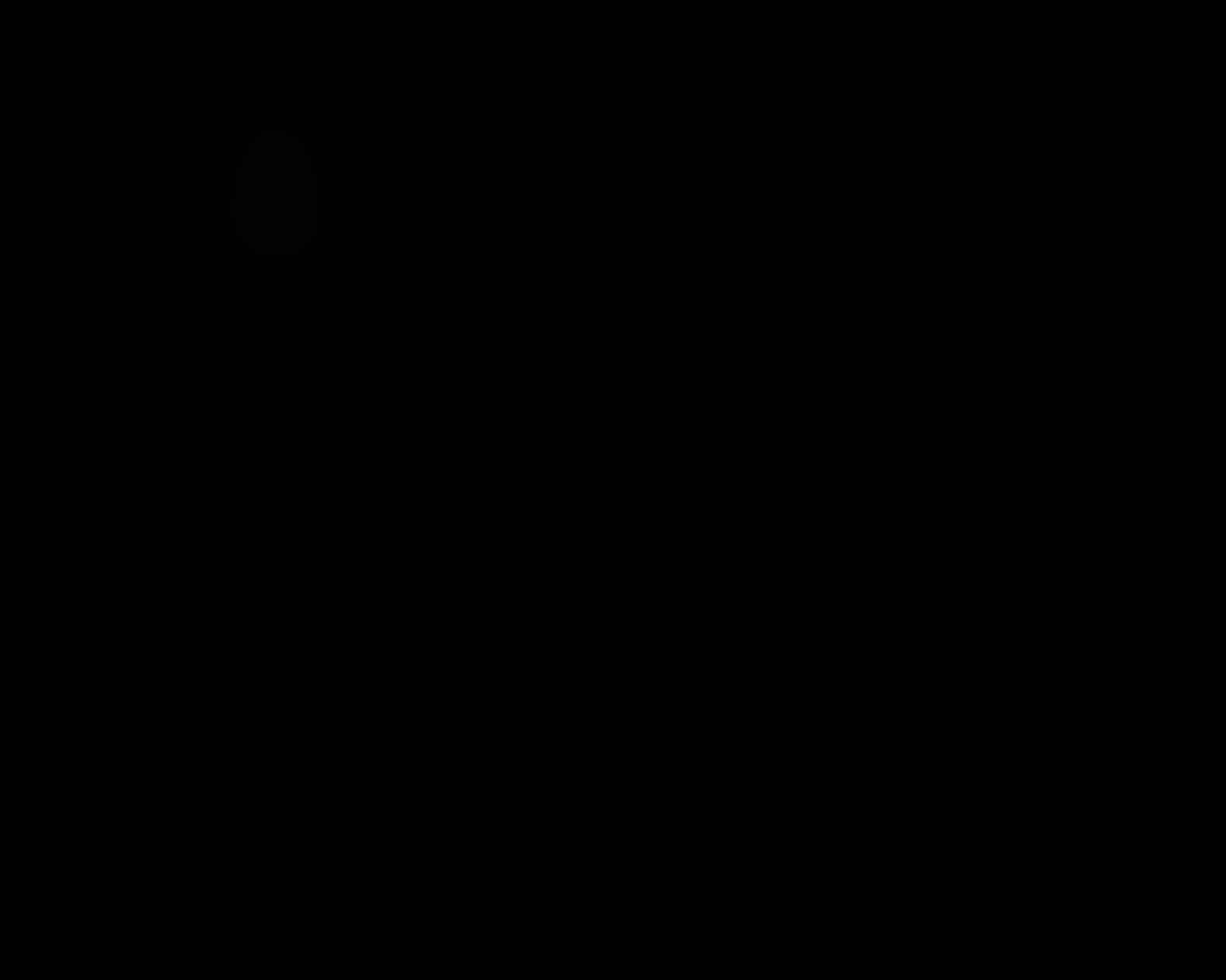

Supplement: S1 File — (ZIP) [file pone.0272206.s003.zip › new/mir30d/M - 4(fld 1 wv S475_20x - HQ535_50m).tif]

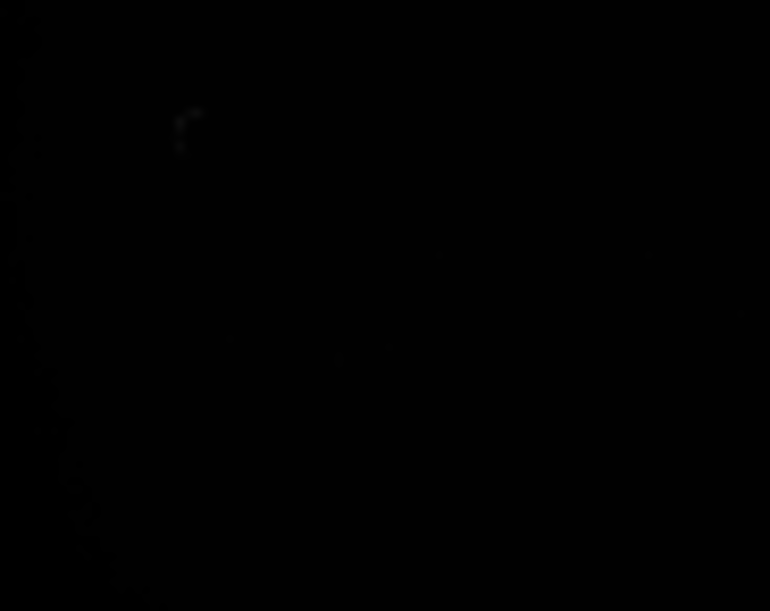

Supplement: S1 File — (ZIP) [file pone.0272206.s003.zip › new/mir30d/M - 4(fld 1 wv S475_20x - HQ535_50m)_thumb.tif]

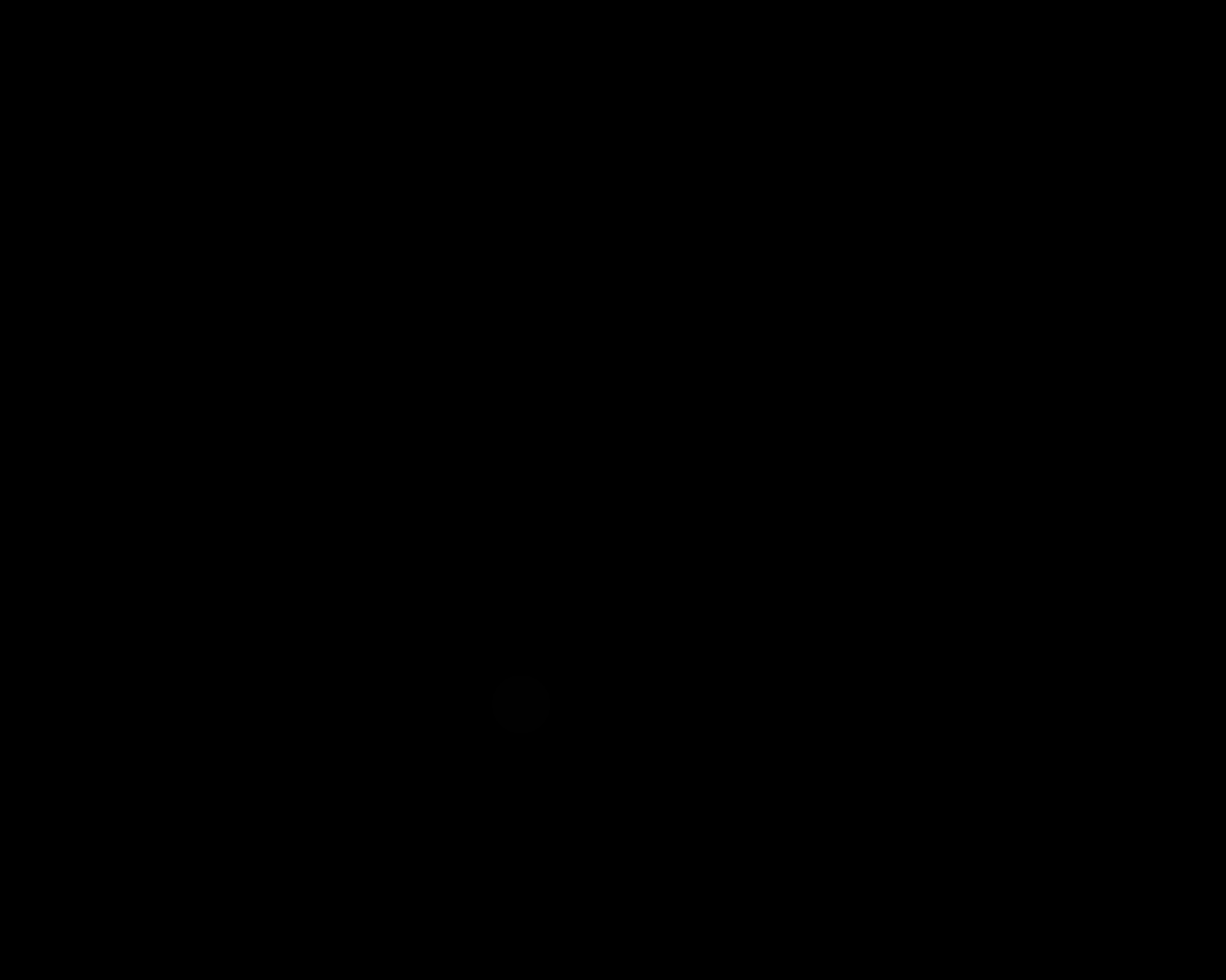

Supplement: S1 File — (ZIP) [file pone.0272206.s003.zip › new/mir30d/M - 4(fld 2 wv D360_40x - HQ460_40m).tif]

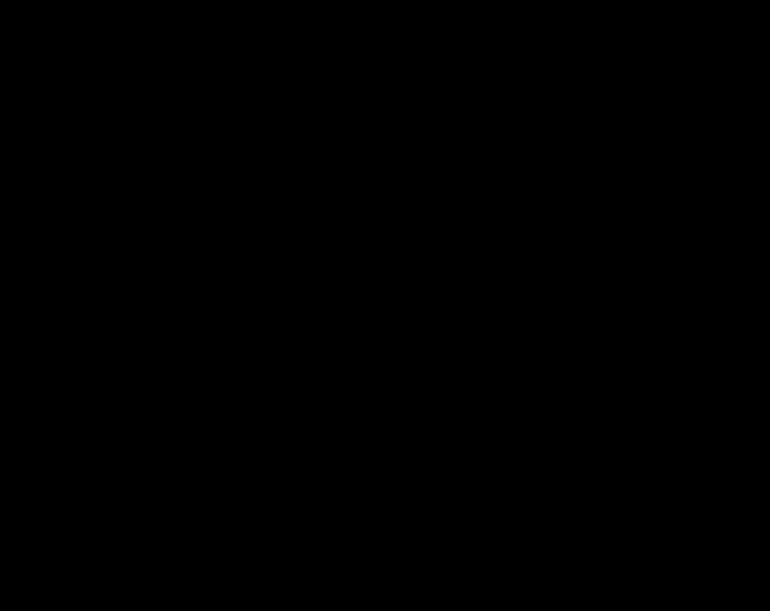

Supplement: S1 File — (ZIP) [file pone.0272206.s003.zip › new/mir30d/M - 4(fld 2 wv D360_40x - HQ460_40m)_thumb.tif]

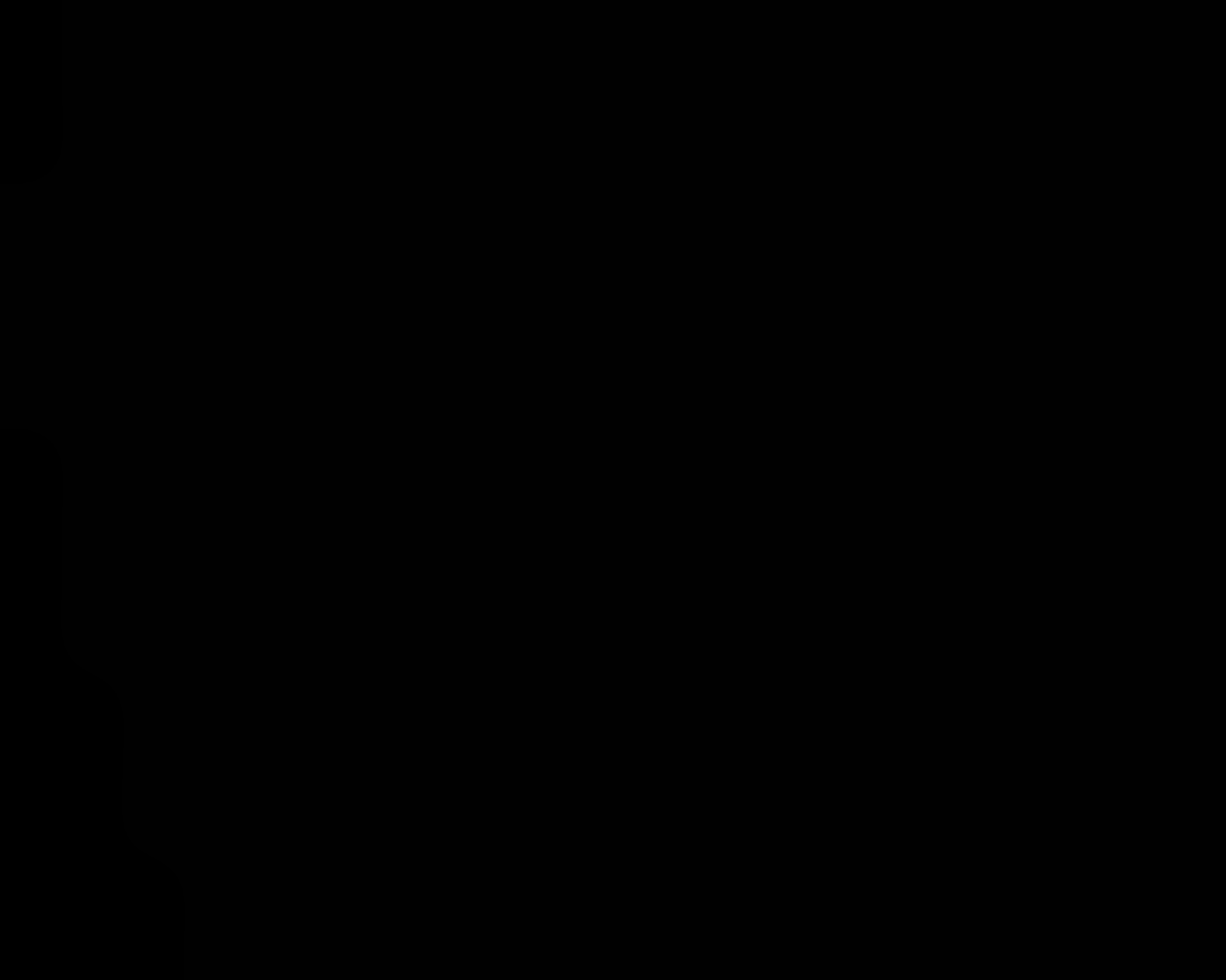

Supplement: S1 File — (ZIP) [file pone.0272206.s003.zip › new/mir30d/M - 4(fld 2 wv S475_20x - HQ535_50m).tif]

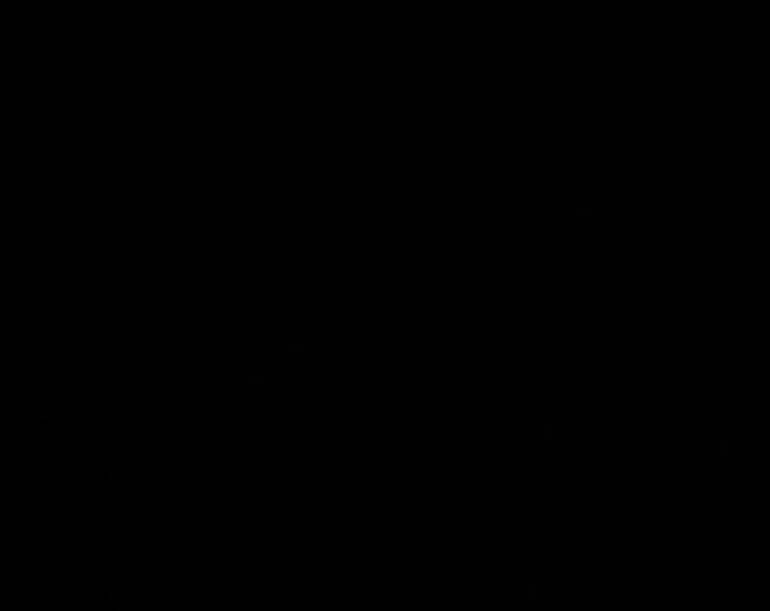

Supplement: S1 File — (ZIP) [file pone.0272206.s003.zip › new/mir30d/M - 4(fld 2 wv S475_20x - HQ535_50m)_thumb.tif]

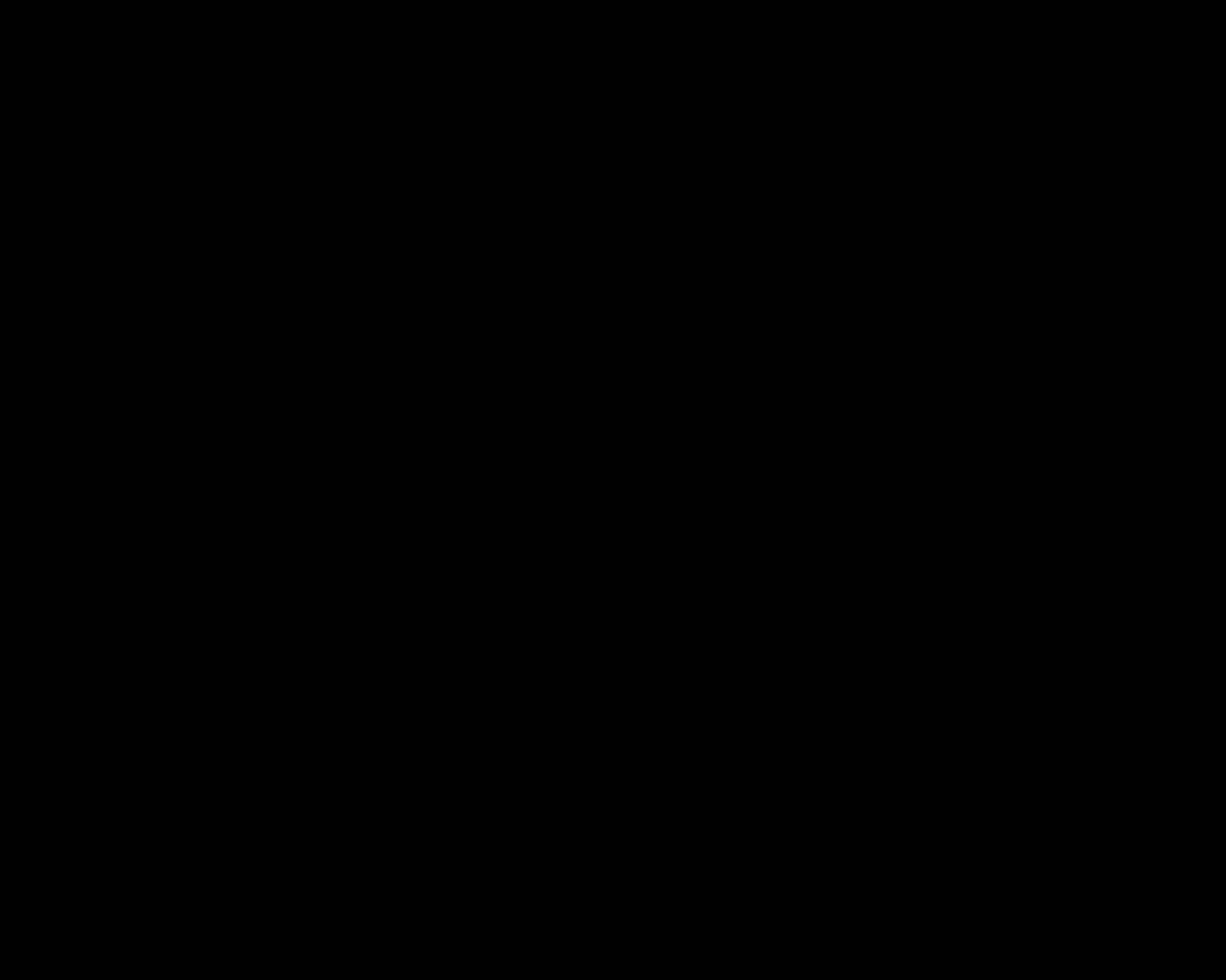

Supplement: S1 File — (ZIP) [file pone.0272206.s003.zip › new/mir30d/M - 4(fld 3 wv D360_40x - HQ460_40m).tif]

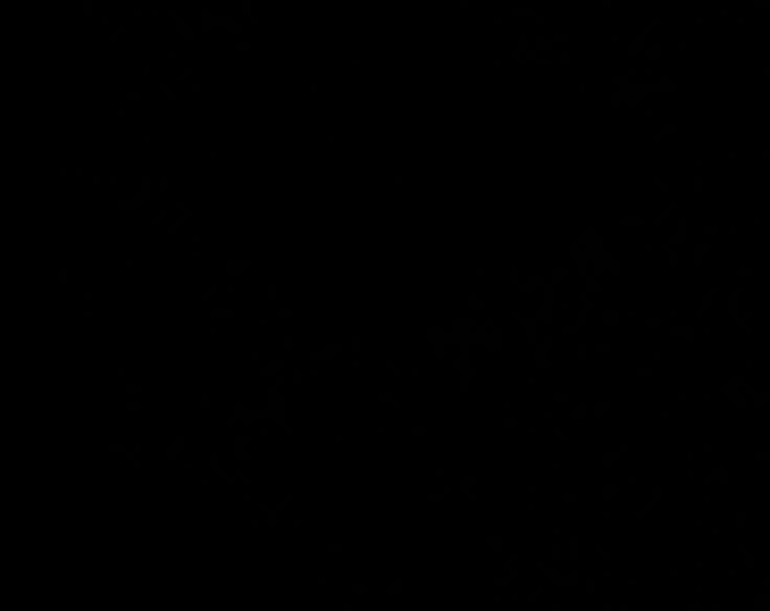

Supplement: S1 File — (ZIP) [file pone.0272206.s003.zip › new/mir30d/M - 4(fld 3 wv D360_40x - HQ460_40m)_thumb.tif]

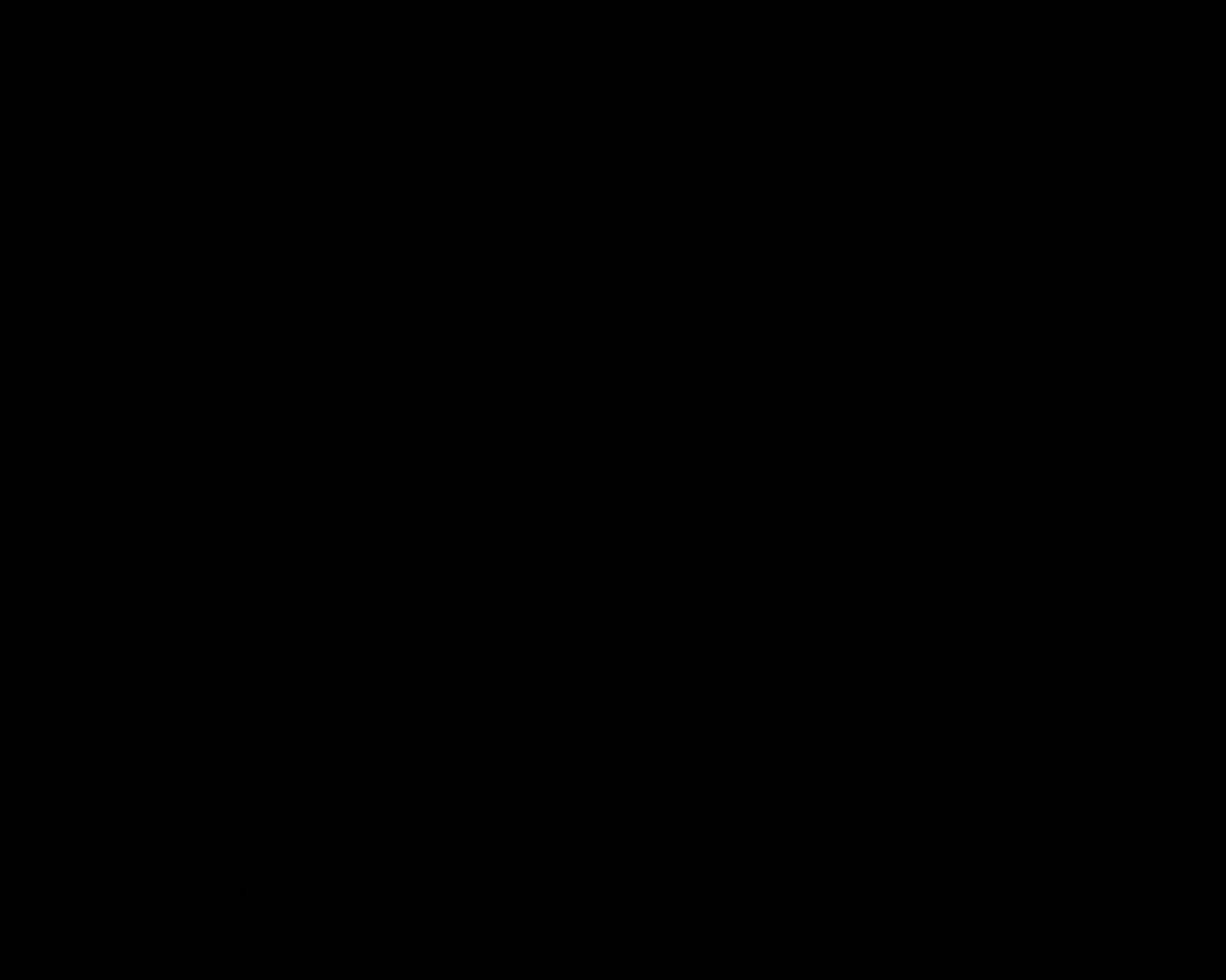

Supplement: S1 File — (ZIP) [file pone.0272206.s003.zip › new/mir30d/M - 4(fld 3 wv S475_20x - HQ535_50m).tif]

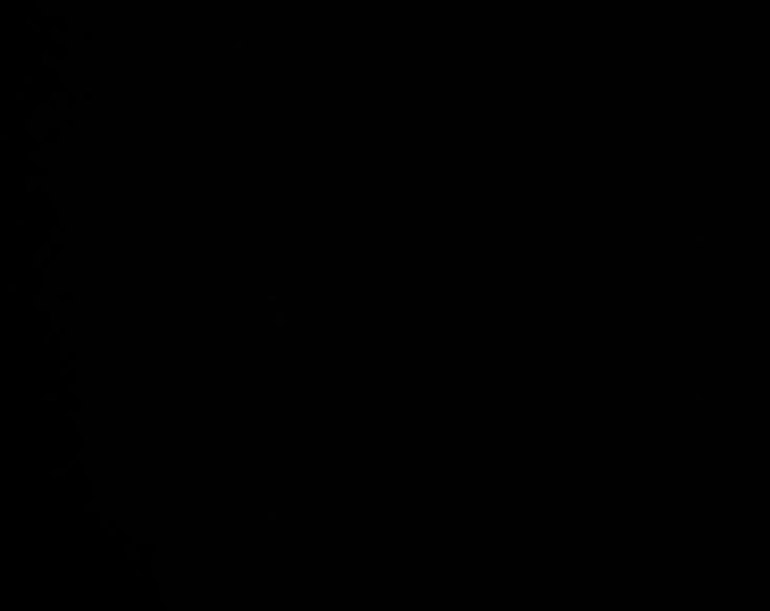

Supplement: S1 File — (ZIP) [file pone.0272206.s003.zip › new/mir30d/M - 4(fld 3 wv S475_20x - HQ535_50m)_thumb.tif]

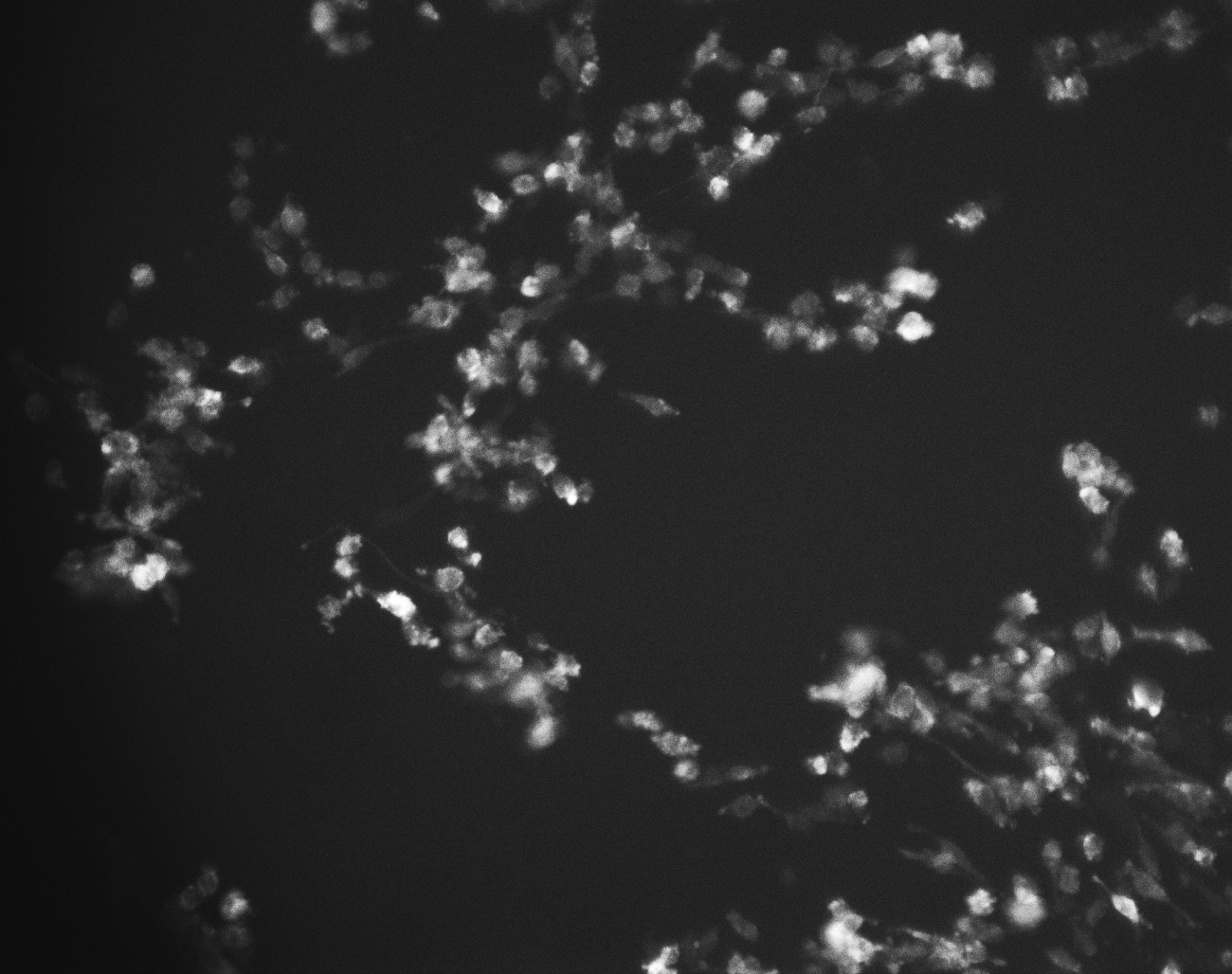

Supplement: S1 File — (ZIP) [file pone.0272206.s003.zip › new/mir30d/p16.tif]

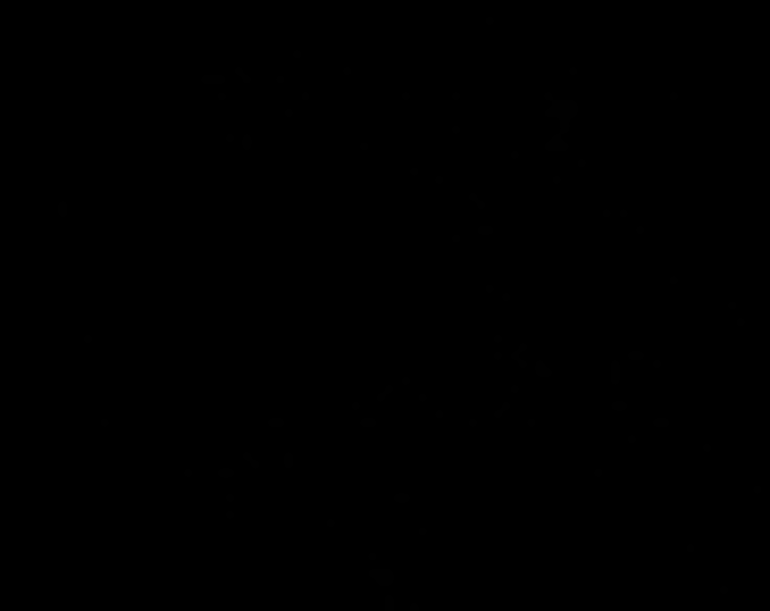

Supplement: S1 File — (ZIP) [file pone.0272206.s003.zip › new/mir34a/B - 10(fld 1 wv D360_40x - HQ460_40m)_thumb.tif]

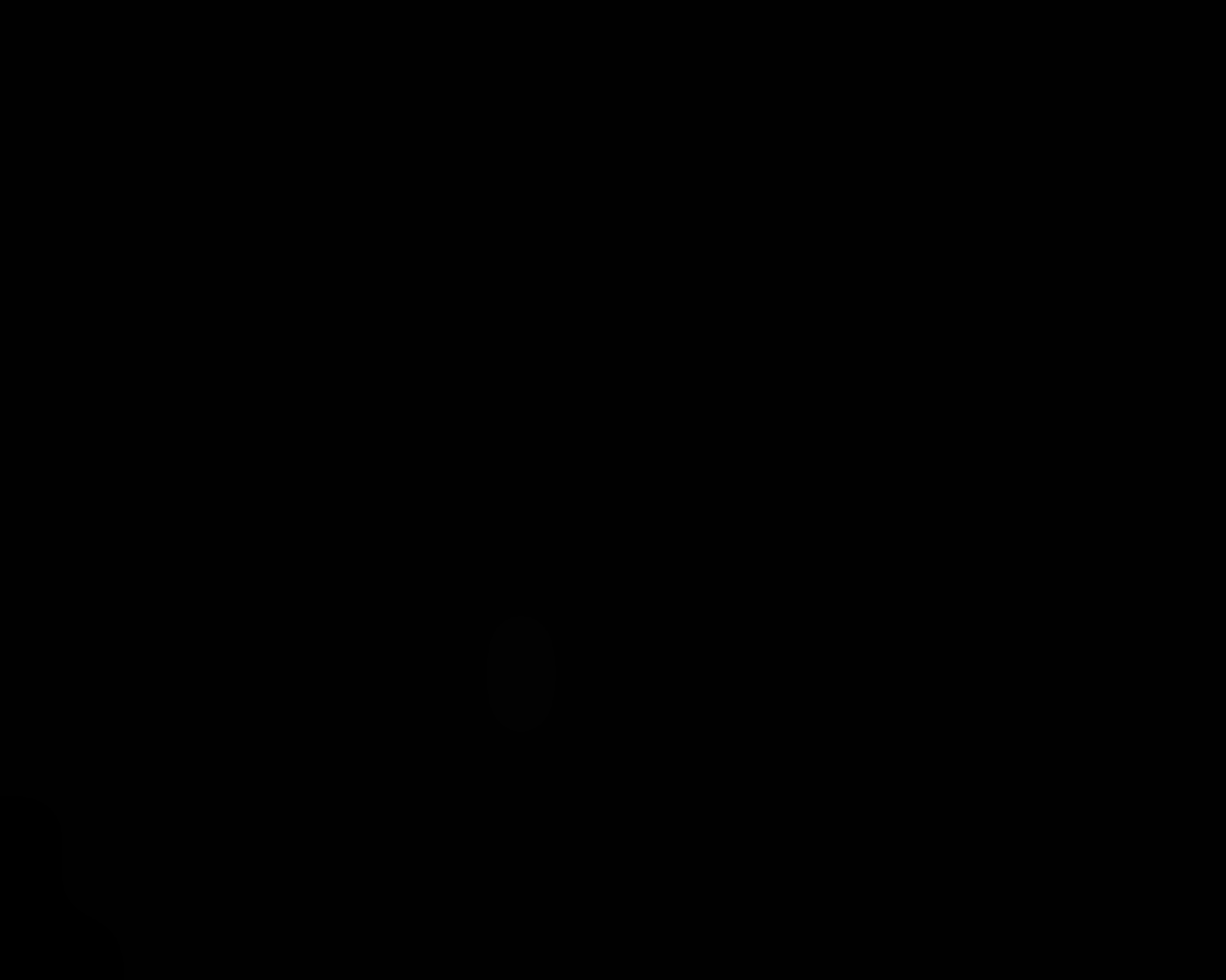

Supplement: S1 File — (ZIP) [file pone.0272206.s003.zip › new/mir34a/B - 10(fld 1 wv S475_20x - HQ535_50m).tif]

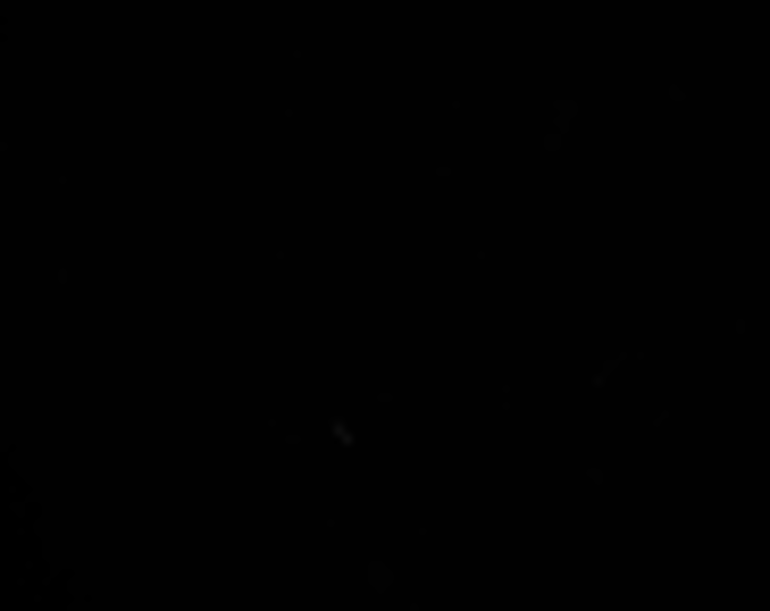

Supplement: S1 File — (ZIP) [file pone.0272206.s003.zip › new/mir34a/B - 10(fld 1 wv S475_20x - HQ535_50m)_thumb.tif]

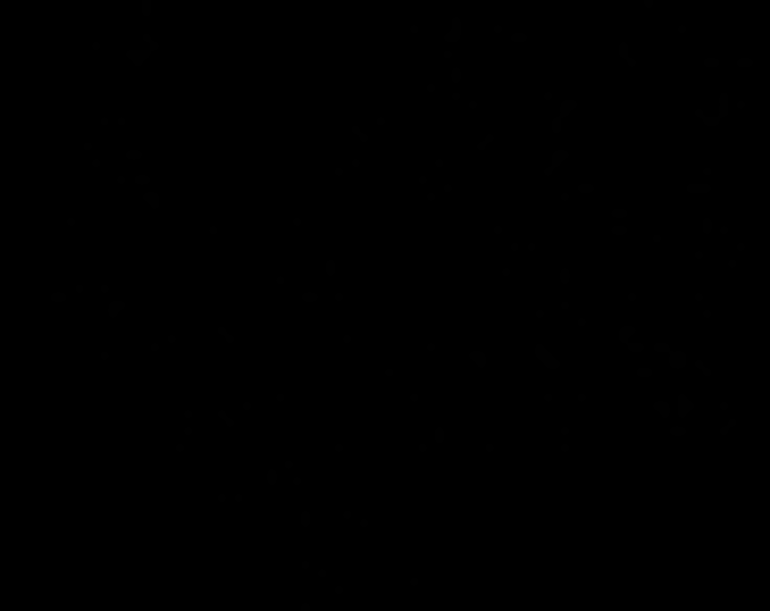

Supplement: S1 File — (ZIP) [file pone.0272206.s003.zip › new/mir34a/B - 10(fld 2 wv D360_40x - HQ460_40m)_thumb.tif]

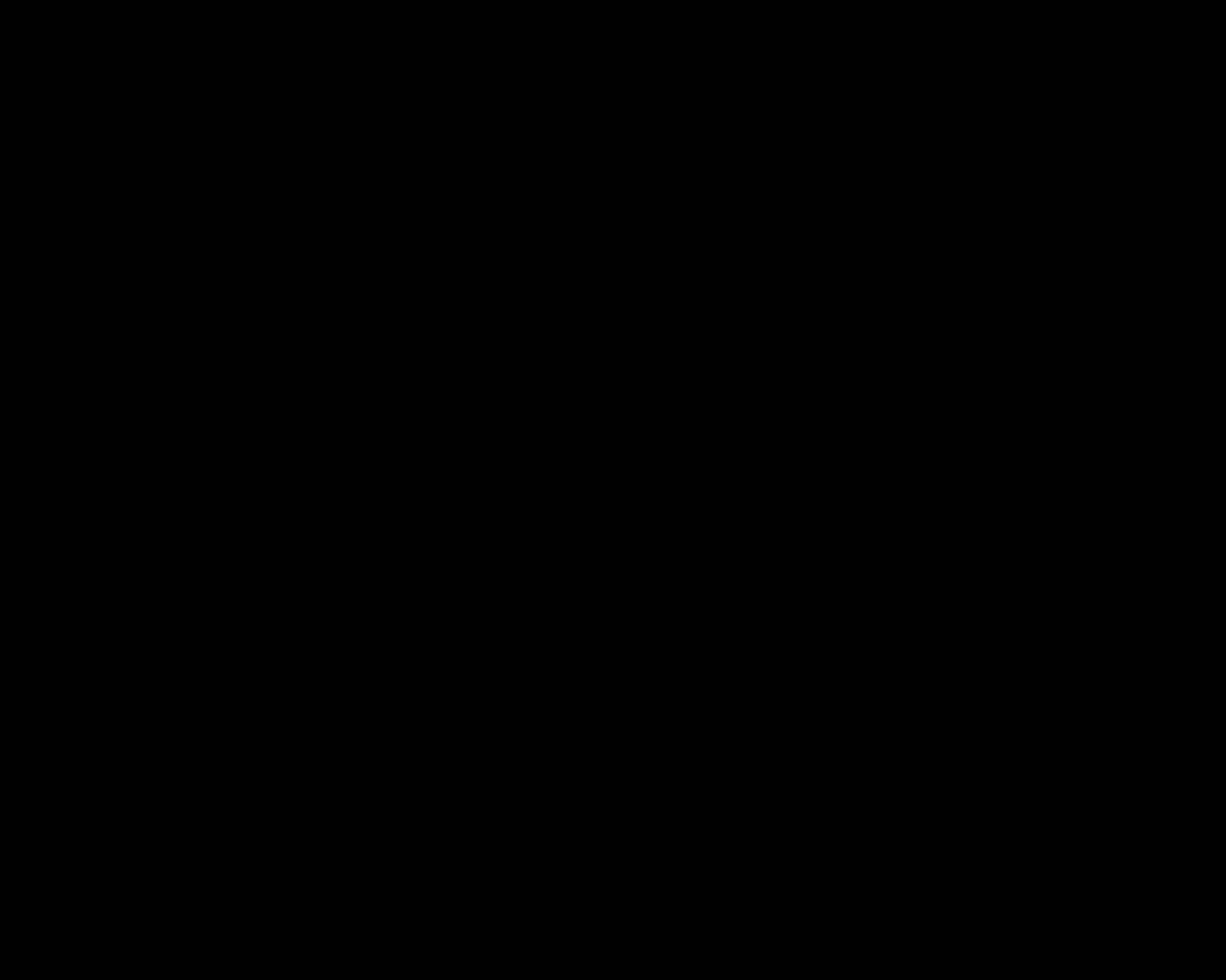

Supplement: S1 File — (ZIP) [file pone.0272206.s003.zip › new/mir34a/B - 10(fld 2 wv S475_20x - HQ535_50m).tif]

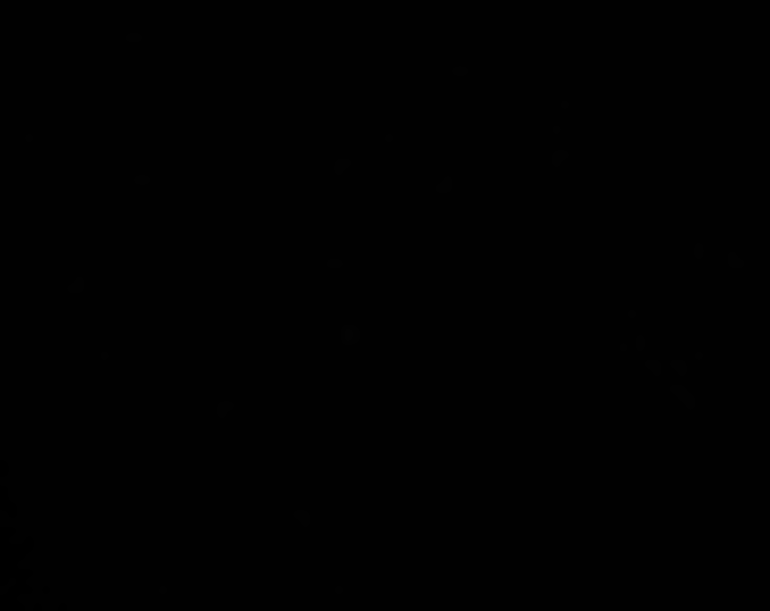

Supplement: S1 File — (ZIP) [file pone.0272206.s003.zip › new/mir34a/B - 10(fld 2 wv S475_20x - HQ535_50m)_thumb.tif]

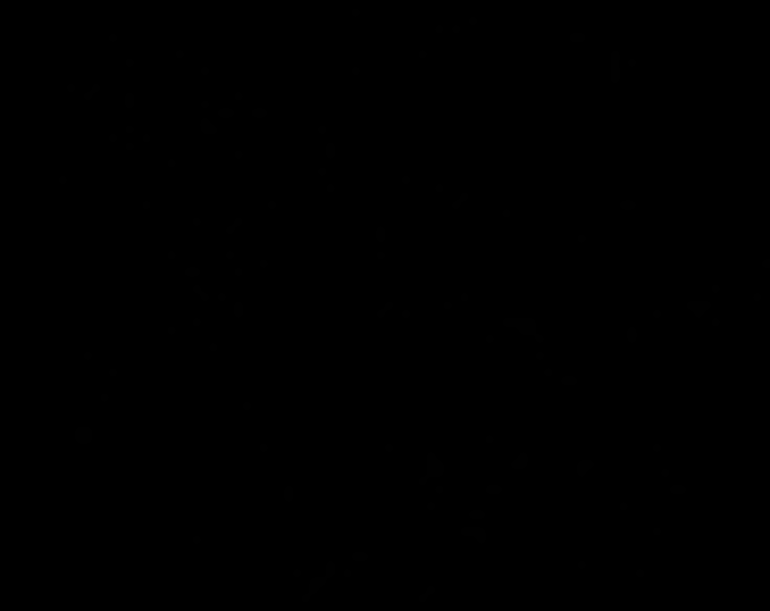

Supplement: S1 File — (ZIP) [file pone.0272206.s003.zip › new/mir34a/B - 10(fld 3 wv D360_40x - HQ460_40m)_thumb.tif]

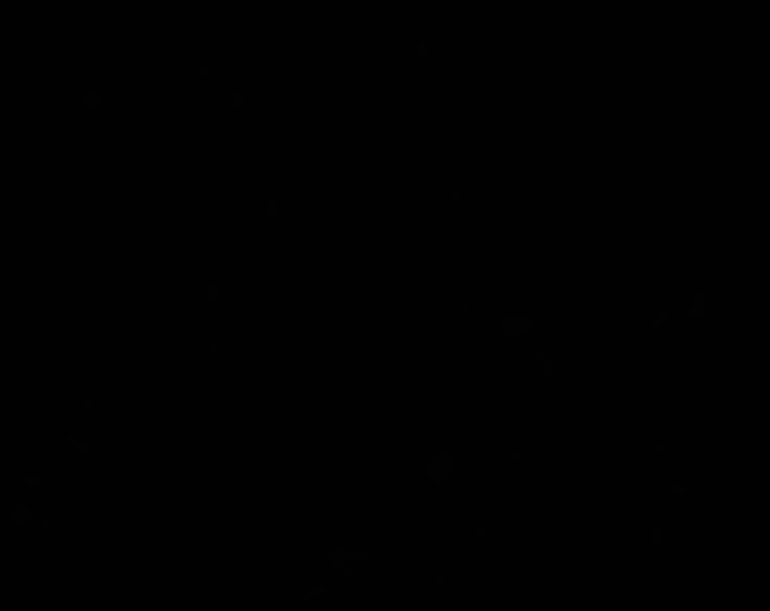

Supplement: S1 File — (ZIP) [file pone.0272206.s003.zip › new/mir34a/B - 10(fld 3 wv S475_20x - HQ535_50m)_thumb.tif]

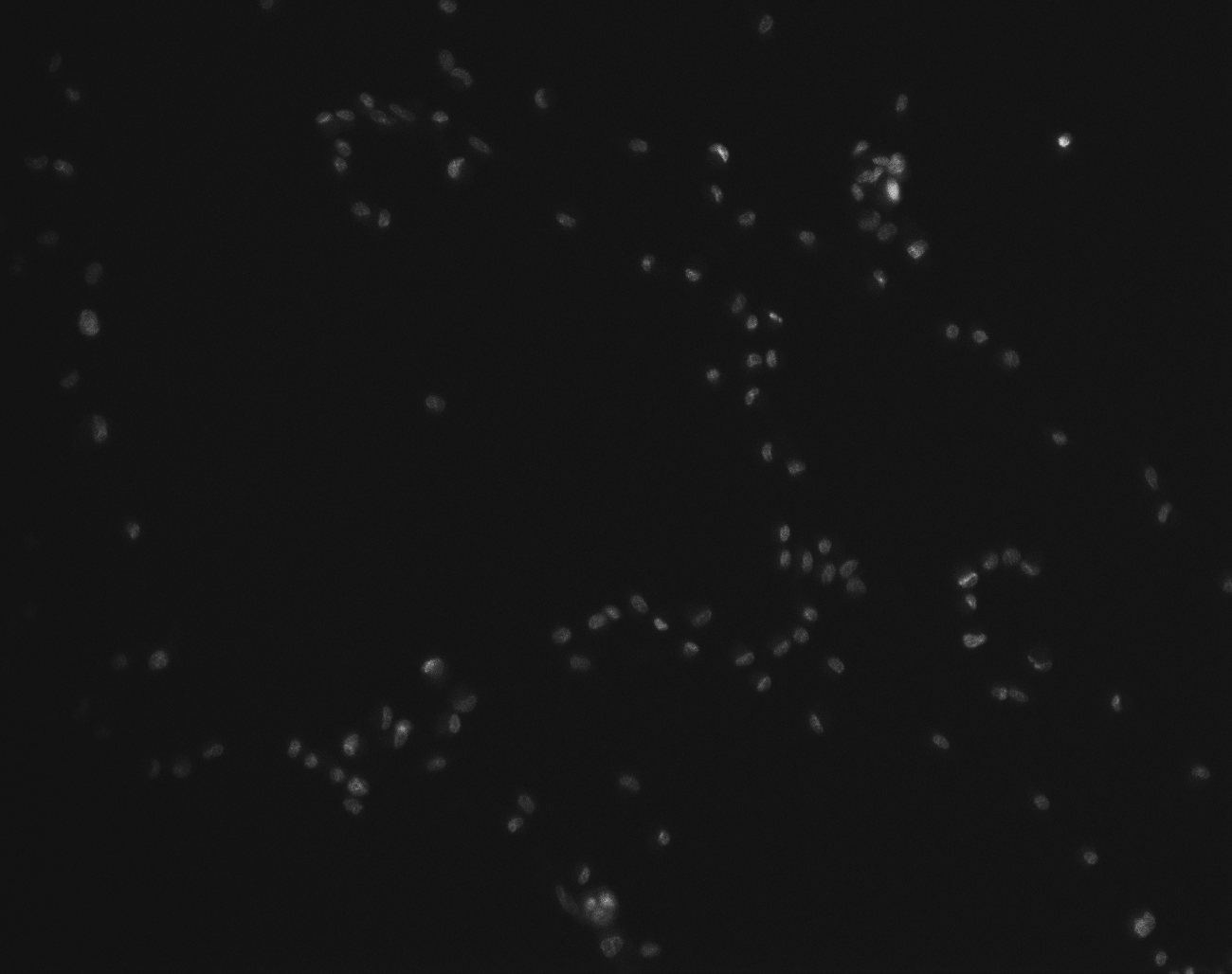

Supplement: S1 File — (ZIP) [file pone.0272206.s003.zip › new/mir34a/dapi.tif]

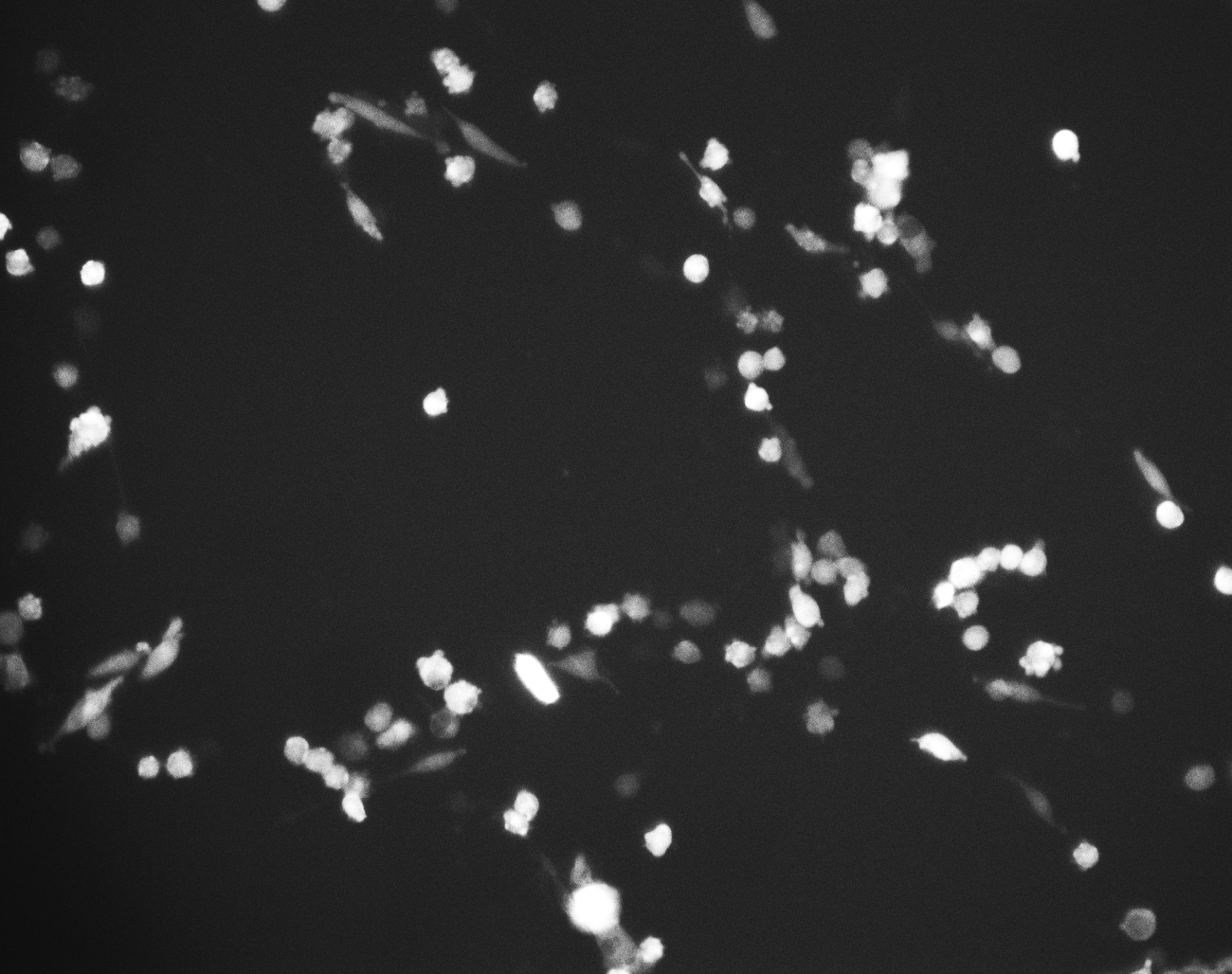

Supplement: S1 File — (ZIP) [file pone.0272206.s003.zip › new/mir34a/p16.tif]

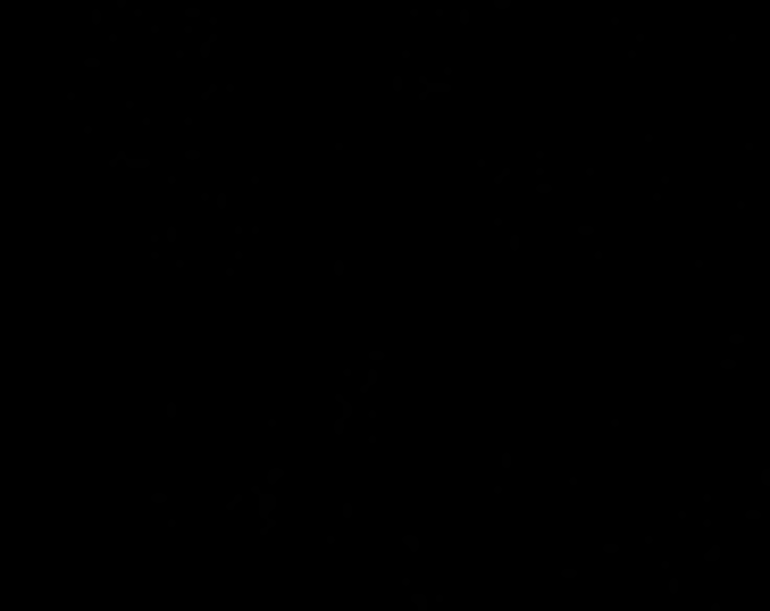

Supplement: S1 File — (ZIP) [file pone.0272206.s003.zip › new/miR34c/B - 14(fld 1 wv D360_40x - HQ460_40m)_thumb.tif]

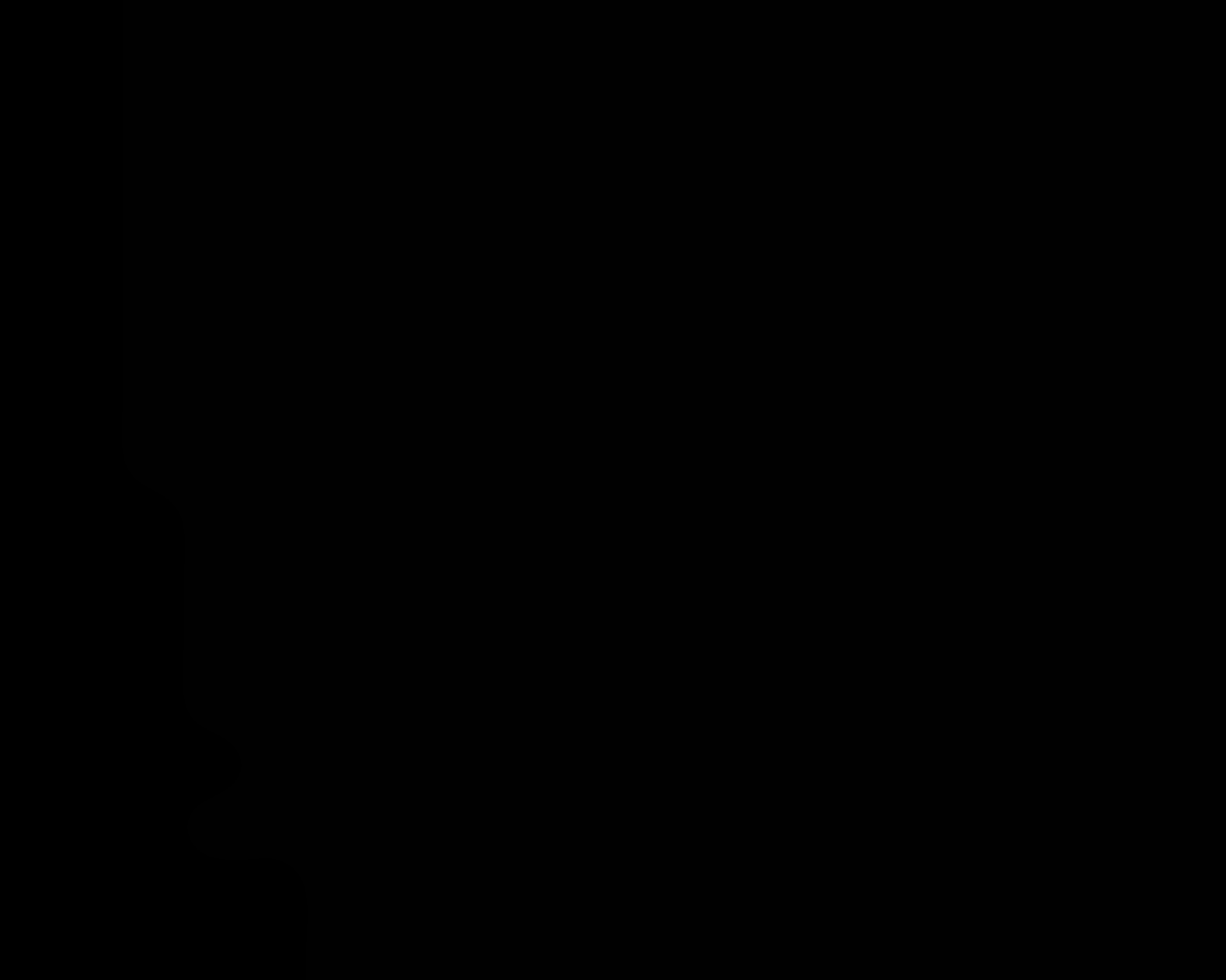

Supplement: S1 File — (ZIP) [file pone.0272206.s003.zip › new/miR34c/B - 14(fld 1 wv S475_20x - HQ535_50m).tif]

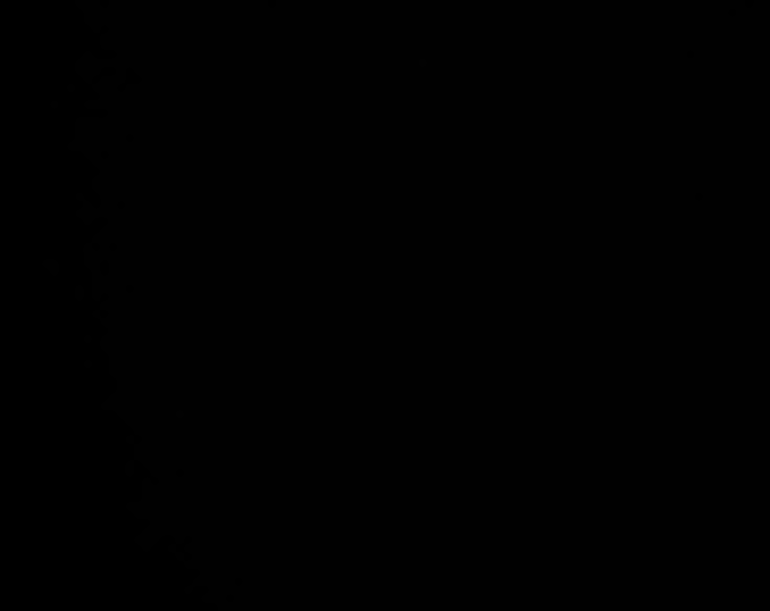

Supplement: S1 File — (ZIP) [file pone.0272206.s003.zip › new/miR34c/B - 14(fld 1 wv S475_20x - HQ535_50m)_thumb.tif]

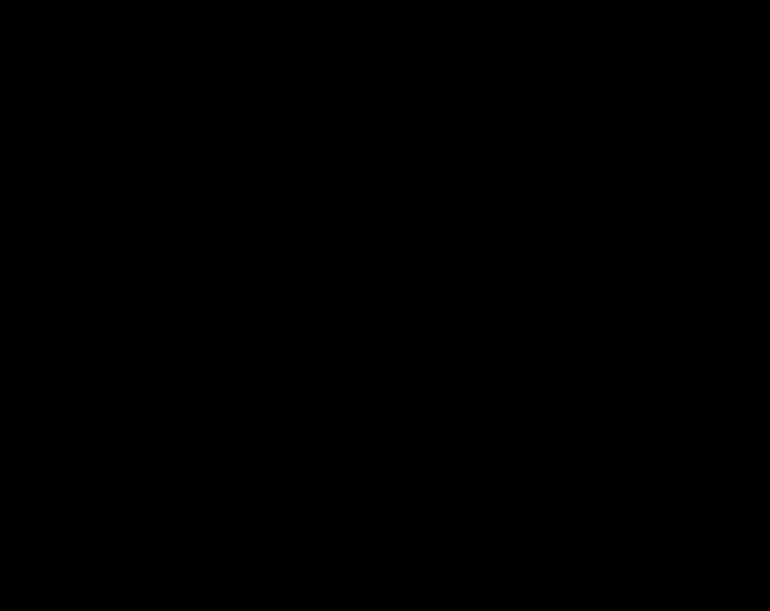

Supplement: S1 File — (ZIP) [file pone.0272206.s003.zip › new/miR34c/B - 14(fld 2 wv D360_40x - HQ460_40m)_thumb.tif]

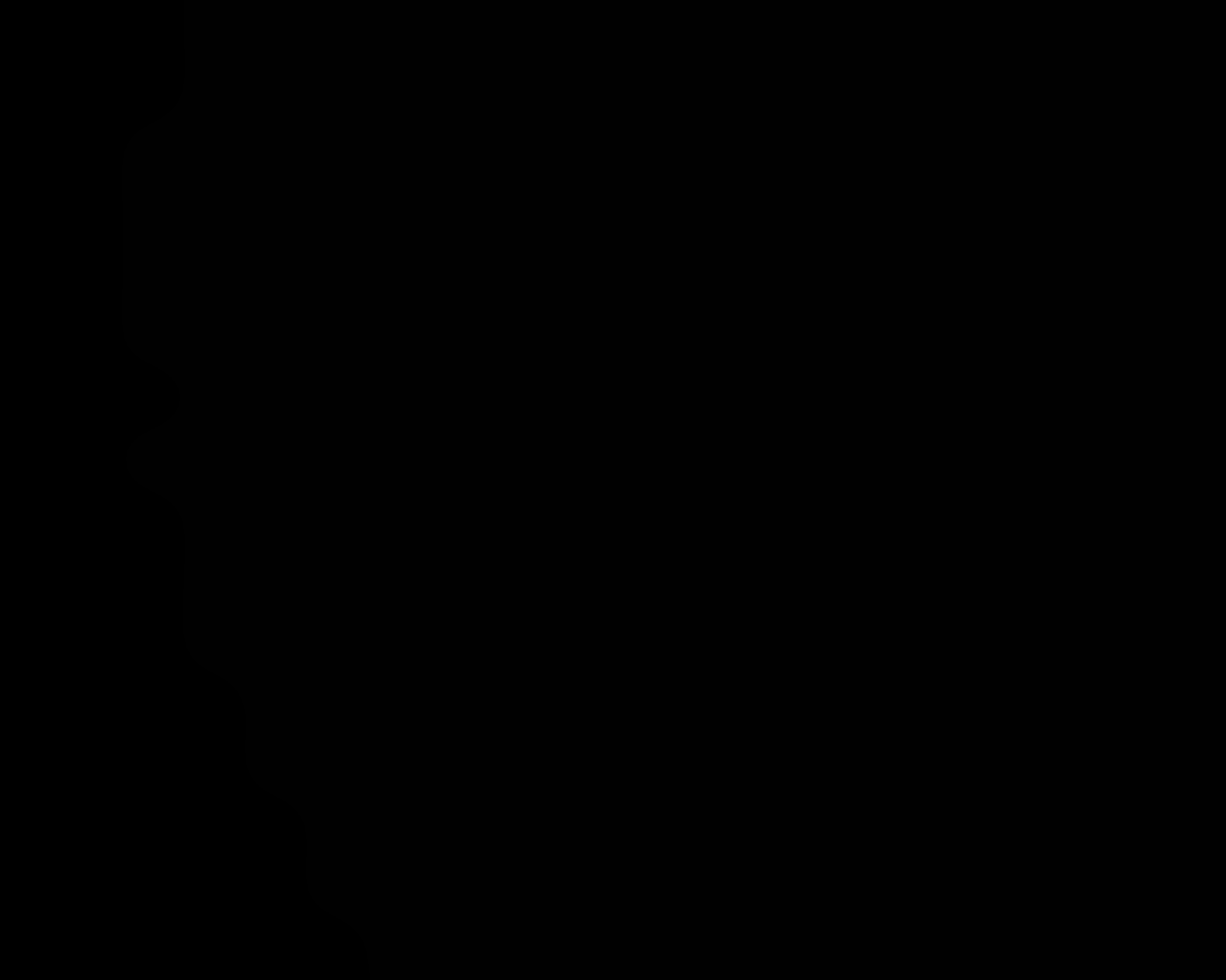

Supplement: S1 File — (ZIP) [file pone.0272206.s003.zip › new/miR34c/B - 14(fld 2 wv S475_20x - HQ535_50m).tif]

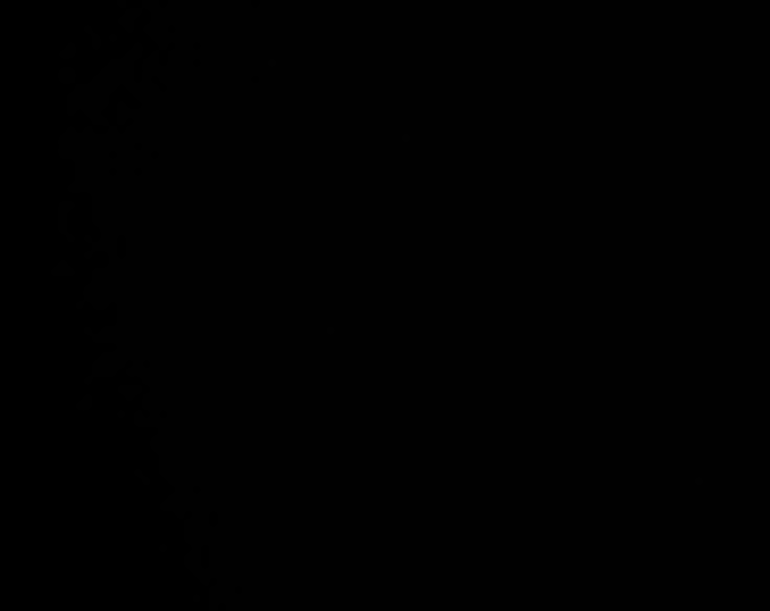

Supplement: S1 File — (ZIP) [file pone.0272206.s003.zip › new/miR34c/B - 14(fld 2 wv S475_20x - HQ535_50m)_thumb.tif]

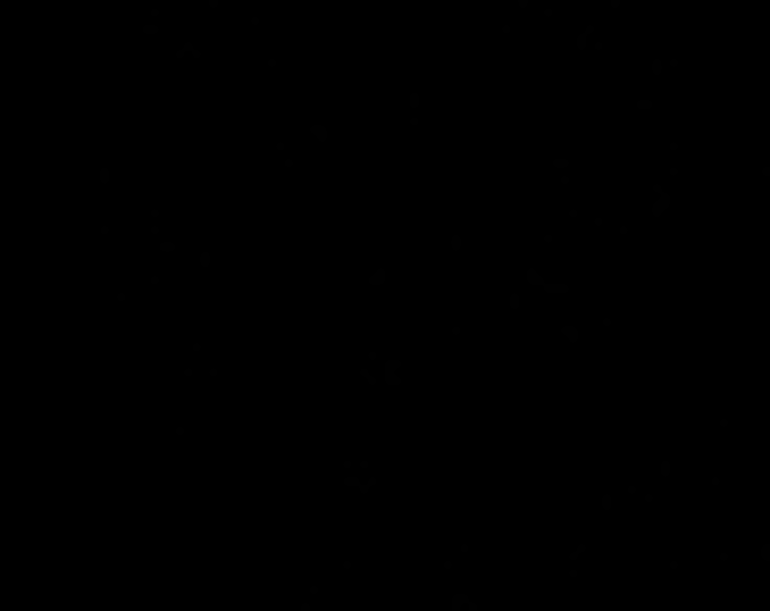

Supplement: S1 File — (ZIP) [file pone.0272206.s003.zip › new/miR34c/B - 14(fld 3 wv D360_40x - HQ460_40m)_thumb.tif]

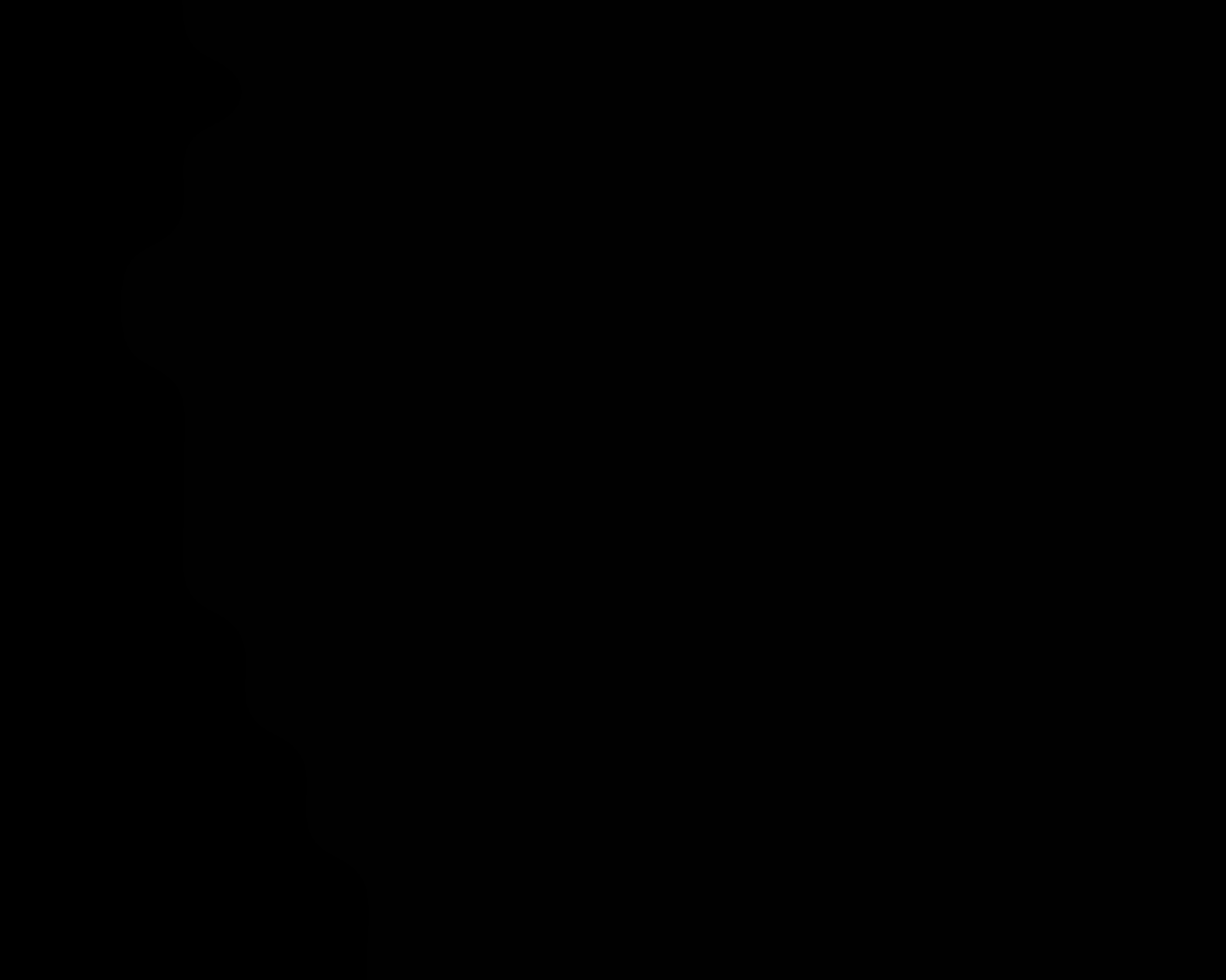

Supplement: S1 File — (ZIP) [file pone.0272206.s003.zip › new/miR34c/B - 14(fld 3 wv S475_20x - HQ535_50m).tif]

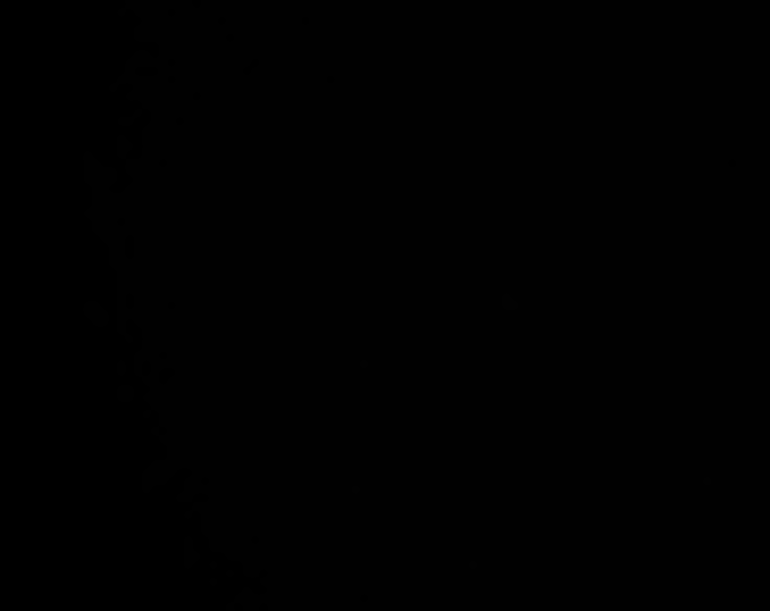

Supplement: S1 File — (ZIP) [file pone.0272206.s003.zip › new/miR34c/B - 14(fld 3 wv S475_20x - HQ535_50m)_thumb.tif]

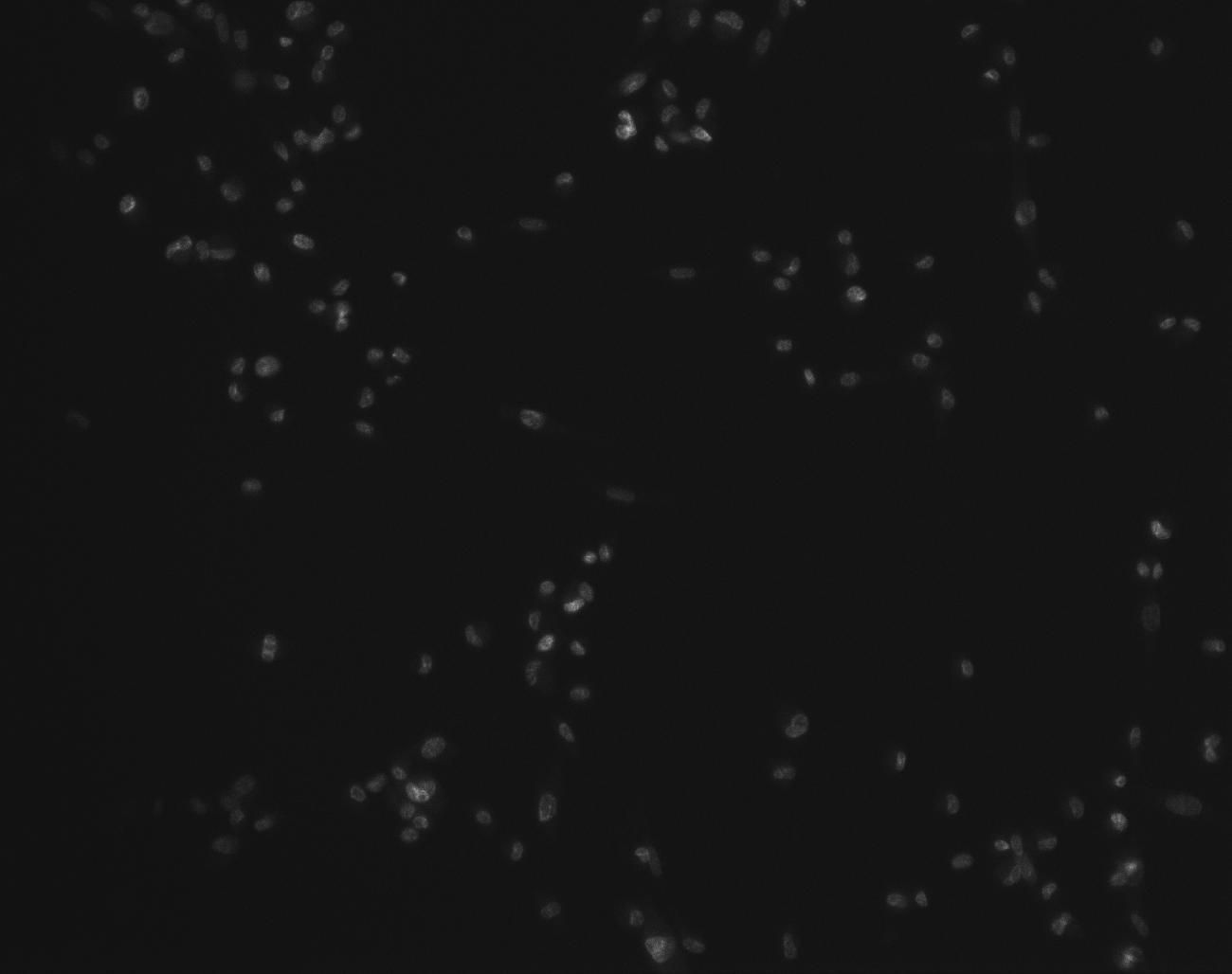

Supplement: S1 File — (ZIP) [file pone.0272206.s003.zip › new/miR34c/dapi.tif]

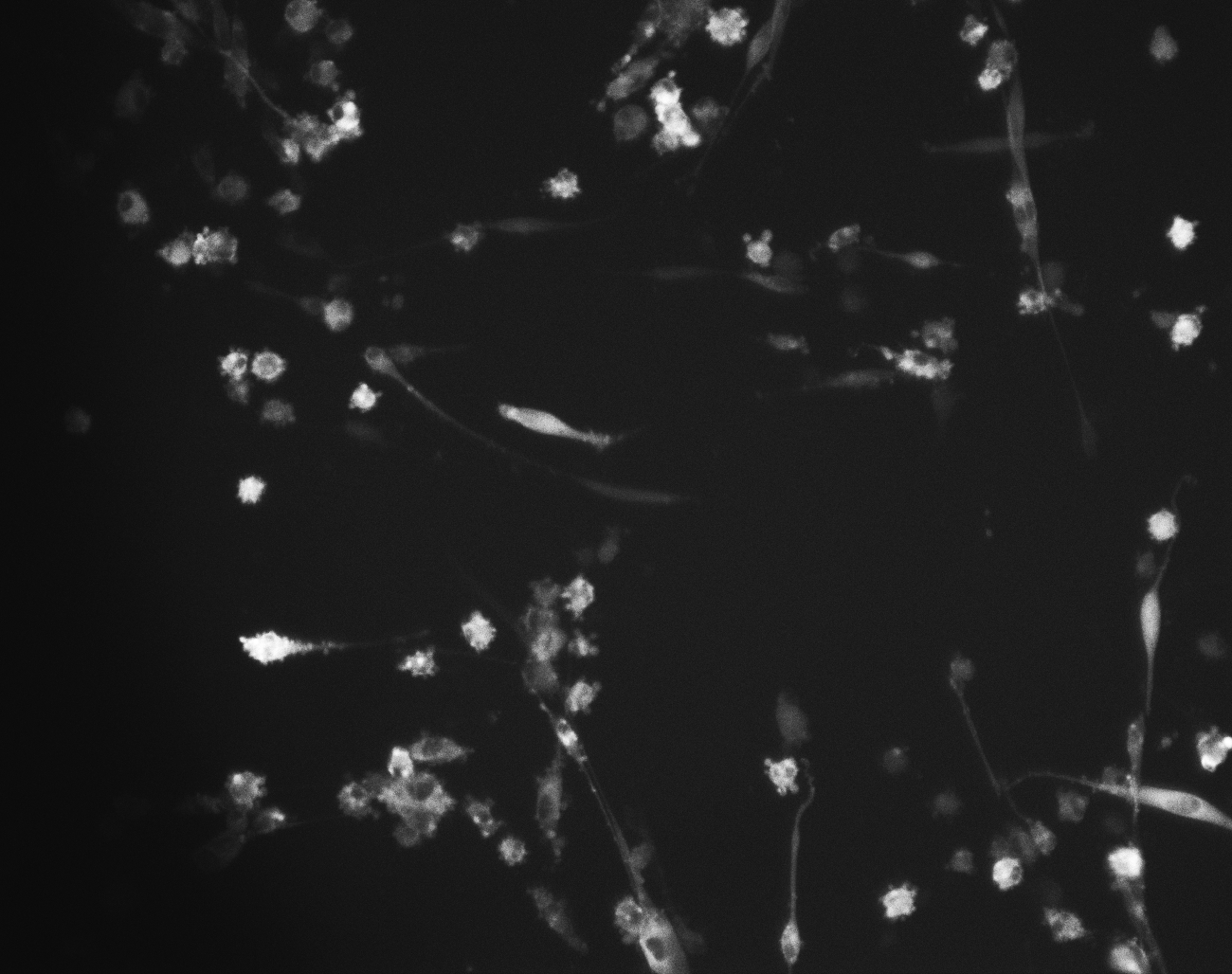

Supplement: S1 File — (ZIP) [file pone.0272206.s003.zip › new/miR34c/p16.tif]

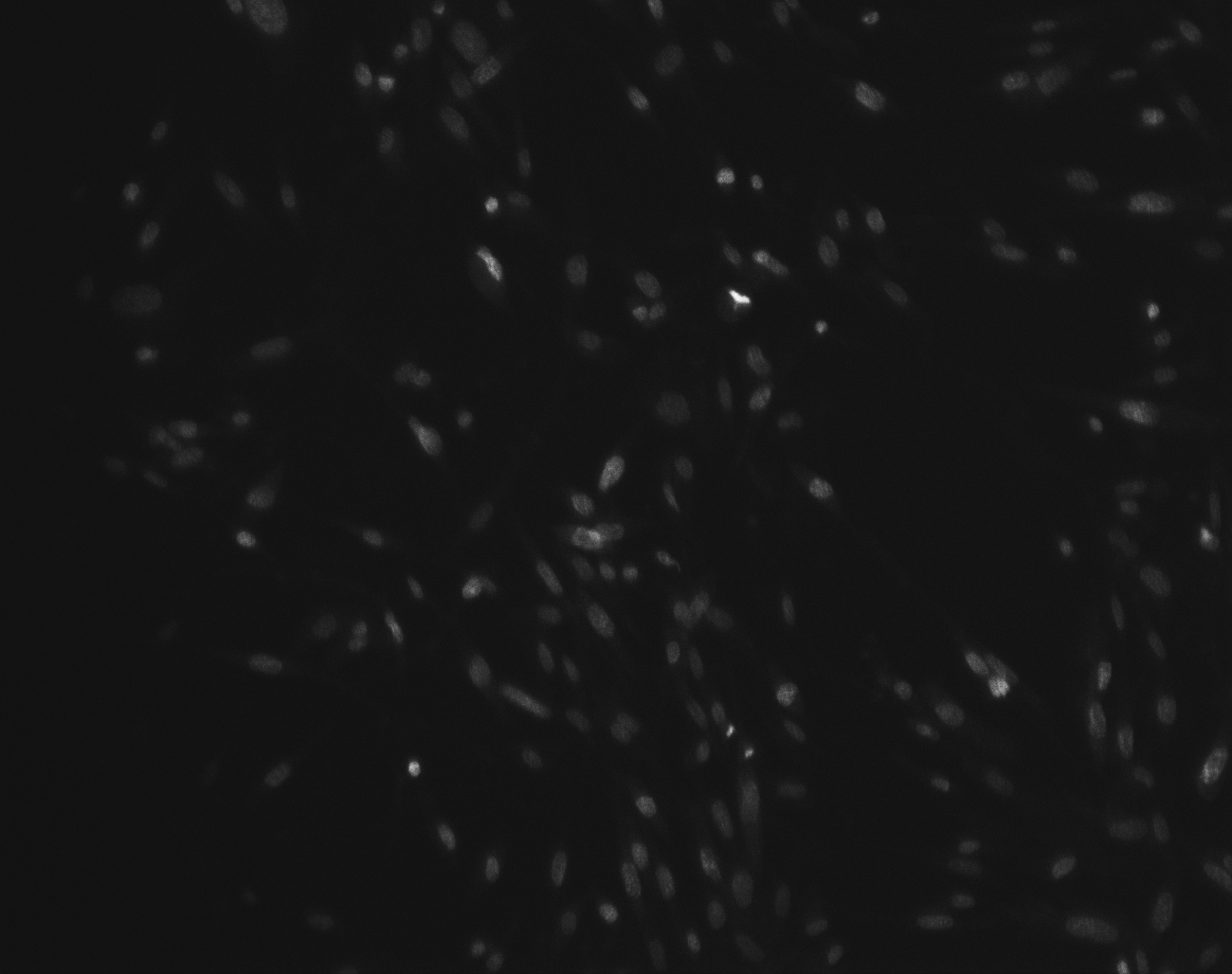

Supplement: S1 File — (ZIP) [file pone.0272206.s003.zip › new/mir378/dapi.tif]

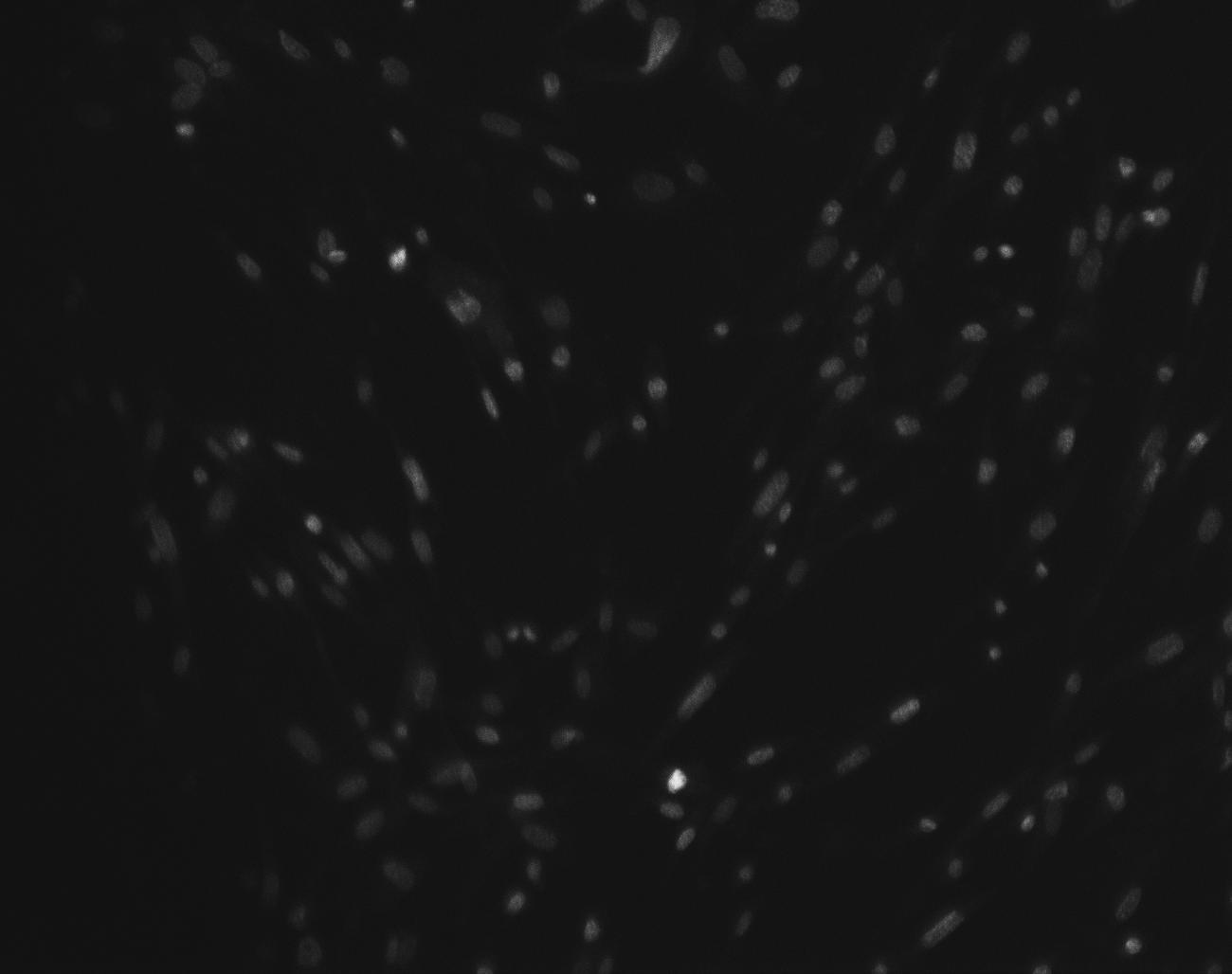

Supplement: S1 File — (ZIP) [file pone.0272206.s003.zip › new/mir378/dapi_2.tif]

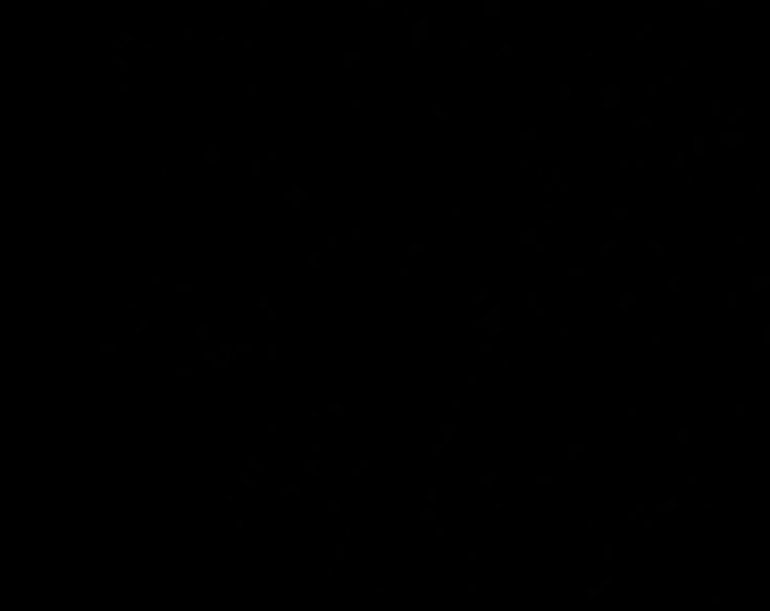

Supplement: S1 File — (ZIP) [file pone.0272206.s003.zip › new/mir378/E - 12(fld 1 wv D360_40x - HQ460_40m)_thumb.tif]

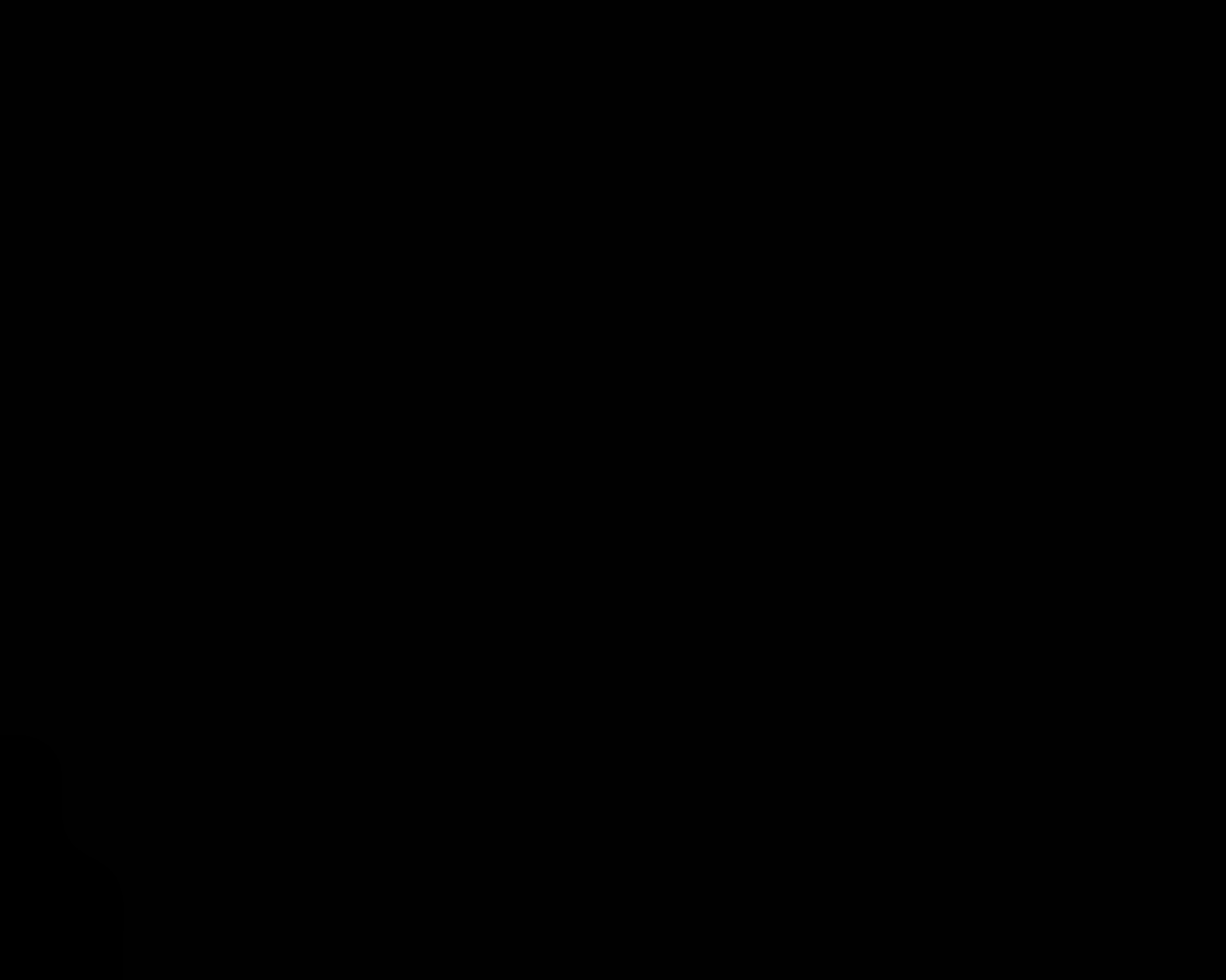

Supplement: S1 File — (ZIP) [file pone.0272206.s003.zip › new/mir378/E - 12(fld 1 wv S475_20x - HQ535_50m).tif]

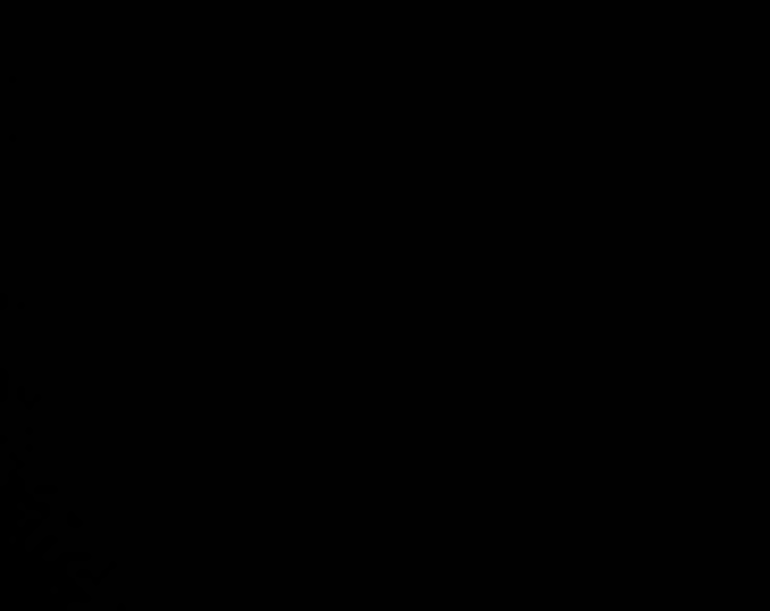

Supplement: S1 File — (ZIP) [file pone.0272206.s003.zip › new/mir378/E - 12(fld 1 wv S475_20x - HQ535_50m)_thumb.tif]

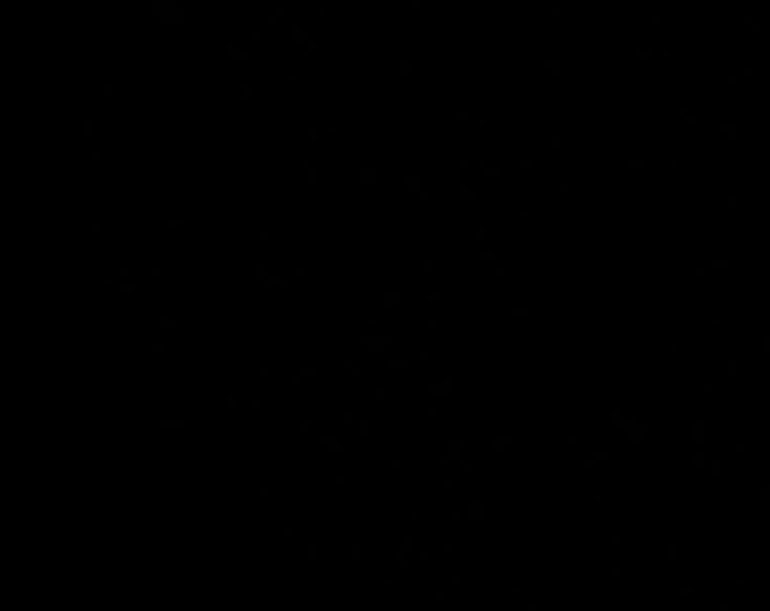

Supplement: S1 File — (ZIP) [file pone.0272206.s003.zip › new/mir378/E - 12(fld 2 wv D360_40x - HQ460_40m)_thumb.tif]

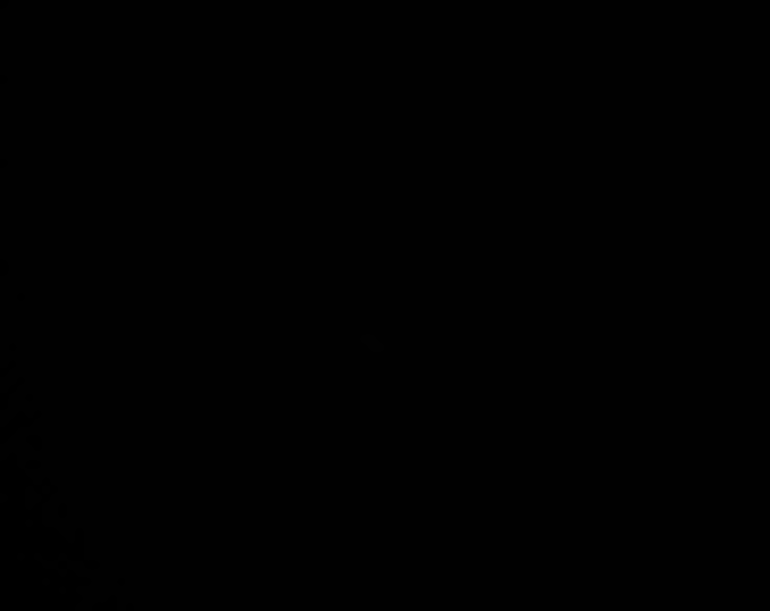

Supplement: S1 File — (ZIP) [file pone.0272206.s003.zip › new/mir378/E - 12(fld 2 wv S475_20x - HQ535_50m)_thumb.tif]

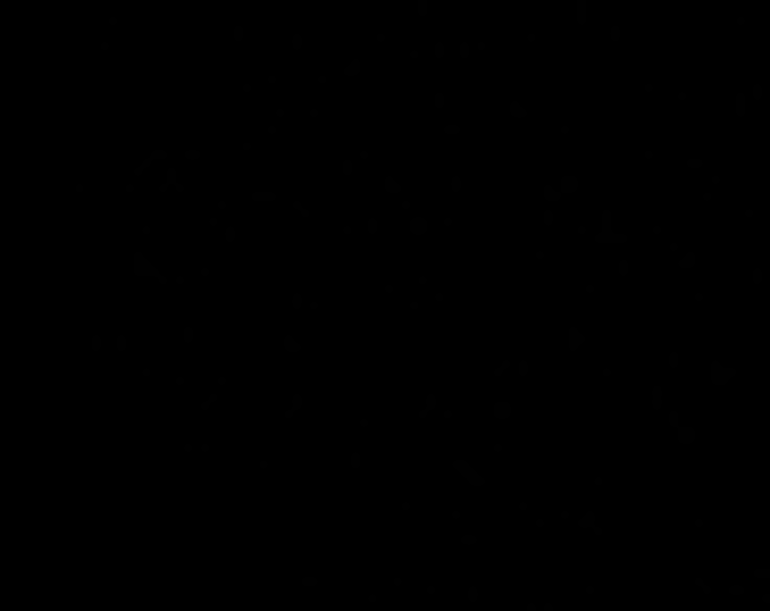

Supplement: S1 File — (ZIP) [file pone.0272206.s003.zip › new/mir378/E - 12(fld 3 wv D360_40x - HQ460_40m)_thumb.tif]

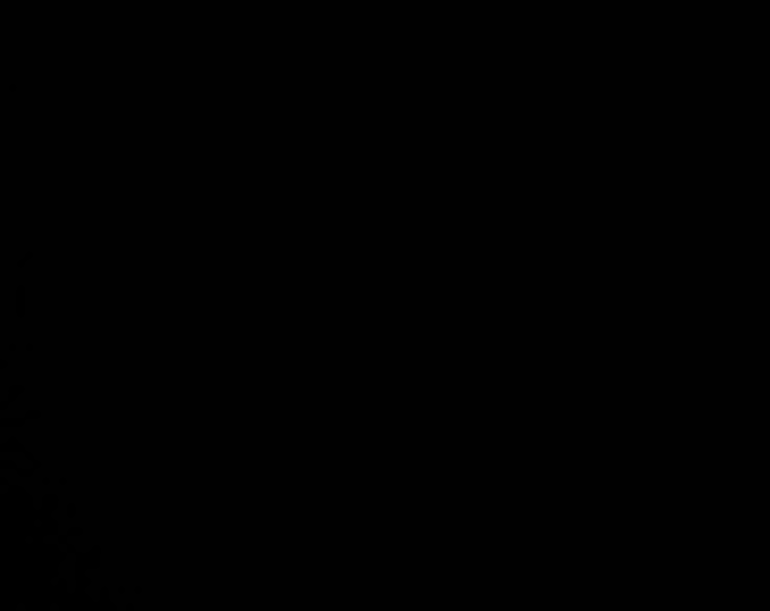

Supplement: S1 File — (ZIP) [file pone.0272206.s003.zip › new/mir378/E - 12(fld 3 wv S475_20x - HQ535_50m)_thumb.tif]

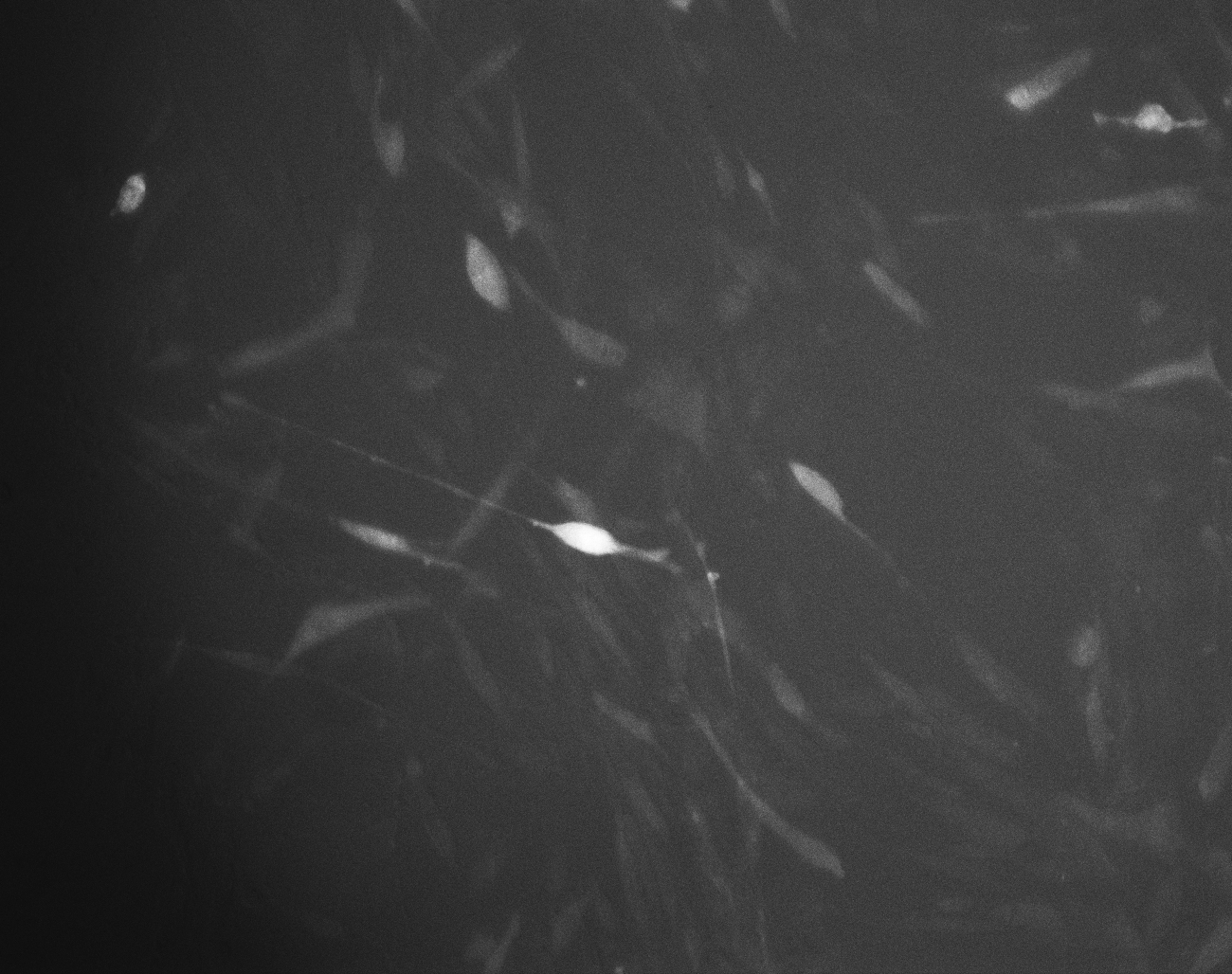

Supplement: S1 File — (ZIP) [file pone.0272206.s003.zip › new/mir378/p16.tif]

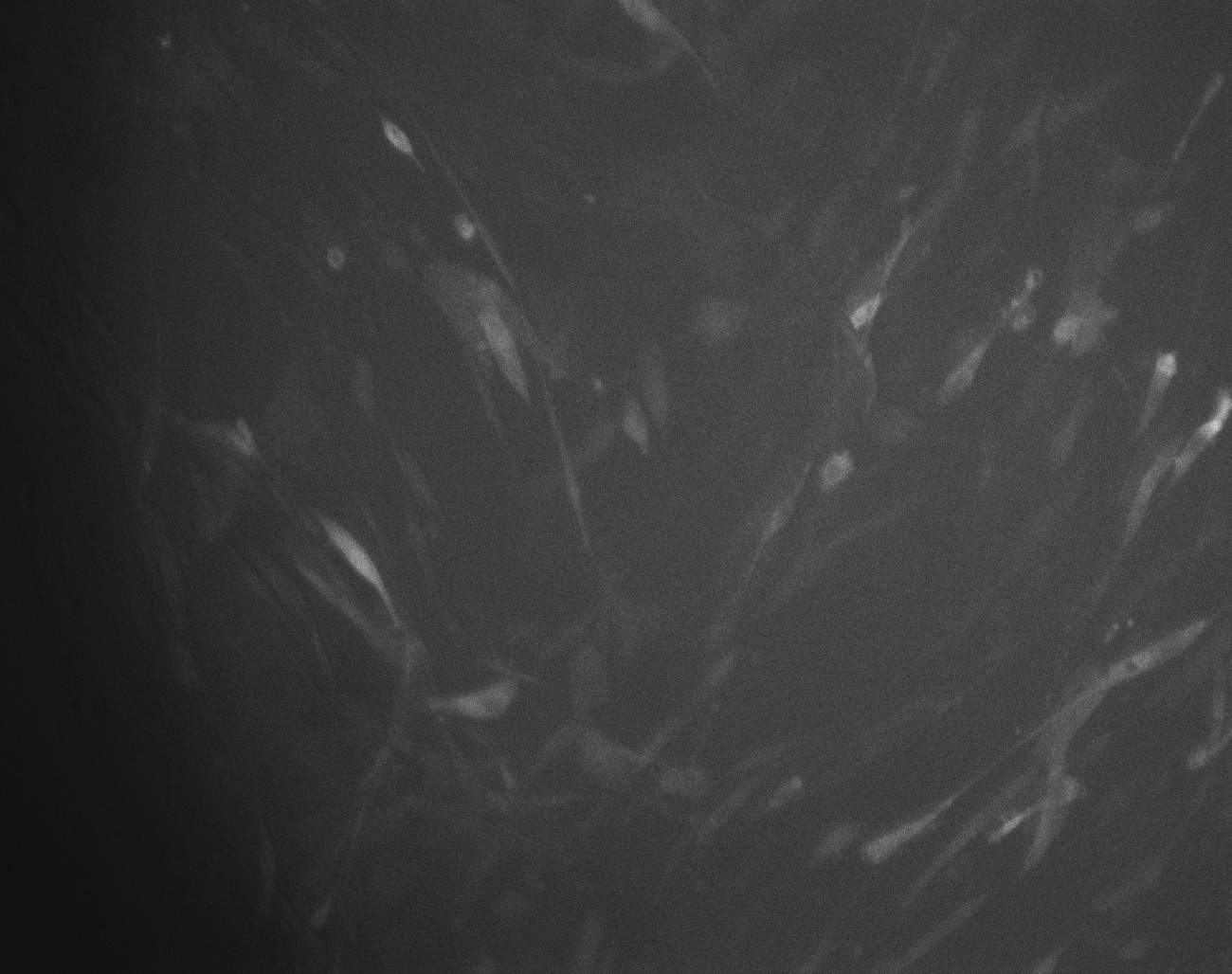

Supplement: S1 File — (ZIP) [file pone.0272206.s003.zip › new/mir378/p16_2.tif]

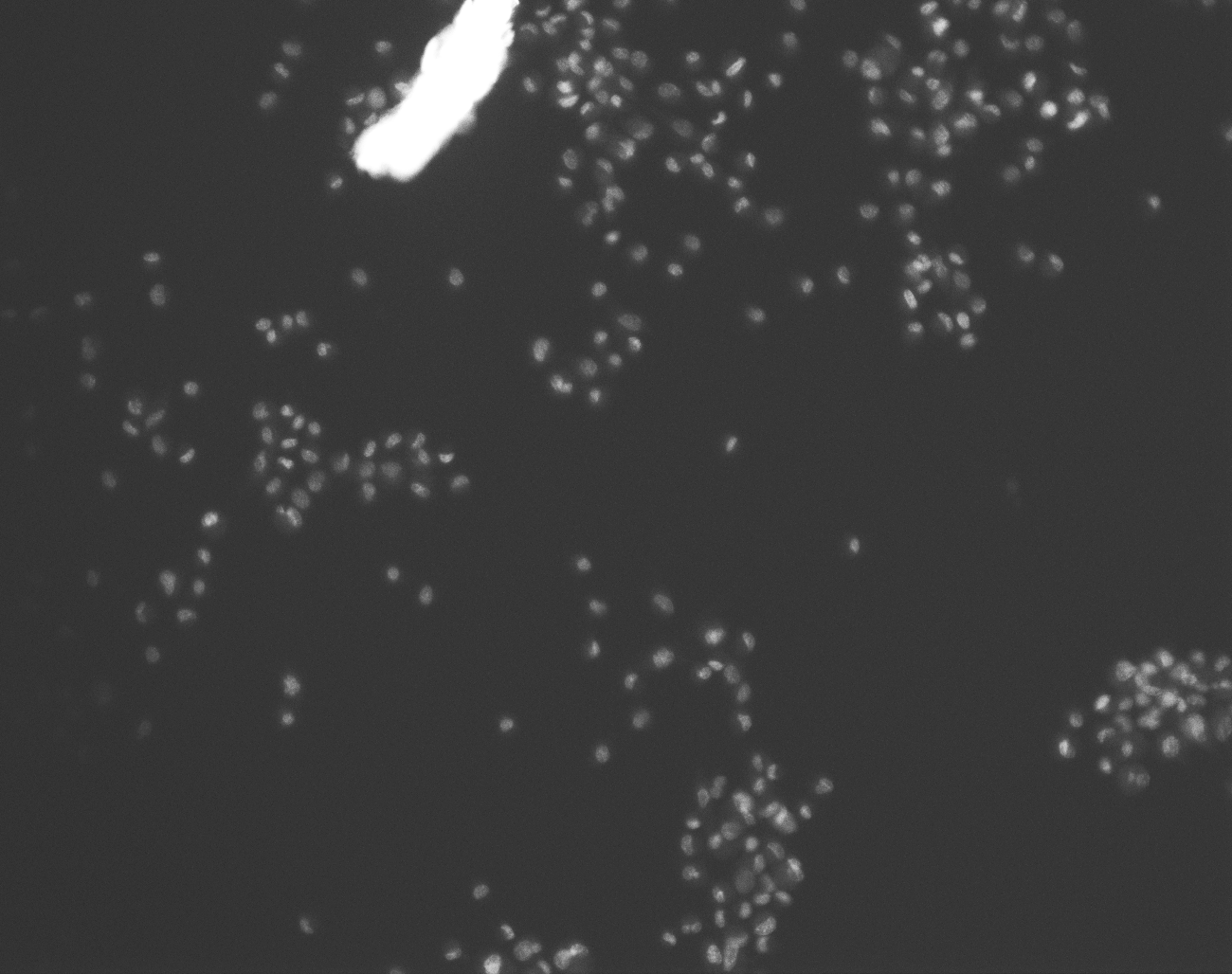

Supplement: S1 File — (ZIP) [file pone.0272206.s003.zip › new/mir429/dapi.tif]
