# Supplementary figures and images for: Correction: A Screen Identifies the Oncogenic Micro-RNA miR-378a-5p as a Negative Regulator of Oncogene-Induced Senescence (part 2 of 2)
Source: PLoS One. 2022 Jul 21;17(7):e0272206. doi: 10.1371/journal.pone.0272206 (PMC9302827; doi:10.1371/journal.pone.0272206)

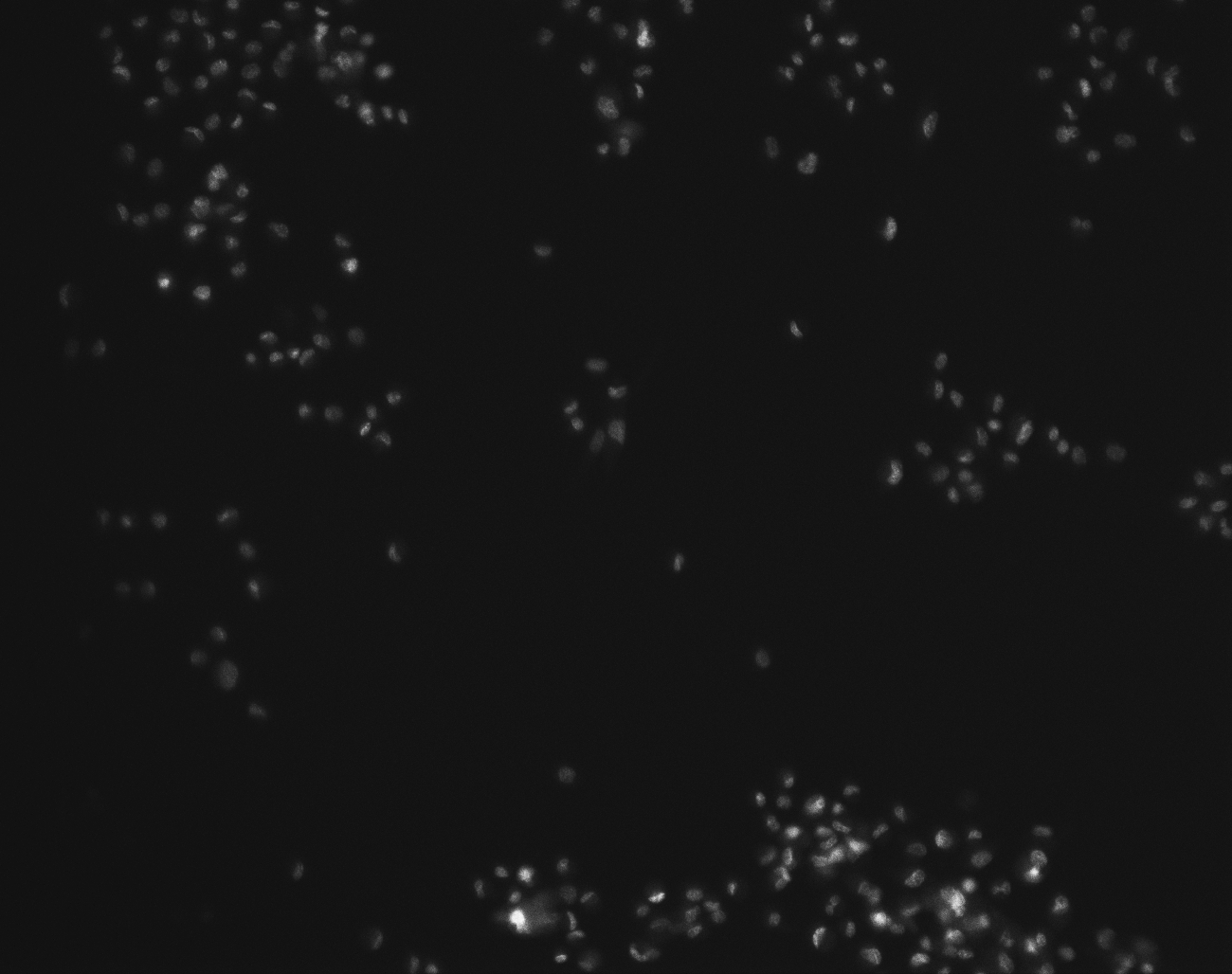

Supplement: S1 File — (ZIP) [file pone.0272206.s003.zip › new/mir429/dapi_2.tif]

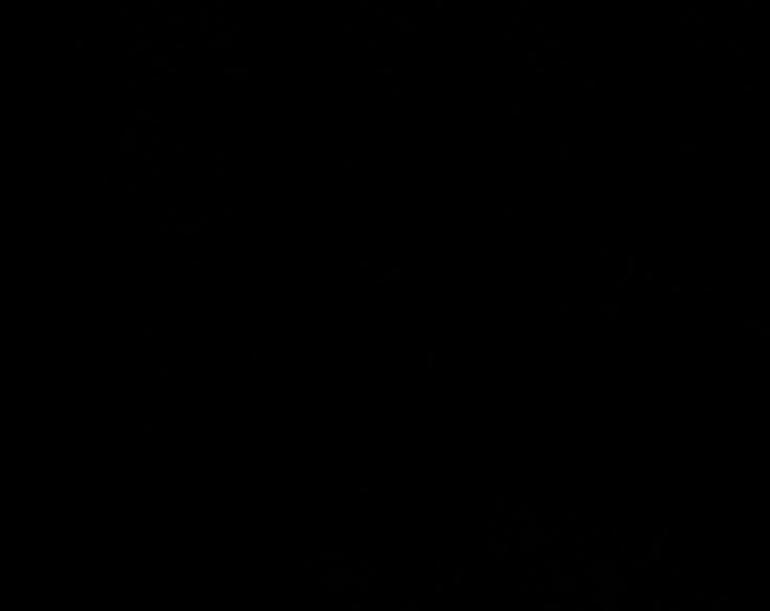

Supplement: S1 File — (ZIP) [file pone.0272206.s003.zip › new/mir429/J - 8(fld 1 wv D360_40x - HQ460_40m)_thumb.tif]

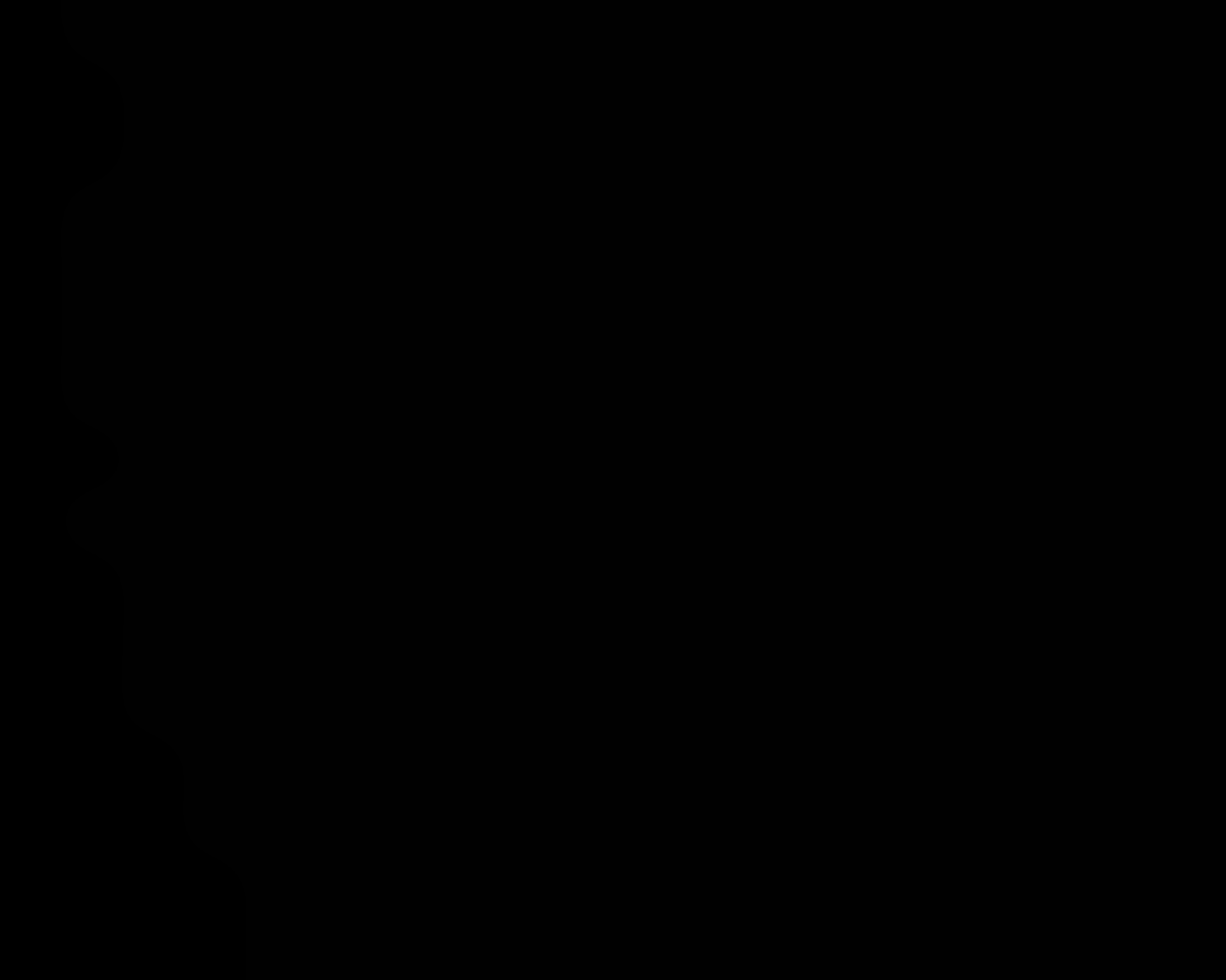

Supplement: S1 File — (ZIP) [file pone.0272206.s003.zip › new/mir429/J - 8(fld 1 wv S475_20x - HQ535_50m).tif]

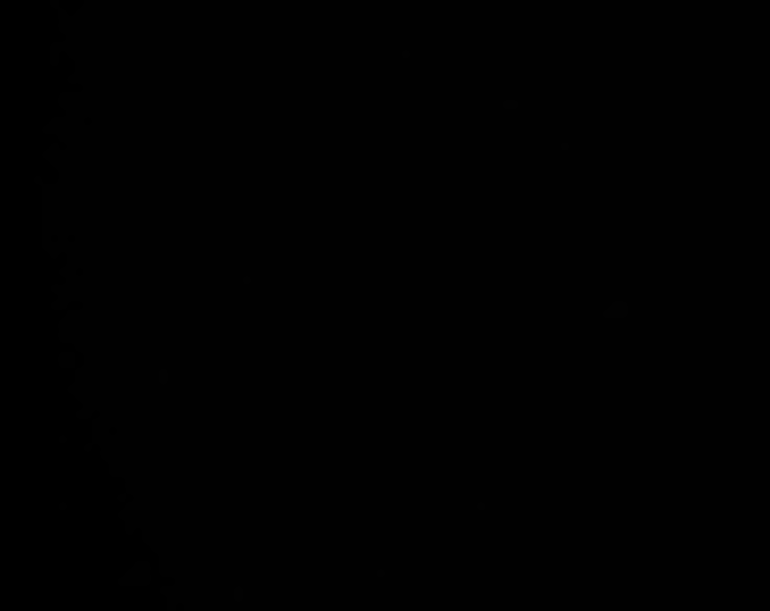

Supplement: S1 File — (ZIP) [file pone.0272206.s003.zip › new/mir429/J - 8(fld 1 wv S475_20x - HQ535_50m)_thumb.tif]

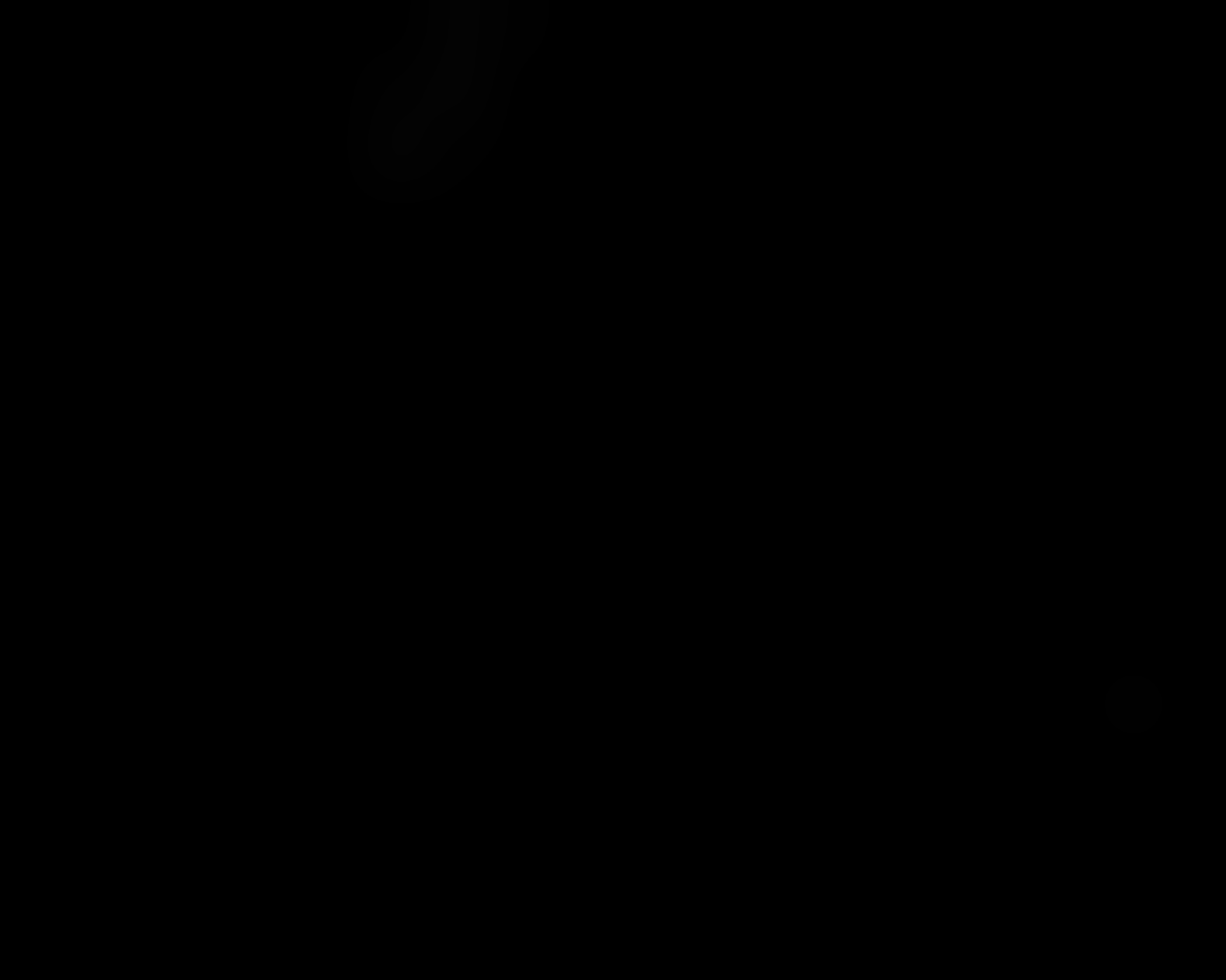

Supplement: S1 File — (ZIP) [file pone.0272206.s003.zip › new/mir429/J - 8(fld 2 wv D360_40x - HQ460_40m).tif]

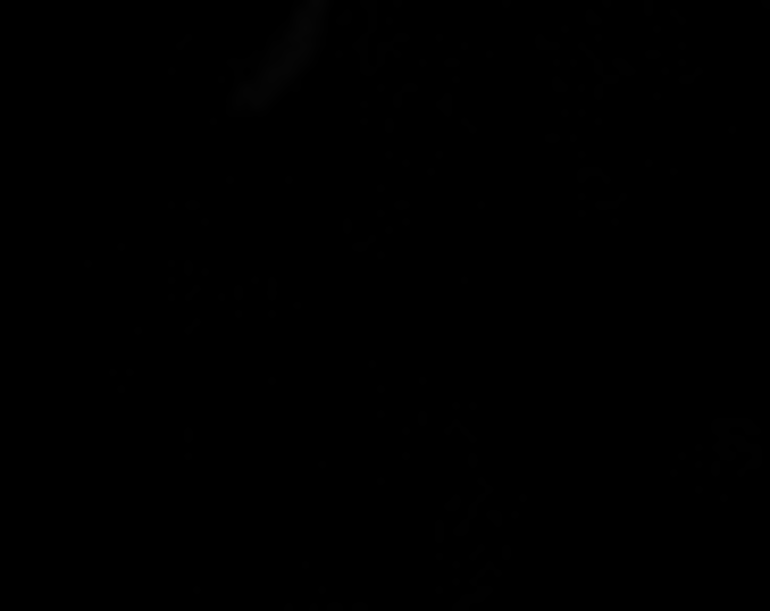

Supplement: S1 File — (ZIP) [file pone.0272206.s003.zip › new/mir429/J - 8(fld 2 wv D360_40x - HQ460_40m)_thumb.tif]

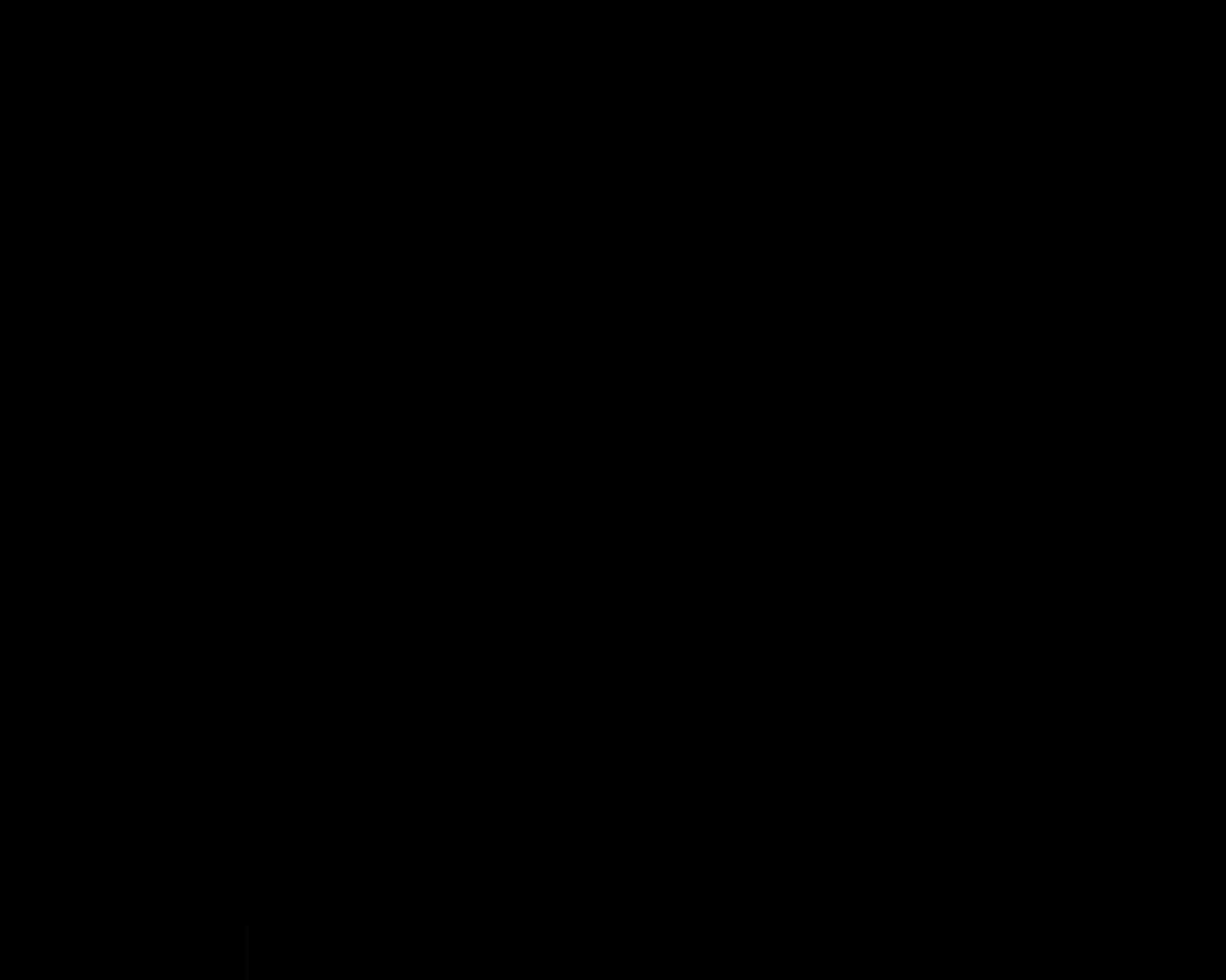

Supplement: S1 File — (ZIP) [file pone.0272206.s003.zip › new/mir429/J - 8(fld 2 wv S475_20x - HQ535_50m).tif]

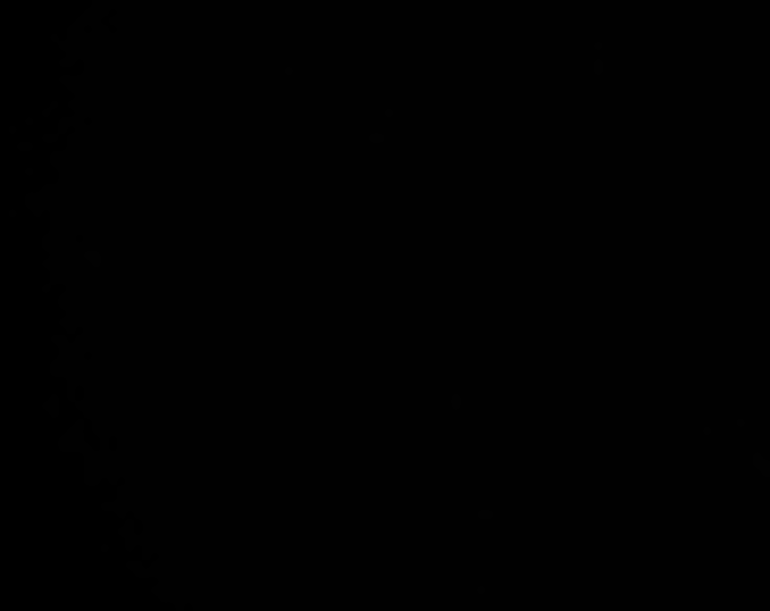

Supplement: S1 File — (ZIP) [file pone.0272206.s003.zip › new/mir429/J - 8(fld 2 wv S475_20x - HQ535_50m)_thumb.tif]

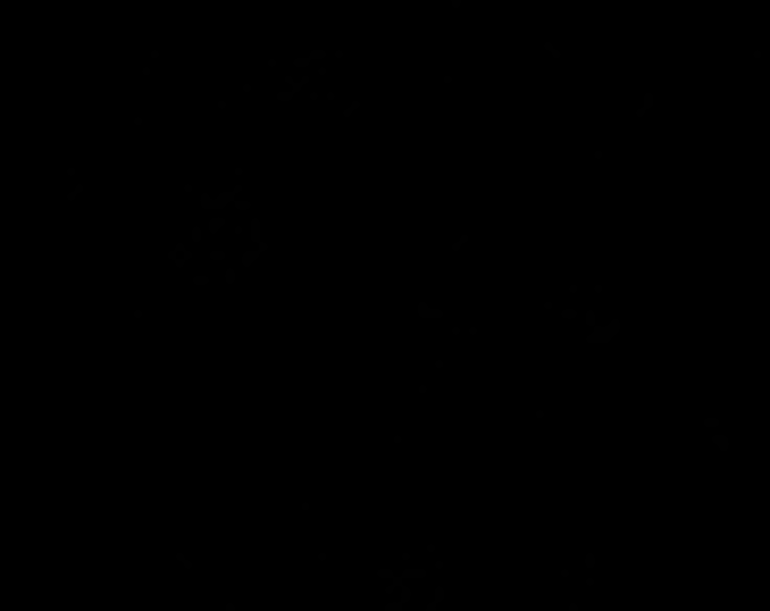

Supplement: S1 File — (ZIP) [file pone.0272206.s003.zip › new/mir429/J - 8(fld 3 wv D360_40x - HQ460_40m)_thumb.tif]

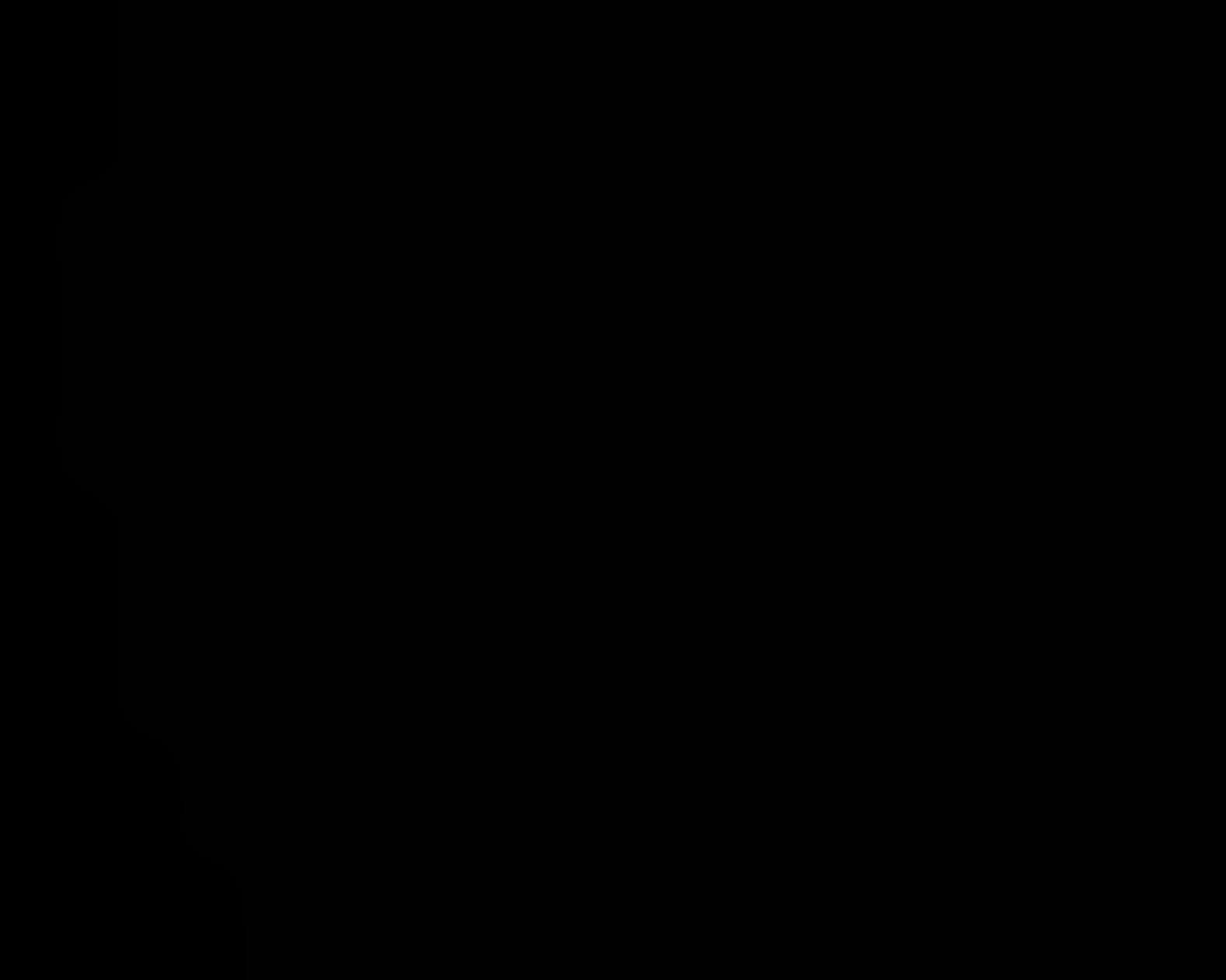

Supplement: S1 File — (ZIP) [file pone.0272206.s003.zip › new/mir429/J - 8(fld 3 wv S475_20x - HQ535_50m).tif]

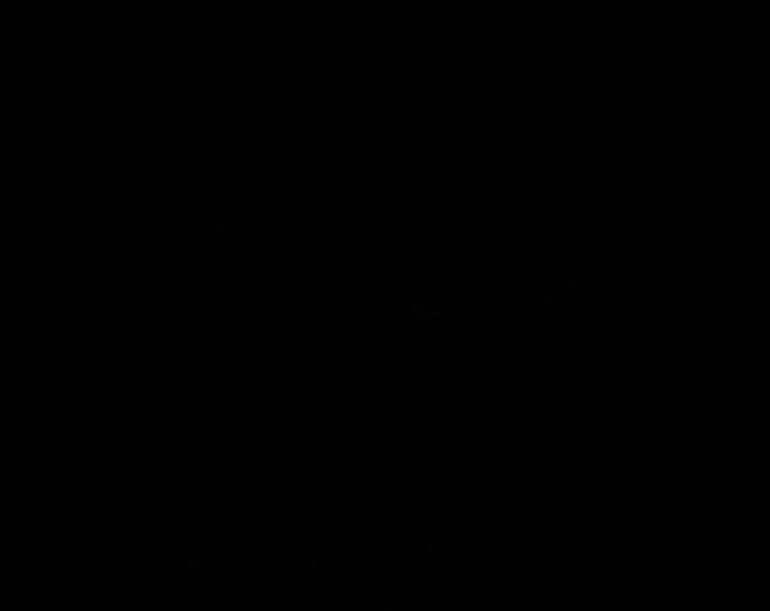

Supplement: S1 File — (ZIP) [file pone.0272206.s003.zip › new/mir429/J - 8(fld 3 wv S475_20x - HQ535_50m)_thumb.tif]

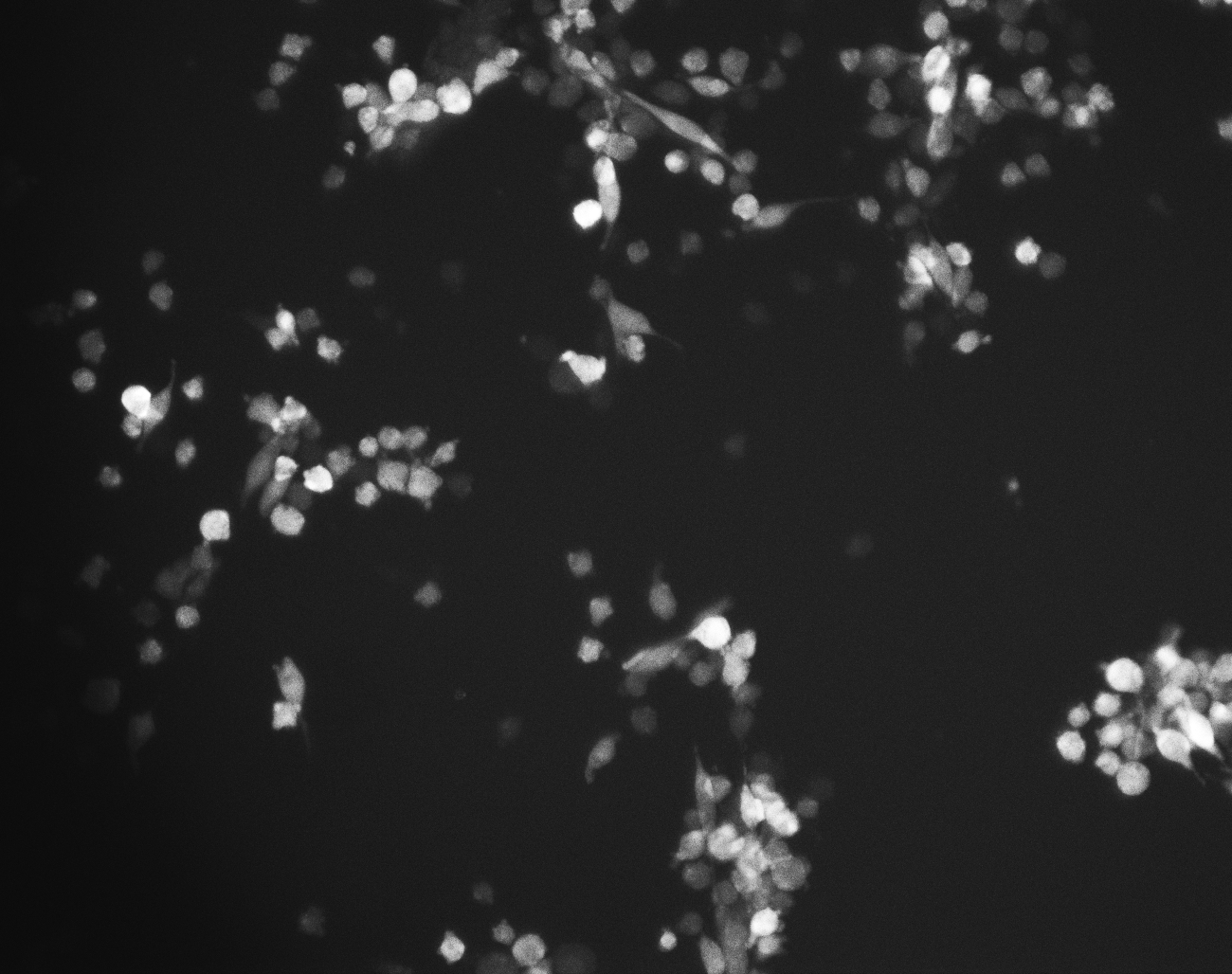

Supplement: S1 File — (ZIP) [file pone.0272206.s003.zip › new/mir429/p16.tif]

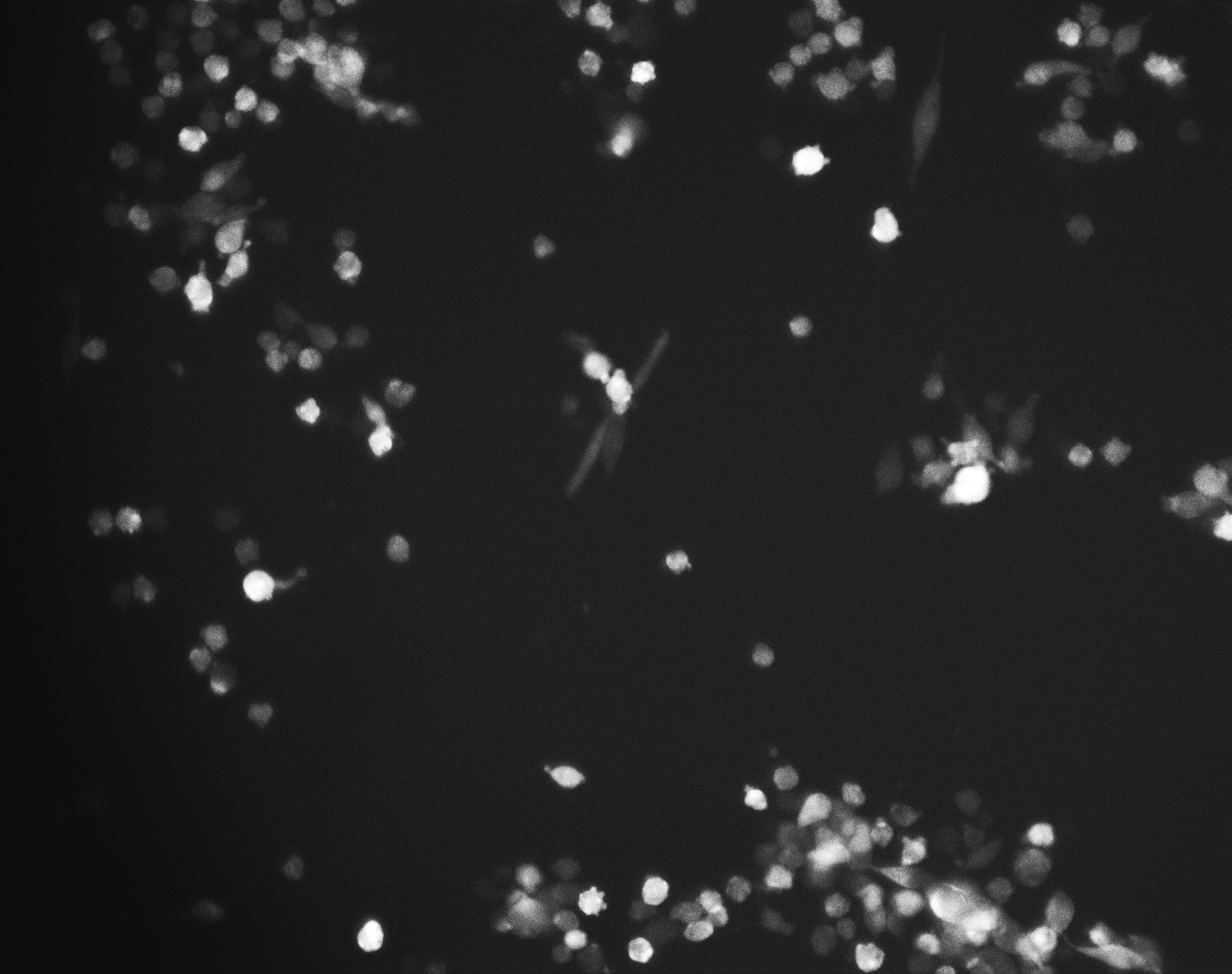

Supplement: S1 File — (ZIP) [file pone.0272206.s003.zip › new/mir429/p16_2.tif]

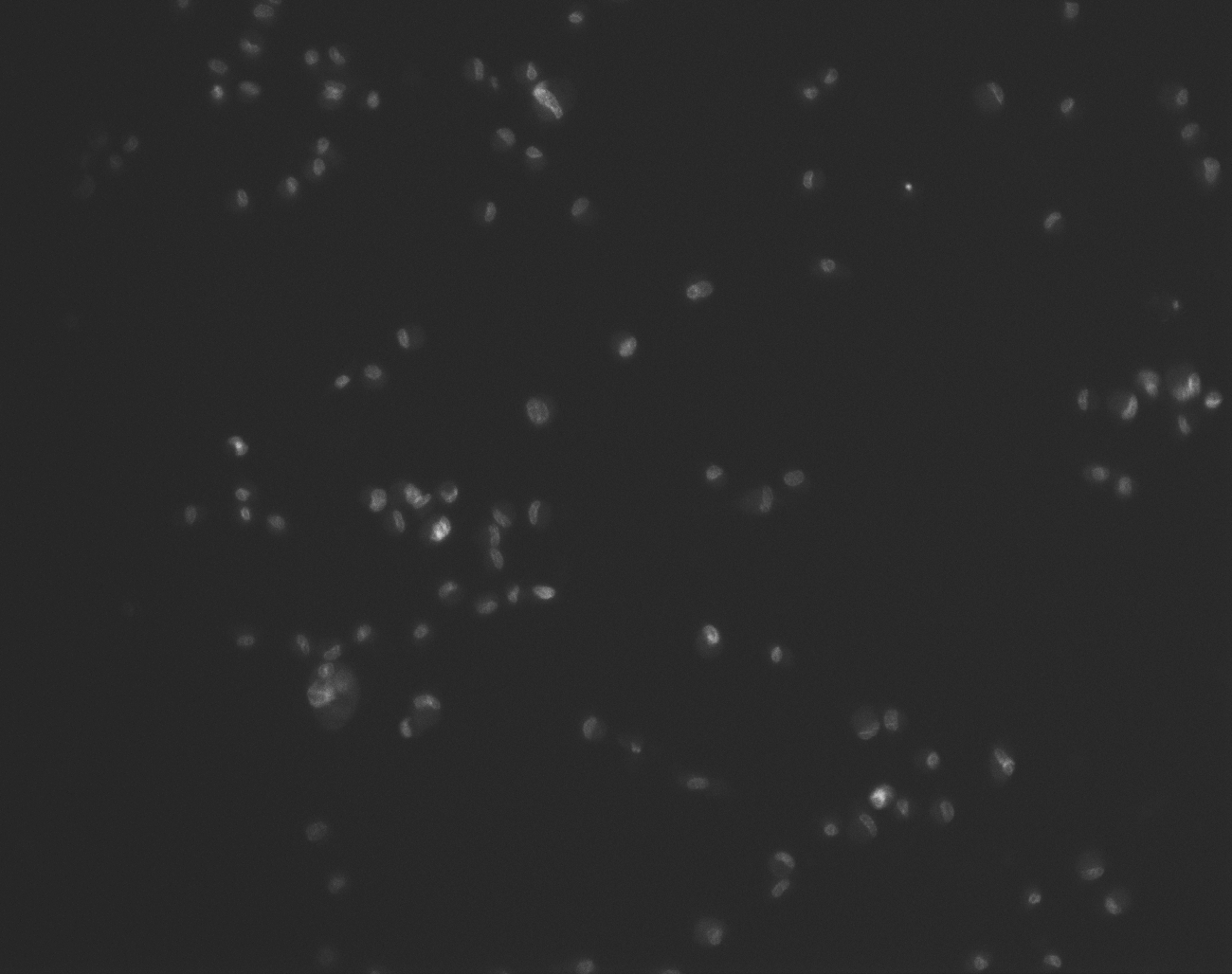

Supplement: S1 File — (ZIP) [file pone.0272206.s003.zip › new/mir449/dapi.tif]

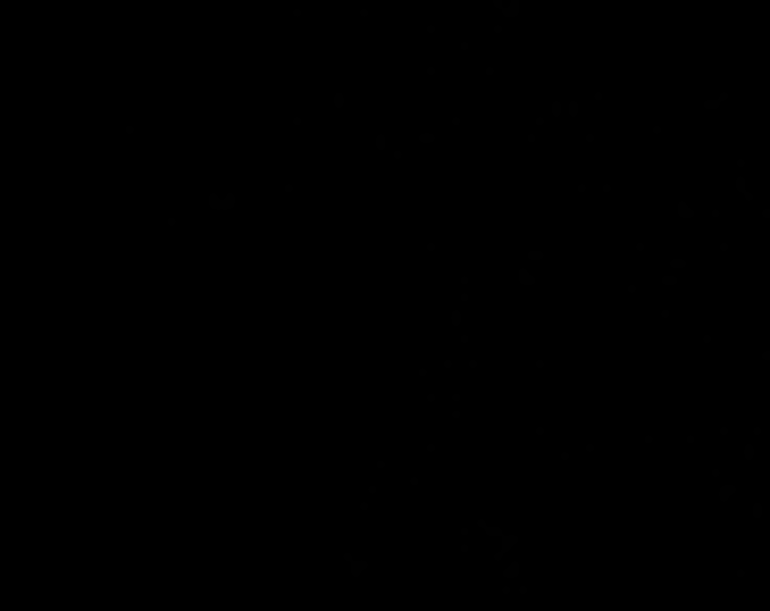

Supplement: S1 File — (ZIP) [file pone.0272206.s003.zip › new/mir449/I - 19(fld 1 wv D360_40x - HQ460_40m)_thumb.tif]

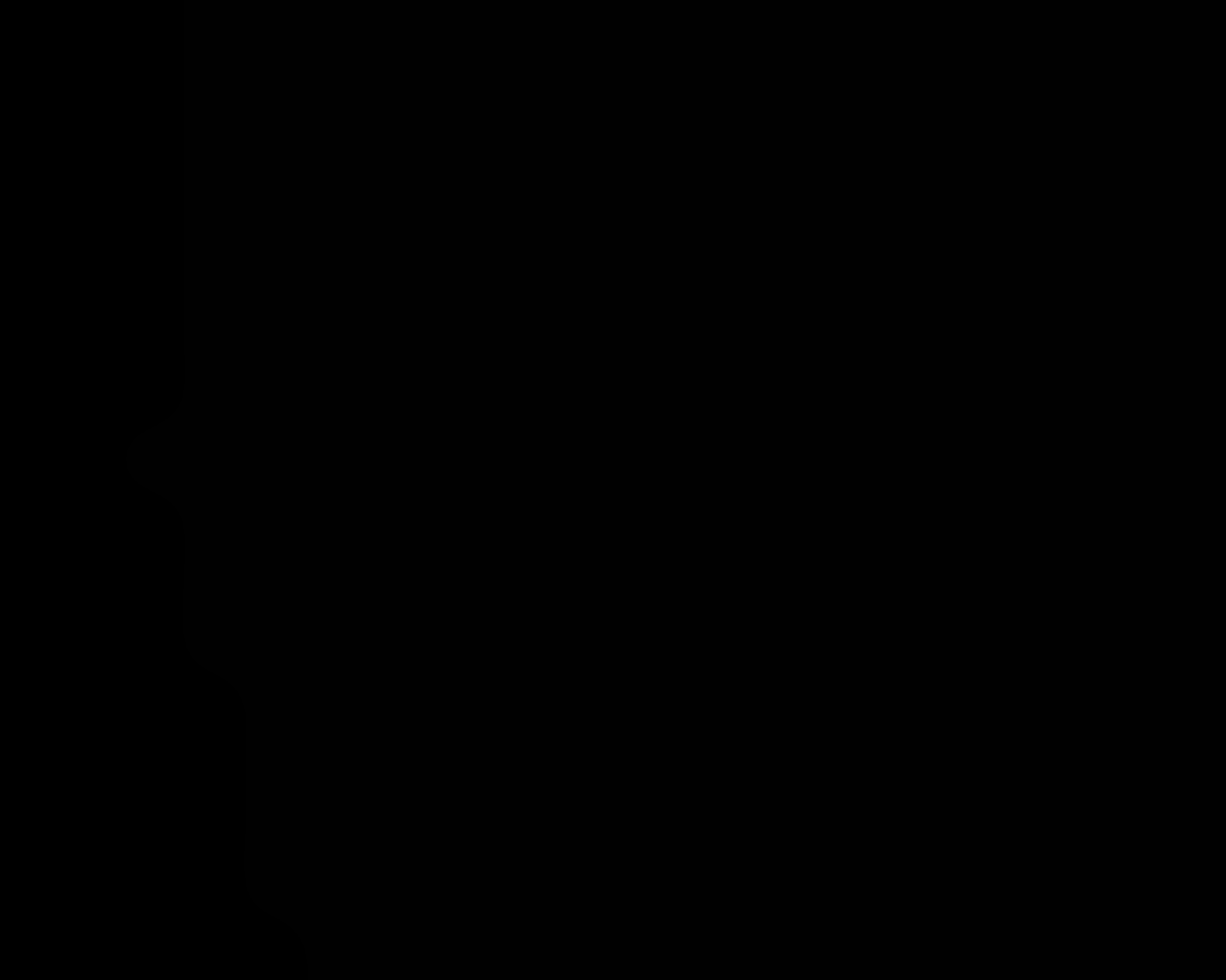

Supplement: S1 File — (ZIP) [file pone.0272206.s003.zip › new/mir449/I - 19(fld 1 wv S475_20x - HQ535_50m).tif]

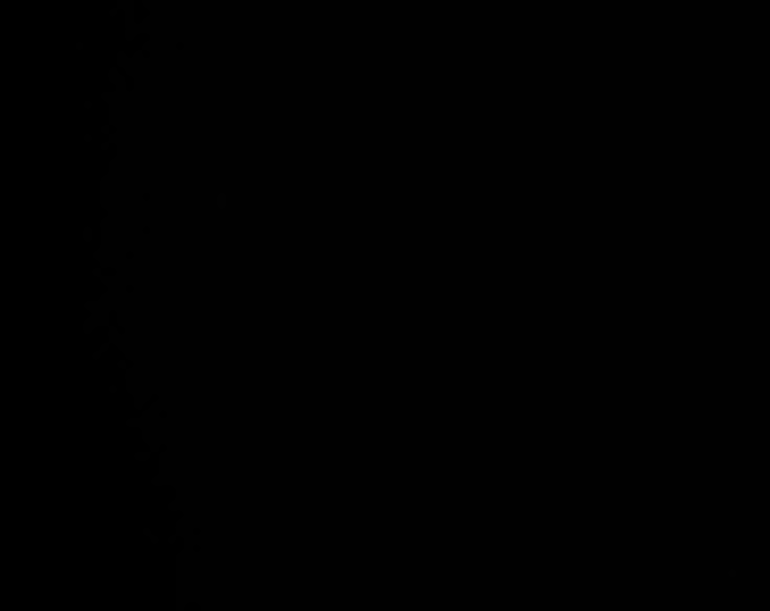

Supplement: S1 File — (ZIP) [file pone.0272206.s003.zip › new/mir449/I - 19(fld 1 wv S475_20x - HQ535_50m)_thumb.tif]

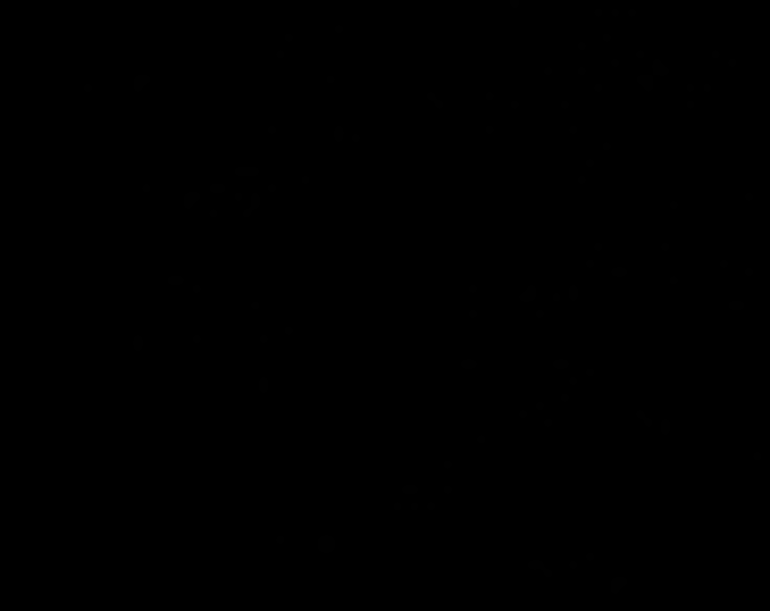

Supplement: S1 File — (ZIP) [file pone.0272206.s003.zip › new/mir449/I - 19(fld 2 wv D360_40x - HQ460_40m)_thumb.tif]

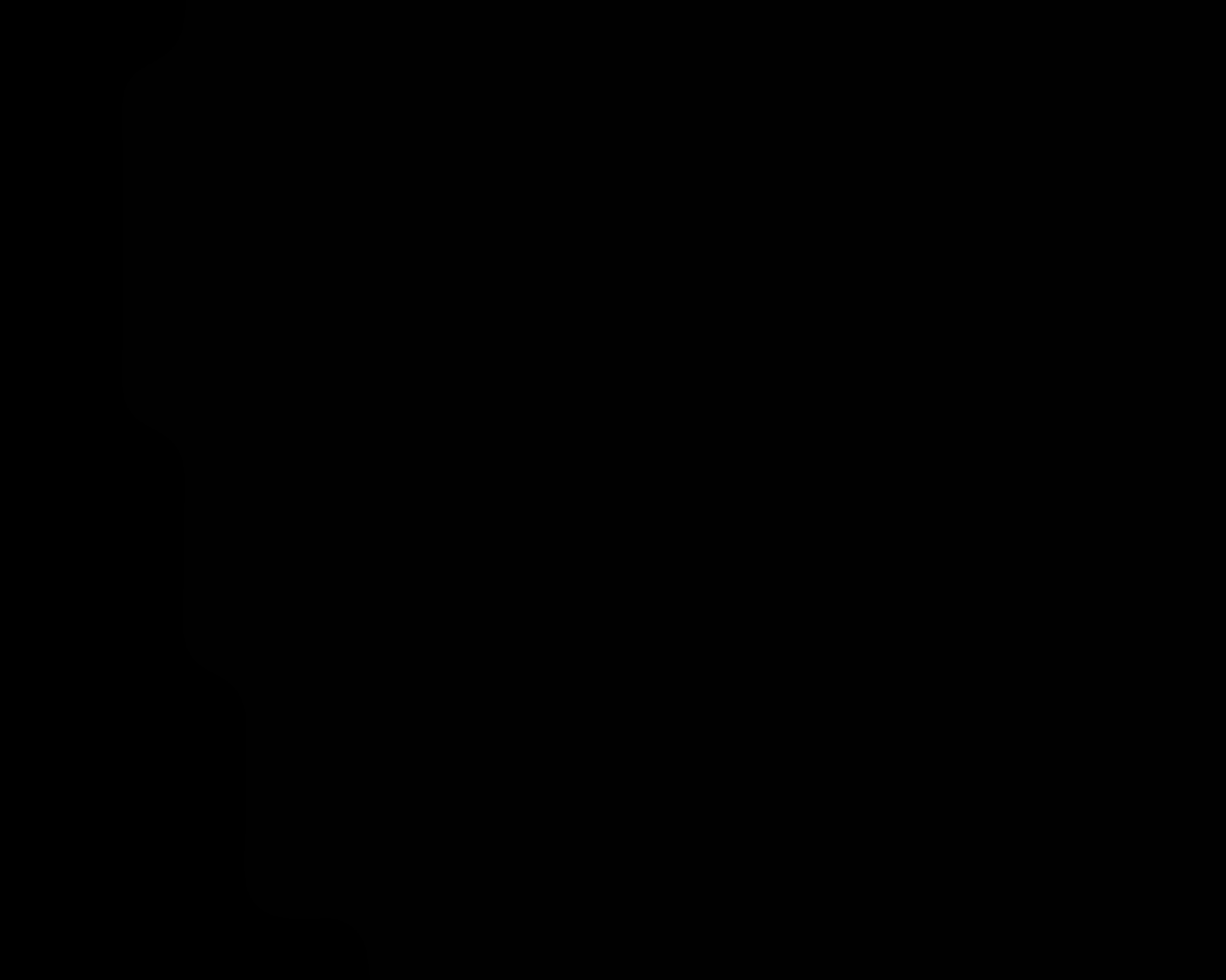

Supplement: S1 File — (ZIP) [file pone.0272206.s003.zip › new/mir449/I - 19(fld 2 wv S475_20x - HQ535_50m).tif]

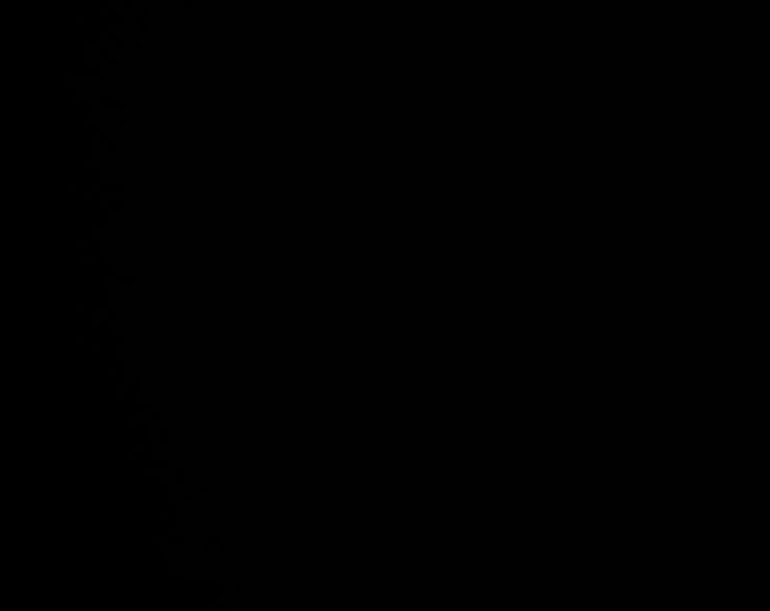

Supplement: S1 File — (ZIP) [file pone.0272206.s003.zip › new/mir449/I - 19(fld 2 wv S475_20x - HQ535_50m)_thumb.tif]

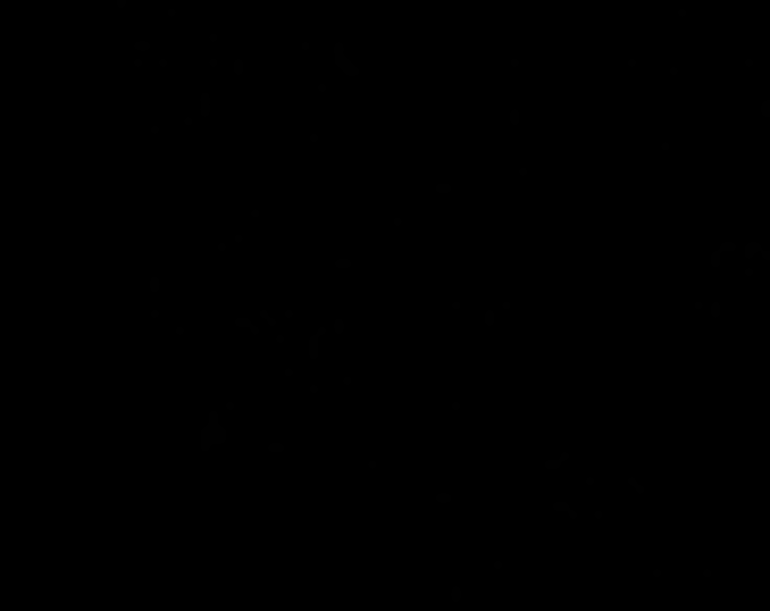

Supplement: S1 File — (ZIP) [file pone.0272206.s003.zip › new/mir449/I - 19(fld 3 wv D360_40x - HQ460_40m)_thumb.tif]

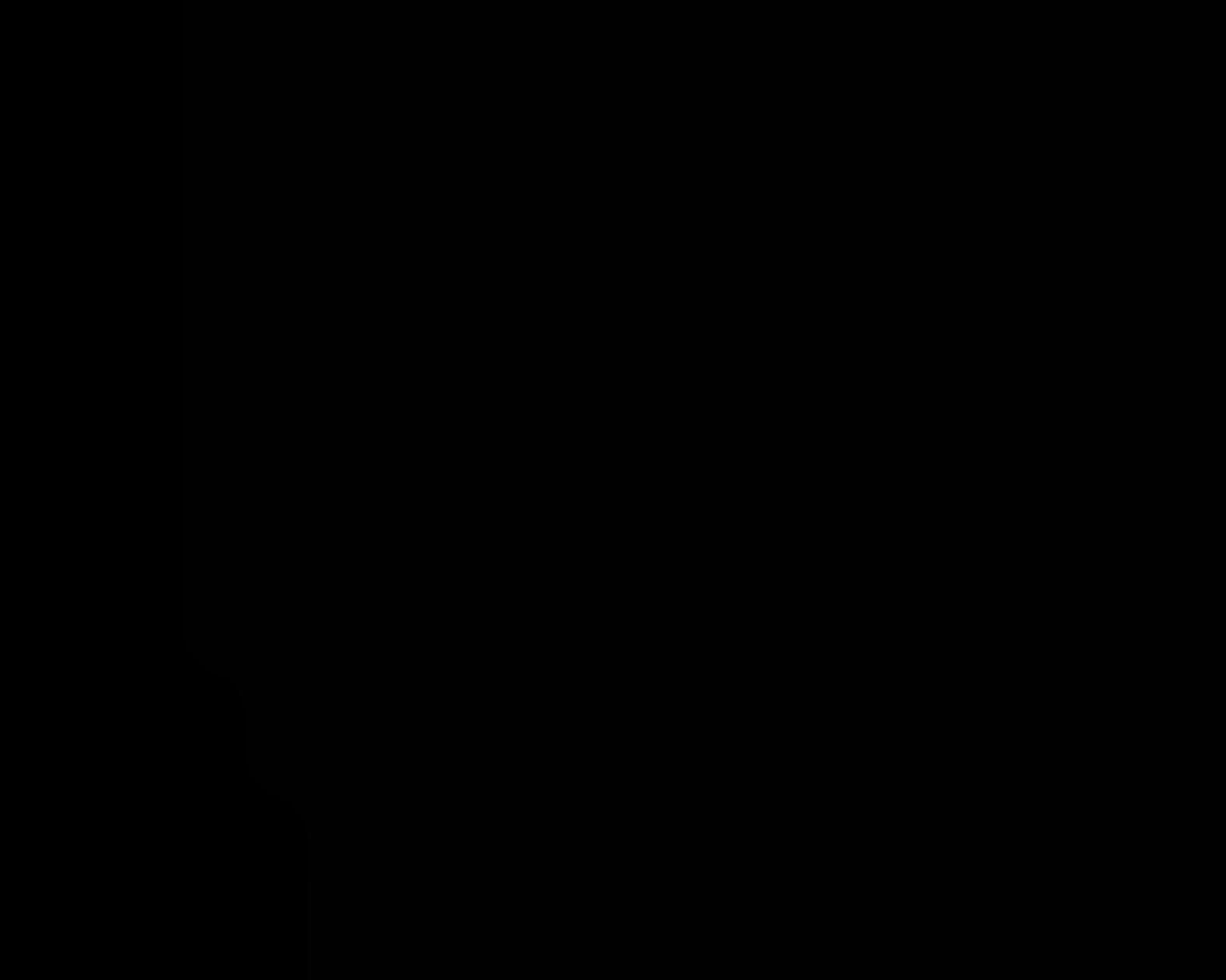

Supplement: S1 File — (ZIP) [file pone.0272206.s003.zip › new/mir449/I - 19(fld 3 wv S475_20x - HQ535_50m).tif]

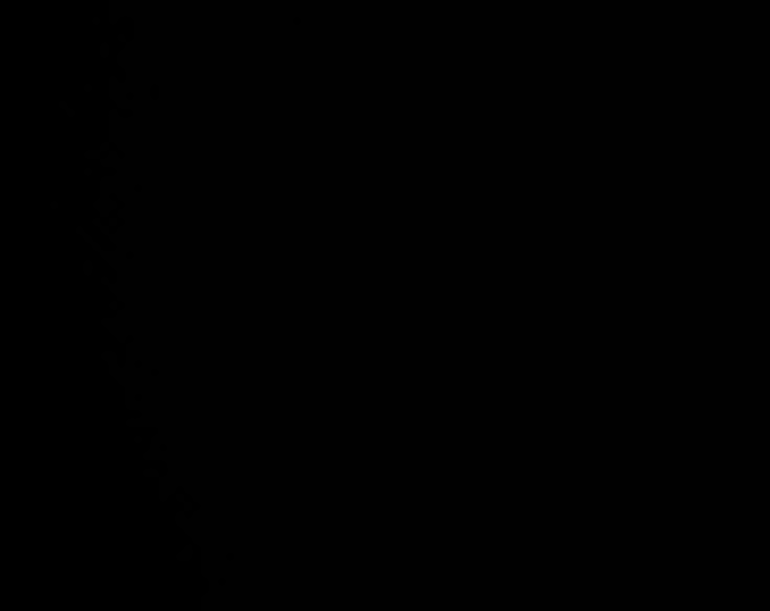

Supplement: S1 File — (ZIP) [file pone.0272206.s003.zip › new/mir449/I - 19(fld 3 wv S475_20x - HQ535_50m)_thumb.tif]

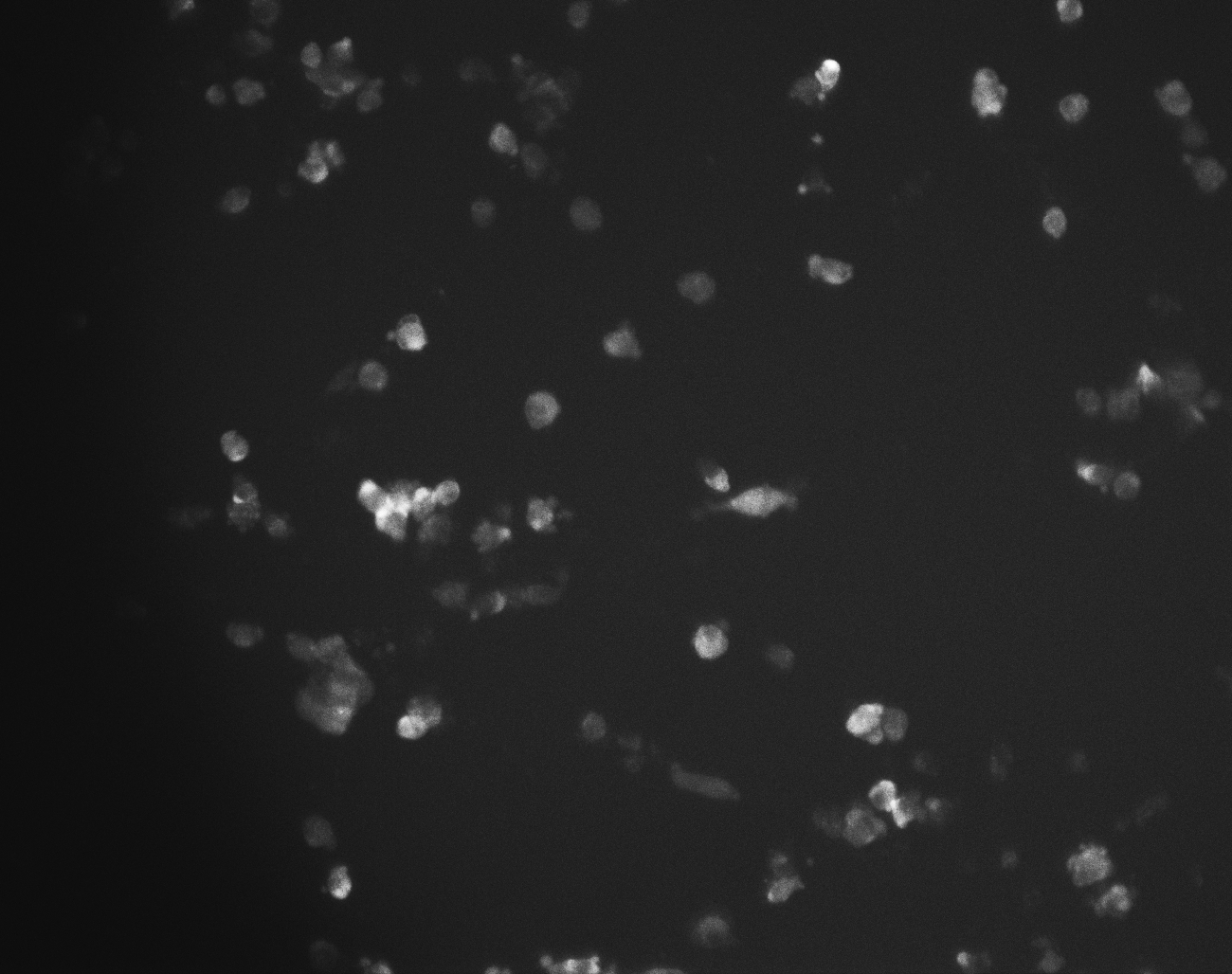

Supplement: S1 File — (ZIP) [file pone.0272206.s003.zip › new/mir449/p16.tif]

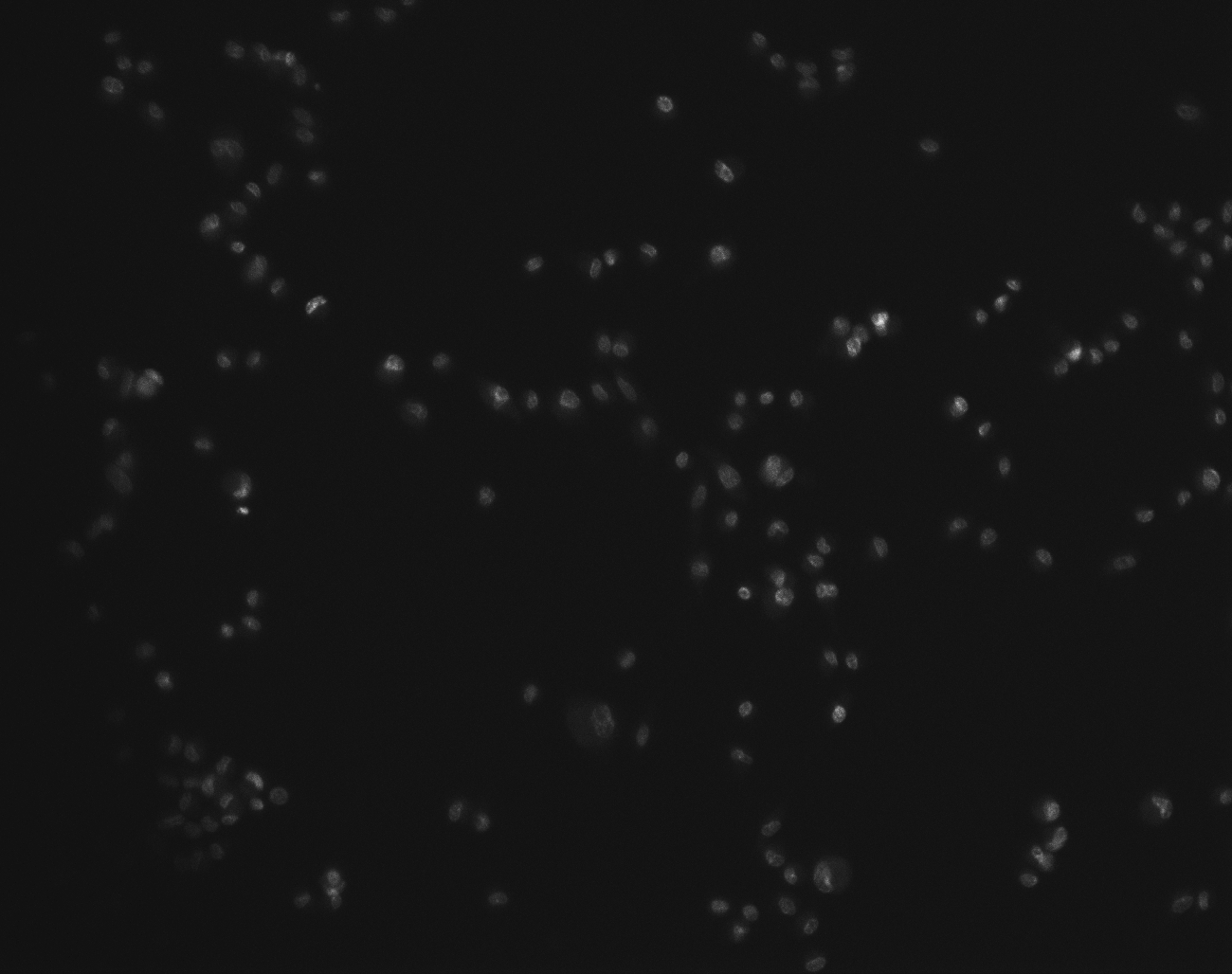

Supplement: S1 File — (ZIP) [file pone.0272206.s003.zip › new/miR449b/dapi.tif]

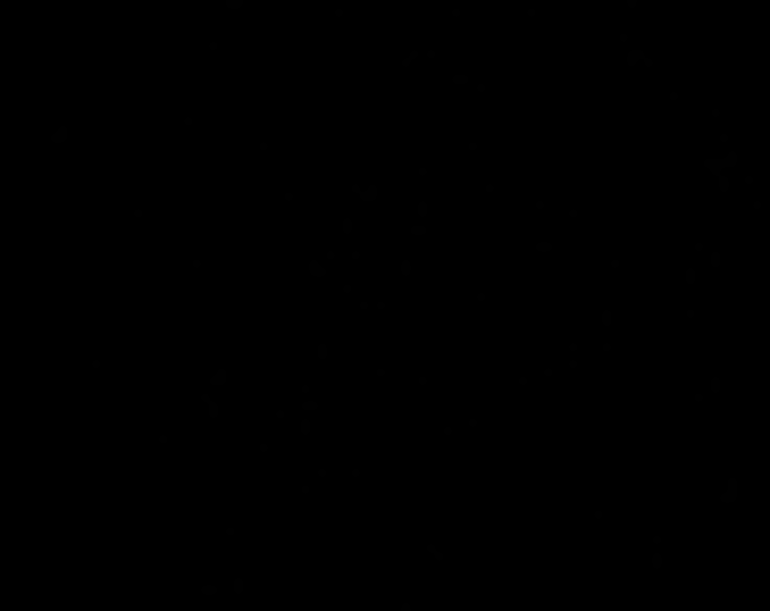

Supplement: S1 File — (ZIP) [file pone.0272206.s003.zip › new/miR449b/J - 22(fld 1 wv D360_40x - HQ460_40m)_thumb.tif]

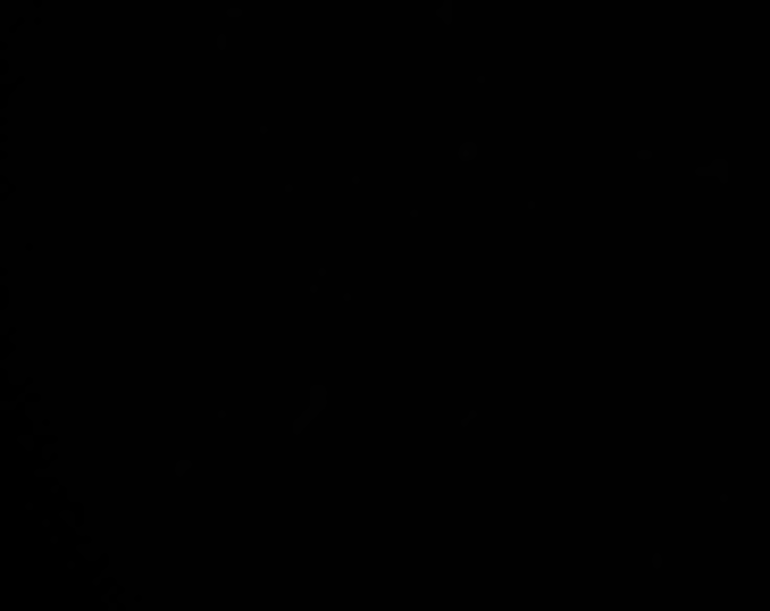

Supplement: S1 File — (ZIP) [file pone.0272206.s003.zip › new/miR449b/J - 22(fld 1 wv S475_20x - HQ535_50m)_thumb.tif]

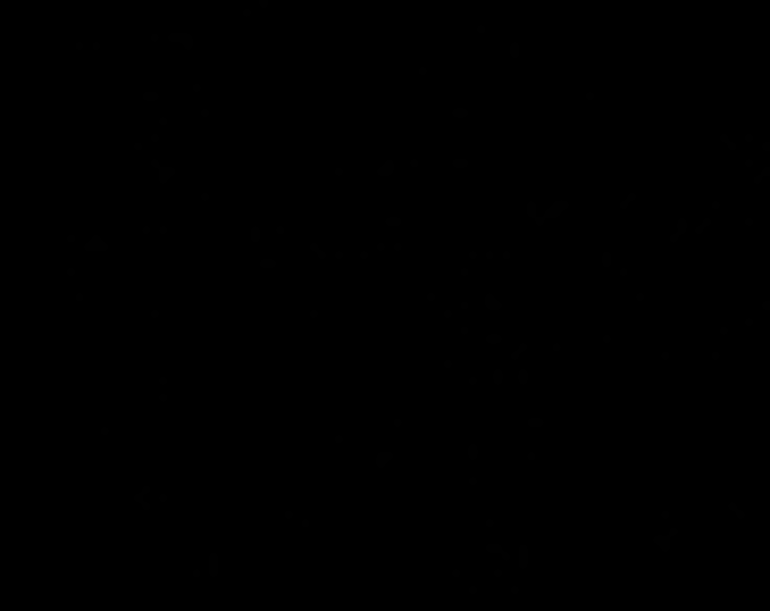

Supplement: S1 File — (ZIP) [file pone.0272206.s003.zip › new/miR449b/J - 22(fld 2 wv D360_40x - HQ460_40m)_thumb.tif]

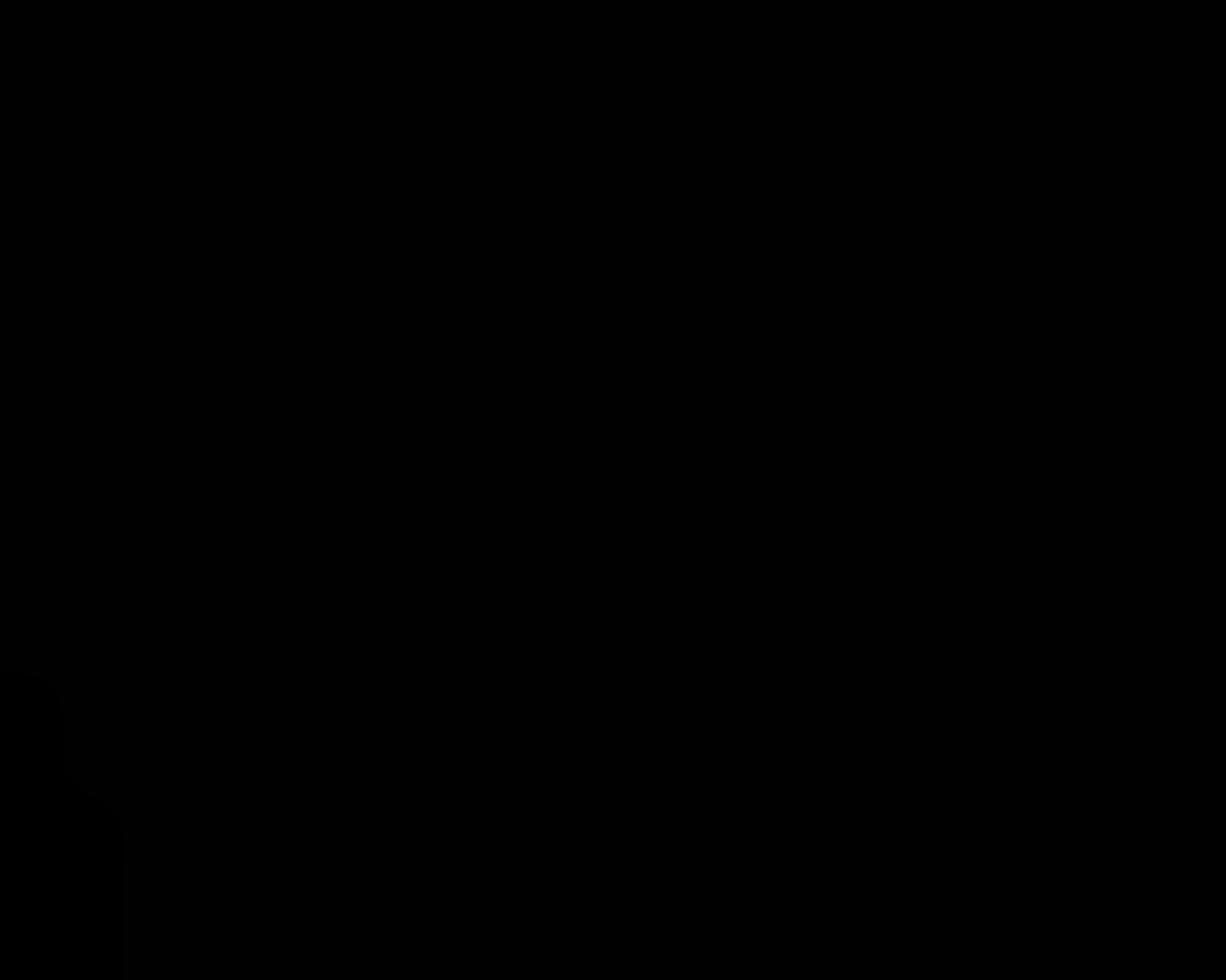

Supplement: S1 File — (ZIP) [file pone.0272206.s003.zip › new/miR449b/J - 22(fld 2 wv S475_20x - HQ535_50m).tif]

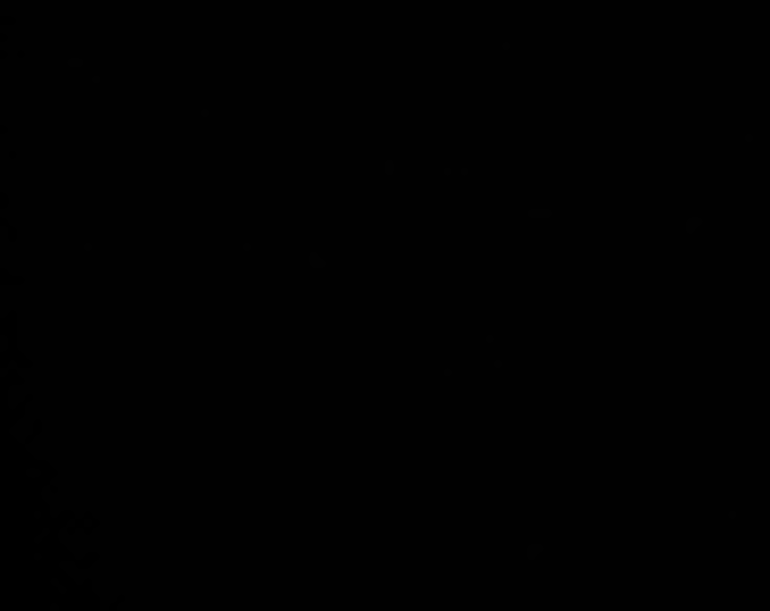

Supplement: S1 File — (ZIP) [file pone.0272206.s003.zip › new/miR449b/J - 22(fld 2 wv S475_20x - HQ535_50m)_thumb.tif]

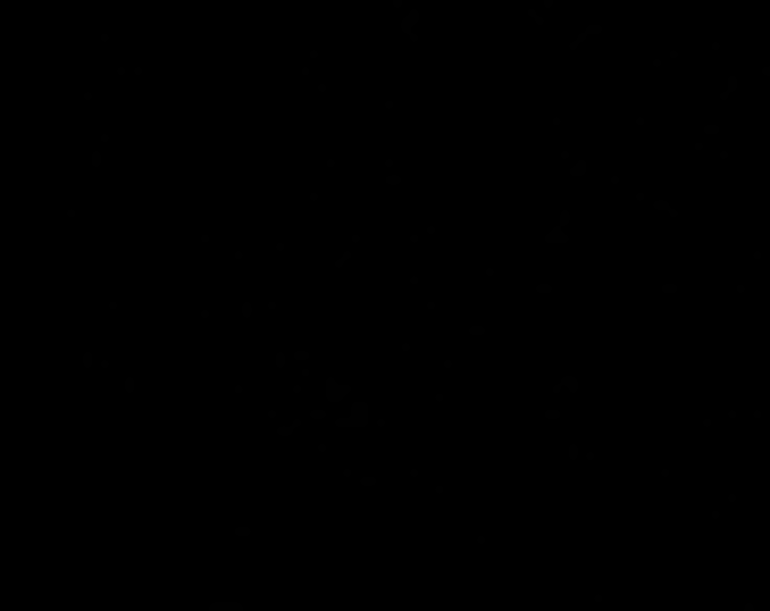

Supplement: S1 File — (ZIP) [file pone.0272206.s003.zip › new/miR449b/J - 22(fld 3 wv D360_40x - HQ460_40m)_thumb.tif]

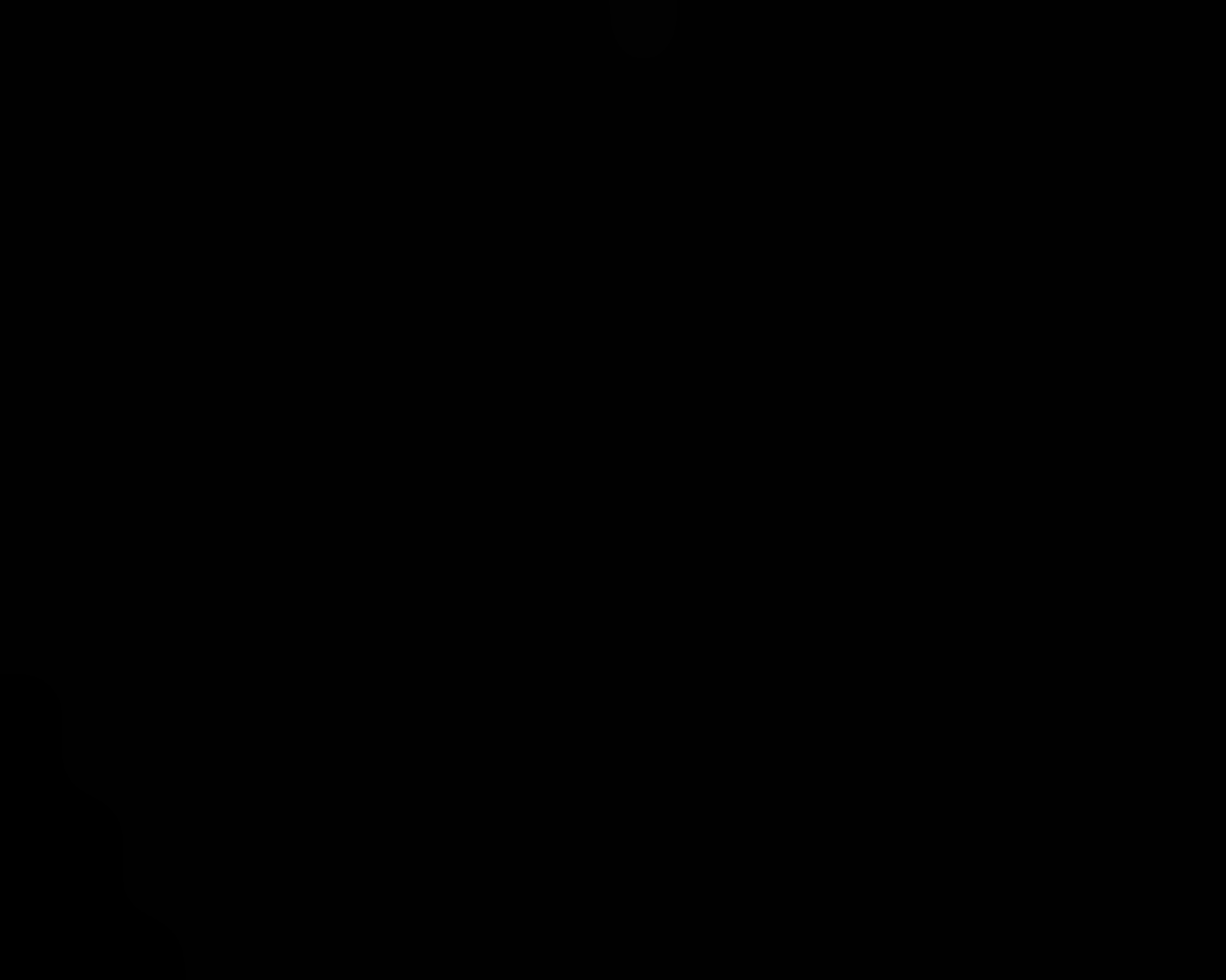

Supplement: S1 File — (ZIP) [file pone.0272206.s003.zip › new/miR449b/J - 22(fld 3 wv S475_20x - HQ535_50m).tif]

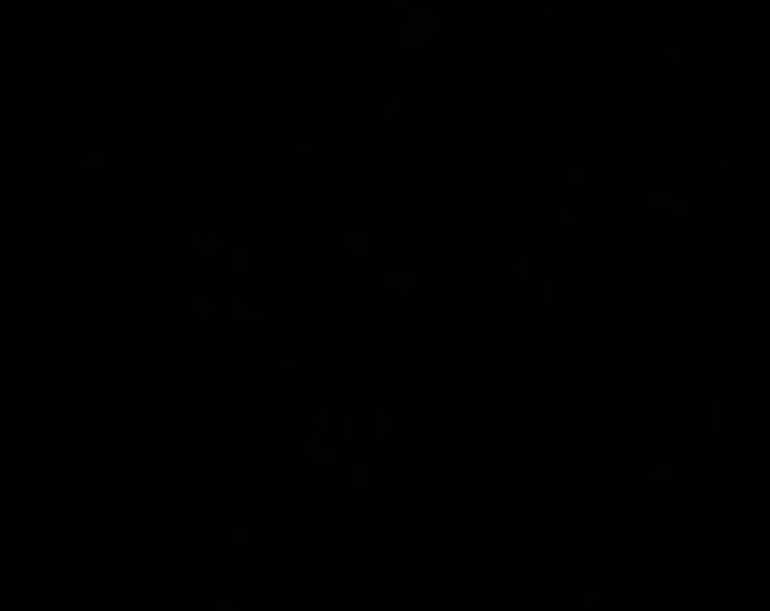

Supplement: S1 File — (ZIP) [file pone.0272206.s003.zip › new/miR449b/J - 22(fld 3 wv S475_20x - HQ535_50m)_thumb.tif]

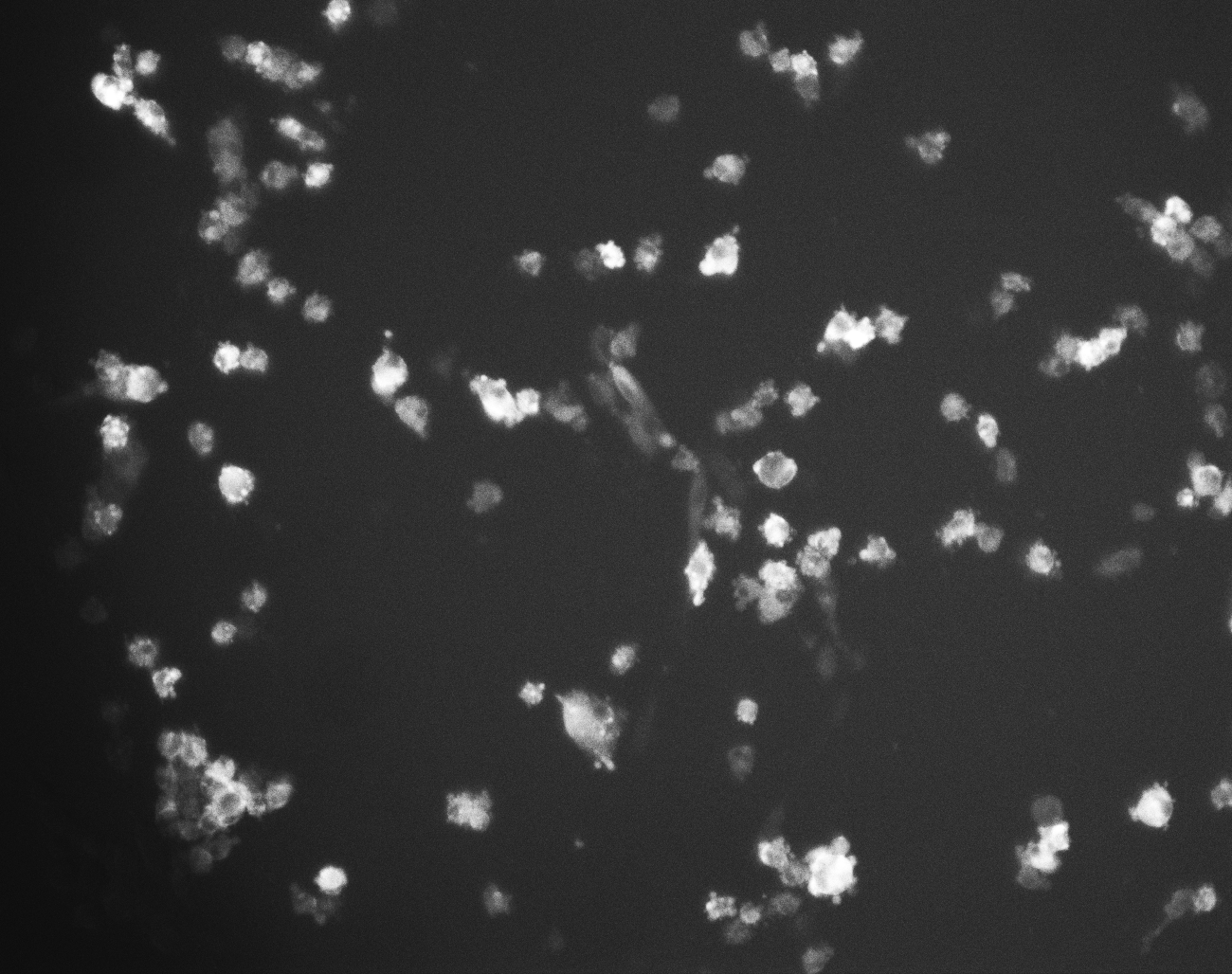

Supplement: S1 File — (ZIP) [file pone.0272206.s003.zip › new/miR449b/p16.tif]

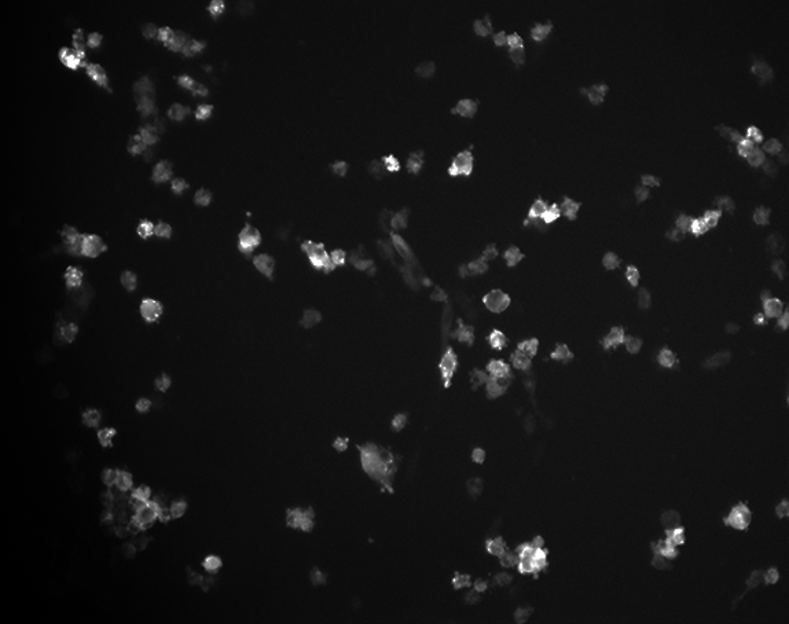

Supplement: S1 File — (ZIP) [file pone.0272206.s003.zip › new/miR449b/p16_mod.tif]

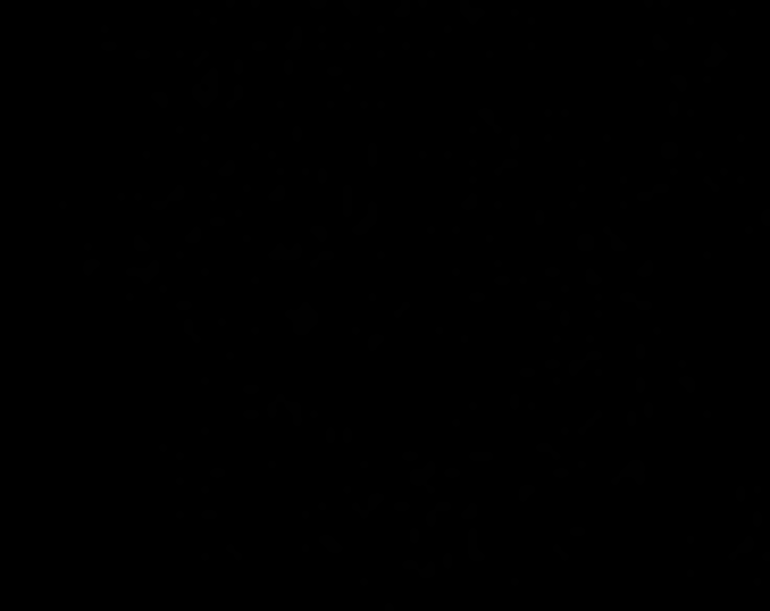

Supplement: S1 File — (ZIP) [file pone.0272206.s003.zip › new/miR517c/C - 16(fld 1 wv D360_40x - HQ460_40m)_thumb.tif]

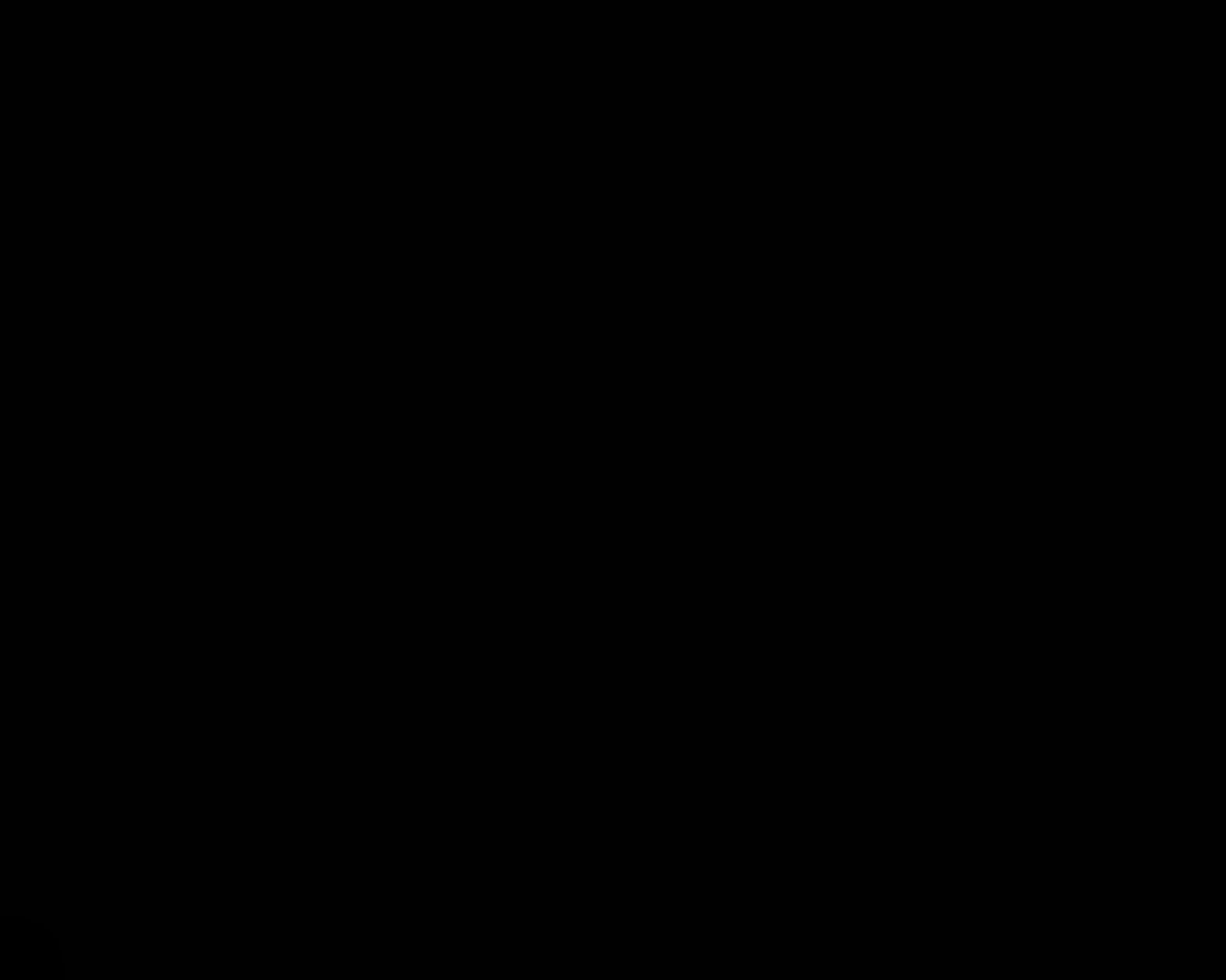

Supplement: S1 File — (ZIP) [file pone.0272206.s003.zip › new/miR517c/C - 16(fld 1 wv S475_20x - HQ535_50m).tif]

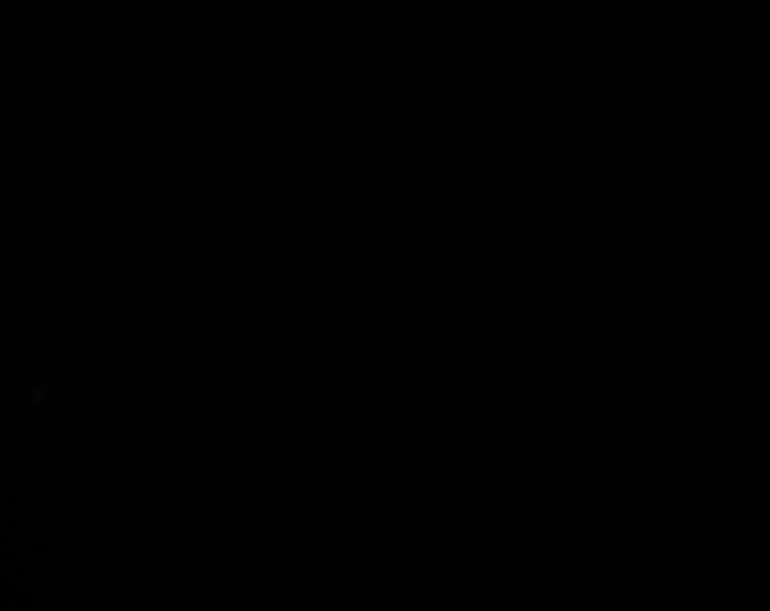

Supplement: S1 File — (ZIP) [file pone.0272206.s003.zip › new/miR517c/C - 16(fld 1 wv S475_20x - HQ535_50m)_thumb.tif]

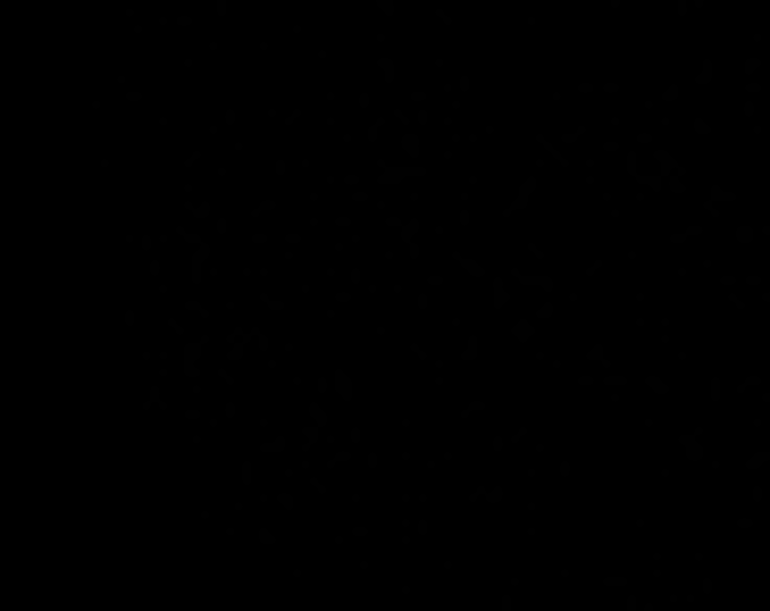

Supplement: S1 File — (ZIP) [file pone.0272206.s003.zip › new/miR517c/C - 16(fld 2 wv D360_40x - HQ460_40m)_thumb.tif]

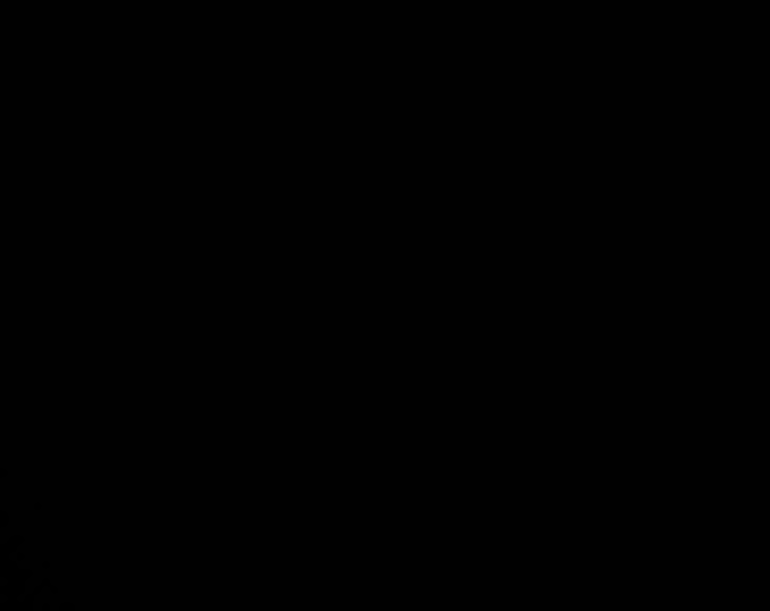

Supplement: S1 File — (ZIP) [file pone.0272206.s003.zip › new/miR517c/C - 16(fld 2 wv S475_20x - HQ535_50m)_thumb.tif]

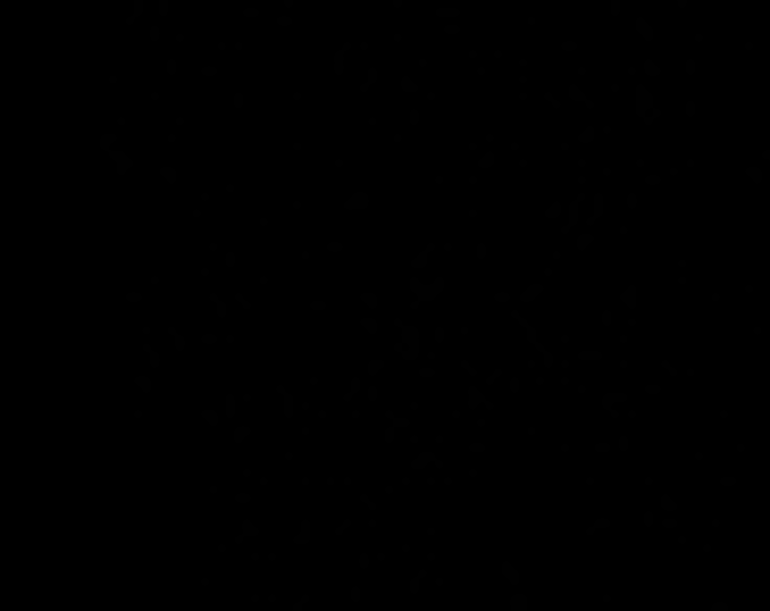

Supplement: S1 File — (ZIP) [file pone.0272206.s003.zip › new/miR517c/C - 16(fld 3 wv D360_40x - HQ460_40m)_thumb.tif]

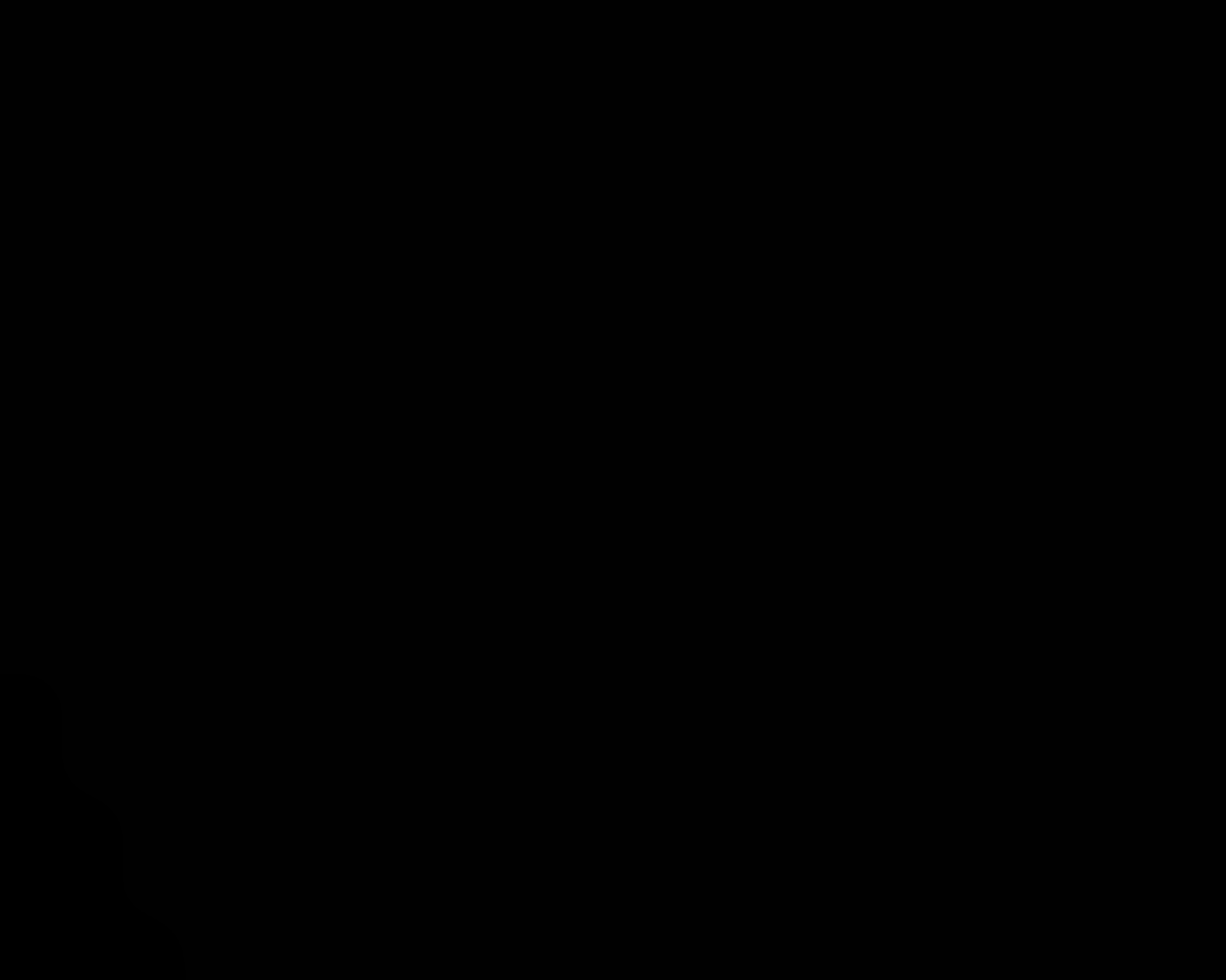

Supplement: S1 File — (ZIP) [file pone.0272206.s003.zip › new/miR517c/C - 16(fld 3 wv S475_20x - HQ535_50m).tif]

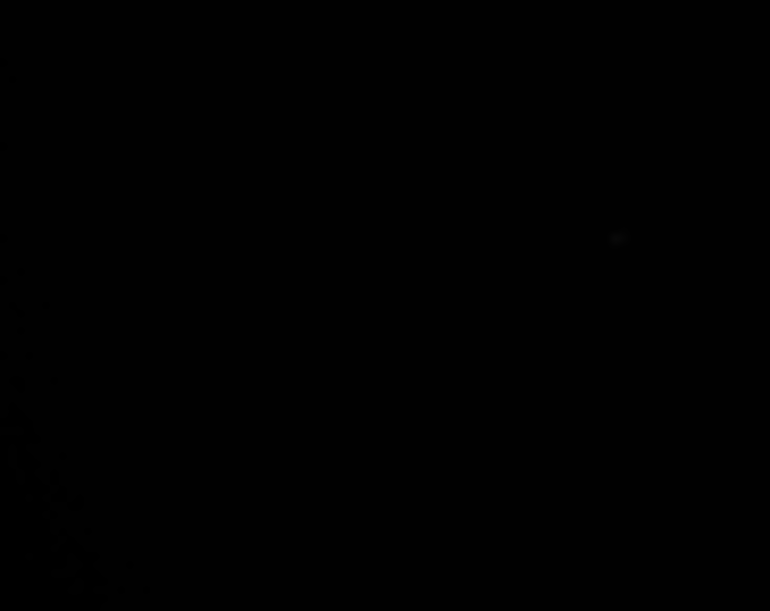

Supplement: S1 File — (ZIP) [file pone.0272206.s003.zip › new/miR517c/C - 16(fld 3 wv S475_20x - HQ535_50m)_thumb.tif]

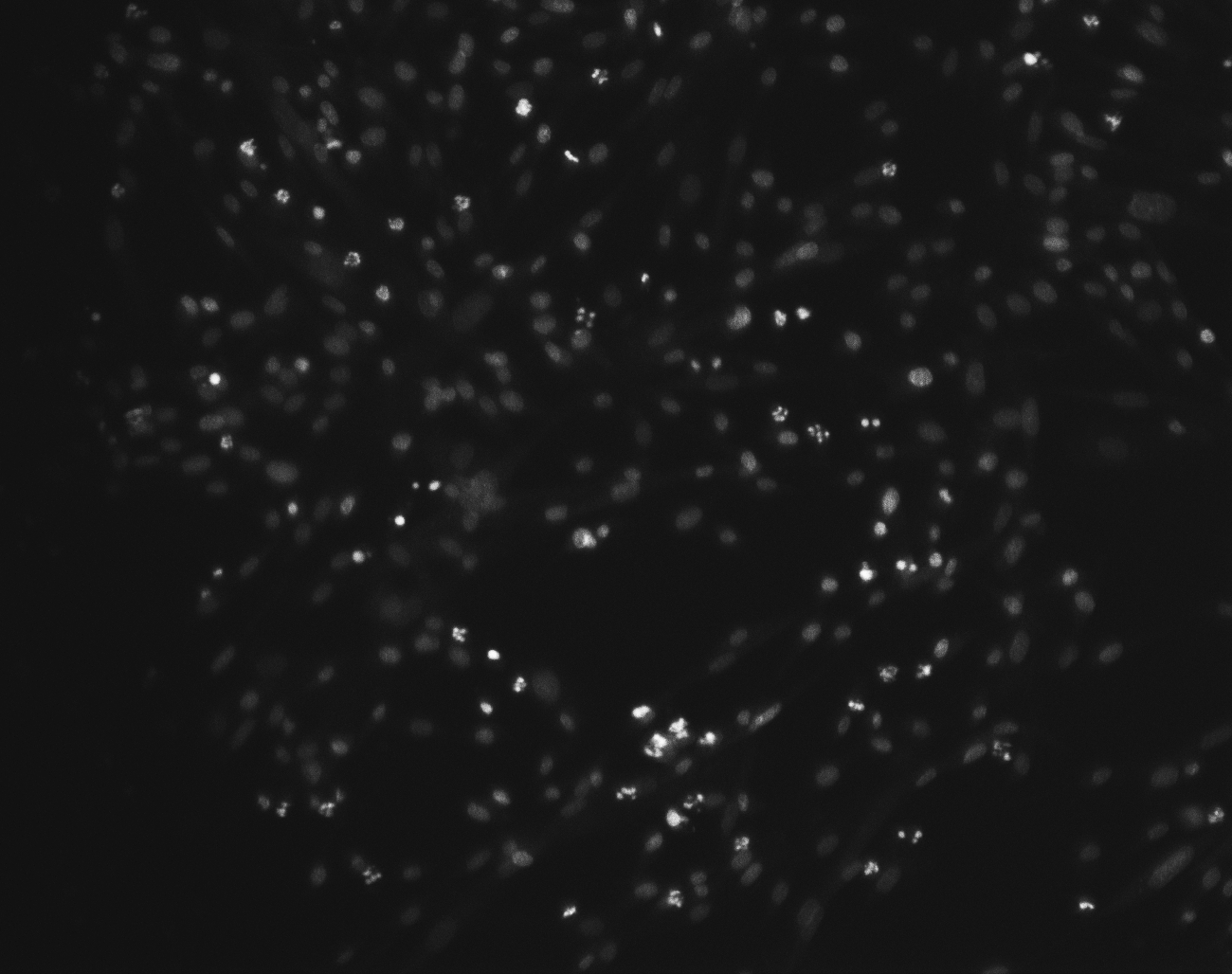

Supplement: S1 File — (ZIP) [file pone.0272206.s003.zip › new/miR517c/dapi.tif]

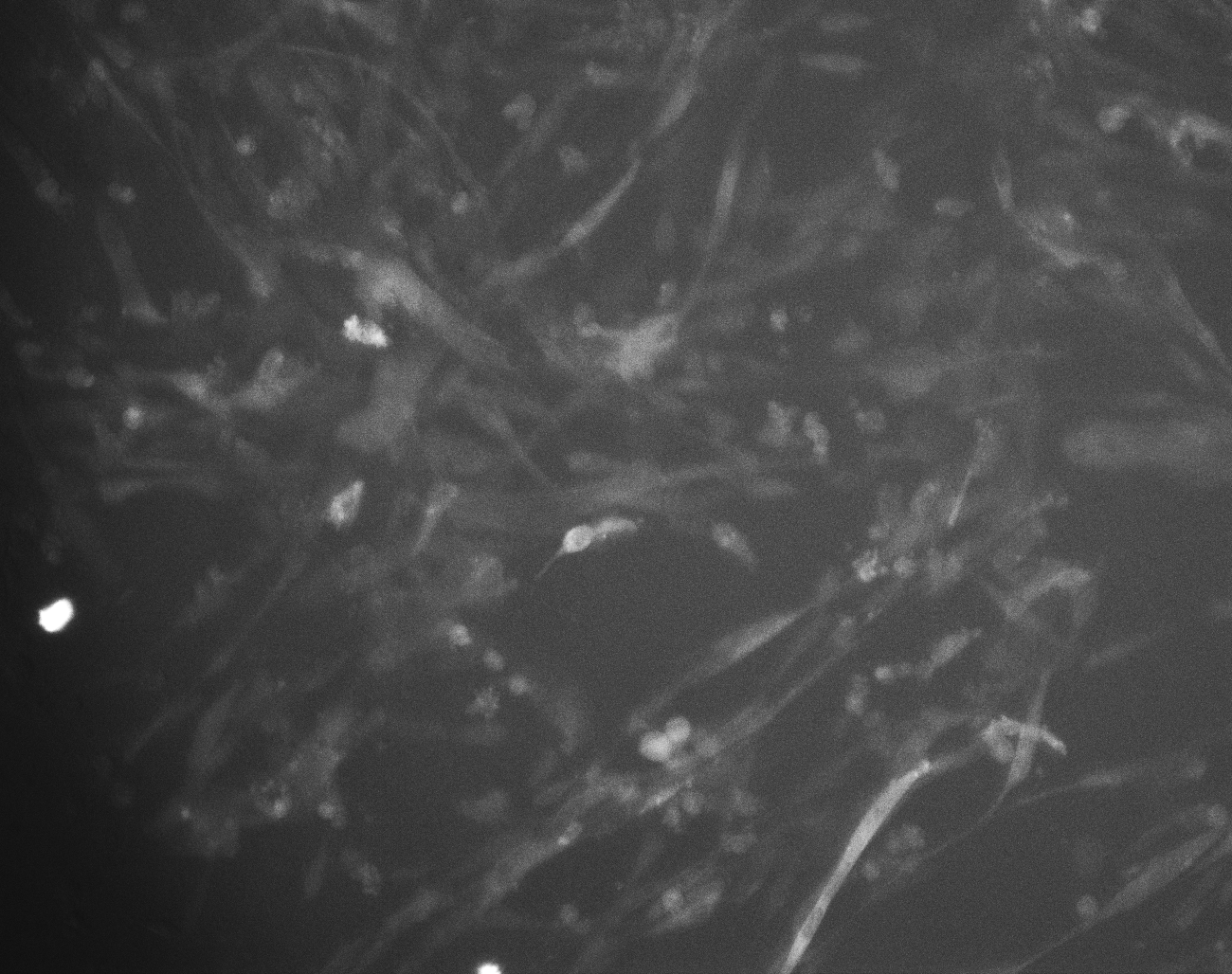

Supplement: S1 File — (ZIP) [file pone.0272206.s003.zip › new/miR517c/p16.tif]

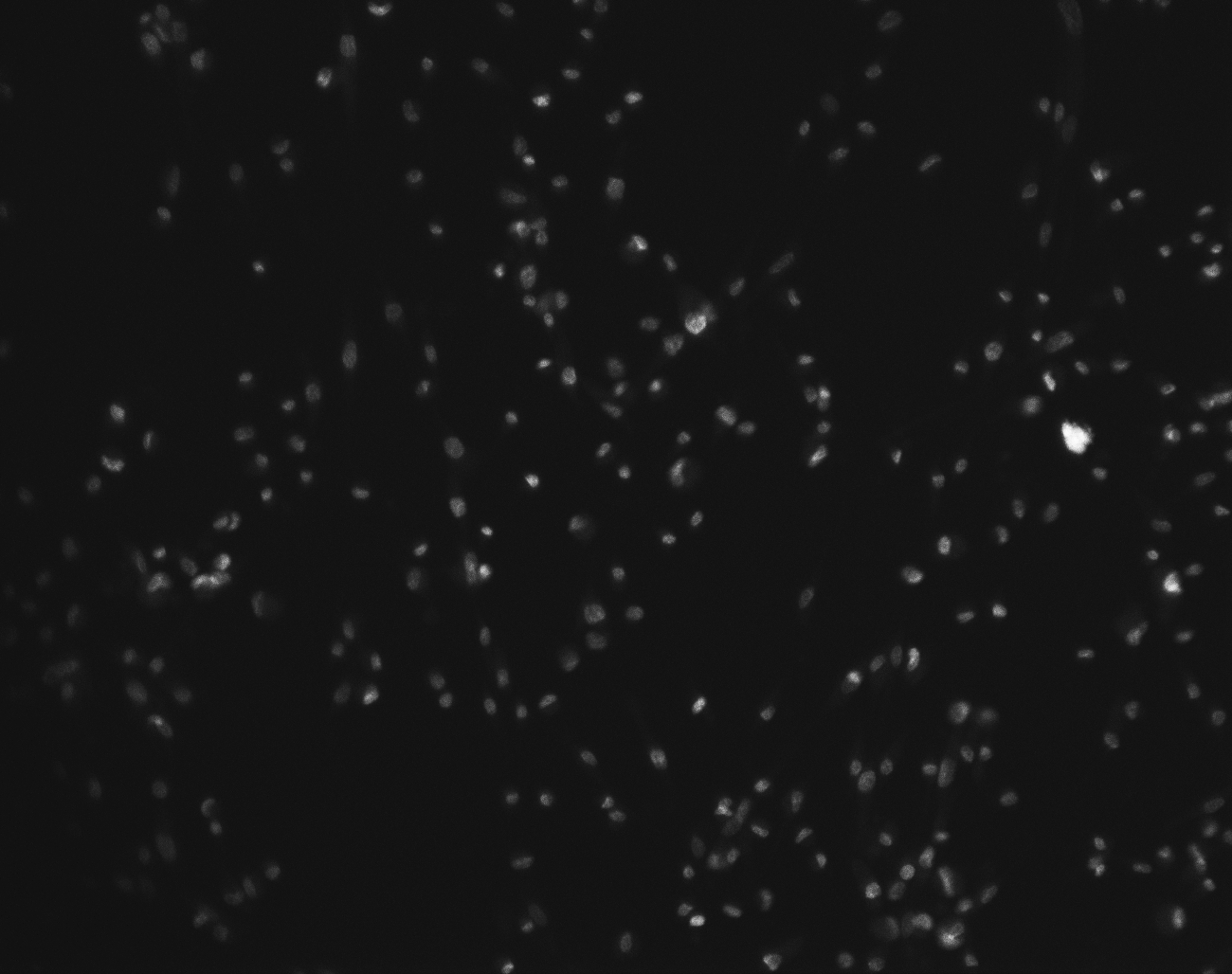

Supplement: S1 File — (ZIP) [file pone.0272206.s003.zip › new/miR557/dapi.tif]

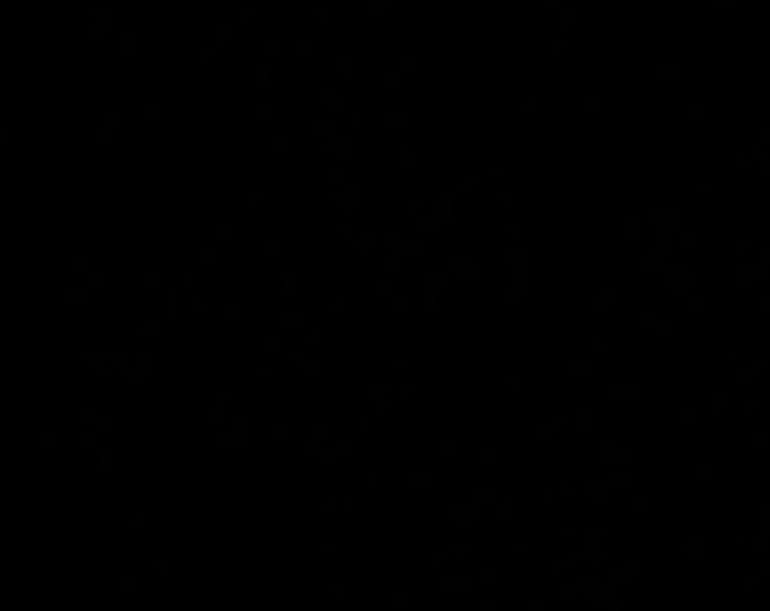

Supplement: S1 File — (ZIP) [file pone.0272206.s003.zip › new/miR557/N - 18(fld 1 wv D360_40x - HQ460_40m)_thumb.tif]

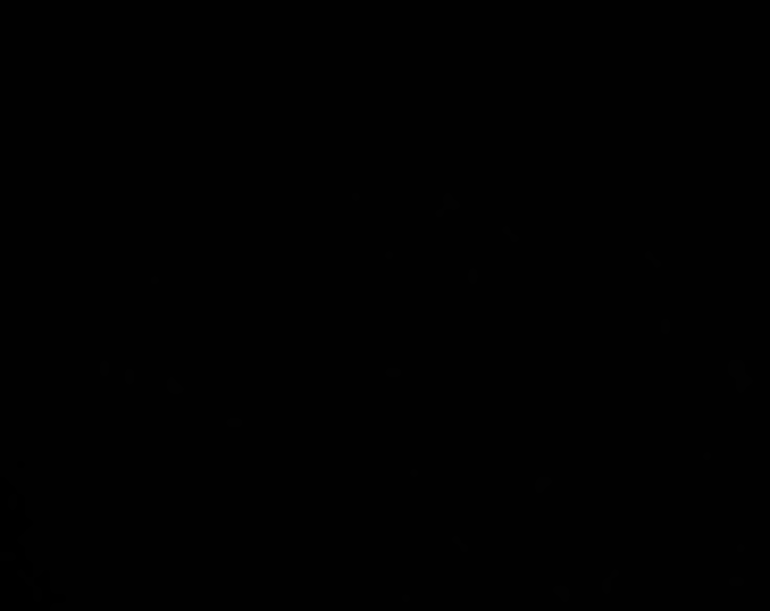

Supplement: S1 File — (ZIP) [file pone.0272206.s003.zip › new/miR557/N - 18(fld 1 wv S475_20x - HQ535_50m)_thumb.tif]

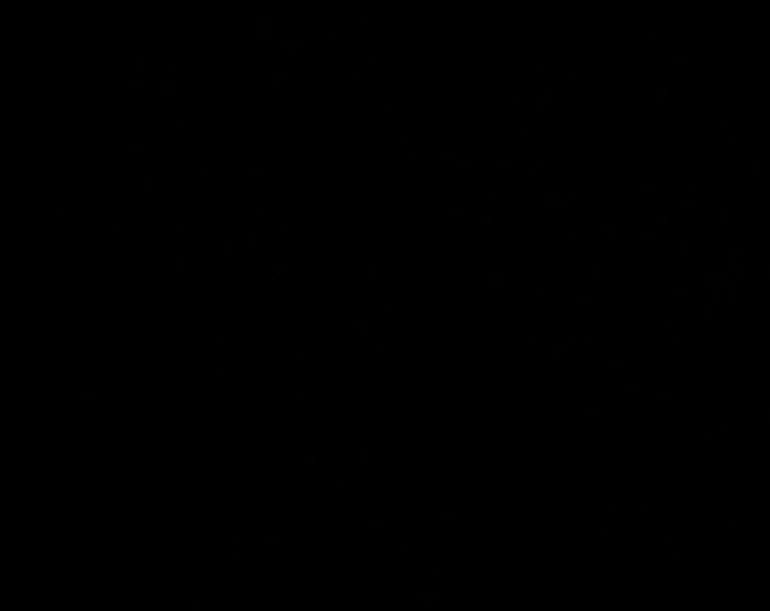

Supplement: S1 File — (ZIP) [file pone.0272206.s003.zip › new/miR557/N - 18(fld 2 wv D360_40x - HQ460_40m)_thumb.tif]

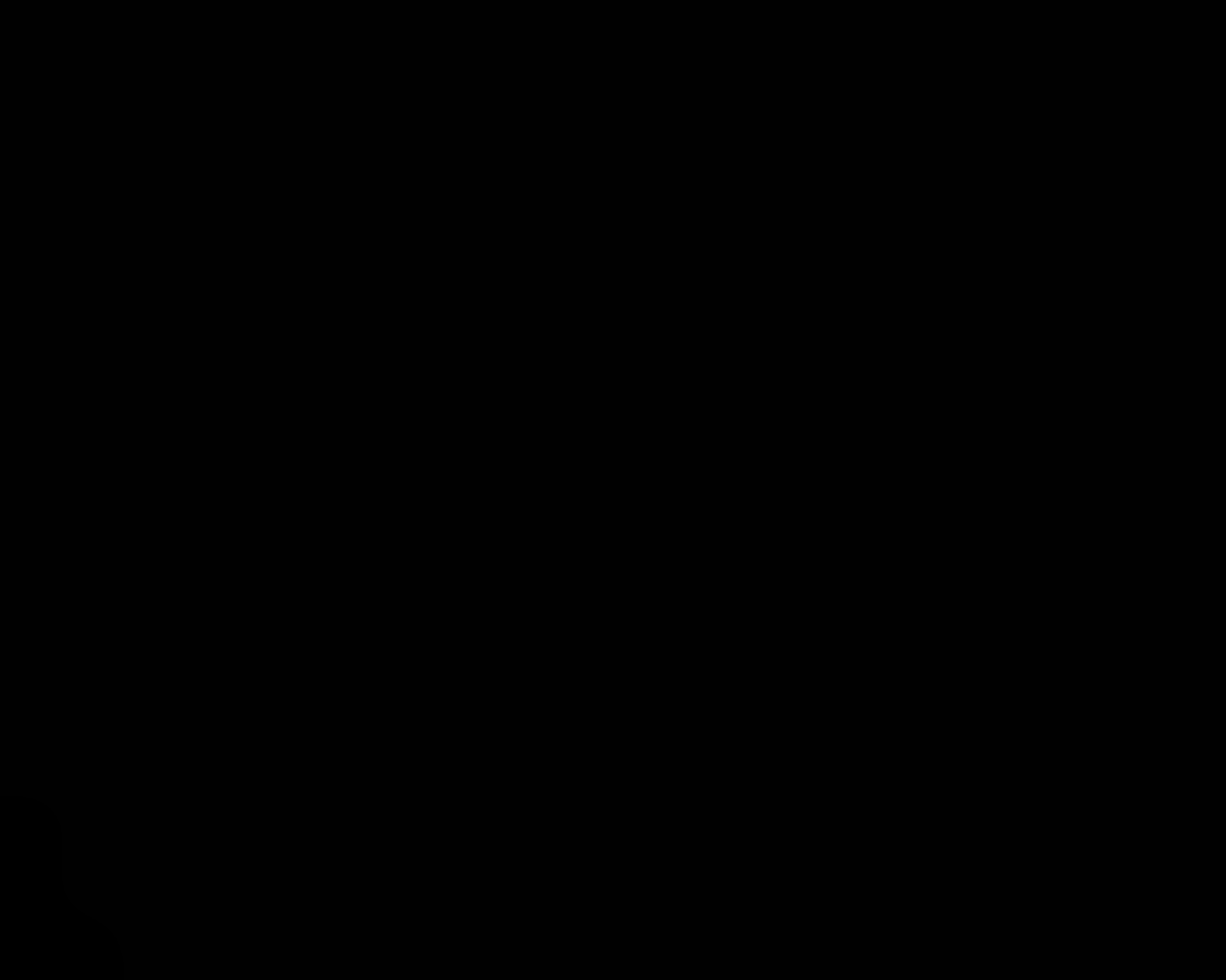

Supplement: S1 File — (ZIP) [file pone.0272206.s003.zip › new/miR557/N - 18(fld 2 wv S475_20x - HQ535_50m).tif]

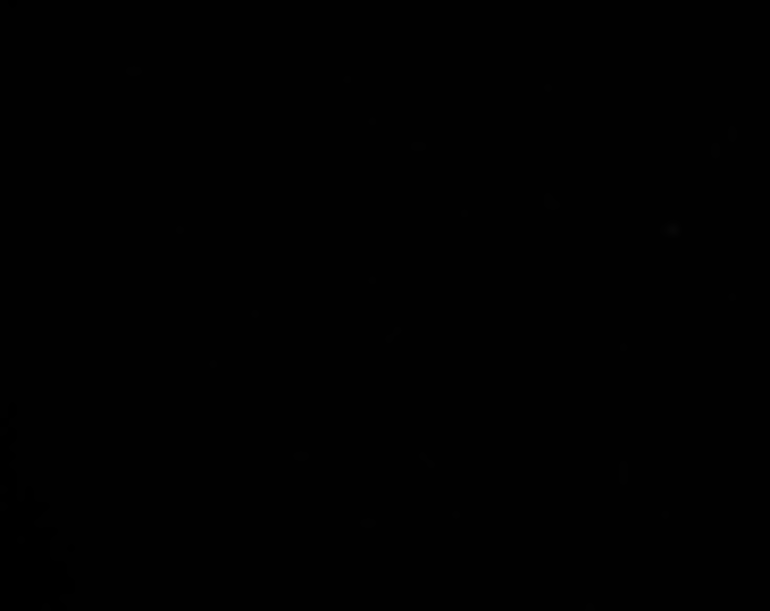

Supplement: S1 File — (ZIP) [file pone.0272206.s003.zip › new/miR557/N - 18(fld 2 wv S475_20x - HQ535_50m)_thumb.tif]

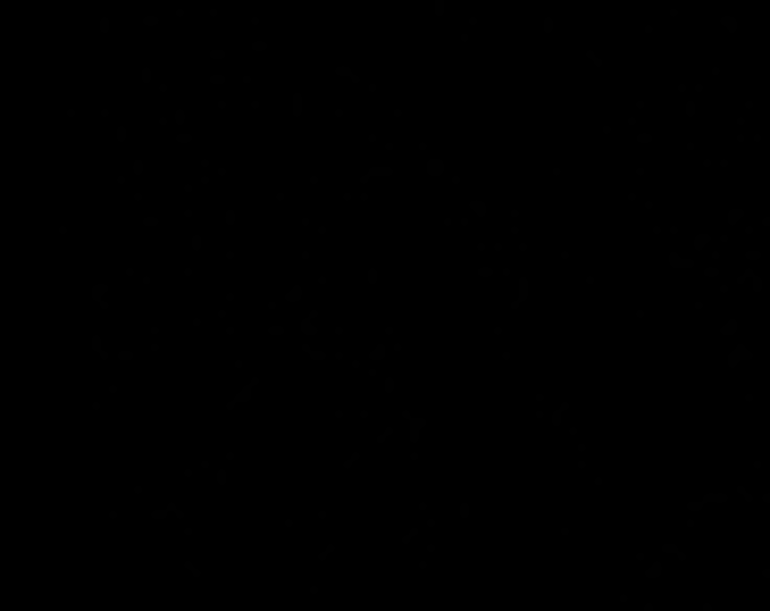

Supplement: S1 File — (ZIP) [file pone.0272206.s003.zip › new/miR557/N - 18(fld 3 wv D360_40x - HQ460_40m)_thumb.tif]

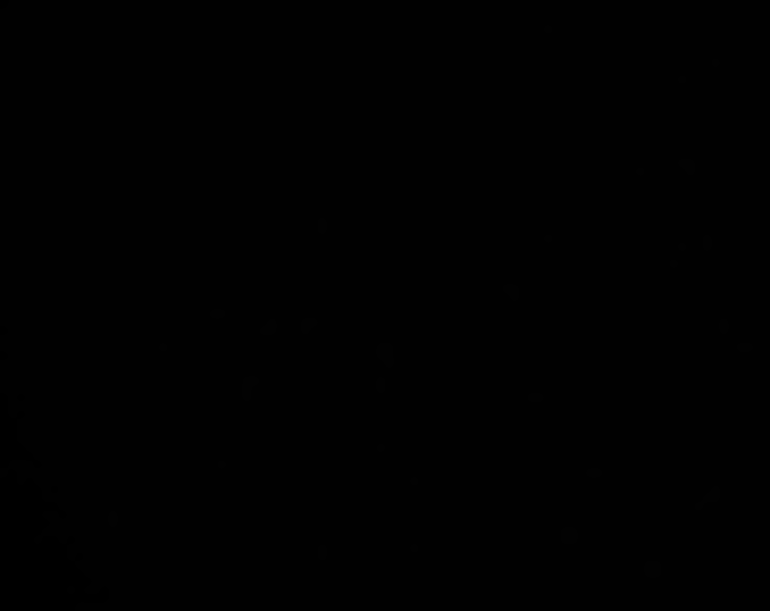

Supplement: S1 File — (ZIP) [file pone.0272206.s003.zip › new/miR557/N - 18(fld 3 wv S475_20x - HQ535_50m)_thumb.tif]
